# NAMPHORA: a fossil and modern pollen database from Northern Africa and adjacent Mediterranean and Arabian regions

**DOI:** 10.1038/s41597-026-07319-8

**Published:** 2026-05-06

**Authors:** Irene Solano, Jakob Bro-Jørgensen, Ignacio A. Lazagabaster, Chris D. Thomas, Saúl Manzano

**Affiliations:** 1 Department of Evolution, Ecology and Behaviour, University of Liverpool, Liverpool, UK; 2 Centro Nacional de Investigación Sobre Evolución Humana CENIEH, Burgos, Spain; 3 Leverhulme Centre for Anthropocene Biodiversity, Department of Biology, University of York, York, UK; 4 Quaternary Palynology Lab. Instituto de Medio Ambiente, Recursos Naturales y Biodiversidad, Universidad de León, León, Spain; 5 The Bolus Herbarium, Department of Biological Sciences, University of Cape Town, Cape Town, 7701 South Africa; 6 Área de Botánica, Departamento de Biodiversidad y Gestión Ambiental, Facultad de Ciencias Biológicas y Ambientales, Universidad de León, León, Spain

## Abstract

Northern Africa’s climate and vegetation underwent significant changes throughout the Holocene, particularly in connection with the termination of the African Humid Period ca. 5500 years ago. Fossil pollen records are key to reconstructing past environments, yet current databases for this region are limited by the omission of significant unpublished data, taxonomic inconsistencies, and the lack of standardised plant trait information. To address these issues, we introduce the Northern African, Arabian, and Mediterranean Pollen Holocene Records Archive (NAMPHORA)— a comprehensive, machine-readable and taxonomically-harmonised database compiling fossil and modern pollen records alongside plant functional traits, and ecological and phytogeographical information. This database includes all of Africa to the north of 7.52° N and constitutes the most complete and comprehensive resource (836 pollen records; 853 harmonised pollen types, and 13 key standardised plant traits) to improve palaeoecological reconstructions, enhance biogeographical analyses, and refine climate models for northern Africa during the Holocene. It enables direct data retrieval via programming languages such as R, and all datasets and code are openly available via Zenodo.

## Background & Summary

Northern Africa has undergone multiple climatic fluctuations that have significantly shaped its physical landscape and vegetation dynamics^1,2^. However, research on the region’s ecological and biogeographical dynamics remain challenging due to difficulties in reconstructing its paleoclimate, particularly during the African Humid Period (AHP: 14,800-5,500 years BP)^1–3^. Studies suggest that diverse but uncertain rainfall patterns—including tropical plumes, Mediterranean winter rains, and the Afro-tropical monsoon—governed northern Africa’s ecosystems during the AHP, particularly in the Sahara^1–4^. Plant migrations from surrounding regions^3,5–8^ transformed the area into a complex, heterogeneous landscape—a “melting pot” of plant communities^9^ thought to include Mediterranean, tropical and Arabian floral affinities^10^. Despite extensive palaeobotanical and paleoclimatic research, an integrated palaeoecological understanding of northern Africa is still lacking due to the scattering of the data through space and time leaving significant gaps remain in our understanding of the origins, past distribution, and climatic influences on northern Africa’s unique vegetation.

Fossil pollen records are among the most widely used proxies in palaeoecological studies^11^ and provide a powerful resource for reconstructing past vegetation and climate dynamics during the AHP. However, to accurately interpret fossil records, it is essential to establish robust relationships between pollen types (the palaeopalynological identification unit) and the environmental parameters they represent (e.g., vegetation composition, climate variables)^12^. This is usually achieved by correlating modern pollen rain with present-day vegetation, to extrapolate relationships between fossil pollen and past vegetation using these modern analogues^13^. Modern pollen records can also be used to identify past plant communities with no modern analogues, shedding light on how vegetation assemblage has responded to global change in unexpected, unpredictable manners^14^. Integrating modern and fossil pollen records is crucial for achieving accurate paleoenvironmental reconstructions and understanding its implications for environmental management relevant to future non-analogue climates.

From a functional ecological point of view, fossil pollen data are increasingly being integrated with plant functional traits (PFTs) to reconstruct past ecological dynamics and understand underpinning patterns of vegetation assembly^15^. PFTs represent groups of morphological, physiological, and phenological traits that influence plant survival, growth, and adaptation^16,17^. Since these traits are related to plant responses to environmental conditions, they can be used to hindcast functional changes in plant communities and to understand past vegetation shifts, and ecological processes^16^. However, no comprehensive database has yet compiled modern and fossil pollen records alongside detailed plant functional traits for the study region, hampering an integrated understanding of the long-term assembly of plant communities in northern Africa and adjacent regions.

Currently, two major pollen databases are available: the Neotoma Paleoecology Database^18^ (https://www.neotomadb.org/), which hosts global multi-proxy records and integrates several regional databases, and the African Pollen Database^19^ (APD: https://africanpollendatabase.ipsl.fr/#/home), which focuses specifically on African pollen data. While Neotoma (established in 2009) offers an accessible web interface and an R package for data retrieval^20^, it includes fewer pollen records for Africa and the Arabian Peninsula than are included in the APD (developed in 1996). Despite their considerable value, neither database covers the full range of available pollen records from northern Africa and the Arabian Peninsula, and both lack the inclusion of plant functional traits and a clearly documented taxonomic harmonisation suitable for integrating records from diverse sources. Additionally, accessing the APD can be slow and inconvenient for retrieving multiple datasets, as it does not support direct access via programming languages like R. These constrain large-scale, reproducible palaeoecological research on the region.

In addition to pollen databases, there are several plant functional trait (PFT) databases, such as the Botanical Information and Ecology Network^21^ (BIEN: https://bien.nceas.ucsb.edu/bien/) and the TRY Plant Trait Database^22^ (TRY: https://www.try-db.org/TryWeb/Home.php), which compile traits from thousands of species. However, there is no database that provides a direct linkage between pollen types and the species-level traits they represent. Pollen grains are assigned to particular morphological ‘types’, which often correspond to multiple species or higher taxa (e.g., genus or family), which can produce morphologically indistinguishable pollen grains, making trait assignment challenging. Furthermore, this process requires extensive data cleaning and processing, which is time-consuming and complicates the integration of functional traits into palaeoecological analyses.

Here we present NAMPHORA: the Northern Africa, Arabian, and Mediterranean Pollen Holocene Records Archive— a comprehensive, reproducible and open-access database that integrates both open data sources and includes a significant amount of information extracted from the grey literature. These include palaeoecological datasets, taxonomical information to harmonise palynomorph identifications (morphotypes) and a selection of key PFTs consolidated into a findable, interoperable, accessible and reusable (FAIR)^23^ machine-readable format, enabling reproducible and large-scale analyses. It will serve as the critical resource for reconstructing past vegetation and climate dynamics across northern Africa during the Holocene. All datasets and code are openly available via Zenodo^24^, and herewith we invite researchers to access, contribute to, and refine the database for future studies. All new contributions to the database will undergo a review process by our team to ensure they meet the same quality standards as the original dataset.

## Methods

**Fig. 1:**
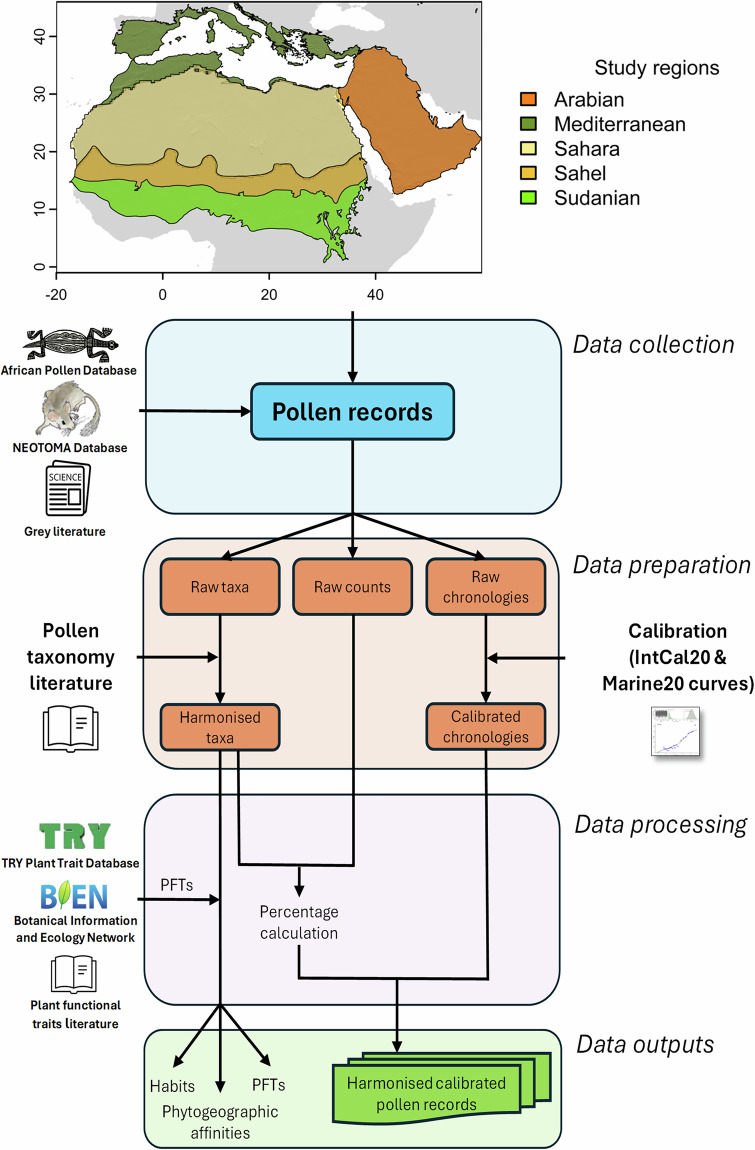
Workflow of database development.

We created a workflow for each stage of database creation, from data collection to the final database products (Fig. 1). We used R version 4.1.1^25^ for part of the data collection, and for the complete preparation and processing of the database (see details in the Zenodo repository^24^).

### Study area

**Fig. 2:**
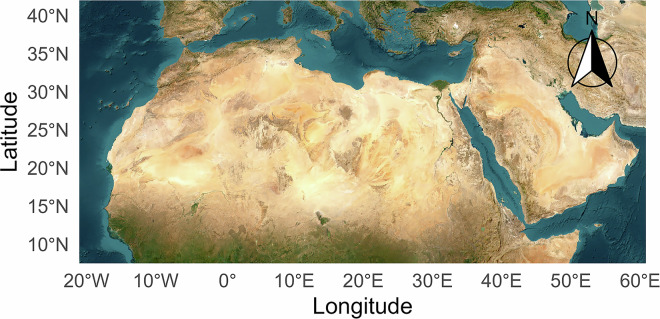
Study area.

The study area spans from 7.52° to 40.96° latitude and from −21.03° to 60.83° longitude (Fig. 2), covering northern Africa and its surrounding areas, including the Arabian Peninsula and the south of the Mediterranean region. These areas were included because of the strong floristic connections between northern Africa, southern Europe and western Asia^26^. The flora of this region reflects the interaction of several biogeographic elements, including Saharo-Arabian, Tropical-African and Mediterranean floristic components^10^. Accordingly, the spatial extent was defined to include the main ecological regions adjacent and thus influencing northern Africa vegetation. The southern boundary (7.52° N) captures the Sudanian region and the regions above, including the Sahel, Sahara and Mediterranean zones. The northern boundary (40.96° N) includes sites along the southern Mediterranean margin of Europe, while limiting the expansion of the database further into Europe in order to maintain the focus on northern Africa and exclude the influence of sub-mediterranean and temperate taxa that become more prevalent in the vegetation above 40°N^27,28^.

The study area therefore exhibits diverse vegetation types, such as deserts and xeric shrublands, Mediterranean woodlands, and tropical grasslands and savannas.

### Data collection

Pollen records— both fossil (fossilised pollen grains in sedimentary deposits) and modern (modern pollen rain, collected since the 1980s) —from the Mediterranean, Arabian Peninsula, Saharan, Sahelian and Sudanian regions were extracted from Neotoma^18^ (https://www.neotomadb.org/) and the African Pollen Database^19^ (APD: https://africanpollendatabase.ipsl.fr/#/home). Additional records, either from grey literature or openly unavailable records were obtained directly from the authors. Although Neotoma integrates several regional databases, including the European Pollen Database (EPD)^29^ and the African Pollen Database (APD)^19^, we also consulted the APD directly to ensure complete coverage of Saharan records, as some datasets in the APD have not yet been integrated into Neotoma at the time of compilation.

To extract the records from the Neotoma database, we used the *neotoma2* R package v.1.0.5^20^, using the dataset IDs that were manually retrieved from the Neotoma website. For standardisation and comparison, the Neotoma records were modified to match the structure of the APD (see the “*01_pollen_list.R”* script in the Zenodo repository). In contrast, the records from the APD were manually retrieved from the APD website (https://africanpollendatabase.ipsl.fr/#/home). Information for each pollen record, including geographic coordinates, record type, source reference(s), and the URLs to the Neotoma and APD databases where the corresponding records are stored, is provided in the file “*database.csv*” located in the metadata folder of the repository.

Additional records that were unavailable in openly accessible databases were obtained directly from the authors. Information about these records, including the authors’ details (i.e. the name of the researcher who provided the data, the name of the organisation, and contact details) and the method of data collection, is provided in the file “*pollen_data_received_from_authors_information.csv*”, located in the metadata folder of the repository.

This database also includes chronological data (uncalibrated radiocarbon dates), which were obtained alongside the pollen records in the same repositories (APD or Neotoma), retrieved from independent research articles, provided by the authors, or sourced from Giesecke *et al*.^30^. Information about the origin of the chronological data and the calibration method is provided in the file “*calibration_information.csv*”, located in the metadata folder of the repository. The current temporal coverage of data available spans the entire Holocene. Only a few records (4 in total) extend into the Middle Pleistocene (up to approximately 240,000 years BP), highlighting the limited availability of older datasets.

Additionally, plant functional traits were collected from trait databases, specifically from the Botanical Information and Ecology Network^21^ (BIEN: https://bien.nceas.ucsb.edu/bien/) and TRY Plant Trait Database^22^ (TRY: https://www.try-db.org/TryWeb/Home.php). Since the BIEN database has an associated R package^31^ (*Bien* R package v. 1.2.7), trait data from this source were collected and processed in R, and the results were stored in the processed data folder. In contrast, the TRY database does not provide an R package for direct data access, so the “leaf type” trait data taken from this source were manually extracted from the website (https://www.try-db.org/TryWeb/Prop0.php), using the “Request by traits/species” interface. The downloaded dataset was stored in the raw data folder, after which data cleaning and processing were performed.

All raw data, including the original pollen records, radiocarbon dates and pollen counts, are stored in the repository’s raw data folder to ensure transparency and reproducibility. All data are legally accessible and reusable, having been obtained from public CC-BY databases or from contributors with permission.

### Data preparation

#### Taxonomic harmonisation

Given the large geographic coverage of this database, the different pollen extraction protocols, and the varied taxonomical approaches to pollen identification, which depend largely on the pollen analyst and the region of study, standardisation of the pollen taxonomy and nomenclature is crucial to allow for the demarcation of comparable pollen taxonomical units (hereafter pollen types). In palynological analysis, the name given to a certain pollen grain refers to a pollen type (a morphological pollen category), rather than a plant systematic taxon, as multiple species, often related but sometimes in different genera or families, can produce morphologically indistinguishable pollen grains^32^. Hence, different analysts may use different names for the same pollen morphological category (synonymy of pollen types). To address this issue, we standardised the palaeopalynological records by harmonising the pollen taxonomy and nomenclature. First, we extracted the raw pollen taxa from all fossil and modern pollen records and compiled them in a list. Taxonomical harmonisation was then based on fully referenced pollen morphological information in original pollen taxonomical works (including detailed descriptions, images, keys and pollen type taxonomies), as well as our expert knowledge, to build FAIR pollen taxonomical and nomenclatural entities (pollen types) with a homogeneous nomenclature at the family, genus, species and pollen type levels. For pollen type nomenclature, we followed the nomenclatural standard proposed by Joosten and de Klerk^32^. A complete description of the harmonisation protocol will be provided in Manzano *et al*. (in prep.).

Additionally, we included other not as comprehensive pollen harmonisation proposals, including Lézine *et al*.^12^, Mottl *et al*.^33^ and the African Pollen Database taxonomy list (https://africanpollendatabase.ipsl.fr/#/taxon-dict) to enhance interoperability of the data. The standardised list of harmonised pollen taxa and the supporting palynological literature for this standardisation can be found in the harmonised taxonomy list file.

Taxonomic harmonisation was applied to each of the raw pollen data files; hence, the original pollen types were standardised to their harmonised pollen type names using the harmonised pollen taxonomy list.

#### Age calibration

To ensure chronological consistency, all radiocarbon dates were recalibrated to calendar years with the most recently updated calibration curves IntCal20^34^ and Marine20^35^ for terrestrial and marine archives respectively. For records that reported two or more radiocarbon dates, we employed Bayesian age-depth modelling using the *rbacon* R package v.3.2.0^36^. In cases where sites had only one dated depth or lacked depth information, we calibrated radiocarbon ages using the probability distribution method implemented in the *rice* R package v. 1.0.0^37^. In fossil pollen records containing post-bomb dates (i.e. after 1950CE) at shallow depths, the percent modern carbon (pMC) values were converted to 14 C dates using the *pMC.age* function within the *rice* package, and then calibrated with the corresponding post-bomb calibration curves^38^ (NH zone 1, zone 2 or zone 3), determined through http://calib.org/CALIBomb/. When available, we accounted for reservoir effects by applying the reported delta values and incorporated hiatuses when documented in the original publication. For sites with chronologies based on tephra layers, biostratigraphic markers, or oxygen isotope dates, we retained the original ages.

### Data processing

#### Plant species corresponding to each pollen type

From the harmonised pollen taxonomy list, each pollen type has been linked to all the potential plant sources extant in the study area. Since pollen types are morphological categories and not plant taxonomic units, depending on the case, a pollen type may source from any plant in a family (e.g., Annonaceae), in a group of genera (e.g., *Acacia* type, which includes the species in the genera *Acacia*, *Senegalia* and *Vachellia*), in a group of species (e.g., *Plantago major*/*media*, including both *P. major* and *P. media*) or even just from a single species (e.g., *Adansonia digitata*). Thus, to assign plant taxa to pollen types, we consulted the palynological literature to identify the plant species ascribable to each pollen type, considering only angiosperm and gymnosperm pollen types. We then used the function *name_lookup* from the R package *rgbif* v. 3.8.0^39^ to retrieve all species names of plant taxa at genus and family level source to every particular pollen type. This resulted in a preliminary list of species corresponding to the taxa assigned to each pollen type.

Next, we discarded those species that were not present in the study area by checking their current distributions in the Global Biodiversity Information Facility (GBIF)^40^. Because retrievals from GBIF via R are limited to a maximum of 100,000 species at a time, it was not feasible to process the full species list directly, as doing so would require excessive time and computational resources. Instead, we extracted a species list filtered by our study area (polygon) directly from the GBIF website^40^. We then matched this GBIF species list with our own species list to exclude species absent from the study area. Plant taxonomy followed the GBIF Backbone Taxonomy^41^.

This process resulted in a final list of pollen types with their corresponding plant sources present in the study area. This list was manually inspected and cross checked against the Kew Botanic Gardens Plants of the World online website (POWO; https://powo.science.kew.org/), to identify introduced species and correct potential errors.

#### Habit categorisation

Plant habit (or growth form) is a key ecological strategy of the plant in response to the surrounding environment^42^. Plant habit categories are a classification of plants according to their morphology, mechanical architecture and development^43^ and are strictly correlated with the abiotic and biotic conditions in which they develop^44^. We assigned plant habits to pollen types by considering all source plants to each pollen type. For example, if a pollen type includes two genera (e.g., *Erythrococca/Micrococca* pollen type includes all the *Erythrococca* and *Micrococca* species), we assigned a plant habit to the pollen type based on the habits of the species circumscribed. When the genera exhibit different habits (e.g., shrub for *Erythrococca* and herb for *Micrococca*), both habits were included in the classification.

Therefore, the harmonised pollen types were sorted into separate groups based on their habit. Two main classifications were conducted: one based on habit diversity (such as trees, shrubs and herbs, as well as forms like epiphytes, or lianas) with 35 categories (Table 1: column b), and another based on competition for space (i.e. trees, shrubs and herbs vs aquatic species) with 17 categories (Table 1, column b). The latter classification was used to calculate summary pollen percentages afterward. To assess the most probable habit of each harmonised taxon, we consulted POWO (https://powo.science.kew.org/), as well as relevant research articles and regional floras, to examine the habit of the plants included in the corresponding pollen type.

**Table 1:** Habit categories.

(a) Habit	(b) Habit (for percentage calculation)	Description	Examples of representative taxa
Algae	Algae	Photosynthetic organisms, mostly aquatic, occurring in unicellular, colonial, filamentous or multicellular forms^58^	*Spirogyra, Botryococcus*
Anthocerotophyta	Anthocerotophyta	Small and thalloid bryophytes with a horn-like sporophyte^59^	*Anthoceros*
Aquatic	Aquatic	Vascular plants adapted to life in or on water, typically with specialised floating structures^60^	*Myriophyllum, Nuphar*
Bryophyta	Bryophyta	Non-vascular plants forming mats or cushions, with a gametophyte-dominated life cycle, and spores produced in a single capsule^61^	*Hedwigia, Sphagnum*
Climbers	Herbs/Shrubs	Vascular plants with flexible and vining stems that use external support structures, such as aerial roots, to grow vertically^62^	*Hedera, Clematis*
Climbing herbs	Herbs	Herbs with climbing habit.	*Luffa, Phaseolus*
Climbing shrubs	Shrubs	Shrubs with climbing habit.	*Baissea*
Fern	Fern	Vascular, spore-producing plants with rhizomatous stems, curled fronds, and free-living gametophytes^63^	*Adiantum, Equisetum*
Fungi	Fungi	Non-photosynthetic eukaryotes that absorb nutrients through hyphae and reproduce by spores^64^	*Apiosordaria, Sordaria*
Geophytes	Geophytes	Herbaceous plants that survive adverse seasons using underground storage organs such as bulbs, tubers or rhizomes^65^	*Allium, Crocus*
Herbs	Herbs	Non-woody plants with annual shoots and annual, biennial or perennial life cycles^66^	*Centaurea scabiosa, Plantago*
Herbs/Climbers	Herbs	Herbs, erect or climbing.	*Cucumis, Trifolium*
Herbs/Shrubs	Herbs/Shrubs	Herbs or shrubs.	*Acalypha, Artemisia*
Herbs/Shrubs/Climbers	Herbs/Shrubs	Herbs or shrubs, erect or climbing.	*Jacquemontia, Vigna*
Herbs/Shrubs/Lianas	Herbs/Shrubs	Herbs or shrubs or lianas (woody climbers).	*Stephania, Cucurbitaceae*
Herbs/Shrubs/Trees	Herbs/Shrubs/Trees	Herbs or shrubs or trees.	*Abutilon, Ricinus*
Herbs/Shrubs/Trees/Climbers	Herbs/Shrubs/Trees	Herbs or shrubs, sometimes climbing, or trees.	*Heliotropium, Solanum*
Herbs/Shrubs/Trees/Lianas	Herbs/Shrubs/Trees	Herbs or shrubs or trees or lianas (woody climbers).	*Adenia, Mimosa*
Lianas	Shrubs	Woody, perennial vines that take root in the ground and climb up other vegetation^66^	*Tetracera alnifolia, Cissampelos*
Mangrove	Aquatic	Woody plants adapted to saline coastal environments, with leaves that excrete salt and specialised aerial roots^67^	*Avicennia, Rhizophora*
Marchantiophyta	Marchantiophyta	Non-vascular bryophytes with thalloid or frondose forms that colonise moist habitats^68^	*Riccia*
Palm	Trees	Tropical monocotyledonous plants with a single, unbranched stem; usually tall and woody, without secondary thickening^69^	*Phoenix, Elaeis*
Parasitic	Parasitic	Angiosperms that obtain resources from host plants via haustoria (specialised organs)^70^	*Cytinus, Loranthus*
Selaginellales	Selaginellales	Small plants with scale-like leaves and branched stems, often mat-forming and moss-like in appearance^71^	*Selaginella*
Shrubs	Shrubs	Woody perennial plants with multiple main stems emerging near ground level^66^	*Myrtus, Ephedra*
Shrubs/Climbers	Shrubs	Shrubs, erect or climbing.	*Jasminum, Rosa*
Shrubs/Lianas	Shrubs	Erect shrubs or lianas.	*Tinospora, Sherbournia*
Shrubs/Trees	Shrubs/Trees	Shrubs or trees.	*Nerium, Syzygium*
Shrubs/Trees/Climbers	Shrubs/Trees	Shrubs, erect or climbing, or trees.	*Allophylus, Zanthoxylum*
Shrubs/Trees/Epiphytic	Shrubs/Trees	Shrubs, sometimes epiphytic (growing on the surface of another plant), or trees.	*Vaccinium*
Shrubs/Trees/Lianas	Shrubs/Trees	Erect shrubs or lianas (a group of woody climbers) or trees	*Capparis, Periploca*
Shrubs/Trees/Lianas/Epiphytic	Shrubs/Trees	Shrubs, erect or climbing, sometimes epiphytic or trees.	*Schefflera*
Trees	Trees	Tall, woody perennial plants with a single main stem supporting a branched crown^66^	*Adansonia digitata, Faidherbia albida*
Trees/Lianas	Shrubs/Trees	Trees or lianas (a group of woody climbers).	*Pycnanthus*
Unknown/Indeterminable	Unknown/Indeterminable	Unknown or indeterminable habit due to unknown pollen grain identification.	Unknown/Indeterminable

#### Phytogeographical affinity categorisation

Phytogeographical affinity indicates the distribution of a plant species, that is, its association with a particular geographic region based on its origin, evolutionary history, and dispersal patterns^45^. As with habit categorisation, each pollen type was assigned a phytogeographical affinity category based on all taxa corresponding to the pollen type. Phytogeographical categories were assigned only to pollen types corresponding to angiosperms, gymnosperms, ferns, and bryophytes.

**Fig. 3:**
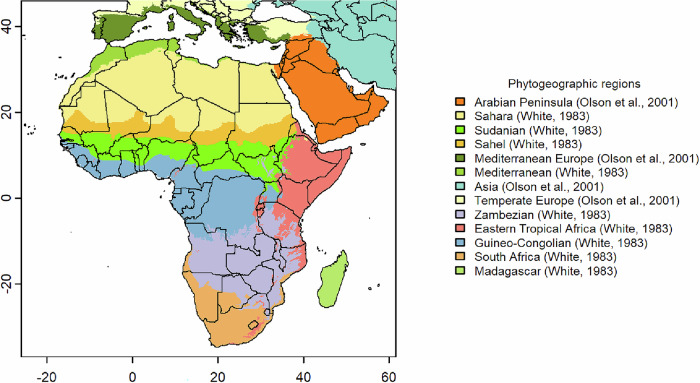
Main summarised phytogeographic regions based on White^46^ and Olson *et al*.^48^, used to categorise pollen types into phytogeographical affinities. Regions from America, Macaronesia, Australasia, and parts of Asia and Europe are excluded from the map.

To assign the most prevalent phytogeographical affinity to each harmonised pollen taxon (Fig. 3), we consulted GBIF^40^, POWO (https://powo.science.kew.org/), and the works of Watrin *et al*.^6^, Hély *et al*.^7^, White^46^, Quézel^47^ and Olson *et al*.^48^, among others, to examine the current distribution of the plants included in the pollen types. 19 phytogeographical affinity categories were established (Table 2) based on White^46^ and Olson *et al*.^48^.

**Table 2:** Phytogeographical affinity categories.

Phytogeographical affinity categories from the works of White^46^ for the African continent and Olson *et al*.^48^ for the rest of the world	Summarised phytogeographical affinity of pollen type	Description
Guineo-Congolian centre of endemism; Guineo-Congolian/Sudanian transition zone & Guineo-Congolian/Zambezian regional transition zone^46^.	Guineo-Congolian	Parent plant occurring in the Guineo-Congolian regional centre of endemism.
Sudanian regional centre of endemism^46^.	Sudanian	Parent plant occurring in the Sudanian regional centre of endemism.
Zambezian centre of endemism^46^.	Zambezian	Parent plant occurring in the Zambezian regional centre of endemism.
Sahel regional transition zone^46^.	Sahel	Parent plant occurring in the Sahel.
Mediterranean Africa, Mediterranean/Sahara transition zone^46^ & Mediterranean region (Mediterranean forests, woodlands and shrubs)^48^.	Mediterranean	Parent plant occurring in the Mediterranean region.
Sahara regional transition zone^46^.	Sahara	Parent plant occurring in the Sahara.
Karoo-Namib centre of endemism, Cape centre of endemism, Tongaland-Pondoland mosaic & Kalahari-Highveld transition zone^46^.	South Africa	Parent plant occurring in South Africa.
Eastern part of the Zambezian centre of endemism, Somalia-Masai centre of endemism, Afromontane and Afroalpine archipelagos, Lake Victoria mosaic & Zanzibar-Inhambane mosaic^46^.	Eastern Tropical Africa	Parent plant occurring in Eastern Tropical Africa (East Zambezian area, Somalia-Masai, Afromontane Archipelago, Lake Victoria or/and Zanzibar-Inhambane).
East Malagasy centre of endemism & West Malagasy centre of endemism^46^.	Madagascar	Parent plant native to Madagascar.
Azonal vegetation: Mangrove, and fresh-water swamp vegetation^46^.	African coast	Parent plant occurring in the coast of Tropical Africa, mangrove type vegetation.
Temperate Europe: Temperate broadleaf and mixed forests, coniferous forests, temperate grasslands, savannas and shrubs, boreal forests & tundra^48^.	Temperate	Parent plant occurring in temperate Europe.
Paleartic Asia & Indo-Malay: Temperate broadleaf and mixed forests, coniferous forests, temperate grasslands, savannas and shrublands, boreal forests, tundra, asian desert and xeric shrublands, montane grasslands and shrublands, tropical and subtropical moist and dry broadleaf forests^48^.	Asia	Parent plant occurring in Asia.
Arabian Peninsula: Arabian desert and xeric shrublands, Arabian xeric grasslands, savannas and shrubs^48^.	Arabia	Parent plant occurring in the Arabian Peninsula.
Neartic and Neotropic: from Tropical and Subtropical forests to Boreal forests and tundra^48^.	America	Parent plant occurring in North America and/or South America.
Australasia: from Mediterranean forests, woodlands and scrubs to deserts and tropical and subtropical forests^48^.	Australasia	Parent plant occurring in Australasia.
Canary Islands dry woodlands and forests & succulent thickets^48^.	Macaronesia	Parent plant occurring in the Macaronesia.
N/A	Cosmopolitan	Parent plant occurring in every continent.
N/A	Exotic/Introduced	Parent plant native to America, Australia or eastern Asia and has been introduced to Africa and/or Europe.
N/A	Widespread	Parent plant occurring in every continent but not present in the Sahara.

#### Plant functional traits

We selected plant functional traits commonly used in palaeoecological studies and available in public trait databases (TRY and BIEN)^3,12,16,17^. In total, we retrieved 13 plant functional traits (PFTs) (Table 3), including widely used PFTs, such as plant height, seed mass, leaf area and leaf dry mass per area (LMA), as well as less common but ecological important traits, such as flower pollination syndrome and whole plant sexual system.

**Table 3:** Plant functional traits compiled for this database.

PFT	Data type	Source	Description
Dispersal syndrome	Categorical: biotic/abiotic	BIEN database^31,72^	Dispersal of seeds, fruits or spores by animals (zoochory), by floating in water, or wind (abiotic)^17^.
Leaf type	Categorical: broad-, needle- or scale- leaved	TRY database^73–87^	Overall shape and structure of leaves. Broad-leaved species adopt more active water-use strategies, while needle-leaved species often exhibit more conservative strategies that limit photosynthesis but increases resilience under extreme conditions^88^.
Vegetative phenology	Categorical: evergreen/ deciduous	BIEN database^31,89^	Vegetative phenology distinguishes evergreen from deciduous leaf habits. Evergreen leaves are long-lived and require fewer nutrients, whereas deciduous leaves are shed to reduce water loss during dry periods, lowering maintenance costs and enabling higher carbon gain when conditions improve^90,91^.
Plant height	Continuous (m)	BIEN database^31,87,92–145^	Plant height is the vertical distance from the ground to the top of the photosynthetic tissue^17^. Greater height promotes seed dispersal and light absorption, but increases construction and maintenance costs^16,146^, thereby shaping competitive ability^147^.
Seed mass	Continuous (mg)	BIEN database^31,103,104,107,111,114–116,130,132,148–164^	Seed mass influences dispersal, colonisation and establishment^146,147^. Lighter seeds are produced in greater numbers and disperse further, whereas, heavier seeds are more beneficial for surviving the early stages of recruitment^16^.
Leaf area	Continuous (mm2)	BIEN database^31,93,97,99,104,106,108,111,114,115,125–128,134,156,165–183^	Leaf area influences photosynthesis, water balance and resource investment^146,147,184^. Larger leaves capture more light, increasing photosynthetic capacity but with higher construction costs^16^.
Habit categories (growth form)	Categorical: tree, shrub, or herb.	Literature and reference regional floras	Habit categories compiled manually for this database; used for pollen percentage calculations.
Whole plant growth form diversity	Categorical: tree, shrub, herb or aquatic.	BIEN database^31,185^	Plant growth form categories are a classification of plants according to their morphology, mechanical architecture and development^43^.
Leaf dry mass per area (LMA)	Continuous (g/mm2)	BIEN database (calculated from leaf dry mass and leaf area data)	Leaf dry mass per area (LMA) is the ratio of the dry mass of the leaf to its area^17,184^. It is related to resource allocation and leaf economy strategies, and is a key predictor of ecosystem productivity and nutrient cyclying^17^.
Whole plant sexual system	Categorical: hermaphrodite/dioecious	BIEN database^31,72^	The plant sexual system describes how sex is allocated within a species, with hermaphrodites and deciduous plants being the most common^186^. Most flowering plants are hermaphroditic and have high reproductive success, although they increase the risk of inbreeding depression. Dioecious species have lower reproductive success but avoid inbreeding depression and can live in harsher environments^187^.
Leaf life span	Continuous (months)	BIEN database^31,126,148,174,188–190^	Leaf lifespan is the period during which a leaf remains physiologically active. It reflects nutrient-use strategies, decomposition rates, and palatability, and influences both ecosystem productivity and the hydrological cycle. Short-lived leaves allocate nutrients towards high rates of photosynthesis, while longer-lived leaves tend to invest nutrients in leaf protection^17^.
Leaf nitrogen content per leaf dry mass	Continuous (mg/g)	BIEN database^31,108,111,119,120,126,127,137,148,150,167,168,170–175,178,180,183,188–210^	Leaf nitrogen concentration is calculated by dividing the total amount of nitrogen in the leaf by its dry mass. This trait is important for protein and nucleotide synthesis^17^.
Longest whole plant longevity	Continuous (years)	BIEN database^31^	This trait reflects the environmental adaptation of plant communities, and how they respond to biotic and abiotic stresses^17^. Short-lived plants have rapid growth, while long-lived plants have slower growth and live in harsh conditions with low resources^17^.
Flower pollination syndrome	Categorical: biotic/abiotic	BIEN database^31,72^	Floral pollination syndrome is the set of floral traits that are linked to a specific functional group of pollinators^211,212^.

Traits were extracted from the BIEN and TRY databases, with the original sources acknowledged alongside each trait. In addition, habit categories were compiled from the literature and regional floras specifically for this database.

**Fig. 4:**
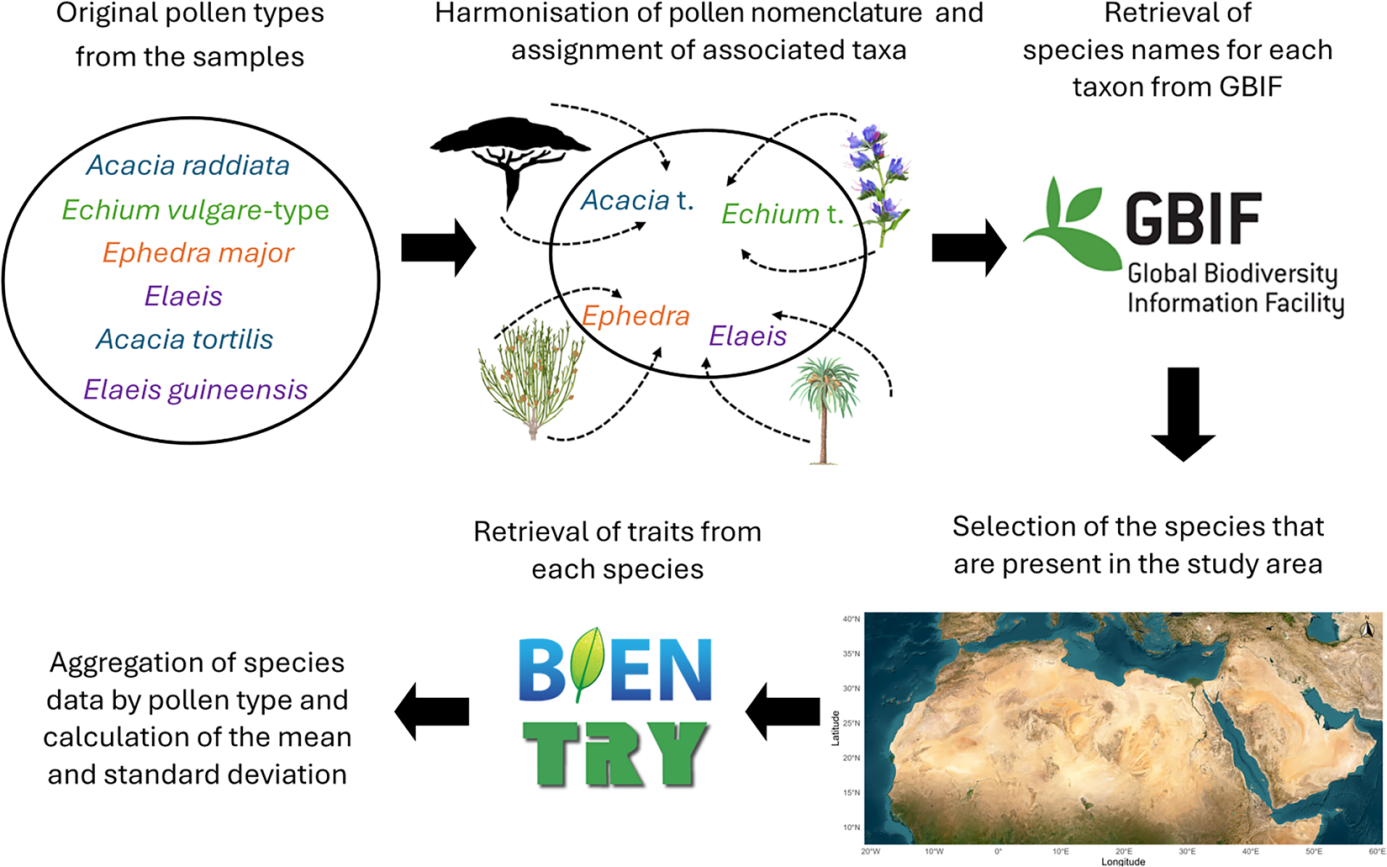
Workflow for assigning plant species to pollen types and processing plant functional traits.

The processing of plant functional traits was conducted in several steps (Fig. 4), following the methodology outlined by Brussel and Brewer^16^. From the final list of pollen types and their corresponding species, we extracted all available traits from all the species in the list. Continuous trait measurements were log10-transformed and normalised to approximate a normal distribution and reduce the influence of outliers. For species with multiple observations, we calculated the mean and standard deviation. Finally, for each pollen type, we calculated the mean value of each trait across all associated species, so that we had a final matrix of pollen types with their averaged traits.

12 categorical and continuous traits were obtained from the BIEN database^21^ using the *Bien* R package v. 1.2.7^31^. Since the leaf dry mass per area (LMA) trait is not directly available in the BIEN database, but its components (leaf dry mass and leaf area) are, we calculated LMA by dividing leaf dry mass by leaf area for each taxon that had data for both traits. The categorical trait “leaf type” was obtained directly from the TRY database^22^ website (https://www.try-db.org/).

#### Calculation of percentages in harmonised pollen records

Pollen types in the original datasets were replaced by the harmonised pollen types. Raw pollen counts from the harmonised pollen records were transformed into percentages to remove the effect of sampling and counting effort^49^ on the basis of the terrestrial pollen sum, including trees, shrubs, and herbs (Table 1, column b). Next, we calculated the total sum, which includes all habit types—terrestrial, parasitic, aquatic, algae, bryophytes, ferns, fungi, and indeterminate or unknown taxa (Table 1, column b).

Percentages were then calculated in relation to the pollen sum, except for indeterminate or unknown pollen types, which were calculated relative to the total pollen sum (all palynomorphs included). The harmonised pollen records with percentage values were subsequently archived in the processed data folder providing a valuable resource for the examination of site-to-site vegetation dynamics.

#### Addition of calibrated dates to harmonised pollen records

To ensure temporal consistency across the dataset, only the median recalibrated dates were extracted and associated with their corresponding harmonised pollen records. Consequently, each harmonised pollen record presented the median calibrated age for each depth, alongside its harmonised pollen taxa. This allows for consistent chronological comparison across sites.

## Data Records

The NAMPHORA database is available via Zenodo^24^. It includes pollen records, chronological data in raw radiocarbon dates and calibrated calendar years, a pollen taxonomic harmonisation list, habit and phytogeographical categorisation, and plant functional traits retrieved from TRY and BIEN databases, for the harmonised pollen taxonomical entities. All files, metadata, data outputs and R scripts are stored under a CC-BY license.

All datasets have associated references, which can be found alongside the processed data files in a reference column. Moreover, a complete list of references associated with each dataset is provided in the Supplementary Information file, together with an acknowledgment of all data contributors.

In addition, the PDF documents of the original studies from which the data were extracted (e.g. pollen records, chronological data and plant functional traits), as well as the references used for taxonomic harmonisation, have been compiled and made available for download in the Zenodo repository^24^.

The database includes the following data entries:

### Pollen records

Pollen data is structured into three separate main folders: raw data, processed data and metadata.

1. **Raw data**: Contains individual raw pollen records (in csv format) with original taxa names, separated into fossil and modern pollen records.
2. **Processed data**: Contains individual harmonised pollen records (in csv format), separated into fossil and modern pollen records, which are further divided into:
  - **Harmonised counts**: In each pollen record, the original pollen type names have been transformed into harmonised pollen type names, with counts summed according to taxa regrouping.
  - **Harmonised percentages**: In each pollen record, the original pollen type names have also been transformed into harmonised pollen type names, with counts converted to percentages.
3. **Metadata**: A list with geographical, temporal and other relevant information of all the pollen records has been compiled in the “*database.csv*” file. Specific metadata details are available in the “*pollen_metadata.csv”* and “*pollen_metadata.html”* files. The metadata includes several key information:
  - **Site location**, records the site name (both original and modified name for machine readability), country, biogeographic area, latitude and longitude in decimal degrees, and altitude in meters.
  - **Data source**, details whether pollen records were retrieved from Neotoma, the APD, or directly from authors if were not publicly available. The *Link to database* field provides a direct link to the data repository where each site was retrieved. The *dataset_id* field stores the Neotoma dataset identifier for downloading pollen records via the *neotoma2* R package^20^. Finally, the *Database* field indicates whether a site is available in a public data repository or was obtained directly from the authors.
  - **Record type** specifies whether the pollen record is modern or fossil.
  - **Archive type** indicates the type of core, such as terrestrial, lacustrine, archaeological, etc.
  - **Chronological information** includes details on whether a site has an associated chronology (*Dated*) and whether raw radiocarbon dates have been recalibrated using updated calibration curves (*Recalibrated*). Sites are classified by the geological time period, including the Late Holocene (Meghalayan: 0 to 4.2 ka BP), Mid Holocene (Northgrippian: 4.2 to 8.2 ka BP), Early Holocene (Greenlandian: 8.2 to 11.7 ka BP), Holocene (0 to 11.7 ka BP), Holocene-Pre-Holocene (present to 240 ka BP) and Pre-Holocene (11.7 to 240 ka BP). Additionally, for each dated fossil site, the youngest age (M*inimum mean age*), the oldest age (*Maximum mean age*) and whether the site coincides fully or partially with the African Humid Period (14.8 to 5.5 ka BP) are detailed.
  - **Bibliographic references** include the original studies from which the data originate.

### Chronologies

Chronological data is structured into three separate main folders: raw data, processed data and metadata.

1. **Raw data**: Contains radiocarbon dates for each dated fossil pollen record (in csv format) in the radiocarbon folder, and calibration information for each dated fossil pollen record in the calibration folder, which is further divided into:
  - **Bacon runs**: Includes files in csv format for those records that have been calibrated using a Bayesian age-depth model. The files are formatted so they can be calibrated with the *rbacon* v. 3.2.0 package^36^.
  - **Single depth calibration**: Includes the plot of individual calibrated dates in pdf format for each fossil pollen record that has been calibrated with the *rice* v. 1.0.0 package^37^.
2. **Processed data**: Contains the updated calibrated dates for all recalibrated pollen records.
3. **Metadata**: A list with details on the calibration information for each site has been compiled in the “*calibration_information.csv*” file. Details on specific metadata information are available in the “*calibration_metadata.csv”* and “*calibration_metadata.html”* files. The metadata documents information on the:
  - **Site location** records the site name (both original and modified name for machine readability), country and biogeographic area.
  - **Chronological information** includes the following fields:
    i. *Dated*: Whether the site contains radiocarbon dates.
    ii. *Recalibrated:* Whether the raw radiocarbon dates have been recalibrated with updated calibration curves.
    iii. *Depths*: Whether the record reports depths along with the radiocarbon dates.
    iv. *Material dated*: Material used for radiocarbon dating (e.g., wood, bulk sediment, etc.).
    v. *Dating method*: Method used for radiocarbon dating (conventional or AMS).
    vi. *Calibration curve:* Calibration curve used in the calibration process (IntCal20^34^ or Marine20^35^).
    vii. *Hiatus and reservoir effect:* Whether there was a hiatus or an effect of reservoir on radiocarbon dating.
    viii. *Post-bomb curve:* Whether there was a post-bomb date and the corresponding post-bomb curve^38^ (NH1, NH2 or NH3) used in the calibration process.
    ix. *Accumulation rate (yr/cm):* Rate of accumulation in years per cm used in the calibration.
  - **Bibliographic references** include the original studies from which the data originate.

### Taxonomy

Taxonomical data is also separated into three main folders: raw data, processed data and metadata.

1. **Raw data**: Contains three subfolders:
  - **Pollen taxonomy harmonisation lists**: Includes published harmonisation tables^12,33^ (in csv and xlsx formats) for cross-reference, and the APD taxonomy list retrieved directly from the APD website (https://africanpollendatabase.ipsl.fr/#/taxon-dict).
  - **Raw taxa list**: Contains the list of raw taxon names (in csv format) extracted from all pollen records (modern and fossil).
  - **GBIF species list**: Contains the list of plant species filtered by the study area, directly obtained from the GBIF website^50^.

1. **Processed data**: Contains the harmonised taxonomy list, the phytogeographical affinity list, the habit list, the list of taxa (family, genus or at species level) corresponding to the pollen types, the list of species names corresponding to the taxa, and the final list of filtered species associated to the pollen types (all in csv format). The harmonised pollen taxonomy list includes the original pollen type names and the consequent taxonomic harmonisation. The phytogeographical affinity and habit lists include the phytogeographical affinities and habits for each harmonised pollen taxon respectively. The list of taxa corresponding to pollen types includes all plants associated with each pollen type, either at family, genus or species level, depending on the resolution of the pollen type. The list of species names includes all the unfiltered species corresponding to each taxon associated to each pollen type. Finally, the species list includes all the filtered species corresponding to the pollen types that are present in the study area.
2. **Metadata**: Specific metadata information for the harmonised pollen taxonomy list is available in the “*taxonomy_metadata.csv*” and “*taxonomy_metadata.html*” files. In addition, there is a file (“*phytogeographical_affinity_categories.csv”*) that provides a definition for each phytogeographic affinity category (Table 2) and the corresponding references. The harmonised pollen taxonomy list metadata documents information on:
  - **Original pollen taxon** reports the original pollen taxonomic names given by the pollen analysts to the grains they identified, these are taken from the original, raw pollen records.
  - **Taxonomical information** includes an assignation of the pollen taxon to a plant taxonomical entity at the order, family, genus and or species level.
  - **Harmonised pollen taxon** refers to the standardised name assigned to each pollen taxon, based on our literature-based interpretation of palynotaxonomy.
  - **Number of pollen records** (dated fossil records, undated fossil records, modern records and total records) in which the original pollen type is present.
  - **Number of pollen records** (dated fossil records, undated fossil records, modern records and total records) in which the harmonised pollen taxon is present.
  - **Pollen type harmonisations** proposals from Lézine *et al*.^12^, Mottl *et al*.^33^ and from the APD (https://africanpollendatabase.ipsl.fr/#/taxon-dict).
  - **Pollen taxonomical references** include the original studies used for taxonomic harmonisation and for habit and phytogeographical affinity categorisations.

### Plant functional traits

Plant functional trait data is structured into raw data, processed data and metadata folders.

1. **Raw data**: Contains the leaf type trait information retrieved from the TRY database *(“leaf_type_TRY.zip”*).
2. **Processed data**: Contains the final processed plant functional traits data from both databases (BIEN and TRY) in the file *“total_pfts.csv”*.
3. **Metadata**: Specific metadata information for the final plant functional traits list is available in the “*pfts_metadata.csv*” and “*pfts_metadata.html*” files. It also contains a file (*“pfts_categories.csv”*) that provides a definition for each plant functional trait and the corresponding reference. The metadata documents information about the:
  - **Taxonomical information**: Family, genus and species name of the plant taxa corresponding to the harmonised pollen taxon.
  - **Harmonised pollen taxon**: Morphological harmonisation of the pollen taxon.
  - **Url source**: URL linking to the original data source of the measured trait.
  - **Project Pi information**: Principal investigator of the original project in which the trait was measured, and its contact information.
  - **The plant functional traits** for each pollen taxa.
  - **Bibliographic citations** include the original studies where the trait leaf-type has been retrieved (from the TRY database).

## Data Overview

**Fig. 5:**
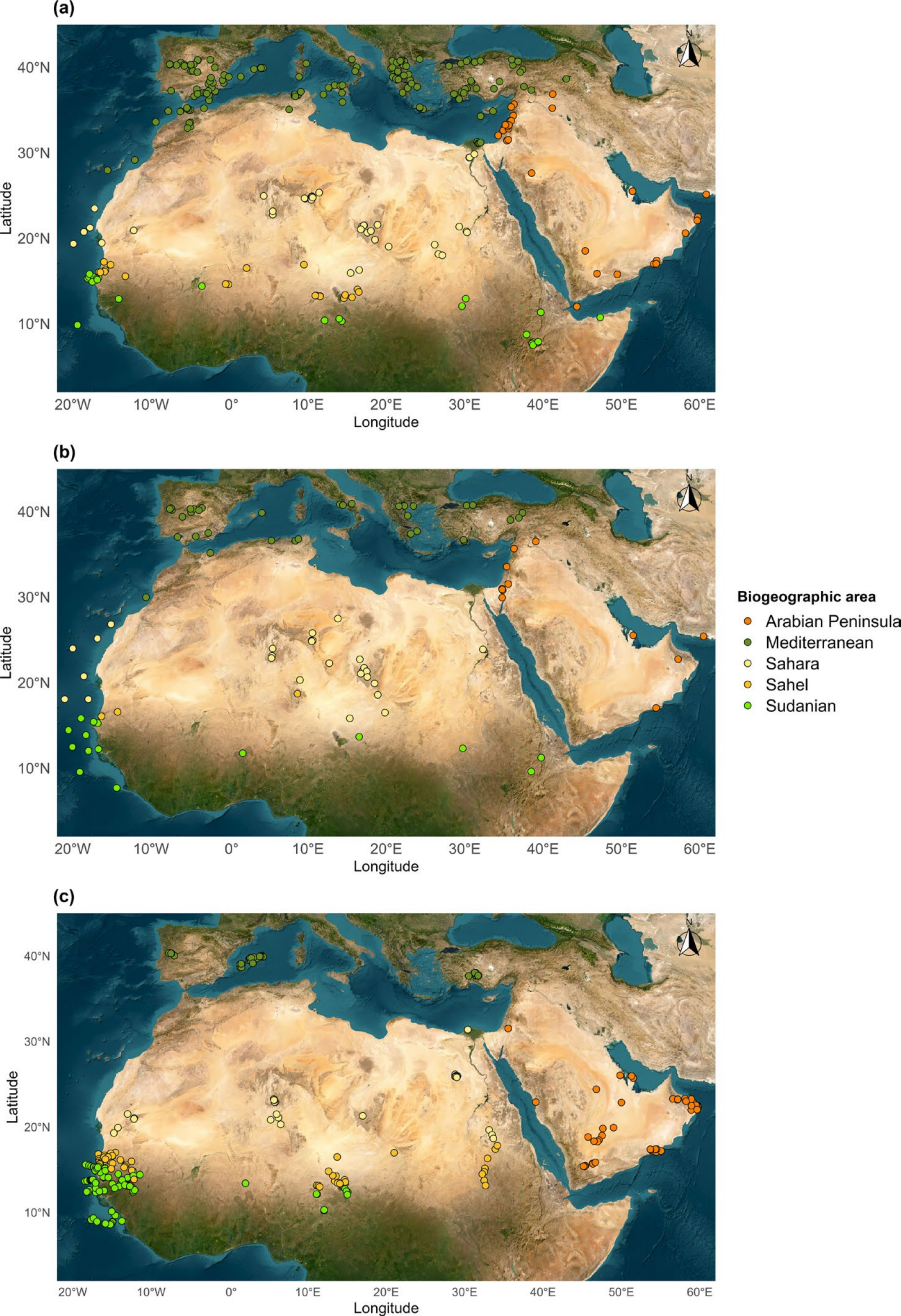
The NAMPHORA database: dated (**a**) and undated (**b**) fossil pollen records, and modern pollen records (**c**). For a more detailed inspection of the pollen records see Fig. 1 in database website (https://irene131998.github.io/NAMPHORA_database/).

**Fig. 6:**
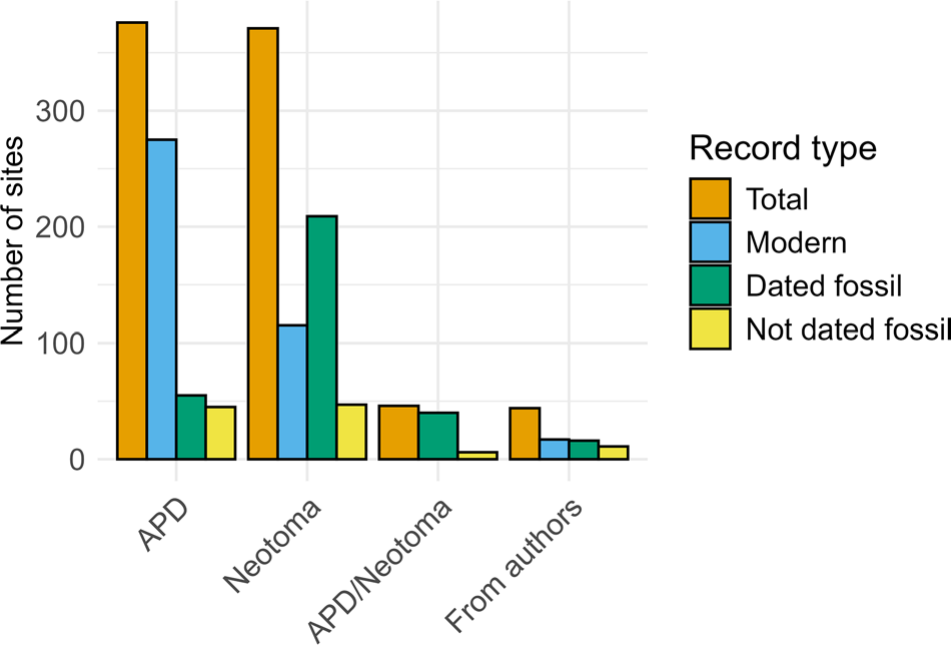
Pollen records according to source database (APD, Neotoma, present in both databases (APD/Neotoma), and received directly from the authors).

**Fig. 7:**
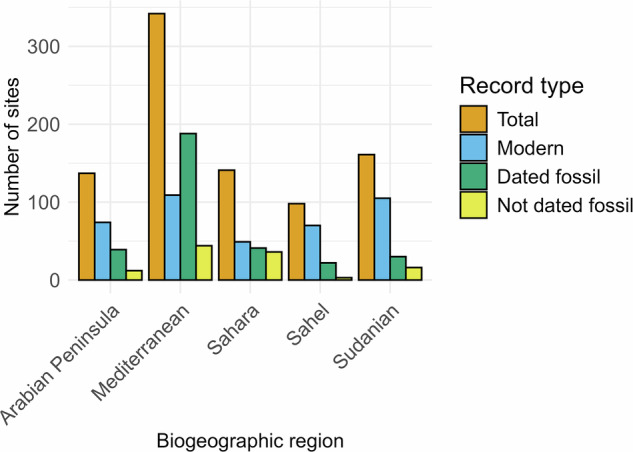
Pollen records according to biogeographic region (Arabian Peninsula, Atlantic Ocean, Mediterranean, Sahara, Sahel and Sudanian regions).

The database includes a total of 836 pollen records (Table 4, Fig. 5), which were sourced from the APD, Neotoma and individual researchers (Fig. 6). While the dataset covers a broad geographical extent, pollen records are unevenly distributed, with the Mediterranean region being by far the most studied (Fig. 7). In contrast, the Sahara, Sahel and Sudanian zones, contain relatively fewer sites, highlighting the gaps in pollen record collection in these regions. This scarcity is largely due to challenges in physical access, political and economic instability, and the limited availability of palaeoecological archives in extreme environments such as deserts^19,51^.

**Table 4:** Pollen records summary table.

Biogeographic region	Total records compiled		Fossil records with chronological data
	Modern	Fossil	
Arabian Peninsula	74	51	39
Mediterranean	109	232	188
Sahara	49	76	41
Sahel	70	25	22
Sudanian	105	45	30
Total	407	429	320

Most of the fossil pollen records relied on radiocarbon dating, which were calibrated to calendar years before present (BP). From the total of 429 fossil pollen records compiled in the database, 320 included chronological data (Table 4). A total of 277 records were recalibrated using updated calibration curves^34,35^ (Table 5). 234 records were recalibrated using a Bayesian age-depth model with the *rbacon* v. 3.2.0 package^36^ and 43 records were recalibrated with the *rice* v. 1.0.0 package^37^, due to insufficient or missing depth information. The remaining 43 records could not be updated due to missing radiocarbon dates, lack of associated errors, or because their chronologies were based on tephra or biostratigraphic timescales, and thus calibration is not needed.

**Table 5:** Fossil pollen records chronological information.

Biogeographic region	Fossil records dated but not recalibrated		Re-calibrated with Rbacon v.3.2.0^36^	Re-calibrated with rice v.1.0.0^37^	Total number of recalibrated records (Rbacon^36^ & rice^37^)
	Could not be recalibrated	Calibration not necessary			
Arabian Peninsula	1	5	29	4	33
Mediterranean	8	19	147	14	161
Sahara	3	1	19	18	37
Sahel	2	1	14	5	19
Sudanian	2	1	25	2	27
Total	16	27	234	43	277

**Fig. 8:**
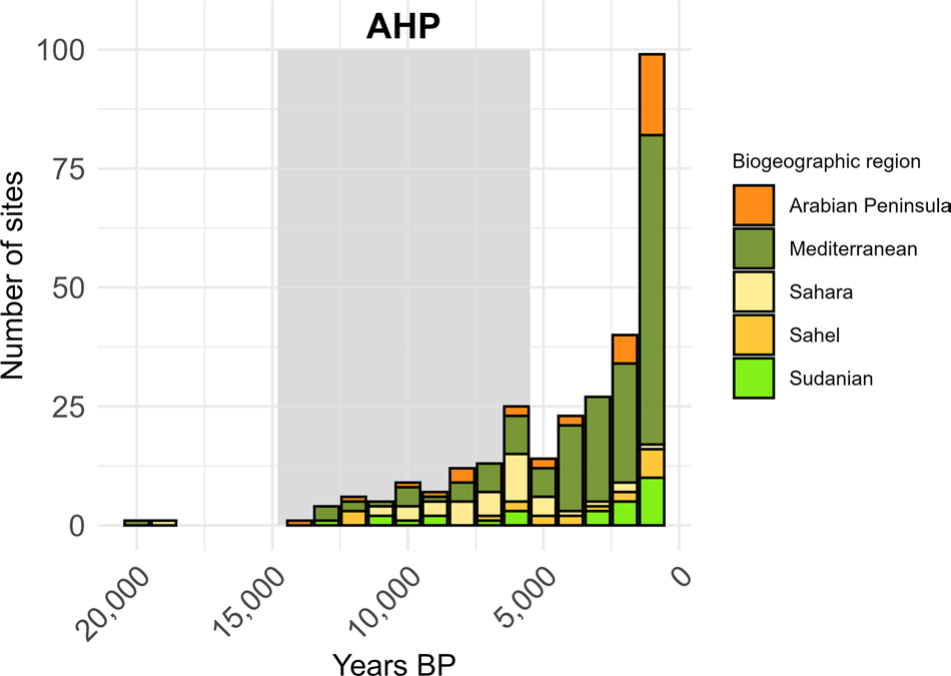
Dated pollen records in 1000-year intervals. The shaded area represents the African Humid Period (AHP: 14,800 to 5,500 years BP). Dates older than 20,000 were omitted, as they were only represented by few records.

As shown in Fig. 8, most dated fossil records belong to the Late Holocene, with some extending into the Pleistocene. Although many records, especially from the Sahara, are from the African Humid Period (AHP: 14,800 to 5,500 BP), they are still less abundant than more recent records, which highlights the need to obtain more Early and Mid-Holocene records to refine our understanding of the AHP.

After harmonisation of the pollen nomenclature, the initial dataset comprising 5,614 pollen types was reduced to 853 harmonised pollen types. After processing the species corresponding to each pollen type, we obtained a final list of 44,970 species (from 202 families and 1,103 genera) assigned to pollen types and present in the study area. These comprise 546 terrestrial pollen types (trees, shrubs, and herbs), 32 aquatic pollen types, and 148 pollen types classified as indeterminate or belonging to other groups (e.g., bryophytes, ferns, algae, fungi) (Table 6).

**Table 6:** Number of pollen taxa per habit category.

Habit (a)	Number or harmonised taxa
Algae	38
Anthocerotophyta	1
Aquatic	32
Bryophyta	4
Climbers	10
Climbing herbs	2
Climbing shrubs	2
Fern	49
Fungi	40
Geophytes	5
Herbs	110
Herbs or Climbers	3
Herbs or Shrubs	79
Herbs or Shrubs or Climbers	7
Herbs or Shrubs or Lianas	3
Herbs or Shrubs or Trees	61
Herbs or Shrubs or Trees or Climbers	11
Herbs or Shrubs or Trees or Lianas	15
Lianas	6
Mangrove	2
Marchantiophyta	2
Palm	8
Parasitic	11
Selaginellales	1
Shrubs	19
Shrubs or Climbers	6
Shrubs or Lianas	4
Shrubs or Trees	178
Shrubs or Trees or Climbers	15
Shrubs or Trees or Epiphytic	2

### Illustrative examples of database use

We present two examples to introduce users to the NAMPHORA database and illustrate its potential applications, ranging from a simple use case (Example A) to a more detailed analysis incorporating trends in plant functional traits (Example B).

#### Example A

**Fig. 9:**
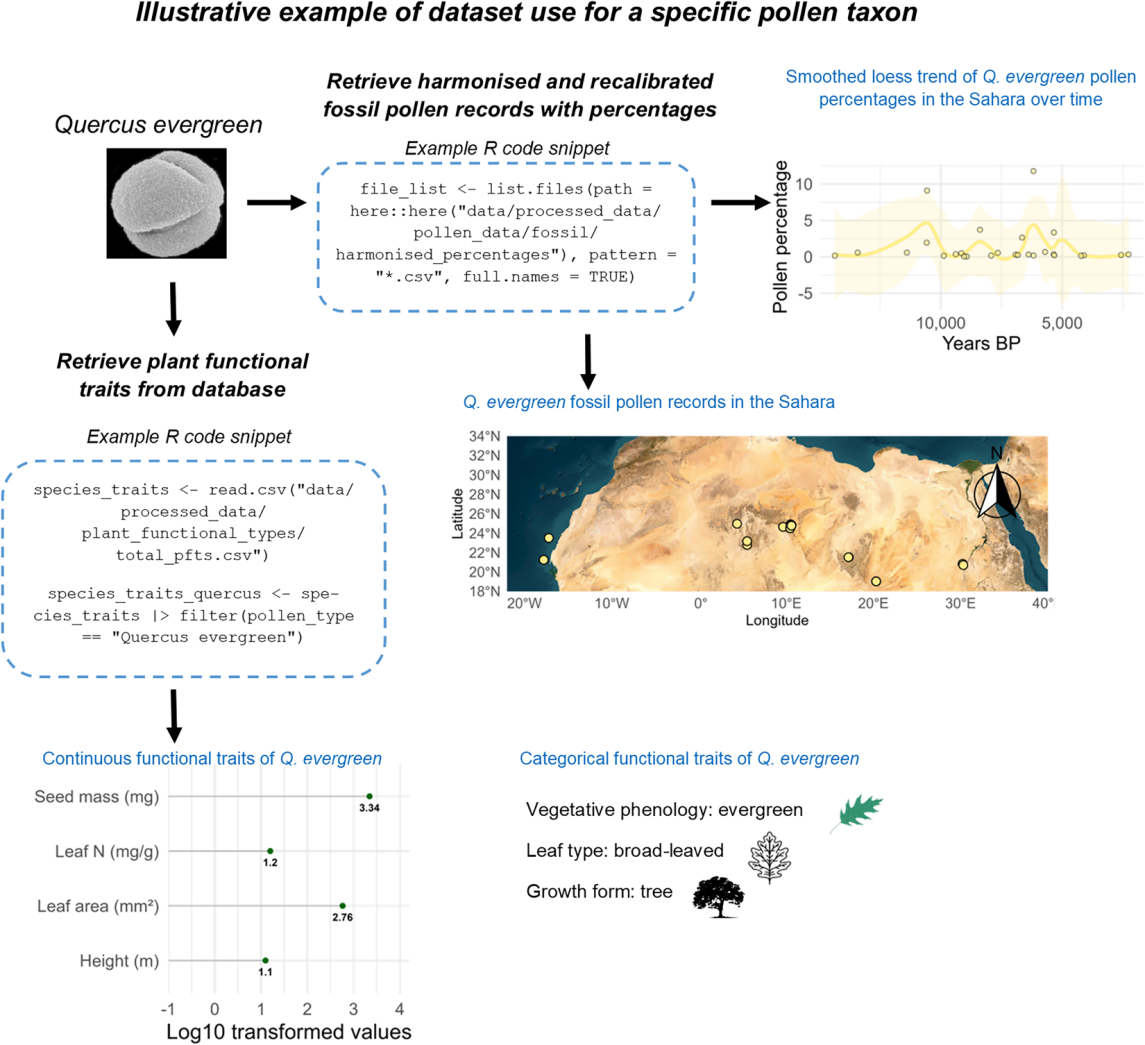
Illustrative example of dataset use for a specific pollen taxon. In this example, a researcher interested in the Holocene distribution of *Quercus evergreen* pollen in the Sahara extracts harmonised and recalibrated fossil pollen records from the NAMPHORA database, selects the sites where *Q. evergreen* is present, and retrieves the corresponding pollen percentages and sample ages. These data are then used to visualise temporal changes in *Q. evergreen* pollen abundance using a LOESS smoothed trend and to map the spatial distribution of fossil occurrences, providing an initial overview of its past abundance and distribution. In addition, the database allows retrieval of plant functional traits associated with the taxon, including both continuous and categorical traits, enabling a basic ecological interpretation of the pollen record.

 A researcher interested in the Holocene distribution of *Quercus evergreen* pollen in the Sahara region may extract all dated fossil pollen records for this taxon and visualise temporal changes in pollen abundance (expressed as pollen percentages), providing an overview of its temporal dynamics in the region (Fig. 9). The researcher may also retrieve information on the plant functional traits associated with this taxon to complement the interpretation of the pollen record.

#### Example B

**Fig. 10:**
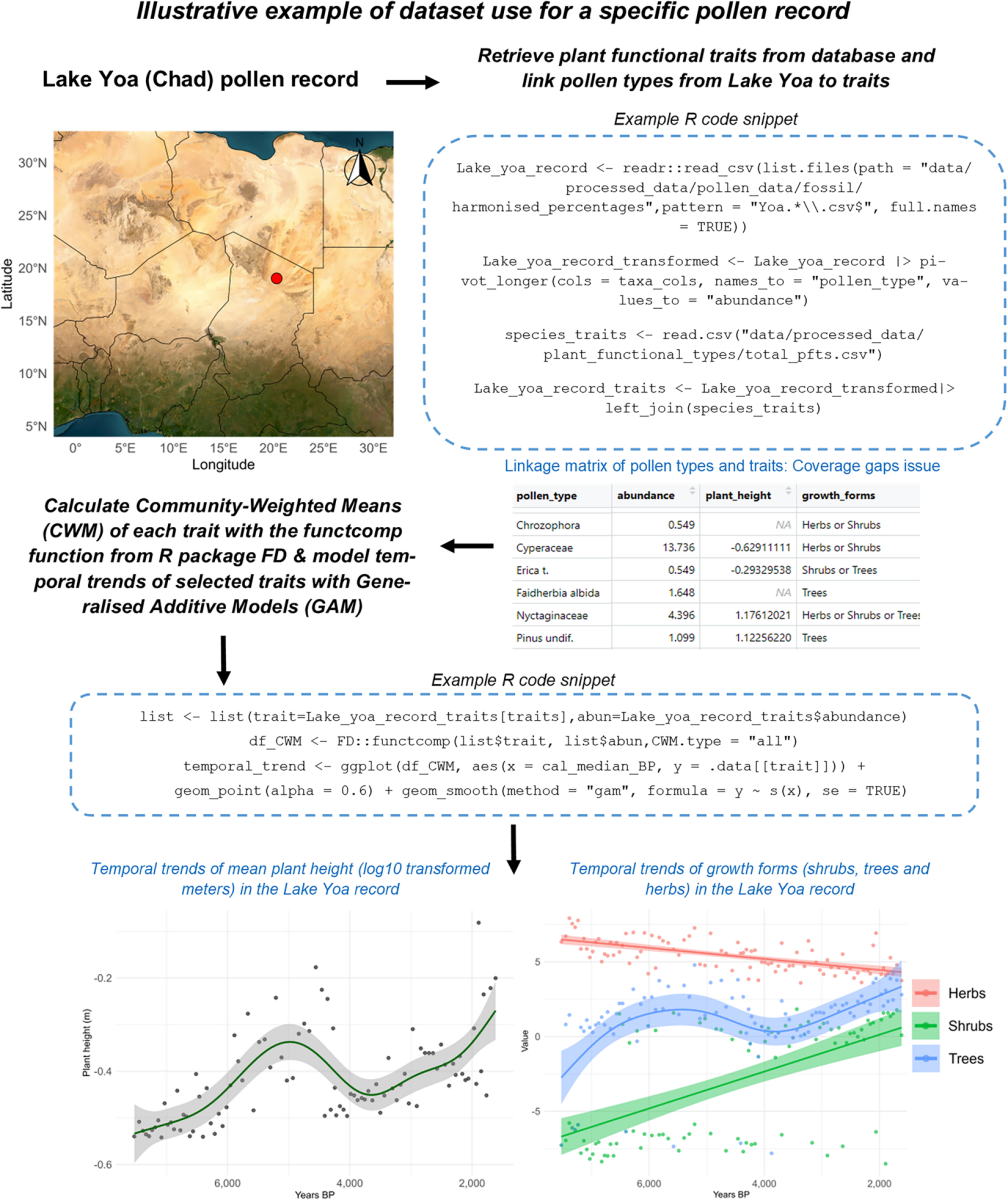
Illustrative example of dataset use for a specific pollen record. In this example, researchers aim to reconstruct temporal trends in plant functional traits in the Lake Yoa pollen record (Chad). They use the harmonised and recalibrated Lake Yoa record from the NAMPHORA database and link the identified pollen types to selected plant functional traits (growth forms and mean plant height). They then calculate the community weighted mean (CWM) of each trait for every pollen sample and apply generalised additive models (GAMs) to estimate temporal trends in these traits, excluding taxa with intermediate growth forms from the analysis. This approach provides a way to estimate changes in ecological strategies through time in a pollen record, complementing traditional vegetation reconstructions.

In a more advanced application, a research group investigates changes in vegetation strategies recorded in the pollen sequence of Lake Yoa (Chad)^52,53^, which is available in NAMPHORA database and has associated trait data. The researchers link the identified pollen taxa to selected plant functional traits (e.g., mean plant height and growth forms) and calculate the community weighted mean (CWM) for each trait^54^ using the *functcomp* function from *FD* R package^55^. This provides the mean trait value of the fossil pollen assemblage weighted by the relative abundance of each pollen taxon. The researchers then model the CWM through time using generalised additive models (GAM) to analyse how dominant ecological strategies changed throughout the Holocene. This approach enables the inference of shifts in vegetation structure and ecological strategies in response to climatic changes associated with the AHP (Fig. 10).

However, the linkage between pollen types and plant functional traits is often incomplete or uncertain (Fig. 10). This uncertainty may arise not only from missing trait information but also because a single pollen taxon can represent species with different functional strategies^56^. For example, a pollen type may include both woody and herbaceous species. In such cases, assigning a unique plant functional trait to the pollen type may oversimplify its ecological interpretation. Researchers can address this issue in several ways: (i) restricting the analysis to taxa with well-defined trait assignments, excluding taxa associated with multiple categories for the same trait (as in this example), (ii) assigning traits based on the most likely ecological affinity using additional ecological or biogeographical information, or (iii) exploring multiple scenarios by testing alternative trait assignments for ambiguous taxa (e.g., one scenario treating the taxon as herbaceous, another as woody). Comparing the trends resulting from these different approaches allows researchers to evaluate the robustness of inferring functional changes through time while accounting for this source of uncertainty.

The workflow underlying these examples is fully documented in the “*11_illustrative_examples_database_use.R”* script, which is available in the Zenodo repository^24^.

## Technical Validation

The pollen records used in this study were sourced from multiple databases, and to ensure consistency, all files were standardised to conform to the structure of the APD. Each record was carefully reviewed, and any discrepancies were cross-checked against the original sources to confirm accuracy. Data processing was carried out using R v. 4.1.1^25^, where the datasets were processed and standardised. Geographic coordinates underwent additional quality control through map projection (Fig. 5).

For pollen taxonomic harmonisation, a comprehensive literature review was conducted, and the harmonised pollen list was cross-referenced with established nomenclature from key studies (Lézine *et al*.^12^, Mottl *et al*.^33^ and the APD dictionary) to ensure robust taxonomic consistency.

Radiocarbon dates were reviewed for reliability, and when necessary, the original sources were consulted to obtain more accurate chronological data.

The phytogeographical affinity and growth habit of each taxon were assigned based on current distribution data from authoritative sources, including GBIF, POWO, and relevant literature (monographs, research articles, theses, and books).

## Usage Notes

The project structure and data pipeline are thoroughly explained in the database website (https://irene131998.github.io/NAMPHORA_database/). Additionally, the R code used for data preparation and processing is fully available and reproducible.

This database has several potential applications, including reconstructing past vegetation and climate and serving as validation for paleoclimatic simulations^2,57^. Contributions and error reports are highly encouraged and can be submitted directly to the corresponding author or through the issues or pull requests sections in the associated GitHub repository (https://github.com/Irene131998/NAMPHORA_database).

Future efforts will focus on expanding the database as new records become available, and the possibility of interconnecting NAMPHORA with the Neotoma database will be explored with data stewards.

## Supplementary information

Supplementary Information

## Data Availability

All raw and processed data are available for download as CSV files from Zenodo repository^24^.

## Code availability

The code used to extract, clean, and process the data, as well as to generate each figure. and map in this manuscript, is written in R programming language. It is openly accessible via Zenodo^24^.
