# Supplementary material for: NAMPHORA: a fossil and modern pollen database from Northern Africa and adjacent Mediterranean and Arabian regions
Source: Sci Data. 2026 May 6;13:1031. doi: 10.1038/s41597-026-07319-8 (PMC13357717; doi:10.1038/s41597-026-07319-8)
Supplement: Supplementary file 1 — Supplementary Information [file 41597_2026_7319_MOESM1_ESM.pdf]

Supplementary Information for **NAMPHORA: a fossil and modern pollen database from Northern Africa and adjacent Mediterranean and Arabian regions**

This supplementary information includes:

Supplementary Information 1. A list with all data contributors. **2-5**

Supplementary Information 2. A complete list of references associated with each dataset **6-83**

- **References pollen records** **6-24**
- **References: taxonomic classification by family and order** **24-27**
- **References: taxonomic classification by subfamily and tribe** **27-32**
- **References: harmonisation of pollen taxonomy** **32-50**
- **References: classification of growth forms** **50-62**
- **References: phytogeographical affinity classification** **63-76**
- **References leaf type (TRY database)** **76-77**
- **References plant functional traits (BIEN database)** **77-83**

Supplementary Information 1. Acknowledgments to Data Contributors: We extend our sincere gratitude to all authors whose data have been included in this database. This database would not exist without their contributions.

Pollen records

Abel-Schaad, D.
Agwu, C. O. C.
Alimen, H.
Allen, J. R. M.
Almogi-Labin, A.
Altolaguirre, Y.
Amaral, P. G. C.
Amrani, S.
Anderson, R. S.
Assémien, P.
Athanasiadis, N
Atherden, M. A.
Bakker, J.
Ballouche, A.
Baruch, U.
Baumhauer, R.
Beffa, G.
Bellini, C.
Ben Tiba, B.
Benslama, M.
Beucher, F.
Bezembinder, L.
Bisculm, M.
Bonnefille, R.
Bordon, A.
Bottema, S.
Bouimetarhan, I.
Brauer, A.
Brisset, E.
Brook, G. A.
Burjachs, F.
Calò, C.
Campbell, J. F. E.
Caratini, C.
Carrión, J. S.
Cerrillo-Cuenca, E.
Châteauneuf, J. J.
Cheddadi, R.
Colombaroli, D.
Connor, S. E.
Cortés-Sánchez, M.,
Coutellier, V.
Creer, K. M.
Cremaschi, M.
Dagnachew Legesse, F.
Darbyshire, I.
Dawelbeit, A.
Delibrias, G.

Plant functional traits from BIEN

Aakala, T.
Abakumova, M.
Ackerly, D. D.
Albrectsen, B. R.
Ameztegui, A.
Angela, M.
Aranda, I.
Austin, A. T.
Bai, Y. F.
Balzotti, C. S.
Bezeng, B. S.
Bhaskar, R.
Bufford, J. L.
Burns, J. H.
Carmona, C. P.
Carus, J.
Cavender-Bares, J.
Chacón, E.
Dalponte, M.
de la Riva, E. G.
Deraison, H.
DeWalt, S. J.
Dostál, P.
Easdale, T. A.
Eastwood, R. J.
Edwards, E. J.
Ellison, A.
Engemann, K.
Enquist, B.
Fang, J.
Feng, Y.
Flynn, S.
Forsyth, D.M.
Fricke, E. C.
Fu, B.
Gapare, W. J.
Gavinet, J.
Goodman, R. C.
Grime, J. P.
Grootemaat, S.
Guehl, J. M.
He, J. S.
Herrera, C. M.
Hodgson, J. G.
Hunt, R.
Ishizuka, W.
Karagatzides, J. D.
Killeen, T.

Plant functional trait from TRY

Cornelissen, J.
Fan Reinfelder, Y.
Han, W.
Harrison, S.
He, T.
Iversen, C.
Kattge, J.
Onoda, Y.
Onstein, R.
Poorter, H.
Reich, P.
van Bodegom, P.
White, M.
Wright, I.

Pollen records	*Plant functional traits from BIEN*	*Plant functional trait from TRY*
Delneuf, M.	Kleinschroth, F.	
Denèfle, M.	Kleyer, M.	
Derks, B.	Kraft, N.	
Dinies, M.	Kraft, T.S.	
Djamali, M.	Kunzmann, D. K.	
Dorado-Valiño, M.	Kurokawa, H.	
Dörfler, W.	Laliberte, E.	
Dupont, L. M.	Leak, J. R.	
Eastwood, W. J.	Leishman, M. R.	
Eggenberger, S.	Letcher, S. G.	
El Ghazali, G. E. B.	Liu, K.	
El Hamouti, N.	Liu, Y.	
El-Moslimany, A. P.	Loughnan, D.	
Engel, M.	Maire, V.	
England, A.	Martin, A. R.	
Fellag, H.	Marx, H. E.	
Feller, C.	Mason, C. M.	
Fersi, W.	Meers, T. L.	
Fletcher, W. J..	Milla, R.	
Florenzano, A.	Molinari, M. A.	
Fofana, C. A. K.,	Moreno-Gutierrez, C.	
Fouache, E.	Mottet, M.	
Foucault, A.	Murali, K. S.	
Garcea, E. A.	Newbery, D. M.	
Garcia Anton, M.	Nidzgorski, D. A.	
Gerasimidis, A.	Niu, K.	
Giralt, S.	Norghauer, J. M.	
Giraudi, C.	Onstein, R. E.	
González-Cordero, A.	Osborne, C. P.	
González-Ramón, A.	Osuri, A. M.	
Grandi, L.	Paine, C. E. T.	
Gremmen, W. H. E.	Pelabon, C.	
Grouard, S.	Pérez-de-Lis, G.	
Grüger, E.	Pico, X.	
Guinet, P.	Ploton, P.	
Hajar, L.	Poorter, L.	
Hamdan, M. A.	Price, C. A.	
Hamilton, A. C.	Prunier, R.	
Haynes, C. V.	Reams, G.	
Hoelzmann, P.	Rodriguez, I.	
Holail, H.	Roe, A. D.	
Hooghiemstra, H.	Royer, D.	
Hoorn, C.	Rusch, G.	
Horisk, N.	Russo, S. E.	
Huntley, B.	Sánchez-Robles, J. M.	
Huysecom, E.	Schmid, B.	
Ivory, S.	Snell-Rood, E. C.	
Jahns, S.	Spasojevic, M. J.	
Jäkel, D.	Steane, D. A.	
Janssen, C. R.	Steendam, R. M.	

Pollen records	***Plant functional traits from BIEN***	***Plant functional trait from TRY***
Joannin, S.	Stuppy, W. H.	
Jouffroy-Bapicot, I.	Szefer, P.	
Kadosh, D.	Thompson, K.	
Kaniewski, D.	Turner, R. M.	
Katrantsiotis, C.	Umaña, M. N.	
Kouli, K.	Urrutia-Jalabert, R. B.	
Kröpelin, S.	van der Plas, F.	
Kuzucuoglu, C.	Violle, C.	
Lamb, H. F.	Welsh, M. E.	
Langgut, D.	Weremijewicz, J.	
Lawson, I. T.	Wigley, B. J.	
Lemonnier, K.	Wilmann, B.	
Lentini, M.	Zanne, A. E.	
Leroy, S.	Zas, R.	
Lézine, A. M.		
Linstädter, J.		
Litt, T.		
López-Merino, L.		
López-Sáez, J. A.		
Luelmo-Lautenschlaeger, R.		
Maley, J.		
Marks, L.		
Marriner, N.		
Médus, J.		
Mercuri, A. M.		
Michel, P.		
Miebach, A.		
Miller, C. S.		
Moe, D.		
Mohammed, M. U.		
Morales, J.		
Morales-Molino, C.		
Morel, A.		
Morhange, C.		
Muller, S.		
Neumann, K.		
Nimmergut, A. P.		
Noti, R.		
Nourelbait, M.		
Palli, J.		
Pantaléon-Cano, J.		
Paquereau, V.		
Parker, A. G.		
Pedrotta, T.		
Petit-Maire, N.		
Pons, A.		
Quézel, P.		
Ravazzi, C.		
Ritchie, J. C.		
Robles López, S.		

Pollen records	***Plant functional traits from BIEN***	***Plant functional trait from TRY***
Roeser, P. A.		
Rossignol-Strick, M.		
Saad, S. I.		
Salzmann, U.		
Sanchez-Goñi, M. F.		
Schiebel, V.		
Schultze, R.		
Schulz, E.		
Schwab, M. J.		
Schwörer, C.		
Sereno, P.		
Servera-Vives, G.		
Stambouli-Essassi, S.		
Stanley, D. J.		
Stevenson, A. C.		
Thinon, M.		
Tinner, W.		
Tissot, C.		
Tovar, C.		
Turner, J.		
Tzedakis, P. C.		
Urban, B.		
Vallé, F.		
Van Campo, M.		
van der Brink, L. M.		
van der Knaap, W. O.		
van Dongen, F. H.		
van Leeuwen, J.		
van Zeist, W.		
Vignola, C.		
Waller, M. P.		
Watts, W. A.		
Whitney, J. W.		
Willis, K. J.		
Winn, K.		
Wulf, S.		
Ybert, J. P.		
Yll, E. I.		
Zaky, A. S.		
Zapata, L.		
Zielhofer, C.		
Zolitschka, B.		

Supplementary Information 2. List of references.

1. References pollen records

Abel-Schaad, D. & López-Sáez, J. A. (2013). Vegetation changes in relation to fire history and human activities at the Peña Negra mire (Bejar Range, Iberian Central Mountain System, Spain) during the past 4,000 years. Vegetation History and Archaeobotany, 22(3), 199–214. https://doi.org/10.1007/s00334-012-0368-9

Abel-Schaad, D., Alba-Sánchez, F., Pérez-Díaz, S. & López-Sáez, J. A. (2017). Contributions to the European Pollen Database. 36. Praillos de Boissier mire, Tejeda Natural Park (Baetic Range, southern Spain). Grana, *56*(6), 475–477. https://doi.org/10.1080/00173134.2016.1276926

Abel-Schaad, D., Hernández-Carretero, A. M., López-Merino, L., Pulido-Díaz, F. J. & López-Sáez, J. A. (2009). Cabras y quemorros: tres siglos de cambios en el paisaje de la vertiente extremeña de la Sierra de Gredos. Revista de Estudios Extremeños, 65(2), 449–478.

Abel-Schaad, D., López-Sáez, J. A. et al. (2009). Evolución de la vegetación en la Sierra de Gata (Cáceres-Salamanca, España) durante el Holoceno reciente. Implicaciones biogeográficas. Revista Española de Micropaleontología, 41(1-2), 91–105.

Agwu, C. O. C. & Beug, H. J. (1982). Palynological studies of marine sediments off the West African coast. "Meteor" Forschungsergebnisse, Reihe C: Geologie und Geophysik, (36), 1–30.

Agwu, C. O. C. (1979). Vegetations- und klimageschichtliche Untersuchung an marinen Sedimenten vor der westafrikanischen Küste. PhD thesis. Universität Göttingen.

Alimen, H., Beucher, F. & Lhote, H. (1968). Les gisements néolithiques de Tan-Tartaït et d'I-n-Itinen, Tassili n-Ajjer (Sahara central). Bulletin de la Société Préhistorique Française, 65(2), 421–458. https://doi.org/10.3406/bspf.1968.4160

Allen, J. R. M. & Huntley, B. (1999). The high resolution paleoenvironmental record of the last 101,800 years from Lago Grande di Monticchio, Italy. PAGES News, 7(3), 10–12. https://doi.org/10.22498/pages.7.3.10

Allen, J. R. M. & Huntley, B. (2009). Last Interglacial palaeovegetation, palaeoenvironments and chronology: a new record from Lago Grande di Monticchio, southern Italy. Quaternary Science Reviews, *28*(15-16), 1521–1538. https://doi.org/10.1016/j.quascirev.2009.02.013

Allen, J. R. M. & Huntley, B. (2018). Effects of tephra falls on vegetation: A Late-Quaternary record from southern Italy. Journal of Ecology, 106(6), 2456–2472. https://doi.org/10.1111/1365-2745.12998

Allen, J. R. M., Brandt, U., Brauer, A., Hubberten, H.-W., Huntley, B., Keller, J., Kraml, M., Mackensen, A., Mingram, J., Negendank, J. F. W., Nowaczyk, N. R., Oberhänsli, H., Watts, W. A., Wulf, S. & Zolitschka, B. (1999). Rapid environmental changes in southern Europe during the last glacial period. Nature, 400(6746), 740–743. https://doi.org/10.1038/23432

Allen, J. R. M., Watts, W. A. & Huntley, B. (2000). Weichselian palynostratigraphy, palaeovegetation and palaeoenvironment; the record from Lago Grande di Monticchio, southern Italy. Quaternary International, 73–74, 91–110. https://doi.org/10.1016/S1040-6182(00)00067-7

Allen, J. R. M., Watts, W. A. & Huntley, B. (2001). The contribution of the palaeovegetation record from Lago Grande di Monticchio to the ELDP. In A. Brauer, J. F. W. Negendank, & A. Berger (Eds.), High-Resolution Lake Sediment Records in Climate and Environment Variability Studies: 6th Workshop of the European Lake Drilling Programme, EDLP: 11–16 May 2001, Potsdam, Germany (pp. 61–64). Terra Nostra.

Allen, J. R. M., Watts, W. A., McGee, E. & Huntley, B. (2002). Holocene environmental variability—the record from Lago Grande di Monticchio, Italy. Quaternary International, 88(1), 69–80. https://doi.org/10.1016/S1040-6182(01)00074-X

Almogi-Labin, A., Bar-Matthews, M., Shriki, D., Kolosovsky, E., Paterne, M., Schilman, B., Ayalon, A., Aizenshtat, Z. & Matthews, A. (2009). Climatic variability during the last ~90 ka of the southern and northern Levantine Basin as evident from marine records and speleothems. Quaternary Science Reviews, 28(25-26), 2882–2896. https://doi.org/10.1016/j.quascirev.2009.07.017

Altolaguirre, Y., Bruch, A. A. & Gibert, L. (2020). A long Early Pleistocene pollen record from Baza Basin (SE Spain): Major contributions to the palaeoclimate and palaeovegetation of Southern Europe. Quaternary Science Reviews, 231, 106199. https://doi.org/10.1016/j.quascirev.2020.106199

Altolaguirre, Y., Postigo-Mijarra, J. M., Barrón, E., Carrión, J. S., Leroy, S. A., & Bruch, A. A. (2019). An environmental scenario for the earliest hominins in the Iberian Peninsula: early Pleistocene palaeovegetation and palaeoclimate. Review of Palaeobotany and Palynology, 260, 51-64. https://doi.org/10.1016/j.revpalbo.2018.10.008

Altolaguirre, Y., Schulz, M., Gibert, L. & Bruch, A. A. (2021). Mapping Early Pleistocene environments and the availability of plant food as a potential driver of early Homo presence in the Guadix-Baza Basin (Spain). Journal of Human Evolution, 155, 102986. https://doi.org/10.1016/j.jhevol.2021.102986

Amaral, P. G. C., Vincens, A., Guiot, J., Buchet, G., Deschamps, P., Doumnang, J. C., & Sylvestre, F. (2013). Palynological evidence for gradual vegetation and climate changes during the African Humid Period termination at 13 N from a Mega-Lake Chad sedimentary sequence. Climate of the Past, 9(1), 223-241. https://doi.org/10.5194/cp-9-223-2013

Amrani, S. (2018). The Holocene flora and vegetation of Ti-n Hanakaten (Tassili n'Ajjer, Algerian Sahara). In A. M. Mercuri, A. C. D'Andrea, R. Fornaciari, & A. Höhn (Eds.), Plants and people in the African past: Progress in African archaeobotany (pp. 123–145). Springer International Publishing. https://doi.org/10.1007/978-3-319-89839-1_8

Anderson, R. S., Jiménez-Moreno, G., Carrión, J. S. & Pérez-Martínez, C. (2011). Postglacial history of alpine vegetation, fire, and climate from Laguna de Río Seco, Sierra Nevada, southern Spain. Quaternary Science Reviews, 30(13-14), 1615–1629. https://doi.org/10.1016/j.quascirev.2011.03.005

Assémien, P. (1971). Etude comparative de flores actuelles et quaternaires récentes de quelques paysages végétaux d'Afrique de l'Ouest. PhD thesis. University of Abidjan.

Athanasiadis, N. (1975). Zur postglazialen Vegetationsentwicklung von Litochoro Katarinis und Pertouli Trikalon (Griechenland). Flora, 164, 99–132. https://doi.org/10.1016/S0367-2530(17)31791-7

Atherden, M. A. & Hall, J. A. (1999). Human impact on vegetation in the White Mountains of Crete since AD 500. The Holocene, 9(2), 183–193. https://doi.org/10.1191/095968399669624306

Bakker, J. (2012). Late Holocene vegetation dynamics in a mountainous environment in the Territory of Sagalassos Turkey (Late Roman till present). Doctoral dissertation. Katholieke Universiteit Leuven.

Bakker, J., Paulissen, E., Kaniewski, D., Poblome, J., De Laet, V., Verstraeten, G., & Waelkens, M. (2013). Climate, people, fire and vegetation: new insights into vegetation dynamics in the Eastern Mediterranean since the 1st century AD. Climate of the Past, 9(1), 57–87. https://doi.org/10.5194/cp-9-57-2013

Ballouche, A. & Neumann, K. (1995). A new contribution to the Holocene vegetation history of the West African Sahel: pollen from Oursi, Burkina Faso and charcoal from three sites in northeast Nigeria. Vegetation History and Archaeobotany, 4(1), 31–39. https://doi.org/10.1007/BF00198613

Ballouche, A. (1992). Dynamique des paysages végétaux sahélo-soudaniens et pratiques agro-pastorales à l'Holocène: exemples du Burkina Faso. Association des Géographes Français.

Ballouche, A. (1998). Holocene dynamics of Sahelo-Sudanian vegetation landscapes and agro-pastoral practices: examples from Burkina Faso. Bulletin de l'Association de Géographes Français, 75(2), 191–200. https://doi.org/10.3406/bagf.1998.2035

Ballouche, A., Reille, M., Thinon, M., Barakat, H. N., & Fontugne, M. (1995). La végétation holocène des montagnes du Sahara Central: une nouvelle conception. In L. Thomas & E. Roche (Eds.), Palynologie africaine (pp. 9–17). CIFEG.

Baruch, U. & Bottema, S. (1991). Palynological evidence for climatic changes in the Levant ca. 17000–9000 BP. In O. Bar-Yosef & F. R. Valla (Eds.), The Natufian culture in the Levant (pp. 11–20). International Monographs in Prehistory.

Baumhauer, R. & Schulz, E. (1984). The Holocene lake of Seguedine, Kaouar, NE-Niger. In J. A. Coetzee & E. M. van Zinderen Bakker (Eds.), Palaeoecology of Africa and the Surrounding Islands (Vol. 16, pp. 283–290). A.A. Balkema.

Beffa, G., Pedrotta, T., Colombaroli, D., Henne, P. D., van Leeuwen, J. F., Süsstrunk, P., ... & Tinner, W. (2016). Vegetation and fire history of coastal north-eastern Sardinia (Italy) under changing Holocene climates and land use. Vegetation History and Archaeobotany, 25(3), 271-289. https://doi.org/10.1007/s00334-015-0548-5

Bellini, C., Mariotti-Lippi, M., & Montanari, C. (2011). Interpretative scenarios emerging from plant micro- and macroremains in the Iron Age site of Salut, Sultanate of Oman. Journal of Archaeological Science, 38(10), 2775–2789. https://doi.org/10.1016/j.jas.2011.06.021

Ben Tiba, B. & Reille, M. (1982). Recherches pollenanalytiques dans les montagnes de Kroumirie (Tunisie septentrionale): premiers résultats. Ecologia Mediterranea, 8(4), 75–86.

Ben Tiba, B. (1987). Recherches pollenanalytiques à Djebel El Ghorra, Tunisie septentrionale: premières approches. Agronomie et Horticulture, 2, 57–60.

Ben Tiba, B. (1995). Cinq millénaires d'histoire de la végétation à Djebel El Ghorra, Tunisie septentrionale. In L. Thomas & E. Roche (Eds.), Palynologie africaine (pp. 49–55). CIFEG.

Benslama, M., Andrieu-Ponel, V., Guiter, F., Reille, M., de Beaulieu, J.-L., Migliore, J., & Djamali, M. (2010). Nouvelles contributions à l'histoire tardiglaciaire et holocène de la végétation en Algérie: analyses polliniques de deux profils

sédimentaires du complexe humide d'El-Kala. Comptes Rendus Biologies, 333(10), 744–754. https://doi.org/10.1016/j.crvi.2010.08.003

Beucher, F. (1979). Flore: contexte palynologique. In N. Petit-Maire (Ed.), Le Sahara Atlantique à l'Holocène - Peuplement et écologie (pp. 235–237). Mémoires du Centre de Recherches Anthropologiques Préhistoriques et Ethnographiques.

Bezembinder, L. & Niessen, A. (1989). Palynologisch en geomorphologisch onderzoek langs de bovenloop van de Rio Zezere, Serra da Estrela, Portugal. Master's thesis. Vakgroep Paleobotanie en Palynologie, Rijksuniversiteit Utrecht.

Bisculm, M., Colombaroli, D., Vescovi, E., van Leeuwen, J. F. N., Henne, P. D., Rothen, J., Procacci, G., Pasta, S., La Mantia, T., & Tinner, W. (2012). Holocene vegetation and fire dynamics in the supra-mediterranean belt of the Nebrodi Mountains (Sicily, Italy). Journal of Quaternary Science, 27(7), 687–698. https://doi.org/10.1002/jqs.2551

Bonnefille, R. & Hamilton, A. C. (1986). Quaternary and Late Tertiary history of Ethiopian vegetation. Symbolae Botanicae Upsalienses, 26(2), 48–63.

Bonnefille, R. & Lézine, A. M. (1981). Evolution des climats dans les zones intertropicales d'Afrique orientale. R&D Programme in the Field of Environment, Climatology Sub-Programme, Contact Group "Reconstitution of Past Climates".

Bonnefille, R. & Lézine, A. M. (1984). Environnement végétal du Rift éthiopien au Quaternaire récent d'après l'étude palynologique du sondage du lac Abiyata. Revue de Paléobiologie, Volume Spécial, 129–135.

Bonnefille, R. & Riollet, G. (1988). Palynologie des sédiments Holocènes de sites archéologiques du Quatar. In M.-L. Inizan (Ed.), Préhistoire à Quatar (Vol. 2, pp. 137–145). Recherche sur les Civilisations.

Bonnefille, R. (1972). Associations polliniques actuelles et quaternaires en Ethiopie (Vallées de l'Awash et de l'Omo). Thesis. University of Paris VI.

Bonnefille, R. (1988). Palynologie des sédiments Holocènes de sites archéologiques du Quatar. In M.-L. Inizan (Ed.), Préhistoire à Quatar (Vol. 2, pp. 137–145). Recherche sur les Civilisations.

Bonnefille, R., & Mohammed, U. (1994). Pollen-inferred climatic fluctuations in Ethiopia during the last 3000 years. Palaeogeography, Palaeoclimatology, Palaeoecology, 109(2-4), 331–343. https://doi.org/10.1016/0031-0182(94)90183-X

Bonnefille, R., Buchet, G. & Vogel, J. C. (1987). Contribution palynologique à l'histoire récente de la forêt de Wenchi (Ethiopie). Mémoires et Travaux de l'École Pratique des Hautes Études, Institut de Montpellier, 17, 143–158.

Bonnefille, R., Robert, C., Delibrias, G., Elenga, H., Herbin, J. P., Lézine, A. M., Perinet, G., & Tiercelin, J. J. (1986). Palaeoenvironment of Lake Abijata, Ethiopia, during the past 2000 years. In L. E. Frostick, R. W. Renaut, I. Reid, & J. J. Tiercelin (Eds.), Sedimentation in the African Rifts (pp. 253–265). Geological Society Special Publication No. 25. https://doi.org/10.1144/gsl.sp.1986.025.01.21

Bonnefille, R., Rossignol-Strick, M., & Riollet, G. (1982). Organic matter and palynology of DSDP site 367 Pliocene-Pleistocene cores off West Africa. Oceanologica Acta, 5(2), 217–224.

Bordon, A., Peyron, O., Lézine, A. M., Brewer, S., & Fouache, E. (2009). Pollen-inferred Late-Glacial and Holocene climate in southern Balkans (Lake Maliq). Quaternary International, 200(1-2), 19–30. https://doi.org/10.1016/j.quaint.2008.05.014

Bottema, S. (1974). Late Quaternary vegetation history of northwestern Greece [Doctoral dissertation, University of Groningen].

Bottema, S. (1979). Pollen analytical investigations in Thessaly (Greece). Palaeohistoria, 21, 19–40.

Bottema, S. (1980). Palynological investigations on Crete. Review of Palaeobotany and Palynology, 31(1-2), 193–217. https://doi.org/10.1016/0034-6667(80)90023-4

Bottema, S. (1982). Palynological investigations in Greece with special reference to pollen as an indicator of human activity. Palaeohistoria, 24, 257–289.

Bottema, S. (1987). Chronology and climatic phases in the Near East from 16,000 to 10,000 B.P. In O. Aurenche, J. Evin, & F. Hours (Eds.), *Chronologies in the Near East: Relative Chronologies and Absolute Chronology, 16,000-4,000 B.P.* (pp. 295–310). British Archaeological Reports International Series 379(ii).

Bottema, S. (1988). A reconstruction of the Halos environment on the basis of palynological information. In H. R. Reinders (Ed.), New Halos: A Hellenistic Town in Thessalía, Greece (pp. 216–226). H&S Publishers.

Bottema, S. (1990). Holocene environment of the Southern Argolid: a pollen core from Kiladha Bay. In T. J. Wilkinson & S. T. Duhon (Eds.), Franchthi Paralia: The Sediments, Stratigraphy, and Offshore Investigations (Vol. 6, pp. 117–138). Indiana University Press.

Bottema, S., & van Zeist, W. (1981). Palynological evidence for the climatic history of the Near East, 50,000-6,000 B.P. In J. Cauvin & P. Sanlaville (Eds.), Préhistoire du Levant (pp. 111–132). Éditions du CNRS.

Bottema, S., & Woldring, H. (1984). Late Quaternary vegetation and climate of southwestern Turkey. Part II. Palaeohistoria, 26, 123–149.

Bottema, S., & Woldring, H. (1990). Anthropogenic indicators in the pollen record of the Eastern Mediterranean. In S. Bottema, G. Entjes-Nieborg, & W. van Zeist (Eds.), Man's Role in the Shaping of the Eastern Mediterranean Landscape (pp. 231–264). A.A. Balkema.

Bottema, S., Woldring, H., & Aytuğ, B. (1993). Late Quaternary vegetation history of northern Turkey. Palaeohistoria, *35/36*, 13–72.

Bouimetarhan, I., Dupont, L., Schefuß, E., Mollenhauer, G., Mulitza, S., & Zonneveld, K. (2009). Palynological evidence for climatic and oceanic variability off NW Africa during the late Holocene. Quaternary Research, 72(2), 188–197. https://doi.org/10.1016/j.yqres.2009.05.004

Bouimetarhan, I., Groeneveld, J., Dupont, L. M., & Zonneveld, K. A. F. (2013). Low- to high-productivity pattern within Heinrich stadial 1: Inferences from dinoflagellate cyst records off Senegal. Global and Planetary Change, 111, 188–199. https://doi.org/10.1016/j.gloplacha.2013.09.009

Bouimetarhan, I., Marret, F., Dupont, L., & Zonneveld, K. (2009). Dinoflagellate cyst distribution in marine surface sediments off West Africa (17–6 N) in relation to sea-surface conditions, freshwater input and seasonal coastal upwelling. Marine Micropaleontology, 71(3-4), 113-130. https://doi.org/10.1016/j.marmicro.2009.02.001

Brauer, A., Allen, J. R. M., Mingram, J., Dulski, P., Wulf, S., & Huntley, B. (2007). Evidence for last interglacial chronology and environmental change from Southern Europe. Proceedings of the National Academy of Sciences, 104(2), 450–455. https://doi.org/10.1073/pnas.0603321104

Brisset, E., Revelles, J., Expósito, I., Bernabeu Aubán, J., & Burjachs, F. (2020). Socio-ecological contingencies with climate changes over the prehistory in the Mediterranean Iberia. Quaternary, *3*(3), 19. https://doi.org/10.3390/quat3030019

Brook, G. A., Burney, D. A., & Cowart, J. B. (1990). Desert paleoenvironmental data from cave speleothems with examples from the Chihuahuan, Somalia-Chalbi, and Kalahari deserts. Palaeogeography, Palaeoclimatology, Palaeoecology, 76(3-4), 311–329. https://doi.org/10.1016/0031-0182(90)90120-5

Burjachs, F. (1996). La secuencia palinológica de La Cruz (Cuenca, España). In M. B. Ruiz-Zapata (Ed.), Estudios palinológicos (pp. 31–36). Servicio de Publicaciones de la Universidad de Alcalá.

Burjachs, F. (2006). Palinología y restitución paleoecológica. Ecosistemas, 15(3), 7–16.

Burjachs, F., & Expósito, I. (2009). Descobrint els paisatges del passat. In M. Marí (Ed.), Vila i Ses Feixes, Els camins de l'aigua (pp. 19–40). GEN-GOB Eivissa.

Burjachs, F., & Expósito, I. (2015). Charcoal and pollen analysis: Examples of Holocene fire dynamics in the Iberian Peninsula. Catena, 135, 340–349. https://doi.org/10.1016/j.catena.2015.08.006

Burjachs, F., Giralt, S., Roca, J. R., Seret, G., & Julià, R. (1997). Palinología holocénica y desertización en el Mediterráneo Occidental. In J. J. Ibáñez, B. L. Valero, & C. Machado (Eds.), El paisaje mediterráneo a través del espacio y del tiempo. Implicaciones en la desertificación (pp. 379–394). Geoforma Ediciones.

Burjachs, F., Jones, S. E., Giralt, S., & Fernández-López de Pablo, J. (2016). Lateglacial to Early Holocene recursive aridity events in the SE Mediterranean Iberian Peninsula: The Salines playa lake case study. Quaternary International, 403, 187–200. https://doi.org/10.1016/j.quaint.2015.10.117

Burjachs, F., Pérez-Obiol, R., Picornell-Gelabert, L., Revelles, J., Servera-Vives, G., Expósito, I., & Yll, E. I. (2017). Overview of environmental changes and human colonization in the Balearic Islands (Western Mediterranean) and their impacts on vegetation composition during the Holocene. Journal of Archaeological Science: Reports, 12, 845–859. https://doi.org/10.1016/j.jasrep.2016.09.018

Burjachs, F., Pérez-Obiol, R., Roure, J. M., & Julià, R. (1994). Dinámica de la vegetación durante el Holoceno en la isla de Mallorca. Trabajos de Palinología básica y aplicada, 199-210.

Calò, C., Henne, P. D., Curry, B. B., Magny, M., Vescovi, E., La Mantia, T., Pasta, S., Vannière, B., & Tinner, W. (2012). Spatio-temporal patterns of Holocene environmental change in southern Sicily. Palaeogeography, Palaeoclimatology, Palaeoecology, 323–325, 110–122. https://doi.org/10.1016/j.palaeo.2012.01.038

Calò, C., Henne, P. D., Eugster, P., van Leeuwen, J. F. N., Gilli, A., Hamann, Y., La Mantia, T., Pasta, S., Vescovi, E., & Tinner, W. (2013). 1200 years of decadal-scale variability of Mediterranean vegetation and climate at Pantelleria Island, Italy. The Holocene, 23(11), 1477–1486. https://doi.org/10.1177/0959683613499056

Campbell, J. F. E., Fletcher, W. J., Joannin, S., Hughes, P. D. M., Rhanem, M., & Zielhofer, C. (2017). Environmental drivers of Holocene Forest development in the Middle Atlas, Morocco. Frontiers in Ecology and Evolution, 5, 113. https://doi.org/10.3389/fevo.2017.00113

Caratini, C., Tissot, C., & Frédoux, A. (1988). Caractérisation des aérosols désertiquesa Niamey (Niger) par leur contenu pollinique. Institut Français de Pondichéry, Travaux de la Section Scientifique et Technique, 25, 251-268.

Carrión, J. S. (2002). Patterns and processes of Late Quaternary environmental change in a montane region of southwestern Europe. Quaternary Science Reviews, 21(18-19), 2047–2066. https://doi.org/10.1016/S0277-3791(02)00011-1

Carrión, J. S., & Dupré, M. (1996). Late Quaternary vegetational history of Navarrés, eastern Spain. A two core approach. New Phytologist, 134(1), 177–191. https://doi.org/10.1111/j.1469-8137.1996.tb01155.x

Carrión, J. S., & van Geel, B. (1999). Fine-resolution Upper Weichselian and Holocene palynological record from Navarrés (Valencia, Spain) and a discussion about factors of Mediterranean forest succession. Review of Palaeobotany and Palynology, 106(3-4), 209–236. https://doi.org/10.1016/S0034-6667(99)00009-8

Carrión, J. S., Andrade, A., Bennett, K. D., Navarro, C., & Munuera, M. (2001). Crossing forest thresholds: inertia and collapse in a Holocene sequence from south-central Spain. The Holocene, 11(6), 635–653. https://doi.org/10.1191/09596830195690

Carrión, J. S., Fuentes, N., González-Sampériz, P., Sánchez Quirante, L., Finlayson, J. C., Fernández, S., & Andrade, A. (2007). Holocene environmental change in a montane region of southern Europe with a long history of human settlement. Quaternary Science Reviews, 26(11-12), 1455–1475. https://doi.org/10.1016/j.quascirev.2007.03.013

Carrión, J. S., Munuera, M., Dupré, M., & Andrade, A. (2001). Abrupt vegetation changes in the Segura Mountains of southern Spain throughout the Holocene. Journal of Ecology, 89(5), 783–797. https://doi.org/10.1046/j.0022-0477.2001.00597.x

Carrión, J. S., Sánchez-Gómez, P., Mota, J. F., Yll, R., & Chaín, C. (2003). Holocene vegetation dynamics, fire and grazing in the Sierra de Gádor, southern Spain. The Holocene, 13(6), 839–849. https://doi.org/10.1191/0959683603hl662rp

Carrión, J. S., Yll, E. I., Willis, K. J., & Sánchez, P. (2004). Holocene forest history of the eastern plateaux in the Segura Mountains (Murcia, southeastern Spain). Review of Palaeobotany and Palynology, 132(3-4), 219–236. https://doi.org/10.1016/j.revpalbo.2004.07.001

Cerrillo-Cuenca, E., & González-Cordero, A. (2011). Burial prehistoric caves in the interior basin of the river Tagus: the complex at Canaleja gorge (Romangordo, Caceres, Spain). BAR International Series, *2219*, 21–42.

Cerrillo-Cuenca, E., González-Cordero, A., & López-Sáez, J. A. (2007). El proyecto de investigación de Garganta Canaleja: aproximación al análisis del Epipaleolítico y el Neolítico en el valle interior del Tajo. In Los Primeros Campesinos de la Raya. Actas de las jornadas de arqueología del Museo de Cáceres (pp. 13–27).

Châteauneuf, J. J., Faure, H., & Lézine, A. M. (1986). Facteurs contrôlant la genèse et la destruction des tourbes tropicales du littoral ouest africain. Documents du Bureau de Recherches Géologiques et Minières, 110, 77–91.

Cheddadi, R., & Khater, C. (2016). Climate change since the last glacial period in Lebanon and the persistence of Mediterranean species. Quaternary Science Reviews, 150, 146–157. https://doi.org/10.1016/j.quascirev.2016.08.010

Cheddadi, R., Bouaissa, O., Rhoujjati, A., & Dezileau, L. (2016). Environmental changes in the Moroccan western Rif mountains over the last 9,000 years. Quaternaire, 27(1), 15–25. https://doi.org/10.4000/quaternaire.7394

Cheddadi, R., Henrot, A. J., François, L., Boyer, F., Bush, M. B., Carré, M., Coissac, E., De Oliveira, P. E., Ficetola, G. F., Hambuckers, A., Huang, K., Lézine, A. M., Nourelbait, M., Rhoujjati, A., Taberlet, P., Sarmiento, F., Abel-Schaad, D., Alba-Sánchez, F., & Zheng, Z. (2017). Microrefugia, climate change, and conservation of Cedrus atlantica in the Rif Mountains, Morocco. Frontiers in Ecology and Evolution, 5, 114. https://doi.org/10.3389/fevo.2017.00114

Cheddadi, R., Lamb, H. F., Guiot, J., & van der Kaars, S. (1998). Holocene climatic change in Morocco: a quantitative reconstruction from pollen data. Climate dynamics, 14(12), 883-890. https://doi.org/10.1007/s003820050262

Cheddadi, R., Nourelbait, M., Bouaissa, O., Tabel, J., Rhoujjati, A., López-Sáez, J. A., ... & Lamb, H. (2015). A history of human impact on Moroccan mountain landscapes. African Archaeological Review, 32(2), 233-248. https://doi.org/10.1007/s10437-015-9186-7

Colombaroli, D., Tinner, W., Van Leeuwen, J., Noti, R., Vescovi, E., Vanniere, B., ... & Bugmann, H. (2009). Response of broadleaved evergreen Mediterranean forest vegetation to fire disturbance during the Holocene: insights from the peri-Adriatic region. Journal of Biogeography, 36(2), 314-326. https://doi.org/10.1111/j.1365-2699.2008.01987.x

Connor, S. E., van Leeuwen, J. F., Van Der Knaap, W. O., Akindola, R. B., Adeleye, M. A., & Mariani, M. (2021). Pollen and plant diversity relationships in a Mediterranean montane area. Vegetation History and Archaeobotany, 30(5), 583-594. https://doi.org/10.1007/s00334-020-00811-0

Cortés-Sánchez, M., Morales-Muñiz, A., Simón-Vallejo, M. D., Bergadà-Zapata, M. M., Delgado-Huertas, A., López-García, P., ... & Vera-Peláez, J. L. (2008). Palaeoenvironmental and cultural dynamics of the coast of Málaga (Andalusia, Spain) during the Upper Pleistocene and early Holocene. Quaternary Science Reviews, 27(23-24), 2176-2193. https://doi.org/10.1016/j.quascirev.2008.03.010

Cortés-Sánchez, M., Morales-Muñiz, A., Simón-Vallejo, M. D., Lozano-Francisco, M. C., Vera-Peláez, J. L., Finlayson, C., ... & Bicho, N. F. (2011). Earliest known use of marine resources by Neanderthals. PloS one, 6(9), e24026. https://doi.org/10.1371/journal.pone.0024026

Coutellier, V. & Stanley, D.J. Late Quaternary stratigraphy and paleogeography of the eastern Nile Delta, Egypt. Marine Geology 77, 257–275 (1987). https://doi.org/10.1016/0025-3227(87)90116-2

Creer, K. M., & Morris, A. (1996). Proxy-climate and geomagnetic palaeointensity records extending back to ca. 75,000 BP derived from sediments cored from Lago Grande di Monticchio, southern Italy. Quaternary Science Reviews, 15(2-3), 167-188. https://doi.org/10.1016/0277-3791(95)00080-1

Cremaschi, M., Zerboni, A., Mercuri, A. M., Olmi, L., Biagetti, S., & Di Lernia, S. (2014). Takarkori rock shelter (SW Libya): an archive of Holocene climate and environmental changes in the central Sahara. Quaternary Science Reviews, 101, 36-60. https://doi.org/10.1016/j.quascirev.2014.07.004

Dagnachew Legesse, F., Gasse, F., Radakovitch, O., Vallet-Coulomb, C., Bonnefille, R., Verschuren, D., Gibert, E., & Barker, P. (2002). Environmental changes in a tropical lake (Lake Abiyata, Ethiopia) during recent centuries. Palaeogeography, Palaeoclimatology, Palaeoecology, 187(3–4), 259–279. https://doi.org/10.1016/S0031-0182(02)00479-0

Darbyshire, I., Lamb, H., & Umer, M. (2003). Forest clearance and regrowth in northern Ethiopia during the last 3000 years. The Holocene, 13(4), 537-546. https://doi.org/10.1191/0959683603hl644rp

Dawelbeit, A. (2018). Later Quaternary sedimentary and paleoclimatic evolution of Kordofan, Sudan (Doctoral dissertation). University of Grenoble, France.

Dawelbeit, A., Jaillard, E., & Eisawi, A. (2019). Sedimentary and paleobiological records of the latest Pleistocene-Holocene climate evolution in the Kordofan region, Sudan. Journal of African Earth Sciences, 160, 103605. https://doi.org/10.1016/j.jafrearsci.2019.103605

Delibrias, G., Petit-Maire, N., & Schulz, E. (1982). Les dépôts récents de la vallée du Shati. In N. Petit-Maire (Ed.), Le Shati, Lac Pléistocène du Fezzan (pp. 86–88). Marseille, France: CNRS.

Delneuf, M., & Médus, J. (1997). Comparaison de deux environnements anthropisés de la période protohistorique du Nord-Cameroun. Geography.

Denèfle, M., Lezine, A. M., Fouache, E., & Dufaure, J. J. (2000). A 12,000-year pollen record from Lake Maliq, Albania. Quaternary Research, 54, 424–432. https://doi.org/10.1006/qres.2000.2179

Derks, B. (1990). Onderzoek in twee hooggelegen Holocene afzettingen in de Serra da Estrela (Master's thesis). University of Utrecht, Utrecht, The Netherlands.

Dinies, M., Plessen, B., Neef, R., & Kürschner, H. (2015). When the desert was green: Grassland expansion during the early Holocene in northwestern Arabia. Quaternary International, 382. https://doi.org/10.1016/j.quaint.2015.03.007

Dinies, M., Schimmel, L., Hoelzmann, P., Kröpelin, S., Darius, F., & Neef, R. (2021). Holocene high-altitude vegetation dynamics on Emi Koussi, Tibesti Mountains (Chad, Central Sahara). Palaeoecology of Africa, 35, 27–49. https://doi.org/10.1201/9781003162766-4

Djamali, M., Gambin, B., Marriner, N., Andrieu-Ponel, V., Gambin, T., Gandouin, E., Lanfranco, S., Médail, F., Pavon, D., Ponel, P., & Morhange, C. (2013). Vegetation dynamics during the early to mid-Holocene transition in NW Malta: Human impact versus climatic forcing. Vegetation History and Archaeobotany, 22, 367–380. https://doi.org/10.1007/s00334-012-0380-0

Dorado-Valiño, M., López-Sáez, J. A., & García-Gómez, E. (2014). Contribution to the European Pollen Database. 21. Patateros, Toledo Mountains (central Spain). Grana, 53, 171–173. https://doi.org/10.1080/00173134.2014.903293

Dörfler, W. (2015). The late 3rd millennium BC in pollen diagrams along a south-north transect from the Near East to northern Central Europe. In H. Meller, H. W. Arz, R. Jung, & R. Risch (Eds.), 2200 BC – Ein Klimasturz als Ursache für den Zerfall der Alten Welt? (pp. 321–332). Landesmuseum für Vorgeschichte Halle.

Dörfler, W., Herking, C., Neef, R., Pasternak, R., & von den Driesch, A. (2011). Environment and economy in Hittite Anatolia. In H. Genz & D. P. Mielke (Eds.), Insights into Hittite history and archaeology (Vol. 2, pp. 99–124). Peeters.

Dupont, L. (1998). Pollen and dinoflagellate cysts of the upper 50 m of site 958. In J. V. Firth (Ed.), Proceedings of the Ocean Drilling Program, Scientific Results, 159T.

Dupont, L. M. (1989). Palynology of the last 680,000 years of ODP Site 658 (off NW-Africa): Fluctuations in paleowind systems. In M. Leinen & M. Sarnthein (Eds.), Paleoclimatology and paleometeorology: Modern and past patterns of global atmospheric transport (NATO ASI Series C, Vol. 282). Kluwer Academic Publishers. https://doi.org/10.1007/978-94-009-0995-3_32

Dupont, L. M. (1992). Marine palynology of interglacial-glacial transitions. In G. Kukla & E. Went (Eds.), Start of a glacial (NATO ASI Series I, Vol. 3, pp. 137–155). Springer. https://doi.org/10.1007/978-3-642-76954-2_11

Dupont, L. M. (1992). Marine palynology of interglacial-glacial transitions. In G. Kukla & E. Went (Eds.), Start of a glacial (NATO ASI Series I, Vol. 3, pp. 137–155). Springer. https://doi.org/10.1007/978-3-642-76954-2_11

Dupont, L. M., & Beug, H. J. (1991). Marine palynological studies of NW Africa. Palaeoecology of Africa.

Dupont, L. M., & Hooghiemstra, H. (1989). The Saharan-Sahelian boundary during the Brunhes chron. Acta Botanica Neerlandica, 38, 139–148.

Dupont, L. M., & Schefuß, E. (2018). The roles of fire in Holocene ecosystem changes of West Africa. Earth and Planetary Science Letters, 497, 56–68. https://doi.org/10.1016/j.epsl.2017.10.049

Eastwood, W. J., Roberts, C. N., Lamb, H. F., & Tibby, J. C. (1999). Holocene environmental change in southwest Turkey: A palaeoecological record of lake and catchment-related changes. Quaternary Science Reviews, 18, 671–695. https://doi.org/10.1016/S0277-3791(98)00104-8

Eggenberger, S., Gobet, E., van Leeuwen, J. F. N., Schwörer, C., van der Knaap, W. O., van Dobben, H. F., Vogel, H., Tinner, W., & Rambeau, C. M. C. (2018). Millennial multi-proxy reconstruction of oasis dynamics in Jordan, by the Dead Sea. Vegetation History and Archaeobotany, 27, 649–664. https://doi.org/10.1007/s00334-017-0663-6

El Ghazali, G. E. B. (1998). Modern lowland pollen spectra and contemporary vegetation in the eastern Sahel Vegetation Zone, Sudan. Review of Palaeobotany and Palynology. https://doi.org/10.1016/s0034-6667(97)00042-0

El Hamouti, N., Lamb, H. F., Fontes, J. C., & Gasse, F. (1991). Changements hydroclimatiques abrupts dans le Moyen Atlas marocain depuis le dernier maximum glaciaire. Comptes Rendus de l'Académie des Sciences, Paris, 313, 259–265.

El-Moslimany, A. P. (1983). History of climate and vegetation in the Eastern Mediterranean and the Middle East from the Pleniglacial to the mid-Holocene (Doctoral dissertation). University of Washington.

Engel, M., Knipping, M., Brückner, H., Kiderlen, M., & Kraft, J. C. (2009). Reconstructing middle to late Holocene palaeogeographies of the lower Messenian and sea level change. Palaeogeography, Palaeoclimatology, Palaeoecology, 284, 257–270. https://doi.org/10.1016/j.palaeo.2009.10.005

England, A., Eastwood, W. J., Roberts, C. N., Turner, R., & Haldon, J. F. (2008). Historical landscape change in Cappadocia (central Turkey): A palaeoecological investigation of annually laminated sediments from Nar Lake. Holocene, 18, 1229–1245. https://doi.org/10.1177/0959683608096598

Fellag, H. (2000). Observations sur la conservation pollinique dans le remplissage de quelques grottes et abris paléolithiques du Sud-Ouest de la France et d'Algérie. Revue d'Archéométrie, 24, 71–83.

Feller, C., Médus, J., Paycheng, C., & Chavane, B. (1981). Étude pédologique et palynologique d'un site protohistorique de la moyenne vallée du fleuve Sénégal. Palaeoecology of Africa, 13, 235–248.

Fersi, W., Bassinot, F., & Lézine, A.M. (2016). Past productivity variations and organic carbon burial in the Gulf of Aden since the Last Glacial Maximum. Quaternaire, 29, 213–226. https://doi.org/10.4000/quaternaire.7655

Fersi, W., Lézine, A.M., & Bassinot, F. (2016). Hydro-climate changes over southwestern Arabia and the Horn of Africa during the last glacial–interglacial transition: A pollen record from the Gulf of Aden. Review of Palaeobotany and Palynology, 234, 176–185. https://doi.org/10.1016/j.revpalbo.2016.04.002

Fletcher, W. J., Boski, T., & Moura, D. (2007). Palynological evidence for environmental and climate change in the lower Guadiana valley, Portugal, during the last 13,000 years. The Holocene, 17, 481–494. https://doi.org/10.1177/0959683607077027

Fletcher, W. J., Sanchez-Goñi, M. F., Peyron, O., & Dormoy, I. (2010). Abrupt climate changes of the last deglaciation detected in a western Mediterranean forest record. Climate of the Past, 6, 245–264. https://doi.org/10.5194/cp-6-245-2010, 2010.

Fletcher, W. J., Zielhofer, C., Mischke, S., Bryant, C. L., Xu, X., & Fink, D. (2017). AMS radiocarbon dating of pollen concentrates in a karstic lake system. Quaternary Geochronology, 39, 112–123. https://doi.org/10.1016/j.quageo.2017.02.006

Florenzano, A., et al. (2016). Palynological evidence of cultural and environmental connections in Sudanese Nubia during the Early and Middle Holocene. Quaternary International, 65–80. https://doi.org/10.1016/j.quaint.2016.01.001

Florenzano, A., et al. (2018). Plants, water and humans: Pollen analysis from Holocene archaeological sites on Sai Island, northern Sudan. Palynology, 43. https://doi.org/10.1080/01916122.2017.1384411

Fofana, C. A. K., Sow, E., & Lézine, A. M. (2020). The Senegal River during the last millennium. Review of Palaeobotany and Palynology, 275. https://doi.org/10.1016/j.revpalbo.2020.104175

Fouache, E., Dufaure, J. J., Denèfle, M., Lezine, A. M., Léra, P., Prendi, F., & Touchais, G. (2001). Man and environment around Lake Maliq (southern Albania) during the Late Holocene. Vegetation History and Archaeobotany, 10, 79–86. https://doi.org/10.1007/PL00006922

Foucault, A., & Stanley, D. J. (1989). Late Quaternary palaeoclimatic oscillations in East Africa recorded by heavy minerals in the Nile Delta. Nature, 339, 44–46. https://doi.org/10.1038/339044a0

Garcea, E. A. (2014). Gobero: The no-return frontier. Archaeology and landscape at the Saharo-Sahelian borderland. Azania: Archaeological Research in Africa, 49(2), 267–269. https://doi.org/10.1080/0067270X.2014.904073

Garcia Anton, M., & Sainz Ollero, H. (n.d.). Étude palynologique menée dans le secteur de Mleiha. In M. Mouton (Ed.), Mleiha 1: Environnements, stratégies de subsistence et artisanats. Travaux de la Maison de l'Orient Méditerranéen, Lyon.

Gerasimidis, A., & Athanasiadis, N. (1995). Woodland history of northern Greece from the mid-Holocene to recent time based on evidence from peat pollen profiles. Vegetation History and Archaeobotany, 4, 109–116. https://doi.org/10.1007/BF00206919

Gerasimidis, A., & Panajiotidis, S. (2010). Flambouro, Pieria Mountains (northern Greece). Grana, 49, 76–78. https://doi.org/10.1080/00173130903407571

Giralt, S., Burjachs Casas, F., Roca, J. R., & Julià Brugués, R. (1999). Late Glacial to Early Holocene environmental adjustment in the Mediterranean semi-arid zone of the Salines playa-lake (Alacante, Spain). Journal of Paleolimnology, 21, 449–460. https://doi.org/10.1023/A:1008000513938

Giraudi, C., & Mercuri, A.-M. (2013). Early to Middle Holocene environmental variations in the Gobero Basin. Journal of African Archaeology, Monograph Series 9, 114–126.

Giraudi, C., Mercuri, A. M., & Esu, D. (2012). Holocene palaeoclimate in the northern Sahara margin (Jefara Plain, northwestern Libya). Holocene, 23, 339–352. https://doi.org/10.1177/0959683612460787

González-Cordero, A., Cerrillo-Cuenca, E., López-Sáez, J.A. & López-Merino, L. El yacimiento de Sierra de La Pepa (La Cumbe, Cáceres). Apuntes sobre el proceso transicional del Neolítico Final al Calcolítico en Extremadura. In: Hernández-Pérez, M.S., Soler-Díaz, J.A. & López-Padilla, J.A. (Eds.), Actas IV Congreso del Neolítico Peninsular. Tomo II 340-347 (Museo Arqueológico de Alicante, Diputacíon de Alicante, Alicante, 2008).

González-Ramón, A., Andreo, B., Ruiz-Bustos, A., Richards, D. A., López-Sáez, J. A., & Alba-Sánchez, F. (2012). Late Quaternary paleoenvironmental record from a sedimentary fill in Cucú cave, Almería, SE Spain. Quaternary Research, 77, 264–272. https://doi.org/10.1016/j.yqres.2011.12.001

Grandi, L., Lippi, D., & Mercuri, A. M. (2008). Pollen in dung layers from rockshelters and caves of Wadi Teshuinat (Libyan Sahara). In M. Cremaschi & S. di Lernia (Eds.), Wadi Teshuinat: Palaeoenvironment and prehistory in south-western Fezzan (Libyan Sahara) (pp. 95–106).

Gremmen, W. H. E., & Bottema, S. (1991). Palynological investigations in the Syrian Gazira. In H. Kühne (Ed.), Die rezente Umwelt von Tall Seh Hamad und Daten zur Umweltrekonstruktion der assyrischen Stadt Dur-Katlimmu (pp. 105–116). Berlin, Germany: Dietrich Reimer Verlag.

Grouard, S., & Lezine, A.M. (2023). At the edge of the desert: The evolution of the Holocene environment in Lake Rkiz, Senegal River valley. Quaternary International, 667, 41–50. https://doi.org/10.1016/j.quaint.2023.05.023

Grüger, E. (2002). Pollen analysis of soil samples from the A.D. 79 level: Boscoreale, Oplontis, and Pompeii. In W. F. Jashemski & F. G. Meyer (Eds.), The Natural History of Pompeii (pp. 189–216). Cambridge University Press.

Grüger, E., & Thulin, B. (1998). First results of biostratigraphical investigations of Lago d'Averno near Naples relating to the period 800 BC–800 AD. Quaternary International, 47–48, 35–40. https://doi.org/10.1016/S1040-6182(97)00068-2

Guinet, P., & Planque, D. (1969). Résultats de l'analyse pollinique. In G. Camps (Ed.), AMEKNI, néolithique ancien du Hoggar (Mémoires du Centre de Recherche Anthropologiques, Préhistoriques et Ethnographiques). Organisme de Coopération Scientifique en Algérie, Paris.

Hajar, L., Haïdar-Boustani, M., Khater, C., & Cheddadi, R. (2010). Environmental changes in Lebanon during the Holocene: Man vs. climate impacts. Journal of Arid Environments, 74, 746–755. https://doi.org/10.1016/j.jaridenv.2008.11.002

Hamdan, M. A., Flower, R. J., Hassan, F. A., & Leroy, S. A. G. (2020). Geochemical and palynological analysis of Faiyum Lake sediments, Egypt: Implications for Holocene paleoclimate. Journal of African Earth Sciences, 167, 1–18. https://doi.org/10.1016/j.jafrearsci.2020.103864

Hamdan, M. A., Hassan, F. A., Flower, R. J., Leroy, S. A. G., Shallaly, N. A., & Flynn, A. (2019). Source of Nile sediments in the floodplain at Saqqara inferred from mineralogical, geochemical, and pollen data, and their palaeoclimatic and geoarchaeological significance. Quaternary International, 501(B), 272–288. https://doi.org/10.1016/j.quaint.2018.02.021

Hamilton, A. C. (1982). Upper Quaternary pollen diagrams from montane eastern Africa. In A. C. Hamilton (Ed.), Environmental history of East Africa: A study of the Quaternary (pp. 111–191). Academic Press.

Haynes, C. V., Eyles, C. H., Pavlish, L. A., Ritchie, J. C., & Rybak, M. (1989). Holocene palaeoecology of the eastern Sahara, Selima Oasis. Quaternary Science Reviews, 8, 109–136. https://doi.org/10.1016/0277-3791(89)90001-2

Hoelzmann, P., Jolly, D., Harrison, S. P., Laarif, F., Bonnefille, R., & Pachur, H. J. (1998). Mid-Holocene land-surface conditions in northern Africa and the Arabian Peninsula: A data set for the analysis of biogeophysical feedbacks in the climate system. Global Biogeochemical Cycles, 12(1), 35–51. https://doi.org/10.1029/97GB02733

Holail, H., El Beialy, S., & El-Ghazaly, G. (1996). Sedimentological and palynological characteristics of mangrove sediments, North Qatar. Neue Jahrbücher für Geologie und Paläontologie, Abhandlungen, 200, 309–323. https://doi.org/10.1127/njgpa/200/1996/309

Hooghiemstra, H. (1988). Changes of major wind belts and vegetation zones in NW Africa 20,000–5,000 yr B.P., as deduced from a marine pollen record near Cap Blanc. Review of Palaeobotany and Palynology, 53, 83–99. https://doi.org/10.1016/0034-6667(88)90056-5

Hooghiemstra, H. (1988). Palynological records from northwest African marine sediments: A general outline of the interpretation of the pollen signal. Philosophical Transactions of the Royal Society of London. Series A, 324, 101–116.

Hooghiemstra, H. (1989). Variations of the NW African trade wind regime during the last 140,000 years: Changes in pollen flux evidenced by marine sediment records. In M. Leinen & M. Sarnthein (Eds.), Paleoclimatology and paleometeorology: Modern and past patterns of global atmospheric transport (pp. 183–194). Kluwer Academic Publisher.

Hooghiemstra, H., & Agwu, C. O. C. (1988). Changes in the vegetation and trade winds in equatorial northwest Africa 140,000–70,000 yr B.P. as deduced from two marine pollen records. Palaeogeography, Palaeoclimatology, Palaeoecology, 65, 123–136. https://doi.org/10.1016/0031-0182(88)90199-X

Hooghiemstra, H., Bechler, A., & Beug, H. J. (1987). Isopollen maps for 18,000 years B.P. of the Atlantic offshore of Northwest Africa: Evidence for paleowind circulation. Paleoceanography, 2, 561–582. https://doi.org/10.1029/PA002i006p00561

Hooghiemstra, H., Stalling, H., Agwu, C. O. C., & Dupont, L. M. (1992). Vegetational and climatic changes at the northern fringe of the Sahara 250,000–5,000 years BP: Evidence from 4 marine pollen records located between Portugal and the Canary Islands. Review of Palaeobotany and Palynology, 73, 1–12. https://doi.org/10.1016/0034-6667(92)90137-6

Hooghiemstra, H., Stalling, H., Agwu, C. O. C., & Dupont, L. M. (1992). Vegetational and climatic changes at the northern fringe of the Sahara 250,000–5,000 years BP: Evidence from 4 marine pollen records located between Portugal and the Canary Islands. Review of Palaeobotany and Palynology, 73, 1–12. https://doi.org/10.1016/0034-6667(92)90137-6

Hoorn, C., & Cremaschi, M. (2004). Late Holocene palaeoenvironmental history of Khawr Rawri and Khawr Al Balid (Dhofar, Sultanate of Oman). Palaeogeography, Palaeoclimatology, Palaeoecology, 213, 1–36. https://doi.org/10.1016/j.palaeo.2004.03.014

Horisk, K., Ivory, S., McCorriston, J., McHale, M., Al-Mehri, A., Anderson, D., & Anderson, W. A. K. F. (2023). Vegetation dynamics in Dhofar, Oman, from the Late Holocene to present inferred from rock hyrax middens. Quaternary Research. https://doi.org/10.1017/qua.2023.42

Huntley, B., Allen, J. R. M., & Watts, W. A. (1996). Weichselian late-glacial palaeoecology and palaeoenvironment at Lago Grande di Monticchio (Basilicata, S Italy). Il Quaternario Ital. Journal of Quaternary Science, 9, 605–616.

Huntley, B., Watts, W. A., Allen, J. R. M., & Zolitschka, B. (1999). Palaeoclimate, chronology and vegetation history of the Weichselian Lateglacial: Comparative analysis of data from three cores at Lago Grande di Monticchio, southern Italy. Quaternary Science Reviews, 18, 945–960. https://doi.org/10.1016/S0277-3791(99)00007-4

Huysecom, E., Boëda, E., Deforce, K., Doutrelepont, H., Downing, A., Fedoroff, N., Konaté, D., Mayor, A., Ozainne, S., Raeli, F., Robert, A., Roche, E., Soriano, S., Sow, O., & Stokes, S. (2000). Unpublished report (55 p.).

Ivory, S., Cole, K., Anderson, R. S., & McCorriston, M. (2021). Human landscape modification and expansion of tropical woodland in southern Arabia during the mid-Holocene from rock hyrax middens. Journal of Biogeography, 48, 2588–2603. https://doi.org/10.1111/jbi.14226

Jahns, S. (1993). On the Holocene vegetation history of the Argive Plain (Peloponese, southern Greece). Vegetation History and Archaeobotany, 2, 187–203. https://doi.org/10.1007/BF00198161

Jahns, S. (1995). A Holocene pollen diagram from El Atrun, northern Sudan. Vegetation History and Archaeobotany, 4, 23–30. https://doi.org/10.1007/BF00198612

Jahns, S. (2003). A late Holocene pollen diagram from the Megaris, Greece, giving possible evidence for cultivation of Ceratonia siliqua L. during the last 2,000 years. Vegetation History and Archaeobotany, 12, 127–130. https://doi.org/10.1007/s00334-003-0013-8

Jahns, S. (2005). The Holocene history of vegetation and settlement at the coastal site of Lake Voulkaria in Acarnania, western Greece. Vegetation History and Archaeobotany, 14, 55–66. https://doi.org/10.1007/s00334-004-0053-8

Jahns, S. (2009). The Holocene history of vegetation and environment of Northern Acarnania, Western Greece. In T. Mattern & A. Vött (Eds.), Mensch und Umwelt im Spiegel der Zeit (pp. 5–26). Harrassowitz Verlag.

Jahns, S. (2013). Palynologische Untersuchungen an Sedimenten des Ozeros-Sees. In F. Lang, P. Funke, L. Kolonas, E. L. Schwandner, & D. Maschek (Eds.), Interdisziplinäre Forschungen in Akarnanien (pp. 81–86). Habelt-Verlag.

Jahns, S. Unpublished

Jahns, S., & Herking, C. (2013). Zur Vegetationsentwicklung im mittleren und späten Holozän in Aetolien, westliches Griechenland. Offa, 69/70, 495–503.

Jäkel, D., & Schulz, E. (1972). Spezielle Untersuchungen an der Mittelterrasse im Enneri Tabi, Tibesti-Gebirge. Zeitschrift für Geomorphologie, 15, 129–143.

Janssen, C. R., & Woldringh, R. E. (1981). A preliminary radiocarbon dated pollen sequence from the Serra da Estrela, Portugal. Finisterra XVI, 299–309. https://doi.org/10.18055/Finis2176

Joannin, S., Brugiapaglia, E., de Beaulieu, J. L., Bernardo, L., Magny, M., Peyron, O., Goring, S., & Vannière, B. (2012). Pollen-based reconstruction of Holocene vegetation and climate in southern Italy: The case of Lago Trifoglietti. Climate of the Past, 8, 1973–1996. https://doi.org/10.5194/cp-8-1973-2012, 2012.

Jouffroy-Bapicot, I., Pedrotta, T., Debret, M., Field, S., Sulpizio, R., Zanchetta, G., Sabatier, P., Roberts, C. N., Tinner, W., Walsh, K., & Vannière, B. (2021). Olive groves around the lake. A ten-thousand-year history of a Cretan landscape (Greece) reveals the dominant role of humans in making this Mediterranean ecosystem. Quaternary Science Reviews, 267, 107072. https://doi.org/10.1016/J.QUASCIREV.2021.107072

Jouffroy-Bapicot, I., Vannière, B., Iglesias, V., Debret, M., & Delerras, J. F. (2016). 2,000 years of grazing history and the making of the Cretan mountain landscape, Greece. PLoS ONE, 11, 1–24. https://doi.org/10.1371/journal.pone.0156875

Kadosh, D., Sivan, D., Kutiel, H., & Weinstein-Evron, M. (2004). A late Quaternary palaeoenvironmental sequence from Dor, Carmel coastal plain, Israel. Palynology, 28, 143–157. https://doi.org/10.2113/28.1.143

Kaniewski, D., et al. (2007). A high-resolution Late Holocene landscape ecological history inferred from an intramontane basin in the Western Taurus Mountains, Turkey. Quaternary Science Reviews, 26, 2201–2218. https://doi.org/10.1016/j.quascirev.2007.04.015

Kaniewski, D., et al. (2008). Late Holocene fire impact and post-fire regeneration from the Bereket basin, Taurus Mountains, southern Turkey. Quaternary Research, 70, 228–239. https://doi.org/10.1016/j.yqres.2008.04.002

Kaniewski, D., Marriner, N., Ilan, D., Morhange, C., Thareani, Y., & van Campo, E. (2017). Climate change and water management in the biblical city of Dan. Science Advances, 3, 1–8. https://doi.org/10.1126/sciadv.1700954

Kaniewski, D., Paulissen, E., van Campo, E., Al-Maqdissi, M., Bretschneider, J., & van Lerberghe, K. (2008). Middle East coastal ecosystem response to middle-to-late Holocene abrupt climate changes. Proceedings of the National Academy of Sciences USA, 105, 13941–13946. https://doi.org/10.1073/pnas.0803533105

Kaniewski, D., van Campo, E., & Weiss, H. (2012). Drought is a recurring challenge in the Middle East. Proceedings of the National Academy of Sciences USA, 109, 3962–3967. https://doi.org/10.1073/pnas.1116304109

Kaniewski, D., van Campo, E., Guiot, J., Le Burel, S., Otto, T., & Baeteman, C. (2013). Environmental roots of the Late Bronze Age crisis. PLoS ONE, 8, 1–10. https://doi.org/10.1371/journal.pone.0071004

Kaniewski, D., van Campo, E., Morhange, C., Guiot, J., Zviely, D., Shaked, I., Otto, T., & Artzy, M. (2013). Early urban impact on Mediterranean coastal environments. Scientific Reports, 3, 3540. https://doi.org/10.1038/srep03540

Kaniewski, D., van Campo, E., Paulissen, E., Weiss, H., Bakker, J., Rossignol, I., & van Lerberghe, K. (2011). The medieval climate anomaly and Little Ice Age in coastal Syria inferred from pollen-derived palaeoclimate patterns. Global and Planetary Change, 78, 178–187. https://doi.org/10.1016/j.gloplacha.2011.06.010

Katrantsiotis, C., Norström, E., Smittenberg, R. H., Finne, M., Weiberg, E., Hättestrand, M., Avramidis, P., & Wastegård, S. (2019). Climate changes in the Eastern Mediterranean over the last 5,000 years and their links to the high-altitude atmospheric patterns and Asian monsoons. Global and Planetary Change, 175, 36–51. https://doi.org/10.1016/j.gloplacha.2019.02.001

Kouli, K., & Dermitzakis, M. D. (2010). Lake Orestiás (Kastoria, northern Greece). Grana, 49, 154–156. https://doi.org/10.1080/00173131003780016

Kröpelin, S., et al. (2008). Climate-driven ecosystem succession in the Sahara. Science. https://doi.org/10.1126/science.1154913

Kuzucuoglu, C., Dörfler, W., Kunesch, S., & Goupille, F. (2011). Mid- to late-Holocene climate change in central Turkey: The Tecer Lake record. The Holocene, 21, 173–188. https://doi.org/10.1177/095968361038416

Lamb, H. F., & van der Kaars, S. (1995). Vegetational response to Holocene climatic change: pollen and palaeolimnological data from the Middle Atlas, Morocco. The Holocene, 5, 400–408. https://doi.org/10.1177/095968369500500402

Langgut, D., & Finkelstein, I. (2023). Environment, subsistence strategies and settlement seasonality in the Negev Highlands (Israel) during the Bronze and Iron Ages: The palynological evidence. PLoS ONE, 18, e0285358. https://doi.org/10.1371/journal.pone.0285358

Langgut, D., Almogi-Labin, A., Bar-Matthews, M., & Weinstein-Evron, M. (2011). Vegetation and climate changes in the South Eastern Mediterranean during the last Glacial-Interglacial cycle (86 ka): new marine record. Quaternary Science Reviews, 30, 3960–3972. https://doi.org/10.1016/j.quascirev.2011.10.016

Langgut, D., et al. (2014). Dead Sea pollen record and history of human activity in the Judean Highlands (Israel) from the Intermediate Bronze into the Iron Ages (2500–500 BCE). Palynology, 38, 280–302. https://doi.org/10.1080/01916122.2014.906001

Langgut, D., et al. (2019). The origin and spread of olive cultivation in the Mediterranean Basin: The fossil pollen evidence. The Holocene, 29, 902–922. https://doi.org/10.1177/0959683619826654

Lawson, I. T., Al-Omari, S., Tzedakis, P. C., Bryant, C. L., & Christanis, K. (2005). Lateglacial and Holocene vegetation history at Nisi Fen and the Boras mountain, northern Greece. The Holocene, 15, 873–887. https://doi.org/10.1191/0959683605hl860ra

Lemonnier, K., & Lézine, A.-M. (2021). Timing and nature of the end of the African Humid Period in the Sahel: Insight from pollen data. In Palaeoecol. Afr. https://doi.org/10.1201/9781003162766-5

Lentini, M. (1988). Preliminary pollen analysis at Geili (Sudan): the changing vegetational pattern. In I. Caneva (Ed.), El Geili (pp. 49–55). BAR International Series, 424.

Leroy, S. A. G. (1992). Palynological evidence of Azolla nilotica Dec. in recent Holocene of the eastern Nile Delta and palaeoenvironment. Vegetation History and Archaeobotany, 1, 43–52. https://doi.org/10.1007/BF00190700

Leroy, S. A. G. (2010). Pollen analysis of core DS7-1SC (Dead Sea) showing intertwined effects of climatic change and human activities in the Late Holocene. Journal of Archaeological Science, 37, 306–316. https://doi.org/10.1016/j.jas.2009.09.042

Leroy, S. A. G., & Dupont, L. M. (1997). Marine palynology of the ODP site 658 (N-W Africa) and its contribution to the stratigraphy of Late Pliocene. Geobios, 30(3), 351–359. https://doi.org/10.1016/S0016-6995(97)80194-5

Leroy, S. A. G., Boyraz, S., & Gürbüz, A. (2009). High-resolution palynological analysis in Lake Sapanca as a tool to detect recent earthquakes on the North Anatolian Fault. Quaternary Science Reviews, 28, 2616–2632. https://doi.org/10.1016/j.quascirev.2009.05.018

Leroy, S. A. G., Freitas, M. C., Endrade, C., Cearreta, A., Maanan, M., & Costa, P. J. M. (2025). A 6600 year history of vegetation changes and sediment infill of the Moulay Bousselham Lagoon, Atlantic Morocco. Journal of African Earth Sciences, 223, 105492. https://doi.org/10.1016/j.jafrearsci.2024.105492

Leroy, S. A. G., Kazanci, N., Ileri, Ö., Kibar, M., Emre, Ö., McGee, E. J., & Griffiths, H. I. (2002). Abrupt environmental changes within a late Holocene lacustrine sequence south of the Marmara Sea (Lake Manyas, N-W Turkey): Possible links with seismic events. Marine Geology, 190, 531–552. https://doi.org/10.1016/S0025-3227(02)00361-4

Leroy, S. A. G., Schwab, M. J., & Costa, P. J. M. (2010). Seismic influence on the last 1500-year infill history of Lake Sapanca (North Anatolian Fault, NW Turkey). Tectonophysics, 486, 12–27. https://doi.org/10.1016/j.tecto.2010.02.005

Leroy, S., & Dupont, L. (1994). Development of vegetation and continental aridity in northwestern Africa during the Late Pliocene: The pollen record of ODP site 658. Palaeogeography, Palaeoclimatology, Palaeoecology, 109, 99–111. https://doi.org/10.1016/0031-0182(94)90181-3

Lézine, A. (1988). Les variations de la couverture forestière mésophile d'Afrique occidentale au cours de l'Holocène. Comptes Rendus de l'Académie des Sciences, Paris, 307, 439–445.

Lézine, A. (1988). New pollen data from the Sahel, Senegal. Review of Palaeobotany and Palynology, 55, 141–154. https://doi.org/10.1016/0034-6667(88)90082-6

Lézine, A. (1991). West African paleoclimates during the last climatic cycle inferred from an Atlantic deep-sea pollen record. Quaternary Research. https://doi.org/10.1016/0033-5894(91)90058-D

Lézine, A. M. (1981). Le Lac Abiyata (Ethiopie) – Palynologie et paléoclimatologie du Quaternaire récent. [Doctoral thesis, Université de Bordeaux].

Lézine, A. M. (1987). Paléoenvironnements végétaux d'Afrique Nord-Tropicale depuis 12 000 B.P.: Analyse pollinique de séries sédimentaires continentales (Sénégal-Mauritanie) [Doctoral thesis, Université Aix-Marseille II].

Lézine, A. M., & Bonnefille, R. (1982). Diagramme pollinique Holocène d'un sondage du Lac Abiyata (Ethiopie, 7°42' Nord). Pollen et Spores. https://doi.org/10.1016/0034-6667(86)90012-6

Lézine, A. M., & Casanova, J. (1991). Correlated oceanic and continental records demonstrate past climate and hydrology of North Africa (0–140 ka). Geology, 19, 563–566. https://doi.org/10.1130/0091-7613(1991)019<0307:COACRD>2.3.CO;2

Lézine, A. M., & Chateauneuf, J. (1991). Peat in the Niayes of Senegal: Depositional environment and Holocene evolution. Journal of African Earth Sciences, 12, 171–179. https://doi.org/10.1016/0899-5362(91)90067-9

Lézine, A. M., Casanova, J., & Hillaire-Marcel, C. (1990). Across an early Holocene humid phase in western Sahara: Pollen and isotope stratigraphy. Geology, 18, 264–267. https://doi.org/10.1130/0091-7613(1990)018<0264:aaehhp>2.3.co;2

Lézine, A. M., et al. (1985). Étude palynologique et sédimentologique d'un milieu margino-littoral: La tourbière de Thiaye (Sénégal). Sciences Géologiques, Bulletin, 38, 79–89.

Lézine, A. M., et al. (1995). Pollen analyses off Senegal: Evolution of the coastal palaeoenvironment during the last deglaciation. Journal of Quaternary Science. https://doi.org/10.1002/jqs.3390100202

Lézine, A. M., et al. (1995). Transport pollinique et circulation atmosphérique au large de l'Afrique tropicale occidentale au cours de la dernière déglaciation. Bulletin de la Société Géologique de France, 1995, 68–76.

Lézine, A. M., et al. (1998). Holocene lakes from Ramlat as-Sab'atayn (Yemen) illustrate the impact of monsoon activity in Southern Arabia. Quaternary Research, 50, 290–299. https://doi.org/10.1006/qres.1998.1996

Lézine, A. M., et al. (2002). Mangroves of Oman during the late Holocene: Climatic implications and impact on human settlements. Journal of Vegetation History and Archaeobotany, 11, 221–232. https://doi.org/10.1007/s003340200025

Lézine, A. M., et al. (2007). Centennial to millennial-scale variability of the Indian monsoon during the early Holocene from a sediment, pollen and isotope record from the desert of Yemen. Palaeogeography, Palaeoclimatology, Palaeoecology, 243, 235–249. https://doi.org/10.1016/j.palaeo.2006.05.019

Lézine, A. M., et al. (2010). Climate change and human occupation in the Southern Arabian lowlands during the last deglaciation and the Holocene. Global and Planetary Change. https://doi.org/10.1016/j.gloplacha.2010.01.016

Lézine, A. M., et al. (2011). Late Holocene plant and climate evolution at Lake. Climate of the Past, 7, 1–2011. https://doi.org/10.5194/cp-7-1-2011

Lézine, A. M., et al. (2017). Timing of the southward retreat of the ITCZ at the end of the Holocene Humid Period in Southern Arabia: Data–model comparison. Quaternary Science Reviews, 164, 68–76. https://doi.org/10.1016/j.quascirev.2017.03.019

Lézine, A., & Hooghiemstra, H. (1990). Land-sea comparisons during the last glacial-interglacial transition: Pollen records from West Tropical Africa. Palaeogeography, Palaeoclimatology, Palaeoecology, 79, 313–331. https://doi.org/10.1016/0031-0182(90)90025-3

Lézine, A., & Hooghiemstra, H. (1990). Land-sea comparisons during the last glacial-interglacial transition: Pollen records from West Tropical Africa. Palaeogeography, Palaeoclimatology, Palaeoecology, 79, 313–331. https://doi.org/10.1016/0031-0182(90)90025-3

Lézine, A.M. unpublished

Lézine, A.-M., Lemonnier, K., & Fofana, C. A. K. (2019). Sahel environmental variability during the last millennium: Insight from a pollen, charcoal, and algae record from the Niayes area, Senegal. Review of Palaeobotany and Palynology. https://doi.org/10.1016/j.revpalbo.2019.104103

Linstädter, J., & Kehl, M. (2012). The Holocene archaeological sequence and sedimentological processes at Ifri Oudadane, NE Morocco. Journal of Archaeological Science, 39(10), 3306–3323. https://doi.org/10.1016/j.jas.2012.05.025

Litt, T., et al. (2009). 'PALEOVAN', International Continental Scientific Drilling Program (ICDP): site survey results and perspectives. Quaternary Science Reviews, 28(15–16), 1555–1567. https://doi.org/10.1016/j.quascirev.2009.03.002

Litt, T., et al. (2012). Holocene climate variability in the Levant from the Dead Sea pollen record. Quaternary Science Reviews, 49, 95–105. https://doi.org/10.1016/j.quascirev.2012.06.012

López-Merino, L., et al. (2009). 2000 years of pastoralism and fire shaping high-altitude vegetation of Sierra de Gredos in central Spain. Review of Palaeobotany and Palynology, 158, 42–51. https://doi.org/10.1016/j.revpalbo.2009.07.003

López-Sáez, J. A. (2011). Paleobotánica en Camino de las Yeseras. VII.1. Análisis palinológicos en el poblado calcolítico de Camino de las Yeseras (San Fernando de Henares, Madrid). In C. Blasco, C. Liesau, & P. Ríos (Eds.), Yacimientos calcolíticos con campaniforme de la región de Madrid: nuevos estudios (pp. 249–261).

López-Sáez, J. A. (2011). Yacimientos calcolíticos con campaniforme de la región de Madrid: nuevos estudios. VII. Paleobotánica en Camino de las Yeseras. VII.1. Análisis palinológicos en el poblado calcolítico de Camino de las Yeseras (San Fernando de Henares, Madrid). In C. Blasco, C. Liesau, & P. Ríos (Eds.), Yacimientos calcolíticos con campaniforme de la región de Madrid: nuevos estudios (pp. 249–261).

López-Sáez, J. A., B. van Geel, S. Farbos-Texier, & M. F. Diot. (1998). Remarques paléoécologiques à propos de quelques palynomorphes non-polliniques provenant de sédiments quaternaires en France. Revue Paléobiologie, 17(2), 445–459.

López-Sáez, J. A., et al. (2009). Paisajes culturales de las villas romanas de Toledo. Cuadernos de la Sociedad Española de Ciencias Forestales, 30, 101–106.

López-Sáez, J. A., et al. (2010). Late Holocene ecological history of Pinus pinaster forests in the Sierra de Gredos of central Spain. Plant Ecology, 206(2), 195–209. https://doi.org/10.1007/s11258-009-9634-z

López-Sáez, J. A., L. López-Merino, & S. Pérez-Díaz. (2008). Historia de la vegetación: una aproximación arqueopalinológica. Antiqvitas, 20, 41–47.

López-Sáez, J. A., M. Sánchez, & P. López. (1999). Evolución del Lanzahíta (Valle del Tiétar, Ávila) durante el Holoceno reciente: Una interpretación palinológica. Trasierra, 4, 81–86.

López-Sáez, J. A., P. López-García, & L. López-Merino. (2010). Zafrín. Un asentamiento del Neolítico antiguo en las islas Chafarinas (Norte de Africa, España). Anexo 1. Paleoambiente de las Islas Chafarinas durante el Neolítico antiguo: análisis polínicos en el yacimiento arqueológico de Zafrín (Isla de Congresco). Studia Archaeologica, 96, 165–175.

López-Sáez, J. A., P. López-García, & M. Cortés-Sánchez. (2007). Chapter VII. Paleovegetación del Cuaternario reciente: Estudio arqueopalinológico. In M. Cortés-Sánchez (Ed.), Cueva Bajondillo (Torremolinos). Secuencia cronocultural y paleoambiental del Cuaternario reciente en la Bahía de Málaga (pp. 131–156). Centro de Ediciones de la Diputación de Málaga; Junta de Andalucía; Universidad de Málaga; Fundación Cueva de Nerja; Fundación Obra Social de Unicaja.

López-Sáez, J. A., P. López-García, & R. Marcías-Rosado. (1997). Acción antrópica y reconstrucción de la vegetación durante el Holoceno reciente en el Valle del Tiétar, Sierra de Gredos (Ávila). Cuaternario y Geomorfología, 11(1–2), 43–54.

López-Sáez, J. A., P. López-García, C. Gómez-Ferreras, & P. Gil-Hernández. (1996). Acerca del origen del castaño (Castanea sativa) en el Valle del Tiétar (Sierra de Gredos, Ávila). In B. Ruiz Zapata et al. (Eds.), Estudios Palinológicos, XI Simposio de Palinología (A.P.L.E) (pp. 79–82). Universidad de Alcalá de Henares.

Luelmo-Lautenschlaeger, R., López-Sáez, J. A., & Pérez-Díaz, S. (2018a). Contribution to the European Pollen Database. 40. Botija, Toledo Mountains (central Spain). Grana, 57(4), 322–324. https://doi.org/10.1080/00173134.2017.1400587

Maley, J. (1980). Les changements climatiques de la fin du Tertiaire en Afrique: Leur conséquence sur l'apparition du Sahara et de sa végétation. In M. Williams & H. Faure (Eds.), The Sahara and the Nile (Vol. 63, pp. 63–86). A. A. Balkema.

Maley, J. (1981). Études palynologiques dans le bassin du Tchad et paléoclimatologie de l'Afrique Nord-Tropicale de 30 000 ans à l'époque actuelle. Review of Palaeobotany and Palynology.

Maley, J. (2000). Last Glacial Maximum lacustrine and fluviatile formations in the Tibesti and other Saharan mountains, and large-scale climatic teleconnections linked to the activity of the Subtropical Jet Stream. Global and Planetary Change, 26, 121–136. https://doi.org/10.1016/s0921-8181(00)00039-4

Maley, J. (2004). Le bassin du Tchad au Quaternaire récent: Formations sédimentaires, paléoenvironnements et préhistoire. La question des paléotchads. In A. M. Sémah & J. Renault-Miskovsky (Eds.), L'évolution de la végétation depuis deux millions d'années (pp. 179–217).

Maley, J., Cohen, J., Faure, H., Rognon, P., & Vincent, P. M. (1970). Quelques formations lacustres et fluviales associées à différentes phases du volcanisme au Tibesti (Nord du Tchad). Cahier ORSTOM, Série Géologie II, 1.

Marks, L., et al. (2019). Cyclonic activity over northeastern Africa at 8.5–6.7 cal kyr BP, based on lacustrine records in the Faiyum Oasis, Egypt. Palaeogeography, Palaeoclimatology, Palaeoecology, 528, 120–132. https://doi.org/10.1016/j.palaeo.2019.04.032

Marriner, N., & Morhange, C. (2005). Under the city centre, the ancient harbour. Tyre and Sidon: Heritages to preserve. Journal of Cultural Heritage, 6, 183–189. https://doi.org/10.1016/j.culher.2005.02.002

Marriner, N., de Beaulieu, J. L., & Morhange, C. (2004). Note on the vegetation landscapes of Sidon and Tyre during antiquity. Archaeology and History in Lebanon, 19, 85–91.

Marriner, N., de Beaulieu, J. L., & Morhange, C. (2006). Geoarchaeological evidence for dredging in Tyre's ancient harbour, Levant. Quaternary Research, 65. https://doi.org/10.1016/j.yqres.2005.07.004

Médus, J. (1975). Spectres palynologiques de deux sédiments du Nouakchottien des environs de Saint-Louis (Sénégal). Bulletin de l'Institut Fondamental d'Afrique Noire, 53, 534–536.

Médus, J. (1981). Données préliminaires sur la palynologie de l'Holocène du delta du fleuve Sénégal. Géobios, 14, 801–805. https://doi.org/10.1016/S0016-6995(81)80153-2

Médus, J. (1984). Analyse pollinique des sédiments holocènes du lac Tanma, Sénégal. Palaeoecology of Africa, 16, 255–264.

Médus, J., & Barbey, P. (1979). Deux analyses polliniques de sédiments minéraux de Mauritanie méridionale. Association Sénégalaise d'Études Quaternaires, 54–55, 75–79.

Médus, J., & Marliac, A. (1997). Un environnement végétal anthropique des abords du XIème siècle sur la rive du Mayo Boula, sud de Maroua, Cameroun septentrional. Colloques et séminaires–Institut français de recherche scientifique pour le développement en coopération, 123–129.

Médus, J., Lappatient, J. R., & Flicoteaux, R. (1981). Faune, palynoflore et argiles du Quaternaire du lac Tanma (Sénégal, Cap Vert). Oceanis, 7, 431–438.

Médus, J., Malléa, M., Marliac, A., & Mathieu, P. (1997). Pollenanalyses et mycoflore de dépôts récents de terrasses fluviatiles du Cameroun septentrional. Géobios, 30, 213–221.

Mercuri, A. (2008). Human influence, plant landscape evolution and climate inferences from the archaeobotanical records of the Wadi Teshuinat area (Libyan Sahara). Journal of Arid Environments, 72, 1950–1976. https://doi.org/10.1016/j.jaridenv.2008.04.008

Mercuri, A. M. (2001). Palynological analyses of the Late Pleistocene, Early Holocene and Middle Holocene layers. In E. A. A. Garcea (Ed.), Uan Tabu in the settlement history of the Libyan Sahara (pp. 161–188, 237–251). Firenze: All'Insegna del Giglio.

Mercuri, A. M. (2015). Humans and water in desert "refugium" areas: Palynological evidence of climate oscillations and cultural developments in early and mid-Holocene Saharan edges. Interdisciplinaria Archaeologica. Natural Sciences in Archaeology, 6, 151–160. https://doi.org/10.24916/iansa.2015.2.2

Mercuri, A. M., Cremaschi, M., & di Lernia, S. (1998). New pollen data from the Uan Muhuggiag rockshelter (Libyan Sahara, VII–IV millennia BP). In M. Cremaschi & S. di Lernia (Eds.), Wadi Teshuinat. Palaeoenvironment and prehistory in south-western Fezzan (pp. 107–122). Firenze: Edizioni All'Insegna del Giglio.

Michel, P., & Asémien, P. (1969). Etudes sédimentologique et palynologique des sondages de Bogué (basse vallée du Sénégal) et leur interprétation morphoclimatique. Revue de Géomorphologie Dynamique, 1997–113.

Miebach, A., Niestrath, P., Roeser, P. A., & Litt, T. (2016). Impacts of climate and humans on the vegetation in northwestern Turkey: Palynological insights from Lake Iznik since the Last Glacial. Climate of the Past, 12, 575–593. https://doi.org/10.5194/cp-12-575-2016, 2016.

Miller, C. S., Leroy, S. A. G., Collins, P. E. F., & Lahijani, H. A. K. (2016). Late Holocene vegetation and ocean variability in the Gulf of Oman. Quaternary Science Reviews, 143, 120–132. https://doi.org/10.1016/j.quascirev.2016.05.010

Moe, D., & van der Knaap, W. O. (1990). Transhumance in mountain areas: Additional interpretation of three pollen diagrams from Norway, Portugal and Switzerland. In PACT 31: Impact of prehistoric and medieval man on the vegetation: Man at the forest limit. Report of the meeting held at Ravello, December 9 and 10, 1990. Rixensiart, Belgium: Pact Network, Palaeoecology.

Mohammed, M. U. (1992). Paléoenvironnement et paléoclimatologie des derniers millénaires en Ethiopie. Contribution palynologique (Thèse, Université Aix-Marseille III).

Mohammed, M. U., & Bonnefille, R. (1992). The recent history of vegetation and climate around Lake Langeno (Ethiopia). Palaeoecology of Africa, 11, 135–152.

Morales, J., et al. (2013). The origins of agriculture in North-West Africa: Macro-botanical remains from Epipalaeolithic and Early Neolithic levels of Ifri Oudadane (Morocco). Journal of Archaeological Science, 40(6), 2659–2669. https://doi.org/10.1016/j.jas.2013.01.026

Morales-Molino, C., Colombaroli, D., Valbuena-Carabaña, M., Tinner, W., Salómon, R. L., Carrión, J. S., & Gil, L. (2017). Land-use history as a major driver for long-term forest dynamics in the Sierra de Guadarrama National Park (central Spain) during the last millennia: Implications for forest conservation and management. Global and Planetary Change, 152, 64–75. https://doi.org/10.1016/j.gloplacha.2017.02.012

Morales-Molino, C., García-Antón, M., Postigo-Mijarra, J. M., & Morla, C. (2013). Holocene vegetation, fire and climate interactions on the westernmost fringe of the Mediterranean Basin. Quaternary Science Reviews, 59, 5–17. https://doi.org/10.1016/j.quascirev.2012.10.027

Morel, A., & Schulz, E. (1981). Recherches palynologiques et géomorphologiques dans le massif de l'Aïr (Niger): Note préliminaire. Revue de Géographie Alpine, 69, 583–592. https://doi.org/10.3406/rga.1981.2481

Morhange, C., Dubuquoy, O., Prunet, N., Ribes, E., de Beaulieu, J. L., Bourcier, M., Carbonel, P., Oberlin, C., & Doumet-Serhal, C. (2000). Étude paléoenvironnementale du port antique de Sidon. Premiers résultats du programme CEDRE. Méditerranée, 94, 91–100.

Muller, S., Cheddadi, R., Giardini, M., et al. (2015). Vegetation history of the western Rif mountains (NW Morocco): Origin, late-Holocene dynamics and human impact. Vegetation History and Archaeobotany, 24, 487–501. https://doi.org/10.1007/s00334-014-0504-9

Neumann, K., & Ballouche, A. (1992). Die Chaine de Gobnangou in SE Burkina Faso – Ein Beitrag zur Vegetationsgechichte der Sudanzone W-Afrikas. Geobotanisches Kolloquium, 8, 53–68.

Neumann, K., Fahmy, A., Lespez, L., Ballouche, A., & Huysecom, E. (2009). The Early Holocene palaeoenvironment of Ounjougou (Mali): Phytoliths in a multiproxy context. Palaeogeography, Palaeoclimatology, Palaeoecology, 276, 87–106. https://doi.org/10.1016/j.palaeo.2009.03.001

Nimmergut, A. P., Allen, J. R. M., Jones, V. J., Huntley, B., & Battarbee, R. W. (1999). Submillennial environmental fluctuations during marine Oxygen Isotope Stage 2: A comparative analysis of diatom and pollen evidence from Lago Grande di Monticchio, South Italy. Journal of Quaternary Science, 14, 111–123. https://doi.org/10.1002/(SICI)1099-1417(199903)14:2<111::AID-JQS427>3.0.CO;2-A

Noti, R., van Leeuwen, J. F. N., Colombaroli, D., Vescovi, E., Pasta, S., La Mantia, T., & Tinner, W. (2009). Mid- and late-Holocene vegetation and fire history at Biviere di Gela, a coastal lake in southern Sicily, Italy. Vegetation History and Archaeobotany, 18(5), 371–387. https://doi.org/10.1007/s00334-009-0218-6

Nourelbait, M., Rhoujjati, A., Eynaud, F., Benkaddour, A., Dezileau, L., Wainer, K. A. I., Goslar, T., Khater, C., & Cheddadi, R. (2014). An 18 000-year pollen and sedimentary record from the cedar forest of the Middle Atlas, Morocco. Journal of Quaternary Science, 29, 417–426. https://doi.org/10.1002/jqs.2708

Pantaléon-Cano, J., et al. (2003). Palynological evidence for vegetational history in semi-arid areas of the western Mediterranean (Almeria, Spain). Holocene, 13, 109–119. https://doi.org/10.1191/0959683603hl598r

Paquereau, V. (n.d.). Evolution de la mangrove ouest africaine au cours de la dernière déglaciation: Analyse palynologique de la carotte A-18056. Mémoire du Magistère de l'Ecole Normale Supérieure, Unpublished.

Parker, A. G., Eckersley, L., Smith, M. M., Goudie, A. S., Stokes, S., Ward, S., White, K., & Hodson, M. J. (2004). Holocene vegetation dynamics in the northeastern Rub' al-Khali desert, Arabian Peninsula: A phytolith, pollen and carbon isotope study. Journal of Quaternary Science, 19, 665–676. https://doi.org/10.1002/jqs.880

Pedrotta, T., Gobet, E., Schwörer, C., Beffa, G., Butz, C., Henne, P. D., Morales-Molino, C., Pasta, S., van Leeuwen, J. F. N., Vogel, H., Zwimpfer, E., Anselmetti, F. S., Grosjean, M., & Tinner, W. (2021). 8,000 years of climate, vegetation, fire and land-use dynamics in the thermo-mediterranean vegetation belt of northern Sardinia (Italy). Vegetation History and Archaeobotany, 30, 789–813. https://doi.org/10.1007/s00334-021-00832-3

Petit-Maire, N. (1981). Holocene lake deposits and palaeoenvironments in Central Sahara, Northeastern Mali. Palaeogeography, Palaeoclimatology, Palaeoecology, 35, 45–61. https://doi.org/10.1016/0031-0182(81)90093-6

Pons, A., & Quézel, P. (1957). Première étude palynologique de quelques paléosols sahariens. Travaux de l'Institut de Recherches Sahariennes, 14, 15–40.

Pons, A., & Quézel, P. (1958). Premières remarques sur l'étude palynologique d'un guano fossile du Hoggar. Compte-Rendus de l'Académie des Sciences, Paris, 246, 2290–2292.

Pons, A., & Reille, M. (1988). The Holocene- and upper Pleistocene pollen record from Padul (Granada, Spain): A new study. Palaeogeography, Palaeoclimatology, Palaeoecology, 66, 243–263. https://doi.org/10.1016/0031-0182(88)90202-7

Quézel, P., & Martinez, C. (1958). Etude palynologique de deux diatomites du Borkou (Territoire du Tchad A.E.F.). Bulletin de la Société d'Histoire Naturelle de l'Afrique du Nord, 49, 230–244.

Quézel, P., & Martinez, C. (1962). Premiers résultats de l'analyse palynologique de sédiments recueillis au Sahara méridional à l'occasion de la mission Berliet-Tchad. Missions Berliet Ténéré-Tchad, Paris, 313, 327.

Ravazzi, C., Mariani, M., Criado, C., Garozzo, L., Naranjo-Cigala, A., Perez-Torrado, F. J., Pini, R., Rodriguez-Gonzalez, A., Nogué, S., Whittaker, R. J., Fernández-Palacios, J. M., & Nascimento, L. de. (2020). The influence of natural fire and cultural practices on island ecosystems: Insights from a 4800 year record from Gran Canaria, Canary Islands. Journal of Biogeography. https://doi.org/10.1111/jbi.13995

Ritchie, J. C. (1984). Analyse pollinique de sédiments Holocènes Supérieurs des Hauts Plateaux du Maghreb Oriental. Pollen et Spores, 26, 489–496.

Ritchie, J. C. (1986). Modern pollen spectra from Dakhleh Oasis, Western Egyptian Desert. Grana, 25, 177–182. https://doi.org/10.1080/00173138609427719

Ritchie, J. C. (1994). Holocene pollen spectra from Oyo, northwestern Sudan: Problems of interpretation in a hyperarid environment. Holocene, 4, 9–15. 1. https://doi.org/10.1177/095968369400400102"

Ritchie, J. C., & Haynes, C. V. (1987). Holocene vegetation zonation in the eastern Sahara. Nature, 330, 645–647. https://doi.org/10.1038/330645a0

Ritchie, J. C., Eyles, C. H., & Haynes, C. V. (1985). Sediment and pollen evidence for an early to mid-Holocene humid period in the eastern Sahara. Nature, 314(6009), 352–355. https://doi.org/10.1038/314352a0

Robles López, S., Manzano-Rodríguez, S., Pérez-Díaz, S., & López-Sáez, J. A. (2017). Contributions to the European Pollen Database. 35. Labradillos mire, Gregos Ranges (central Spain). Grana, 56(5), 398–400. https://doi.org/10.1080/00173134.2017.1282976

Roeser, P. A., Franz, S. O., Litt, T., Ülgen, U. B., Hilgers, A., Wulf, S., Wennrich, V., Akçer Ön, S., Viehberg, F. A., Çağatay, M. N., & Melles, M. (2012). Lithostratigraphic and geochronological framework for the palaeoenvironmental reconstruction of the last ~36 ka cal BP from a sediment record from Lake Iznik (NW Turkey). Quaternary International, 274, 73–87. https://doi.org/10.1016/j.quaint.2012.01.023

Rossignol-Strick, M., & Duzer, D. (1979). Quaternary pollen and dinoflagellate cysts in marine cores off West Africa. Meteor Forschungsergebnisse, Deutsche Forschungsgemeinschaft, Reihe C, 30, 1–46.

Rossignol-Strick, M., & Duzer, D. (1979). West African vegetation and climate since 22500 BP from deep-sea cores palynology. Pollen et Spores.

Saad, S. I. (1967). Studies of pollen and spores content of Nile Delta deposits (Berenbal region). Pollen et Spores, 9, 467–503.

Salzmann, U. (1996). Holocene vegetation history of Sahelian-zone of NE-Nigeria: Preliminary results. Palaeoecology of Africa, 24, 103–114.

Salzmann, U. (1999). Zur holozänen vegetations- und klimaentwicklung der westafrikanischen savannen: Paläoökologische untersuchungen in der Sahel- und Sudanzone NO-Nigerias (PhD Thesis, University of Wuerzburg, Frankfurt).

Salzmann, U. (2000). Are modern savannas degraded forests? A Holocene pollen record from the Sudanian vegetation zone of NE Nigeria. Vegetation History and Archaeobotany, 9, 1–15. https://doi.org/10.1007/BF01295010

Salzmann, U. (2002). Late Quaternary climate and vegetation of the Sudanian Zone of northeast Nigeria. Quaternary Research, 58(1), 73–83. https://doi.org/10.1006/qres.2002.2356

Salzmann, U., & Waller, M. (1998). The Holocene vegetational history of the Nigerian Sahel based on multiple pollen profiles. Review of Palaeobotany and Palynology, 100, 39–72. https://doi.org/10.1016/s0034-6667(97)00053-5

Sanchez-Goñi, M. F., Cacho, I., Turon, J. L., Guiot, J., Sierro, F. J., Peypouquet, J. P., Grimalt, J. O., & Shackleton, N. J. (2002). Synchroneity between marine and terrestrial responses to millennial-scale variability during the last glacial period in the Mediterranean region. Climate Dynamics, 19, 95–105. https://doi.org/10.1007/s00382-001-0212-x

Schiebel, V., & Litt, T. (2017). Holocene vegetation history of the southern Levant based on a pollen record from Lake Kinneret (Sea of Galilee), Israel. Vegetation History and Archaeobotany, 27, 577–590. https://doi.org/10.1007/s00334-017-0658-3

Schultze, R. (1994). Les indicateurs limniques holocènes au Nord-Est du Niger - Les pigments des plantes, signaux de l'existence des communautés algaires et bactériennes planctoniques et benthiques. In R. Maire, S. Pomel, & J. N. Salomon (Eds.), Enregistreurs et indicateurs de l'évolution de l'environnement en zone tropicale. Presses Universitaires de Bordeaux.

Schulz, E. (1973). Zur quartären Vegetationsgeschichte der zentralen Sahara unter Berücksichtigung eigener pollenanalystischer Untersuchungen aus dem Tibesti-Gebirge. Geographische Arbeiten.

Schulz, E. (1976). Aktueller Pollenniederschlag in der zentralen Sahara und Interpretationsmöglichkeiten quartärer Pollenspektren. Palaeoecology of Africa, 9, 8–14.

Schulz, E. (1980). Zur Vegetation der östlichen zentralen Sahara und zu ihrer Entwicklung im Holozän. Würzburger Geographische Arbeiten, 51, 194 p.

Schulz, E. (1991). Holocene environments in the central Sahara. Hydrobiologia, 214, 359–365. https://doi.org/10.1007/BF00050971

Schulz, E., & Whitney, J. W. (1986). Upper Pleistocene and Holocene lakes in the An Nafud, Saudi Arabia. Hydrobiologia, 143, 175–190. https://doi.org/10.1007/BF00026660

Schulz, E., Pomel, S., Abichou, A., & Salzmann, U. (1995). Climate and man. Questions and answers from both sides of the Sahara. 2e symposium de palynologie africaine/2nd Symposium on African Palynology, Tervuren, Belgique, 35–47.

Schwab, M. J., Neumann, F. H., Litt, T., Negendank, J. F. W., & Stein, M. (2004). Holocene palaeoecology of the Golan Heights (Near East): Investigation of lacustrine sediments from Birkat Ram crater lake. Quaternary Science Reviews, 23, 1723–1731. https://doi.org/10.1016/j.quascirev.2004.05.001

Schwörer, C., Morales-Molino, C., Gobet, E., Henne, P. D., Pasta, S., Pedrotta, T., van Leeuwen, J. F. N., Vannière, B., & Tinner, W. (2024). Simulating past and future fire impacts on Mediterranean ecosystems. Journal of Ecology. https://doi.org/10.1111/1365-2745.14293

Sereno, P., El-Haj, M., & Kuper, M. B. (2008). Lakeside Cemeteries in the Sahara: 5000 Years of Holocene Population and Environmental Change. PLoS ONE, 3. https://doi.org/10.1371/journal.pone.0002995

Servera-Vives, G., Mus Amezquita, M., Snitker, G., Florenzano, A., Torri, P., Estrany Bertos, J., & Mercuri, A. M. (2022). Modern analogs for understanding pollen-vegetation dynamics in a Mediterranean mosaic landscape (Balearic Islands, Western Mediterranean). The Holocene, 32(7), 716–734. https://doi.org/10.1177/09596836221088229

Stambouli-Essassi, S., Roche, E. & Bouzid, S. Evolution de la végétation et du climat dans le Nord-ouest de la Tunisie au cours des 40 derniers millénaires. Geo-Eco-Trop. 31, 171–214 (2007).

Stambouli-Essassi, S., Roche, E., & Bouzid, S. (2007). Evolution de la végétation et du climat dans le Nord-ouest de la Tunisie au cours des 40 derniers millénaires. Geo-Eco-Trop, 31, 171–214.

Stanley, D. J., Sheng, H., & Pan, Y. (1988). Heavy minerals and provenance of late Quaternary sands, eastern Nile Delta. Journal of African Earth Sciences, 7, 735–741.

Stevenson, A. C. (1984). Studies in the vegetational history of S.W. Spain. III. Palynological investigations at El Asperillo, Huelva. Journal of Biogeography, 11, 527–551. https://doi.org/10.2307/2844798

Thinon, M., Ballouche, A., & Reille, M. (1996). Holocene vegetation of the Central Saharan Mountains: the end of a myth. The Holocene, 6(4), 457–462. https://doi.org/10.1177/095968369600600408

Tinner, W., van Leeuwen, J. F. N., Colombaroli, D., Vescovi, E., van der Knaap, W. O., Henne, P. D., Pasta, S., D'Angelo, S., & La Mantia, T. (2009). Holocene environmental and climatic changes at Gorgo Basso, a coastal lake in southern Sicily, Italy. Quaternary Science Reviews, 28, 1498–1510. https://doi.org/10.1016/j.quascirev.2009.02.001

Tinner, W., Vescovi, E., van Leeuwen, J. F. N., Colombaroli, D., Henne, P. D., Kaltenrieder, P., Morales-Molino, C., Beffa, G., Gnaegi, B., van der Knaap, W. O., La Mantia, T., & Pasta, S. (2016). Holocene vegetation and fire history of the mountains of Northern Sicily (Italy). Vegetation History and Archaeobotany, 25, 499–519. https://doi.org/10.1007/s00334-016-0569-8

Tissot, C., Marius, C., & Feller, C. (1983). Continuité des paléofaciès palynologiques et physico-chimiques de sédiments récents en milieu de mangrove du Sénégal. In Géomorphologie littorale. Travaux et Documents de Géographie Tropicale CEGET, 49, 100–115.

Tovar, C., Harris, D. J., Breman, E., Brncic, T., & Willis, K. J. (2019). Tropical monodominant forest resilience to climate change in Central Africa: A Gilbertiodendron dewevrei forest pollen record over the past 2,700 years. Journal of Vegetation Science, 30, 575–586. https://doi.org/10.1111/jvs.12746

Turner, J., & Greig, J. R. A. (1975). Some Holocene pollen diagrams from Greece. Review of Palaeobotany and Palynology, 20, 171–204. https://doi.org/10.1016/0034-6667(75)90020-2

Tzedakis, P. C. (1993). Long-term tree populations in northwest Greece through multiple Quaternary climatic cycles. Nature, 364, 437–440. https://doi.org/10.1038/364437a0

Tzedakis, P. C. (1994). Vegetation change through glacial–interglacial cycles: A long pollen sequence perspective. Philosophical Transactions of the Royal Society of London. Series B: Biological Sciences, 345(1314), 403–432. https://doi.org/10.1098/rstb.1994.0118

Urban, B., & Buerkert, A. (2009). Palaeoecological analysis of a Late Quaternary sediment profile in northern Oman. Journal of Arid Environments, 73, 296–305. https://doi.org/10.1016/j.jaridenv.2008.09.023

Vallé, F., Dupont, L. M., Leroy, S. A. G., Schefuß, E., & Wefer, G. (2014). Pliocene environmental change in West Africa and the onset of strong NE trade winds (ODP Sites 659 and 658). Palaeogeography, Palaeoclimatology, Palaeoecology. https://doi.org/10.1016/j.palaeo.2014.09.023

Van Campo, M., Cohen, J., Guinet, P., & Rognon, P. (1965). Contribution à l'étude du peuplement végétal quaternaire des montagnes sahariennes. II-Flore contemporaine d'un gisement de mammifères tropicaux dans l'Atakor. Pollen Spores, 7, 361–371.

Van Campo, M., Guinet, P., & Cohen, J. (1968). Fossil pollen from late Tertiary and middle Pleistocene deposits of the Kurkur oasis. In K. W. Butzer & C. L. Hansen (Eds.), Desert and River in Nubia. University of Wisconsin Press.

Van Campo, M., Guinet, P., Cohen, J., & Dutil, P. (1966). Nouvelle flore pollinique des alluvions pléistocènes d'un versant Sud du Hoggar. Comptes Rendus Acad. Sci. Paris, 263/D, 487–490.

van der Brink, L. M., & Janssen, C. R. (1985). The effect of human activities during cultural phases on the development of montane vegetation in the Serra da Estrela, Portugal. Review of Palaeobotany and Palynology, 44, 193–215. https://doi.org/10.1016/0034-6667(85)90016-8

van der Knaap, W. O. (Pim). Unpublished.

van der Knaap, W. O., & van Leeuwen, J. F. N. (1994). Holocene vegetation, human impact, and climatic change in the Serra da Estrela, Portugal. Dissertationes Botanicae, 234, 497–535.

van der Knaap, W. O., & van Leeuwen, J. F. N. (1995). Holocene vegetation succession and degradation as responses to climatic change and human activity in the Serra da Estrela, Portugal. Review of Palaeobotany and Palynology, 89(1–2), 153–211. https://doi.org/10.1016/0034-6667(95)00040-2

van der Knaap, W. O., & van Leeuwen, J. F. N. (1997). Late-Glacial and early Holocene vegetation succession, altitudinal vegetation zonation, and climatic change in the Serra da Estrela, Portugal. Review of Palaeobotany and Palynology, 97, 239–285. https://doi.org/10.1016/S0034-6667(97)00008-0

van Dongen, F. H. (1989). Palynologisch onderzoek aan de afzettingen van Lagoacho das Favas, Serra da Estrela, Portugal (Master's thesis). University of Utrecht, Utrecht, The Netherlands.

van Leeuwen, Jacqueline; van der Knaap, W. O. (Pim) Unpublished

van Zeist, W., & Woldring, H. (1978). A postglacial pollen diagram from Lake Van in east Anatolia. Review of Palaeobotany and Palynology, 26, 249–276. https://doi.org/10.1016/0034-6667(78)90015-5

van Zeist, W., Baruch, U., & Bottema, S. (2009). Holocene palaeoecology of the Hula area, northeastern Israel. In K. Kaptijn & L. P. Petit (Eds.), A Timeless Vale: Archaeological and Related Essays on the Jordan Valley in Honour of Gerrit Van Der Kooij on the Occasion of his Sixty-Fifth Birthday (pp. 29–64). Leiden University Press, Leiden.

van Zeist, W., Timmers, R. A., & Bottema, S. (1968). Studies of modern and Holocene pollen precipitation in southeastern Turkey. Paleohistoria, 14, 19–39.

van Zeist, W., Woldring, H., & Stapert, D. (1975). Late Quaternary vegetation and climate of southwestern Turkey. Palaeohistoria, 17, 53–144.

Vignola, C., Sadori, L., Masi, A., Kouli, K., Triantaphyllou, M., Francke, A., Wagner, B., Peyron, O., Mercuri, A. M., & Florenzano, A. (2022). Mid–late Holocene vegetation history of the Argive Plain (Peloponnese, Greece) as inferred from a pollen record from ancient Lake Lerna. PLoS ONE, 17(4), e0266601. https://doi.org/10.1371/journal.pone.0266601

Waller, M. P., & Salzmann, U. (1999). Holocene vegetation changes in the Sahelian zone of NE Nigeria: The detection of anthropogenic activity. Palaeoecology of Africa, 26, 85–102.

Waller, M. P., Street-Perrott, F. A., & Wang, H. (2007). Holocene vegetation history of the Sahel: pollen, sedimentological and geochemical data from Jikariya Lake, north-eastern Nigeria. Journal of Biogeography, 34, 1575–1590. https://doi.org/10.1111/j.1365-2699.2007.01721

Watts, W. A. (1985). A long pollen record from laghi di Monticchio, southern Italy. Journal of the Geological Society, 142, 491–499. https://doi.org/10.1144/gsjgs.142.3.0491

Watts, W. A., Allen, J. R. M., & Huntley, B. (1996). Vegetation history and palaeoclimate of the last glacial period at Lago Grande di Monticchio, southern Italy. Quaternary Science Reviews, 15, 133–153. https://doi.org/10.1016/0277-3791(95)00093-3

Watts, W. A., Allen, J. R. M., & Huntley, B. (2000). Palaeoecology of three interstadial events during oxygen-isotope Stages 3 and 4: a lacustrine record from Lago Grande di Monticchio, southern Italy. Palaeogeography, Palaeoclimatology, Palaeoecology, 155, 83–93. https://doi.org/10.1016/S0031-0182(99)00096-6

Watts, W. A., Allen, J. R. M., Huntley, B., & Fritz, S. C. (1996). Vegetation history and climate of the last 15,000 years at Laghi di Monticchio, southern Italy. Quaternary Science Reviews, 15, 113–132. https://doi.org/10.1016/0277-3791(95)00038-0

Whitney, J. W., Faulkender, D. J., & Rubin, M. (1993). The environmental history and present condition of Saudi Arabia's northern sand seas (U.S. Geological Survey, Open-File Report 83-759). U.S. Geological Survey. https://doi.org/10.3133/ofr83759

Willis, K. J. (1992). The late Quaternary vegetational history of northwest Greece. II. Rezina marsh. New Phytologist, 121, 119–138. https://doi.org/10.1111/j.1469-8137.1992.tb01097.x

Winn, K., Sarnthein, M., & Erlenkeuser, H. (1991). δ18O stratigraphy and chronology of Kiel sediment cores from the East Atlantic. Berichte und Repertorium Geologie und Paläontologie, Institut der Universität Kiel, 45, 99 pp.

Wulf, S., Kraml, M., Brauer, A., Keller, J., & Negendank, J. F. W. (2004). Tephrochronology of the 100 ka lacustrine sediment record of Lago Grande di Monticchio (southern Italy). Quaternary International, 122, 7–30. https://doi.org/10.1016/j.quaint.2004.01.028

Ybert, J. P. (1977). Les pollens atmosphériques en Côte d'Ivoire et au Tchad. Unpublished.

Yll, E. I., Cubas, P., de Beaulieu, J. L., & de Beaulieu, J. L. (1994). Vegetational change in the Balearic Islands (Spain) during the Holocene. Historical Biology, 9(1), 83–89. https://doi.org/10.1080/10292389409380541

Yll, E. I., Pérez-Obiol, R. P., Pantaléon-Cano, J., & Roure, J. M. (1995). Dinamica del paisaje vegetal en la vertiente Mediterranea de la peninsula Iberica e islas Baleares desde el tardig glaciar hasta el presente. In T. Aleixandre & A. Perez (Eds.), Reconstituction de la ambiente y cambios climaticos durante el Cuaternarion (pp. 319–328). Centro de Ciencias Medio, Madrid, Spain.

Yll, E. I., Pérez-Obiol, R. P., Pantaléon-Cano, J., & Roure, J. M. (1997). Palynological evidence for climatic change and human activity during the Holocene on Minorca (Balearic Islands). Quaternary Research, 48, 339–347. https://doi.org/10.1006/qres.1997.1925

Yll, E. I., Roure, J. M., Pantaléon-Cano, J., & Pérez-Obiol, R. P. (1994). Analisis polinico de una secuencia holocenica en Roquetas de Mar (Almeria). In M. Mateu, J. Guemes & M. E. Burgaz (Eds.), Trabajos de palinologia basica y aplicada (pp. 189–198). Universitat de Valencia, Valencia, Spain.

Zaky, A. S. (2020). Mid-to Late Holocene paleoclimatic changes and paleoenvironmental shifts in Egypt. Quaternary International, 542, 109–120. https://doi.org/10.1016/j.quaint.2020.03.024

Zapata, L., et al. (2013). Holocene environmental change and human impact in NE Morocco: Palaeobotanical evidence from Ifri Oudadane. The Holocene, 23(9), 1286–1296. https://doi.org/10.1177/0959683613486944

Zielhofer, C., Fletcher, W. J., Mischke, S., De Batist, M., Campbell, J. F. E., Joannin, S., Tjallingii, R., El Hamouti, N., Junginger, A., Stele, A., Bussmann, J., Schneider, B., Lauer, T., Spitzer, K., Strupler, M., Brachert, T., & Mikdad, A. (2017). Atlantic forcing of the Western Mediterranean winter rain minima during the last 12,000 years. Quaternary Science Reviews, 157, 29–51. https://doi.org/10.1016/j.quascirev.2016.11.037

Zolitschka, B., & Negendank, J. F. W. (1993). Lago Grande di Monticchio (southern Italy): A high-resolution sedimentary record of the last 70,000 years. In Paleolimnology of European Maar Lakes (Lecture Notes in Earth Sciences, 49, pp. 277–288). Springer-Verlag.

Zolitschka, B., & Negendank, J. F. W. (1996). Sedimentology, dating and palaeoclimatic interpretation of a 76.3 ka record from Lago Grande di Monticchio, southern Italy. Quaternary Science Reviews, 15, 101–112. https://doi.org/10.1016/0277-3791(95)00022-4

2. References: taxonomic classification by family and order

Angiosperm Phylogeny Group, Chase, M. W., Christenhusz, M. J., Fay, M. F., Byng, J. W., Judd, W. S., ... & Stevens, P. F. (2016). An update of the Angiosperm Phylogeny Group classification for the orders and families of flowering plants: APG IV. Botanical journal of the Linnean Society, 181(1), 1-20.

Anissimova, O. V. (2016). Architecture of cell wall of Euastrum Ralfs new genus critheria. Moscow University Biological Sciences Bulletin, 71(3), 155-159.

Bhagya, A. T., Phukhamsakda, C., Jones, E. G., & Hyde, K. D. (2025). Unveiling a novel Zopfiella species (Lasiosphaeriaceae, Sordariales): morphological characteristics and multigene phylogeny from Carex sp.,(Cyperaceae) in Thailand. New Zealand Journal of Botany, 1-12.

Buchheim, M., Buchheim, J., Carlson, T., Braband, A., Hepperle, D., Krienitz, L., ... & Hegewald, E. (2005). Phylogeny of the Hydrodictyaceae (Chlorophyceae): inferences from rDNA data 1. Journal of phycology, 41(5), 1039-1054.

Cai, L., Jeewon, R., & Hyde, K. D. (2005). Phylogenetic evaluation and taxonomic revision of Schizothecium based on ribosomal DNA and protein coding genes. Fungal Diversity.

Cain, R. F. (1956). Studies of Coprophilous Ascomygetes: IV. Tripterospora, a New Cleistocarpous Genus in a New Family. Canadian Journal of Botany, 34(4), 699-710.

Calabon, M. S., Hyde, K. D., Jones, E. B. G., Bao, D. F., Bhunjun, C. S., Phukhamsakda, C., ... & Balasuriya, A. (2023). Freshwater fungal biology. Mycosphere, 14(1), 195-413.

Chethana, T., Liu, M., Ariyawansa, H. A., Konta, S., Wanasinghe, D. N., Zhou, Y., ... & Li, X. (2015). Splanchnonema-like species in Pleosporales introducing Pseudosplanchnonema gen. nov. in Massarinaceae. Phytotaxa, 231(2), 133-144.

Cole, T. C., Bachelier, J. B., & Hilger, H. H. (2017). Tracheophyte phylogeny poster-Vascular plants: systematics and characteristics. PeerJ Preprints.

Cole, T. C., Hilger, H. H., & Goffinet, B. (2019). Bryophyte phylogeny poster (BPP). PeerJ Preprints.

Dávila, C., & Toranzos, G. A. (2020). Effect of nutrient availability on lipid productivity of Botryococcus sp.(Botryococcaceae, Chlorophyta), a newly isolated tropical microalgae strain from Puerto Rico. Caribbean Journal of Science, 50(1), 60-73.

Deason, T. R., & Floyd, G. L. (1987). Comparative Ultrastructure of Three Species of Chlorosarcina (chlorosarcinaceae, Chlorophyta) 1, 2. Journal of phycology, 23(1), 187-195.

Denboh, T., Hendrayanti, D., & Ichimura, T. (2001). Monophyly of the genus Closterium and the order Desmidiales (Charophyceae, Chlorophyta) inferred from nuclear small subunit rDNA data. Journal of Phycology, 37(6), 1063-1072.

Dubey, M. K., Shah, Z., Upadhyay, R. S., & Gupta, R. C. (2019). First report of Brachysporium britannicum (Trichosphaeriaceae) from India. Indian Phytopathology, 72(3), 555-559.

Fensome, R. A., & Williams, G. L. (2017). The Lentin and Williams Index of Fossil Dinoflagellates (Contribution Series 42, 909 pp.). Dallas, TX: American Association of Stratigraphic Palynologists Foundation.

Gabyshev, V., Davydov, D., Vilnet, A., Sidelev, S., Chernova, E., Barinova, S., ... & Zhakovskaya, Z. (2023). Gloeotrichia cf. natans (Cyanobacteria) in the Continuous Permafrost Zone of Buotama River, Lena Pillars Nature Park, in Yakutia (Russia). Water, 15(13), 2370.

García, D., Stchigel, A. M., Cano, J., Calduch, M., Hawksworth, D. L., & Guarro, J. (2006). Molecular phylogeny of Coniochaetales. Mycological research, 110(11), 1271-1289.

Gontcharov, A. A., & Melkonian, M. (2008). In search of monophyletic taxa in the family Desmidiaceae (Zygnematophyceae, Viridiplantae): the genus Cosmarium. American journal of botany, 95(9), 1079-1095.

Gu, H., Mertens, K. N., Derrien, A., Bilien, G., Li, Z., Hess, P., ... & Shin, H. H. (2022). Unraveling the Gonyaulax baltica Species Complex Cyst–theca Relationship of Impagidinium variaseptum, Spiniferites pseudodelicatus sp. nov. and S. ristingensis (Gonyaulacaceae, Dinophyceae), With Descriptions of Gonyaulax bohaiensis sp. nov, G. amoyensis sp. nov. and G. portimonensis sp. nov. Journal of Phycology, 58(3), 465-486.

Guiry, M. D. (2013). Taxonomy and nomenclature of the Conjugatophyceae (= Zygnematophyceae). Algae, 28(1), 1-29.

Gurdebeke, P. R., Mertens, K. N., Pospelova, V., Matsuoka, K., Li, Z., Gribble, K. E., ... & Louwye, S. (2020). Taxonomic revision, phylogeny, and cyst wall composition of the dinoflagellate cyst genus Votadinium Reid (Dinophyceae, Peridiniales, Protoperidiniaceae). Palynology, 44(2), 310-335.

Hawksworth, D. L., van Geel, B., & Wiltshire, P. E. J. (2016). The enigma of the Diporotheca palynomorph. Review of Palaeobotany and Palynology, 235, 94–98. doi:10.1016/j.revpalbo.2016.09.010

Head, M. J. (1992). Zygospores of the Zygnemataceae (Division Chlorophyta) and other freshwater algal spores from the uppermost Pliocene St. Erth Beds of Cornwall, southwestern England. Micropaleontology, 237-260.

Head, M. J. (1996). Modern dinoflagellate cysts and their biological affinities. Palynology: principles and applications, 3, 1197-1248.

Head, M. J., Mertens, K. N., & Fensome, R. A. (2024). Dual nomenclature in organic-walled dinoflagellate cysts I: concepts, methods and applications. Palynology, 48(2), 2290200.

Head, M. J., Pospelova, V., Radi, T., & Marret, F. (2020). Stelladinium bifurcatum n. sp., a distinctive extant thermophilic heterotrophic dinoflagellate cyst from the late Quaternary of the eastern Pacific and east equatorial Atlantic oceans. Marine Micropaleontology, 159, 101754.

Hegewald, E. (1997). Taxonomy and phylogeny of Scenedesmus. Algae, 12(4), 235-246.

Hegewald, E., Wolf, M., Keller, A., Friedl, T., & Krienitz, L. (2010). ITS2 sequence-structure phylogeny in the Scenedesmaceae with special reference to Coelastrum (Chlorophyta, Chlorophyceae), including the new genera Comasiella and Pectinodesmus. Phycologia, 49(4), 325-335.

Herrera, G. M. R., & González, S. G. M. (2014). A revision of the genus Kretzschmaria (Ascomycota, Xylariaceae) in Cuba. Willdenowia, 44(1), 57-64.

Huang, S. K., Hyde, K. D., Maharachchikumbura, S. S. N., McKenzie, E. H. C., & Wen, T. C. (2021). Taxonomic studies of Coronophorales and Niessliaceae (Hypocreomycetidae). Mycosphere, 12(1), 875-992.

Inderbitzin, P., Mehta, Y. R., & Berbee, M. L. (2009). Pleospora species with Stemphylium anamorphs a four locus phylogeny resolves new lineages yet does not distinguish among species in the Pleospora herbarum clade. Mycologia, 101(3), 329-339.

Kang, J. C., Hyde, K. D., & Kong, R. Y. (1999). Studies on Amphisphaeriales: the Amphisphaeriaceae (sensu stricto). Mycological Research, 103(1), 53-64.

Kim, J. H. (2015). New records of the genus Spirogyra (Zygnemataceae, Conjugatophyceae) in Korea. Journal of Ecology and Environment, 38(4), 611-618.

Ko, Y. Z., Liyanage, W. K., Shih, H. C., Tseng, M. N., Shiao, M. S., & Chiang, Y. C. (2023). Unveiling cryptic species diversity and genetic variation of Lasiodiplodia (Botryosphaeriaceae, Botryosphaeriales) infecting fruit crops in Taiwan. Journal of Fungi, 9(9), 950.

Korsa, G., Alemu, D., & Ayele, A. (2024). Azolla plant production and their potential applications. International Journal of Agronomy, 2024(1), 1716440.

Kristiansen, J., Škaloud, P. (2017). Chrysophyta. In: Archibald, J., Simpson, A., Slamovits, C. (eds) Handbook of the Protists. Springer, Cham. https://doi.org/10.1007/978-3-319-28149-0_43

Kück, U., Pöggeler, S., Nowrousian, M., Nolting, N., Engh, I. (2009). Sordaria macrospora, a Model System for Fungal Development. In Anke, T., Weber, D. (eds) Physiology and Genetics. The Mycota, vol 15. Springer, Berlin, Heidelberg. httpsdoi.org10.1007978-3-642-00286-1_2

Lee, O. M., McCourt, R. M., Nam, M., & Karol, K. G. (2000). Species Phylogeny of Cosmarium and Staurastrum (desmidiaceae) Based on Rbc L Sequences. Journal of Phycology, 36, 42-43.

Li, J., Phookamsak, R., Jiang, H., Bhat, D. J., Camporesi, E., Lumyong, S., ... & Suwannarach, N. (2022). Additions to the inventory of the genus Alternaria section Alternaria (Pleosporaceae, Pleosporales) in Italy. Journal of Fungi, 8(9), 898.

Li, Z., Mertens, K. N., Gottschling, M., Gu, H., Söhner, S., Price, A. M., ... & Shin, H. H. (2020). Taxonomy and molecular phylogenetics of Ensiculiferaceae, fam. nov.(Peridiniales, Dinophyceae), with consideration of their life-history. Protist, 171(5), 125759.

Liao, C. F., Wei, D., Chethana, K. W., Pem, D., Phookamsak, R., Suwannarach, N., & Doilom, M. (2022). Morphological and phylogenetic evidence reveal Tetraploa cylindrica sp. nov.(Tetraplosphaeriaceae, Pleosporales) from Saccharum arundinaceum (Poaceae) in Yunnan Province, China.

Liu, B., Hu, Y., Hu, Z., Liu, G., & Zhu, H. (2020). Taxonomic scheme of the order Chaetophorales (Chlorophyceae, Chlorophyta) based on chloroplast genomes. BMC genomics, 21(1), 442.

Luo, J., & Zhang, N. (2013). Magnaporthiopsis, a new genus in Magnaporthaceae (Ascomycota). Mycologia, 105(4), 1019-1029.

Marin-Felix, Y., Miller, A. N., Cano-Lira, J. F., Guarro, J., García, D., Stadler, M., ... & Stchigel, A. M. (2020). Re-evaluation of the order Sordariales: Delimitation of Lasiosphaeriaceae s. str., and introduction of the new families Diplogelasinosporaceae, Naviculisporaceae, and Schizotheciaceae. Microorganisms, 8(9), 1430.

Marin-Felix, Y., Miller, A. N., Cano-Lira, J. F., Guarro, J., García, D., Stadler, M., ... & Stchigel, A. M. (2020). Re-evaluation of the order Sordariales: Delimitation of Lasiosphaeriaceae s. str., and introduction of the new families Diplogelasinosporaceae, Naviculisporaceae, and Schizotheciaceae. Microorganisms, 8(9), 1430.

McCourt, R. M., Karol, K. G., Guerlesquin, M., & Feist, M. (1996). Phylogeny of extant genera in the family Characeae (Charales, Charophyceae) based on rbcL, sequences and morphology. American journal of botany, 83(1), 125-131.

Mertens, K. N., Gu, H., Takano, Y., Price, A. M., Pospelova, V., Bogus, K., ... & Matsuoka, K. (2017). The cyst-theca relation of Trinovantedinium pallidifulvum, with erection of Protoperidinium lousianensis sp. nov. and their phylogenetic position within the Conica group. Palynology, 41(2), 183-202.

Mohite, B.V., Koli, S.H., Salunkhe, J.D., Patil, S.V. (2025). Ustilago. In Amaresan, N., Kumar, K. (eds) Compendium of Phytopathogenic Microbes in Agro-Ecology . Springer, Cham. httpsdoi.org10.1007978-3-031-81770-0_38

Mudie, P. J., Fensome, R. A., Rochon, A., & Bakrač, K. (2020). The dinoflagellate cysts Thalassiphora subreticulata n. sp. and Thalassiphora balcanica: their taxonomy, ontogenetic variation and evolution. Palynology, 44(2), 237-269.

Niechwedowicz, M. (2022). Dinoflagellate cysts from the Upper Cretaceous (upper Campanian to lowermost Maastrichtian) of the Middle Vistula River section, Poland. Palynology, 46(1), 1-37.

Pintye, A., & Knapp, D. G. (2021). Two pleosporalean root-colonizing fungi, Fuscosphaeria hungarica gen. et sp. nov. and Delitschia chaetomioides, from a semiarid grassland in Hungary. Mycological Progress, 20(1), 39-50.

Radi, T., Bonnet, S., Cormier, M. A., de Vernal, A., Durantou, L., Faubert, É., ... & Van Nieuwenhove, N. (2013). Operational taxonomy and (paleo-) autecology of round, brown, spiny dinoflagellate cysts from the Quaternary of high northern latitudes. Marine Micropaleontology, 98, 41-57.

Rich, F. J., Kuehn, D., & Davies, T. D. (1982). The paleoecological significance of Ovoidites. Palynology, 6(1), 19-28.

Riess, K., Schön, M. E., Ziegler, R., Lutz, M., Shivas, R. G., Piątek, M., & Garnica, S. (2019). The origin and diversification of the Entorrhizales: deep evolutionary roots but recent speciation with a phylogenetic and phenotypic split between associates of the Cyperaceae and Juncaceae. Organisms Diversity & Evolution, 19(1), 13-30.

Romero, M. F., Samoluk, S. S., Seijo, J. G., & Gonzalez, A. M. (2025). Histopathology of Thecaphora frezzii Colonization A Detailed Analysis of Its Journey Through Peanut (Arachis hypogaea L.) Tissues. Plants, 14(7), 1083.

Seethapathy, P. (2025). Tilletia. In: Amaresan, N., Kumar, K. (eds) Compendium of Phytopathogenic Microbes in Agro-Ecology . Springer, Cham. https://doi.org/10.1007/978-3-031-81770-0_36

Skinner, S., & Entwisle, T. J. (2015). Mougeotia (Zygnemaceae, Streptophyta) in Australia. Telopea, 18, 481-494.

Sluijs, A., Brinkhuis, H., Crouch, E. M., John, C. M., Handley, L., Munsterman, D., ... & Dickens, G. R. (2008). Eustatic variations during the Paleocene-Eocene greenhouse world. Paleoceanography, 23(4).

Stancheva, R., Sheath, R. G., & Hall, J. D. (2012). Systematics of the genus Zygnema (Zygnematophyceae, Charophyta) from Californian watersheds. Journal of Phycology, 48(2), 409–422. httpsdoi.org10.1111j.1529-8817.2012.01135.x

Stürmer, S. L. (2012). A history of the taxonomy and systematics of arbuscular mycorrhizal fungi belonging to the phylum Glomeromycota. Mycorrhiza, 22(4), 247-258.

Van der Merwe, M., Ericson, L., Walker, J., Thrall, P. H., & Burdon, J. J. (2007). Evolutionary relationships among species of Puccinia and Uromyces (Pucciniaceae, Uredinales) inferred from partial protein coding gene phylogenies. Mycological research, 111(2), 163-175.

Wang, X. W., Han, P. J., Bai, F. Y., Luo, A., Bensch, K., Meijer, M., ... & Houbraken, J. (2022). Taxonomy, phylogeny and identification of Chaetomiaceae with emphasis on thermophilic species. Studies in mycology, 101, 121.

Wang, X. W., Han, P. J., Bai, F. Y., Luo, A., Bensch, K., Meijer, M., ... & Houbraken, J. (2022). Taxonomy, phylogeny and identification of Chaetomiaceae with emphasis on thermophilic species. Studies in mycology, 101, 121.

Whitton, B.A., Mateo, P. (2012). Rivulariaceae. In: Whitton, B. (eds) Ecology of Cyanobacteria II. Springer, Dordrecht. https://doi.org/10.1007/978-94-007-3855-3_22

Wu, H. X., Schoch, C. L., Boonmee, S., Bahkali, A. H., Chomnunti, P., & Hyde, K. D. (2011). A reappraisal of Microthyriaceae. Fungal Diversity, 51(1), 189-248.

Yang, Y., Ferguson, D. K., Liu, B., Mao, K. S., Gao, L. M., Zhang, S. Z., ... & Zhang, Z. X. (2022). Recent advances on phylogenomics of gymnosperms and a new classification. Plant diversity, 44(4), 340-350.

Zhang, N., & Blackwell, M. (2002). Molecular phylogeny of Melanospora and similar pyrenomycetous fungi. Mycological Research, 106(2), 148-155.

3. References: taxonomic classification by subfamily and tribe

Ajao, A. A. N., Akinlabi, F. M., Stewart, R. D., Oladipo, O. T., & Moteetee, A. N. (2024). Wood anatomical diversity and distribution modelling of Pterocarpus Jacq.(Fabaceae: Dalbergieae): Ecological and systematical implications. Taiwania, 69(4).

Akah, P. A., Nworu, C. S., Mbaoji, F. N., Nwabunike, I. A., & Onyeto, C. A. (2012). Genus Detarium Ethnomedicinal, phytochemical and pharmacological profile. Phytopharmacology, 3(2), 367-375.

Amirkhosravi, A., Asri, Y., Assadi, M., & Mehregan, I. (2021). Genetic structure of Alhagi (Hedysareae, Fabaceae) populations using ISSR data in Iran. Molecular Biology Reports, 48(6), 5143-5150.

Aragão, F.J.L., Brondani, R.P.V., Burle, M.L. (2011). Phaseolus. In Kole, C. (eds) Wild Crop Relatives Genomic and Breeding Resources. Springer, Berlin, Heidelberg. httpsdoi.org10.1007978-3-642-14387-8_11

Arroyo, J., Aparicio, A., Albaladejo, R. G., MuNOz, J., & Braza, R. (2008). Genetic structure and population differentiation of the Mediterranean pioneer spiny broom Calicotome villosa across the Strait of Gibraltar. Biological Journal of the Linnean Society, 93(1), 39-51.

Banks, H., & Gasson, P. (2000). Pollen morphology and wood anatomy of the Crudia group (Leguminosae, Caesalpinioideae, Detarieae). Botanical Journal of the Linnean Society, 134(1-2), 19-59.

Bansal, M., Mishra, S., & Prasad, V. (2022). Biogeographic and evolutionary history of Crotonoideae based on pollen evidence from Indian Late Cretaceous and Paleogene sediments. Biotropica, 54(6), 1331-1348.

Bellot, S., Dias, P. M., Affagard, M., Aïnouche, M. L., Misset, M. T., & Aïnouche, A. (2023). Molecular phylogenetics shed light on polyploid speciation in gorses (Ulex, Fabaceae: Genisteae) and on the origin of the invasive Ulex europaeus. Botanical Journal of the Linnean Society, 202(1), 52-75.

Benor, S. (2018). Molecular phylogeny of the genus Corchorus (Grewioideae, Malvaceae sl) based on nuclear rDNA ITS sequences. The Crop Journal, 6(5), 552-563.

Boatwright, J. S., Savolainen, V., Van Wyk, B. E., Lise Schutte-Vlok, A., Forest, F., & Van Der Bank, M. (2008). Systematic position of the anomalous genus Cadia and the phylogeny of the tribe Podalyrieae (Fabaceae). Systematic Botany, 33(1), 133-147.

Boom, A. F., Migliore, J., Kaymak, E., Meerts, P., & Hardy, O. J. (2022). Nuclear ribosomal phylogeny of Brachystegia. Plant Ecology and Evolution, 155(2), 301-314.

Bremer, B., Andreasen, K., & Olsson, D. (1995). Subfamilial and tribal relationships in the Rubiaceae based on rbcL sequence data. Annals of the Missouri Botanical Garden, 383-397.

Brottier, L., Chaintreuil, C., Simion, P., Scornavacca, C., Rivallan, R., Mournet, P., ... & Arrighi, J. F. (2018). A phylogenetic framework of the legume genus Aeschynomene for comparative genetic analysis of the Nod-dependent and Nod-independent symbioses. BMC Plant Biology, 18(1), 333.

Brullo, S., Brullo, C., Cambria, S., Acar, Z., Salmeri, C., & Del Galdo, G. G. (2018). Taxonomic and Phylogenetic Investigations on Psoralea acaulis (Psoraleeae Fabaceae) with the Description of a New Genus Kartalinia1. Annals of the Missouri Botanical Garden, 103(4), 604-627.

Bruneau, A., de Queiroz, L. P., Ringelberg, J. J., Borges, L. M., da Costa Bortoluzzi, R. L., Brown, G. K., ... & Terra, V. (2024). Advances in Legume Systematics 14. Classification of Caesalpinioideae. Part 2: Higher-level classification. PhytoKeys, 240, 1.

Castillon, E. E., Quintanilla, J. A. V., Delgado-Salinas, A., & Rebman, J. P. (2023). The genus Astragalus (Leguminosae: Papilionoideae: Galegeae) in Mexico. Phytotaxa, 586(1), 1-162.

Chan, E. W. C., Yeong, S. W., Wong, C. W., Soo, O. Y. M., Phua, A. C. Y., & Ng, Y. K. (2022). Ceiba pentandra (L.) Gaertn.: An overview of its botany, uses, reproductive biology, pharmacological properties, and industrial potentials. Journal of Applied Biology and Biotechnology, 10(20), 1-7.

Christenhusz, M. J., Pannell, J. R., Twyford, A. D., of Life, W. S. I. T., & Darwin Tree of Life Consortium. (2024). The genome sequence of the annual mercury, Mercurialis annua L., 1753 (Euphorbiaceae). Wellcome Open Research, 9, 102.

Colli-Silva, M., Pérez-Escobar, O. A., Ferreira, C. D., Costa, M. T., Gerace, S., Coutinho, T. S., ... & Antonelli, A. (2025). Taxonomy in the light of incongruence: An updated classification of Malvales and Malvaceae based on phylogenomic data. Taxon, 74(2), 361-385.

Conserva, L. M., & Jesu Costa Ferreira, J. (2012). Borreria and Spermacoce species (Rubiaceae): A review of their ethnomedicinal properties, chemical constituents, and biological activities. Pharmacognosy reviews, 6(11), 46.

Coutinho, T. S., Barbosa-Silva, R. G., & Dorr, L. J. (2025). A synopsis of Christiana DC.(Malvaceae, Brownlowioideae), with a new species from the Brazilian Atlantic Forest. PhytoKeys, 253, 33.

Dalavi, J. V., Bramhadande, S. P., Mane, R. N., Mane, P. R., & SR, Y. (2020). Two new varieties of Alysicarpus (Fabaceae) from Peninsular India. Journal of the Indian Association for Angiosperm Taxonomy, 30(2), 270-277.

Das, G., Shin, H. S., Ningthoujam, S. S., Talukdar, A. D., Upadhyaya, H., Tundis, R., ... & Patra, J. K. (2021). Systematics, phytochemistry, biological activities and health promoting effects of the plants from the subfamily bombacoideae (family Malvaceae). Plants, 10(4), 651.

de Block, P., & Robbrecht, E. (1998). Pollen morphology of the Pavetteae (Rubiaceae, Ixoroideae) and its taxonomic significance. Grana, 37(5), 260-275.

De La Estrella, M., Aedo, C., & Velayos, M. (2009). A morphometric analysis of Daniellia (Fabaceae–Caesalpinioideae). Botanical Journal of the Linnean Society, 159(2), 268-279.

de la Estrella, M., Wieringa, J. J., Mackinder, B., van der Burgt, X., Devesa, J. A., & Bruneau, A. (2014). Phylogenetic analysis of the African genus Gilbertiodendron J. Léonard and related genera (Leguminosae-Caesalpinioideae-Detarieae). International Journal of Plant Sciences, 175(9), 975-985.

de Souza, L. R., Carneiro-Torres, D. S., Saba, M. D., & dos Santos, F. D. A. R. (2017). Pollen morphology of the Acalyphoideae and Euphorbioideae (Euphorbiaceae) of the Caatinga ecoregion in Brazil. Plant Systematics and Evolution, 303(9), 1161-1180.

Degtjareva, G. V., Valiejo-Roman, C. M., Kramina, T. E., Mironov, E. M., Samigullin, T. H., & Sokoloff, D. D. (2003). Taxonomic and phylogenetic relationships between Old World and New World members of the tribe Loteae (Leguminosae) new insights from molecular and morphological data, with special emphasis on Ornithopus. Wulfenia, 10, 15-50.

Degtjareva, G. V., Valiejo-Roman, C. M., Samigullin, T. H., Guara-Requena, M., & Sokoloff, D. D. (2012). Phylogenetics of Anthyllis (Leguminosae: Papilionoideae: Loteae): Partial incongruence between nuclear and plastid markers, a long branch problem and implications for morphological evolution. Molecular Phylogenetics and Evolution, 62(2), 693-707.

Donkpegan, A. S., Doucet, J. L., Migliore, J., Duminil, J., Dainou, K., Pineiro, R., ... & Hardy, O. J. (2017). Evolution in African tropical trees displaying ploidy-habitat association: the genus Afzelia (Leguminosae). Molecular Phylogenetics and Evolution, 107, 270-281.

Du Preez, B., Schrire, B. D., Dreyer, L. L., Stirton, C. H., Chimphango, S. B., & Muasya, A. M. (2025). Global biogeographic patterns of the genus Indigofera (Fabaceae: Indigofereae). Brazilian Journal of Botany, 48(1), 19.

Ekalu, A., & Habila, J. D. (2020). Phytochemistry, pharmacology and medicinal uses of Cola (Malvaceae) family a review. Medicinal Chemistry Research, 29(12), 2089-2105.

Elliott, A., Hyam, R., Watson, M., Wrankmore, E., Hartley, H., Krieger, J., ... & Rankin, D. (2024). World Flora Online Plant List December 2024. World Flora Online Plant List December 2024.

Ellis, T. N., Smýkal, P., Maxted, N., Coyne, C. J., Domoney, C., Burstin, J., ... & Chayut, N. (2024). The taxonomic status of genera within the Fabeae (Vicieae), with a special focus on Pisum. Diversity, 16(7), 365.

Farruggia, F. T. (2009). Phylogenetic and monographic studies of the pantropical genus Sesbania Adanson (Leguminosae). Arizona State University.

Feng, J., Xiong, Y., Su, X., Liu, T., Xiong, Y., Zhao, J., ... & Ma, X. (2023). Analysis of complete chloroplast genome Structure, phylogenetic relationships of Galega orientalis and evolutionary inference of Galegeae. Genes, 14(1), 176.

Fensome, R. A., & Williams, G. L. (2017). The Lentin and Williams Index of Fossil Dinoflagellates (Contribution Series 42, 909 pp.). Dallas, TX: American Association of Stratigraphic Palynologists Foundation.

Fortuna-Perez, A. P., de Moraes Castro, M., & Goulart de Azevedo Tozzi, A. M. (2012). Leaflet secretory structures of five taxa of the genus Zornia JF Gmel.(Leguminosae, Papilionoideae, Dalbergieae) and their systematic significance. Plant systematics and evolution, 298(8), 1415-1424.

Galloni, M., Podda, L., Vivarelli, D., & Cristofolini, G. (2007). Pollen presentation, pollen-ovule ratios, and other reproductive traits in Mediterranean Legumes (Fam. Fabaceae-Subfam. Faboideae). Plant Systematics and Evolution, 266(3), 147-164.

Gama, T. D. S. S., Cordeiro, I., & Demarco, D. (2019). Floral structure and development in Alchornea sidifolia (Acalyphoideae) and the evolution of wind pollination in Euphorbiaceae. Brazilian Journal of Botany, 42(2), 307-317.

Garg, A., & Tripathi, S. (2025). Pollen morphometric analogue in Adansonia digitata L. from India implications for taxonomy, systematics and evolution. Grana, 1-23.

Gargiulo, R., Guacchio, E. D., & Caputo, P. (2015). Phylogenetic reconstruction of Asperula sect. Cynanchicae (Rubiaceae) reveals a mosaic of evolutionary histories. Taxon, 64(4), 754-769.

Gbadamosi, S. O., Esther Aluko, O., & Victor, I. A. (2020). Heating and biochemical processing of Kariya (Hildegardia Bateri) seeds: chemical composition, antinutrients and functional properties. Integr food. Nutr Metab, 7, 1-9.

Ghanavati, F., & Amirabadizadeh, H. (2012). Pollen grain morphology in Iranian Hedysareae (Fabaceae). Crop Breeding Journal, 2(1), 25-33.

Goncharov, M. Y., Povydysh, M. N., & Yakovlev, G. P. (2011). Taxonomic revision of the genus Baphia (Baphieae, Fabaceae).

Head, M. J. (1996). Modern dinoflagellate cysts and their biological affinities. Palynology: principles and applications, 3, 1197-1248.

Hiroyoshi, O., & Kazuaki, O. (2019). Desmodium (Leguminosae tribe Desmodieae) of africa, madagascar and the mascarene islands. Journal of Japanese Botany, 94(3), 135-148.

Hu, G., Cheng, L., Huang, W., Cao, Q., Zhou, L., Jia, W., & Lan, Y. (2020). Chloroplast genomes of seven species of Coryloideae (Betulaceae): structures and comparative analysis. Genome, 63(7), 337-348.

Hu, J. M., Lavin, M., Wojciechowski, M. F., & Sanderson, M. J. (2000). Phylogenetic systematics of the tribe Millettieae (Leguminosae) based on chloroplast trnKmatK sequences and its implications for evolutionary patterns in Papilionoideae. American Journal of Botany, 87(3), 418-430.

Hunter, S., Cardoso, D., Ruhlman, T. A., & Jansen, R. K. (2025). Phylogenomic analyses unravel the tangled evolutionary history of Genisteae (Fabaceae). Molecular Phylogenetics and Evolution, 204, 108249.

Jamil, I., Kousar, S., & Abid, R. (2025). Chloroplast DNA region based phylogenetic relationships within genus Abutilon Mill. Pak. J. Bot, 57(4), 1529-1535.

Jangid, P. P., & Gupta, S. (2016). Systematic wood anatomy of the subfamily Acalyphoideae sl (Euphorbiaceae) from India, with remarks on synonymy. Nordic Journal of Botany, 34(2), 197-216.

Javaid, N., Ramzan, M., Jabeen, S., Du, Y., Anwar, M., & Xiqiang, S. (2024). The chloroplast genome of Chrozophora sabulosa Kar. & Kir. and its exploration in the evolutionary position uncertainty of genus Chrozophora. BMC genomics, 25(1), 597.

Jourdain-Fievet, L., Dubuisson, J. Y., Applequist, W., Skema, C., & Le Pechon, T. (2024). Updated nomenclature and re-establishment of the genus Dombeya Cav. section Dombeya (dombeyoideae, Malvaceae). Botany Letters, 171(3), 348-356.

Kadiri, A. B., & Adeniran, S. (2016). Study of anatomy of the genus Hura L.(Euphorbiaceae). Ife Journal of Science, 18(2), 413-426.

Kainulainen, K. (2021). A taxonomic revision of Melanoxerus (Rubiaceae), with descriptions of three new species of trees from Madagascar. Candollea, 76(1), 105-116.

Kårehed, J., & Bremer, B. (2007). The systematics of Knoxieae (Rubiaceae)—molecular data and their taxonomic consequences. Taxon, 56(4), 1051-1076.

Ki-Ryong, P. A. R. K. (2019). Pollen morphology and character evolution in the subtribe Neoguillauminiinae (Euphorbiaceae). Korean Journal of Plant Taxonomy, 49(2), 101-106.

Kouamé, T., Okpekon, T., Bony, N. F., Ferron, S., Bonnaffé, D., Vanheuverzwijn, J., ... & Le Pogam, P. (2021). Anticipate, Target and Characterize MS^2-anticipated C-glycosylflavones from Erythrococca anomala. Planta Medica International Open, 8(03), e131-e142.

Krapovickas, A., Gregory, W. C., Williams, D. E., & Simpson, C. E. (2007). Taxonomy of the genus Arachis (Leguminosae). Bonplandia, 16, 7-205.

Kumar, V., Uthappa, A. R., Srivastava, M., Vijay, D., Kumaranag, K. M., Manjunatha, N., ... & Chaturvedi, O. P. (2017). Floral biology of Grewia flavescens Juss. an underutilized crop. Genetic Resources and Crop Evolution, 64(7), 1789-1795.

Lavin, M., Pennington, R. T., Klitgaard, B. B., Sprent, J. I., de Lima, H. C., & Gasson, P. E. (2001). The dalbergioid legumes (Fabaceae): delimitation of a pantropical monophyletic clade. American Journal of Botany, 88(3), 503-533.

Malcomber, S. T., & Taylor, C. M. (2009). A Systematic Revision of Gaertnera (Rubiaceae, Gaertnereae) 1. Annals of the Missouri Botanical Garden, 96(4), 575-671.

Marzouk, R. (2018). Taxonomic consequences of seed morphology and anatomy in three Lupinus species (Fabaceae-Genisteae). Catrina: The International Journal of Environmental Sciences, 1(2), 1-8.

McCourt, R. M., Karol, K. G., Guerlesquin, M., & Feist, M. (1996). Phylogeny of extant genera in the family Characeae (Charales, Charophyceae) based on rbcL, sequences and morphology. American journal of botany, 83(1), 125-131.

Mehmood, F., Shahzadi, I., Waseem, S., Mirza, B., Ahmed, I., & Waheed, M. T. (2020). Chloroplast genome of Hibiscus rosa-sinensis (Malvaceae) comparative analyses and identification of mutational hotspots. Genomics, 112(1), 581-591.

Mendes, J. C. R., Rosário, A. S. D., Bigio, N. C., Pereira-Silva, R. A., & Secco, R. D. S. (2021). Dalechampia L.(Euphorbiaceae) in the Brazilian Amazon. Biota Neotropica, 21, e20201010.

Montero-Muñoz, I., Levin, G. A., Lorenzo, C. V., González, L., & Cardiel, J. M. (2023). Novelties in the genus Acalypha (Euphorbiaceae, Acalyphoideae). Plant Ecology and Evolution, 156(3), 365-373.

Mudie, P. J., Fensome, R. A., Rochon, A., & Bakrač, K. (2020). The dinoflagellate cysts Thalassiphora subreticulata n. sp. and Thalassiphora balcanica: their taxonomy, ontogenetic variation and evolution. Palynology, 44(2), 237-269.

Neill, D. A. (1988). Experimental studies on species relationships in Erythrina (Leguminosae Papilionoideae). Annals of the Missouri Botanical Garden, 886-969.

Nguyen, N. N., & Do, H. D. K. (2025). Characterization of structural variation in the complete chloroplast genomes of Croton L.(Crotonoideae, Euphorbiaceae). Genetic Resources and Crop Evolution, 1-14.

Niechwedowicz, M. (2022). Dinoflagellate cysts from the Upper Cretaceous (upper Campanian to lowermost Maastrichtian) of the Middle Vistula River section, Poland. Palynology, 46(1), 1-37.

Nyadoi, P., Okori, P. J. B. L., Okullo, J. B. L., Obua, J., Burg, K., Nasoro, M., & Jamnadass, R. (2009). Tamarinds'(Tamarindus indica L.) niche tree species diversity characterisation reveals conservation needs and strategies. Int. J. Biodivers. Conserv, 1(4), 151-176.

Ojeda, F. S., Hoc, P. S., & Amela García, M. T. (2013). Morphology of seeds and seedlings of four species of Vigna Savi (Leguminosae, Phaseolinae). Acta Botanica Brasilica, 27, 483-489.

Patro, S. K., Sasmal, D., Mazumndar, P., Behera, P., Lal, U. R., Dash, S. K., & Padhy, R. K. (2014). Review on genus Canthium Special reference to Canthium coromandelicum-an unexplored traditional medicinal plant of Indian Subcontinent. American J Phytomed Clin Therap, 2, 796-813.

Pennington, R. T., Klitgaard, B. B., Ireland, H. E. L. E. N., & Lavin, M. A. T. T. (2000). New insights into floral evolution of basal Papilionoideae from molecular phylogenies. Advances in legume systematics, part, 9, 233-248.

Radi, T., Bonnet, S., Cormier, M. A., de Vernal, A., Durantou, L., Faubert, É., ... & Van Nieuwenhove, N. (2013). Operational taxonomy and (paleo-) autecology of round, brown, spiny dinoflagellate cysts from the Quaternary of high northern latitudes. Marine Micropaleontology, 98, 41-57.

Razafimandimbison, S. G., & Rydin, C. (2024). Phylogeny and classification of the coffee family (Rubiaceae, Gentianales): Overview and outlook. Taxon, 73(3), 673-717.

Razafimandimbison, S. G., Kainulainen, K., Wong, K. M., Beaver, K., & Bremer, B. (2011). Molecular support for a basal grade of morphologically distinct, monotypic genera in the species-rich Vanguerieae alliance (Rubiaceae, Ixoroideae): Its systematic and conservation implications. Taxon, 60(4), 941-952.

Rogers, G. K. (2005). The genera of Rubiaceae in the southeastern United States, Part II. Subfamily Rubioideae, and subfamily Cinchonoideae revisited (Chiococca, Erithalis, and Guettarda) 1. Harvard Papers in Botany, 10(1), 1-45.

Romero, I. C., & Punyasena, S. W. (2024). Niche conservatism in the Legume Amherstieae tribe Insights from the tropical Berlinia and Brownea clades. bioRxiv, 2024-09.

Santos, M. D. O., Lima, L. C. L. E., Sales, M. F. D., & Silva, J. S. (2019). Pollen morphology of the Brazilian species of Bernardia Houst. ex Mill. and Tragia L.(Euphorbiaceae, Acalyphoideae). Acta Botanica Brasilica, 33(3), 474-485.

Seixas, D. P., Fortuna-Perez, A. P., & Rodrigues, T. M. (2019). Leaf anatomical features of the Eriosema campestre Benth.(Leguminosae, Papilionoideae, Phaseoleae) complex and potential taxonomic implications. Flora, 253, 107-115.

Shukla, A., Mehrotra, R. C., Verma, P., Chandra, K., & Singh, A. (2021). "Out-of-India" dispersal for Adina (tribe Naucleeae; family Rubiaceae): evidence from the early Eocene fossil record from India. Palaeoworld, 30(4), 737-745.

Sinou, C., Forest, F., Lewis, G. P., & Bruneau, A. (2009). The genus Bauhinia sl (Leguminosae) a phylogeny based on the plastid trn L–trn F region. Botany, 87(10), 947-960.

Soza, V. L., & Olmstead, R. G. (2010). Molecular systematics of tribe Rubieae (Rubiaceae): Evolution of major clades, development of leaf–like whorls, and biogeography. Taxon, 59(3), 755-771.

Stępkowski, T., Banasiewicz, J., Granada, C. E., Andrews, M., & Passaglia, L. M. (2018). Phylogeny and phylogeography of rhizobial symbionts nodulating legumes of the tribe Genisteae. Genes, 9(3), 163.

Stirton, C. H., Bello, A., & Muasya, A. M. (2024). Ten new species and notes on the genus Psoralea L.(Psoraleeae, Fabaceae) from South Africa. Plant Ecology and Evolution, 157(3), 291-312.

Taia, W. K. (2004). Tribe Trifolieae Evidence from seed characters. Pakistan Journal of Biological Sciences, 7(7), 1287-1302.

Thureborn, O., Razafimandimbison, S. G., Wikström, N., Khodabandeh, A., & Rydin, C. (2019). Phylogeny of Anthospermeae of the coffee family inferred using clock and nonclock models. International Journal of Plant Sciences, 180(5), 386-402.

Tucker, S. C. (2001). Floral development in Schotia and Cynometra (Leguminosae: Caesalpinioideae: Detarieae). American Journal of Botany, 88(7), 1164-1180.

Tucker, S. C. (2002). Comparative floral ontogeny in Detarieae (Leguminosae Caesalpinioideae). 1. Radially symmetrical taxa lacking organ suppression. American Journal of Botany, 89(6), 875-887.

Tucker, S. C. (2002). Comparative floral ontogeny in Detarieae (Leguminosae Caesalpinioideae). 2. Zygomorphic taxa with petal and stamen suppression. American Journal of Botany, 89(6), 888-907.

Tucker, S. C. (2002). Floral ontogeny of Cercis (Leguminosae: Caesalpinioideae: Cercideae): does it show convergence with papilionoids?. International Journal of Plant Sciences, 163(1), 75-87.

Van Welzen, P. C., Sweet, F. S. T., & Fernández-Casas, F. J. (2017). A revision of Jatropha (Euphorbiaceae) in Malesia. Blumea-Biodiversity, Evolution and Biogeography of Plants, 62(1), 58-74.

Verstraete, B., Lachenaud, O., Smets, E., Dessein, S., & Sonké, B. (2013). Taxonomy and phylogenetics of Cuviera (Rubiaceae–Vanguerieae) and reinstatement of Globulostylis with the description of three new species. Botanical Journal of the Linnean Society, 173(3), 407-441.

Viot, C. R., & Wendel, J. F. (2023). Evolution of the cotton genus, Gossypium, and its domestication in the Americas. Critical Reviews in Plant Sciences, 42(1), 1-33.

Whipple, I. G., Barkworth, M. E., & Bushman, B. S. (2007). Molecular insights into the taxonomy of Glyceria (Poaceae: Meliceae) in North America. American Journal of Botany, 94(4), 551-557.

Yang, S., Mao, L., Zheng, Z., Chen, B., & Li, J. (2020). Pollen atlas for selected subfamilies of Euphorbiaceae from Southern China: a complementary contribution to Quaternary pollen analysis. Palynology, 44(4), 659-673.

Yang, Y., Ferguson, D. K., Liu, B., Mao, K. S., Gao, L. M., Zhang, S. Z., ... & Zhang, Z. X. (2022). Recent advances on phylogenomics of gymnosperms and a new classification. Plant diversity, 44(4), 340-350.

Yaradua, S. S. (2018). A review of the genus Crotalaria L.(Crotalarieae, Fabaceae). International Journal of Scientific and Research Publications, 8(6), 316321.

Zhou, Z., Gu, B. J., Sun, H., Zhu, H., & Tan, Y. H. (2017). Molecular phylogenetic analyses of Euphorbiaceae tribe Epiprineae, with the description of a new genus, Tsaiodendron gen. nov., from south-western China. Botanical Journal of the Linnean Society, 184(2), 167-184.

Zimmerman, E., Prenner, G., & Bruneau, A. (2013). Floral ontogeny in Dialiinae (Caesalpinioideae Cassieae), a study in organ loss and instability. South African Journal of Botany, 89, 188-209.

4. References: harmonisation of pollen taxonomy

African Pollen Database Image Bank

Al-Eisawi, D., & Al-Khatib, M. (2015). Palynological properties of the genus Haplophyllum (Rutaceae) in Jordan. International Journal of Current Microbiology and Applied Sciences, 4(9), 281-287.

Amirabadizadeh, H., & Ghanavati, F. (2012). Pollen grain morphology in Iranian Hedysareae (Fabaceae). Crop Breeding Journal, 2(1), 25-33.

Anikster, Y., Eilam, T., Bushnell, W. R., & Kosman, E. (2005). Spore dimensions of Puccinia species of cereal hosts as determined by image analysis. Mycologia, 97(2), 474-484.

Anisaa, A. H. S., Maideena, H., & Latiffa, A. (2013). Palynological study of the genera Ruellia, Ecbolium, Asystasia, Blepharis and Dicliptera (Acanthaceae) of Yemen. In AIP Conference Proceedings (Vol. 1571, p. 389).

Argue, C. L. (1980). Pollen morphology in the genus Mimulus (Scrophulariaceae) and its taxonomic significance. American Journal of Botany, 67(1), 68-87.

Argue, C. L. (1993). Pollen morphology in the Selagineae, Manuleae (Scrophulariaceae), and selected Globulariaceae, and its taxonomic significance. American Journal of Botany, 80(6), 723-733.

Assis, A. C. R. D., Gasparino, E. C., & Saba, M. D. (2021). Pollen morphology of selected species of Anacardiaceae and its taxonomic significance. Rodriguésia, 72, e01422020.

Association des Palynologues de Langue Française. (1974). Pollen et spores d'Afrique tropicale. CNRS, Talence. Travaux et Documents de Géographie Tropicale (No. 16).

Bagheri, A., Akhavan Roofigar, A., Abbasi, S., Maassoumi, A. A., Rutten, T., & Blattner, F. R. (2019). Pollen morphology of Astragalus section Hymenostegis (Fabaceae) and evaluation of its systematic implications. Grana, 58(5), 328-336.

Banks, H. A. N. N. A. H., & Klitgaard, B. B. (2000). Palynological contribution to the systematics of detarioid legumes (Leguminosae: Caesalpinioideae). Advances in legume systematics, 9, 79-106.

Banks, H., & Gasson, P. (2000). Pollen morphology and wood anatomy of the Crudia group (Leguminosae, Caesalpinioideae, Detarieae). Botanical Journal of the Linnean Society, 134(1-2), 19-59.

Banks, H., & Klitgaard, B. B. (2000). Palynological contribution to the systematics of detarioid legumes (Leguminosae: Caesalpinioideae). Advances in legume systematics, 9, 79-106.

Banks, H., & Lewis, G. (2009). Pollen morphology of the Dimorphandra group (Leguminosae, Caesalpinioideae). Grana, 48(1), 19-26.

Banks, H., & Lewis, G. (2018). Phylogenetically informative pollen structures of 'caesalpinioid'pollen (Caesalpinioideae, Cercidoideae, Detarioideae, Dialioideae and Duparquetioideae: Fabaceae). Botanical Journal of the Linnean Society, 187(1), 59-86.

Banks, H., Forest, F., & Lewis, G. (2013). Palynological contribution to the systematics and taxonomy of Bauhinia sl (Leguminosae: Cercideae). South African Journal of Botany, 89, 219-226.

Basak RK (1967) Studies on the pollen morphology of Simaroubaceae. Bull Bot Surv India 9:63–67

Beaumont, A. J., Beckett, R. P., Edwards, T. J., & Stiron, C. H. (1999). Revision of the genus Calpurnia (Sophoreae: leguminosae). Bothalia, 29(1), 5-23.

Behnke, H. D. (1977). Pollen-exine sculpturing in three Centrospermae (Gisekia, Limeum, Hectorella), in Gyrostemonaceae and Rhabdodendraceae. Plant Systematics and Evolution, 128, 227-235.

Bell, B. A., Bishop, T. H., Fletcher, W. J., Ryan, P., & Ilmen, R. (2018). Cedrus atlantica pollen morphology and investigation of grain size variability using laser diffraction granulometry. Palynology, 42(3), 339-353.

Belling, A. J., & Heusser, C. J. (1974). Spore Morphology of the Polypodiaceae of Northeastern North America. I. Bulletin of the Torrey Botanical Club, 326-339.

Beraldi-Campesi, H., Cevallos-Ferriz, S. R., & Chacón-Baca, E. (2004). Microfossil algae associated with Cretaceous stromatolites in the Tarahumara Formation, Sonora, Mexico. Cretaceous Research, 25(2), 249-265.

Beretta, M., Rodondi, G., Adamec, L., & Andreis, C. (2014). Pollen morphology of european bladderworts (Utricularia L., Lentibulariaceae). Review of Palaeobotany and Palynology, 205, 22-30.

Bezuidenhout, A. (1964). The pollen of the African Podostemataceae. Pollen et Spores, 6(2), 463-478.

Blackmore, S. (1984). Compositae-lactuceae. Review of Palaeobotany and Palynology, 42(1-4), 45-85.

Blackmore, S., & Heath, G. L. A. (1984). Menyanthaceae. Review of palaeobotany and palynology, 42(1-4), 121-132.

Bogle, A. L., & Philbrick, C. T. (1980). A generic atlas of hamamelidaceous pollens. Contributions from the Gray Herbarium of Harvard University, (210), 29-103.

Bonnefille, R. (1971). Atlas des pollens d'Ethiopie. Pollens actuels de la basse vallée de l'Omo, récoltes botaniques 1968. Adansonia (2) 11/3, 463-518.

Bonnefille, R. (1971). Atlas des pollens d'Éthiopie. Principales espèces des forêts de montagne. Pollen et Spores, 13(1), 15-72.

Bonnefille, R., & Riollet, G. (1980). Pollens des savanes d'Afrique orientale (p. 140). Paris: Éditions du Centre National de la Recherche Scientifique.

Bonnefille, R., Lobreau, D., & Riollet, G. (1982). Fossil pollen of Ximenia (Olacaceae) in the Lower Pleistocene of Olduvai, Tanzania: Palaeoecological implications. Journal of Biogeography, 469-486.

Boonmee, S., D'souza, M.J., Luo, Z. et al. Dictyosporiaceae fam. nov.. Fungal Diversity 80, 457–482 (2016). https://doi.org/10.1007/s13225-016-0363-z

Borsch, T. (1998). Pollen types in the Amaranthaceae. Morphology and evolutionary significance. Grana, 37(3), 129-142.

Bortenschlager, S. (1990). Aspects of pollen morphology in the Cupressaceae. Grana, 29(2), 129-138.

Bos, J. A., & Punt, W. (1991). Juglandaceae. Review of palaeobotany and palynology, 69(1-3), 79-95.

Bouchal, J. M., Geier, C., Ulrich, S., Wilde, V., Lenz, O. K., Zetter, R., & Grímsson, F. (2024). Qualitative LM and SEM study of the Messel palynoflora: part I. Algae to Vitales. Grana, 63(3), 193-246.

Bremekamp, C. E. B. (1942). The position of the genus Thomandersia Baill. Recueil des travaux botaniques néerlandais, 39(1), 166-175.

Burgaz Moreno, M.E. (1989). Palinología de las Papaveraceae valencianas. Tesis de Licenciatura. Universidad de Valencia.

Burgess, T. I., Barber, P. A., Mohali, S., Pegg, G., de Beer, W., & Wingfield, M. J. (2006). Three new Lasiodiplodia spp. from the tropics, recognized based on DNA sequence comparisons and morphology. Mycologia, 98(3), 423-435.

Cai, L., Jeewon, R., & Hyde, K. D. (2005). Phylogenetic evaluation and taxonomic revision of Schizothecium based on ribosomal DNA and protein coding genes. Fungal Diversity.

Cai, L., Jeewon, R., & Hyde, K. D. (2006). Phylogenetic investigations of Sordariaceae based on multiple gene sequences and morphology. Mycological research, 110(2), 137-150.

Cain, R. F. (1956). Studies of Coprophilous Ascomygetes: IV. Tripterospora, a New Cleistocarpous Genus in a New Family. Canadian Journal of Botany, 34(4), 699-710.

Campbell, L. M. (2012). Pollen morphology of Xyridaceae (Poales) and its systematic potential. Botanical Review, 428-439.

Cao, M., Zhang, D. X., Shah, A., & Dong, L. (2014). Pollen morphology and its systematic significance in Zanthoxylum (Rutaceae) from China. Pakistan Journal of Botany, 46(4), 1325-1330.

Carrión J.S., van Geel B. (1999). Fine-resolution Upper Weichselian and Holocene palynological record from Navarrés (Valencia, Spain) and a discussion about factors of meditteranean forest succession. Rev. Palaeobot. Palynol. 106:209-236.

Carrión, J. S. (2002). A taphonomic study of modern pollen assemblages from dung and surface sediments in arid environments of Spain. Review of Palaeobotany and Palynology, 120(3-4), 217-232.

Carris, L. M., Castlebury, L. A., & Goates, B. J. (2006). Nonsystemic bunt fungi—Tilletia indica and T. horrida a review of history, systematics, and biology. Annu. Rev. Phytopathol., 44(1), 113-133.

Çelemli, Ö. G. (2022). Characterization of palynological features of Cyclamen species native to Turkey and new approaches for their systematic significance. Palynology, 46(1), 1-11.

Cerceau, M-T. (1959) Clé de détermination d'Ombellifères de France et d'Afrique du Nord d'après leurs grains de pollen. Pollen et Spores 1(2) 145-190.

Ceter, T., Ekici, M., Pinar, N. M., & Ozbek, F. (2013). Pollen morphology of Astragalus L. section Hololeuce Bunge (Fabaceae) in Turkey. Acta Botanica Gallica, 160(1), 43-52.

Chakraborty, P., Gupta-Bhattacharya, S., Roy, I., & Chanda, S. (2004). Identification of shared allergenic components from four common and dominant pollen taxa of Arecaceae. Current Science, 1539-1543.

Chanda, S., Nilsson, S., & Blackmore, S. (1988). Phylogenetic trends in the Alismatales with reference to pollen grains. Grana, 27(4), 257-272.

Chao, Y. S., & Huang, Y. M. (2018). Spore morphology and its systematic implication in Pteris (Pteridaceae). PloS One, 13(11), e0207712.

Chinnappa, C. C., & Warner, B. G. (1981). Pollen morphology in the genus Coffea (Rubiaceae) and its taxonomic significance. Botanical Journal of the Linnean Society, 83(3), 221-236.

Chmura, G. L., Stone, P. A., & Ross, M. S. (2006). Non-pollen microfossils in Everglades sediments. Review of Palaeobotany and Palynology, 141(1-2), 103-119.

Christensen, P. B., & Blackmore, S. (1988). Tiliaceae. Review of Palaeobotany and Palynology, 57(1-2), 33-43.

Clarke, G. (1978). Pollen morphology and generic relationships in the Valerianaceae. Grana, 17(2), 61-75.

Clarke, G. C. S. (1976). Guttiferae. Review of Palaeobotany and Palynology, 21(3), 125-142.

Clarke, G. C. S., & Jones, M. R. (1977). Plantaginaceae. Review of Palaeobotany and Palynology, 24(4), 129-154.

Clarke, G. C. S., & Jones, M. R. (1978). Aceraceae. Review of Palaeobotany and Palynology, 26(5), 181-193.

Clarke, G. C. S., & Jones, M. R. (1981). Dipsacaceae. Review of Palaeobotany and Palynology, 33(1), 1-25.

Clarke, G. C. S., Punt, W., & Hoen, P. P. (1991). Ranunculaceae. Review of palaeobotany and palynology, 69(1-3), 117-271.

Clò, E., & Florenzano, A. (2022). Heterocysts of Rivularia Type for Interpreting a Palaeoenvironmental Context of the Late Quaternary in Northern Italy. Sustainability, 14(22), 15332.

Coetzee, J. A., & Praglowski, J. (1984). Pollen evidence for the occurrence of Casuarina and Myrica in the Tertiary of South Africa. Grana, 23(1), 23-41.

Cooper, R. L., Osborn, J. M., & Philbrick, C. T. (2000). Comparative pollen morphology and ultrastructure of the Callitrichaceae. American Journal of Botany, 87(2), 161-175.

Copenhaver, G. P. (2005). A compendium of plant species producing pollen tetrads. Journal of the North Carolina Academy of Science, 17-35.

Dahl, A. O. (1952). The comparative morphology of the Icacinaceae, VI. The pollen. Journal of the Arnold Arboretum, 33(3), 252-295.

Dapena, M. J. D., & Ferguson, I. K. (1990). Studies of the pollen morphology and taxonomy of the tribes Loteae and Coronilleae (Leguminosae: Papilionoideae). 1. Anthyllis L. and related genera. Lagascalia, 16(1), 77-94.

Davidson, C. (1973). An anatomical and morphological study of Datiscaceae. Aliso: A Journal of Systematic and Floristic Botany, 8(1), 49-110.

Davis, R. H. (2000). Neurospora: contributions of a model organism. Oxford University Press.

de Block, P., & Robbrecht, E. (1998). Pollen morphology of the Pavetteae (Rubiaceae, Ixoroideae) and its taxonomic significance. Grana, 37(5), 260-275.

de Borges, R. L. B., dos Santos, F. D. A. R., & Giulietti, A. M. (2009). Comparative pollen morphology and taxonomic considerations in Eriocaulaceae. Review of Palaeobotany and Palynology, 154(1-4), 91-105.

De Paz, J. P. (2004). Rosaceae-Sanguisorbeae de Macaronesia: Géneros Marcetella, Bencomia y Dendriopoterium. Palinología, biogeografía, sistemas sexuales y filogenia. Bot Macaronesica, 25, 95-126.

de Silva, F. H. M., & de Assis Ribeiro dos Santos, F. (2009). Pollen morphology of the shrub and arboreal flora of mangroves of Northeastern Brazil. Wetlands Ecology and Management, 17, 423-443.

de Vernal A., Rochon A. and Radi T. (2013) Dinoflagellates. In: Elias S.A. (ed.) The Encyclopedia of Quaternary Science, vol. 2, pp. 800-815. Amsterdam: Elsevier.

Dehgan, B., & Dehgan, N. B. (1988). Comparative pollen morphology and taxonomic affinities in Cycadales. American Journal of Botany, 75(10), 1501-1516.

Desprat, S., Díaz Fernández, P. M., Coulon, T., Ezzat, L., Pessarossi-Langlois, J., Gil, L., ... & Sánchez Goñi, M. F. (2015). Pinus nigra (European black pine) as the dominant species of the last glacial pinewoods in south-western to central Iberia: a morphological study of modern and fossil pollen. Journal of Biogeography, 42(10), 1998-2009.

Dessein, S., Huysmans, S., Robbrecht, E., & Smets, E. (2002). Pollen of African Spermacoce species (Rubiaceae) morphology and evolutionary aspects. Grana, 41(2), 69-89.

Dessein, S., Scheltens, A., Huysmans, S., Robbrecht, E., & Smets, E. (2000). Pollen morphological survey of Pentas (Rubiaceae–Rubioideae) and its closest allies. Review of Palaeobotany and Palynology, 112(4), 189-205.

Devi, S. (1979). Spore types, morphological evolution and phylogeny in the Pteridaceae. Grana, 18(1), 41-46.

Dickison WC. (1979). A survey of pollen morphology of the Connaraceae. Pollen Spores 21: 31–79.

Dickison, W. C. (1987). A palynological study of the Staphyleaceae. Grana, 26(1), 11-24.

Dickison, W. C., & Sweitzer, E. M. (1970). The morphology and relationships of Barbeya oleoides. American Journal of Botany, 57(4), 468-476.

Dickison, W. C., Nowicke, J. W., & Skvarla, J. J. (1982). Pollen morphology of the Dilleniaceae and Actinidiaceae. American Journal of Botany, 69(7), 1055-1073.

Díez Dapena, M. J. (1981). Estudio palinológico de las especies españolas de la familia boraginaceae. Tesis Doctoral. Universidad de Sevilla

Díez, M. J., & Ferguson, I. K. (1996). Studies of the pollen morphology and taxonomy of the tribes Loteae and Coronilleae (Papilionoideae; Leguminoseae). 3, Coronilla L. and related genera and systematic conclusions. Review of Palaeobotany and Palynology, 94(3-4), 239-257.

Diez, M. J., Talavera, S., & Garcia-Murillo, P. (1988). Contributions to the palynology of hydrophytic, non-entomophilous angiosperms. 1. Studies with LM and SEM. Candollea, 43(1), 147-158.

Dolan, J. R. (2013). Introduction to tintinnids. The biology and ecology of tintinnid ciliates: models for marine plankton, 1, 1-16.

Dowding, E. S. (1933). Gelasinospora, a new genus of pyrenomycetes with pitted spores. Canadian Journal of Research, 9(3), 294-305.

Doyle, J. A., & Le Thomas, A. (2012). Evolution and phylogenetic significance of pollen in Annonaceae. Botanical Journal of the Linnean Society, 169(1), 190-221.

Dunbar, A. (1975). On pollen of Campanulaceae and related families with special reference to the surface ultrastructure. I. Campanulaceae Subfam. Campanuloidae. families with special reference to the surface ultrastructureII. Campanulaceae subfam. Cyphioideae and subfam. Lobelioideae; Goodeniaceae; Sphenocleaceae. Bot. Not. 128: 102–118.

Dunbar, A. (1984). Pollen morphology in Campanulaceae IV. Nordic journal of Botany, 4(1), 1-19.

Dutra, F. V., Bellonzi, T. K., de Souza, C. N., & Gasparino, E. C. (2020). Pollen morphology of Rubiaceae from Cerrado forest fragments: pollen unit, polarity and diversity of the types of apertures. Review of Palaeobotany and Palynology, 282, 104297.

E.A. Molinari Novoa in Guiry, M.D. & Guiry, G.M. (2025). AlgaeBase. World-wide electronic publication, National University of Ireland, Galway. http://www.algaebase.org; searched on 24 July 2025.

El Atfy, H., Bomfleur, B. & Kerp, H. Botryococcus: exceptionally well-preserved fossil examples of a tiny colonial green alga. PalZ 98, 391–393 (2024). https://doi.org/10.1007/s12542-024-00701-0

El Ghazali, G. E. (1993). A study on the pollen flora of Sudan. Review of palaeobotany and palynology, 76(2-4), 99-345.

El Ghazali, G. E. B. (1993). A study on the pollen flora of Sudan. Review of Palaeobotany and Palynology, 76, 99-345.

El Naggar, S. M. (2002). Taxonomic significance of pollen morphology in some taxa of Resedaceae. Feddes Repertorium: Zeitschrift für botanische Taxonomie und Geobotanik, 113(7-8), 518-527.

El Naggar, S., & El-Husseini, N. (2001). Pollen Atlas of the Flora of Egypt. 2. Species of Polygonaceae. Taeckholmia, 21(1), 143-151.

El Naggar, S., & El-Husseini, N. (2001). Pollen Atlas of the Flora of Egypt. 2. Species of Polygonaceae. Taeckholmia, 21(1), 143-151.

Elliott, A., Hyam, R., Watson, M., Wrankmore, E., Hartley, H., Krieger, J., ... & Rankin, D. (2024). World Flora Online Plant List December 2024. World Flora Online Plant List December 2024.

El-Noamani, Z. M., & Saleh, A. (2018). Cretaceous algal palynomorphs from northeast Sinai, Egypt: Systematics and paleoenvironmental implications. Egyptian Journal of Botany, 58(1), 63-72.

Engel, M. S. (1978). Haloragaceae. Review of Palaeobotany and Palynology, 26(5), 199-207.

Engel, M. S. (1978). Hippuridaceae. Review of Palaeobotany and Palynology, 26(5), 195-198.

Fensome, R. A., & Williams, G. L. (2017). The Lentin and Williams Index of Fossil Dinoflagellates (Contribution Series 42, 909 pp.). Dallas, TX: American Association of Stratigraphic Palynologists Foundation.

Ferguson, I. K. (1975). Pollen morphology of the tribe Triclisieae of the Menispermaceae in relation to its taxonomy. Kew Bulletin, 49-75.

Ferguson, I. K. (1985). The pollen morphology of Moringaceae. Kew Bulletin, 25-34.

Ferguson, I.K. (1980). The pollen morphology of Ceratonia (LeguminosaeCaesalpinioideae). Kew Bulletin 35: 273–277.

Fernández-González, D., & Lobreau-Callen, D. (1996). Le pollen de la tribu des Acalypheae (Acalyphoideae, Euphorbiaceae). Grana, 35(5), 266-284.

Fernández-Mensaque, P. C. (1976). Palinología en Caryophyllaceae de España meridional (Doctoral dissertation). Universidad de Sevilla

Ferreira de Sousa, H. C., Gonçalves-Esteves, V., Trovó, M., & Mendonça, C. B. F. (2020). Palynology of Ochnaceae from the Itatiaia National Park, Brazil. Feddes Repertorium, 131(2), 93-100.

Feuer, S. M. (1978). Aperture evolution in the genus Ptychopetalum Benth.(Olacaceae). American Journal of Botany, 65(7), 759-763.

Fineran, B. A., & Fineran, J. M. (1992). Teliospore wall structure in Entorrhiza (Tilletiaceae) and its relationship to taxonomy of the genus. Canadian journal of botany, 70(10), 1964-1983.

Firat, M., & Selvi, S. (2021). Palynological observations on the genus Gundelia L.(Asteraceae) growing in Turkey. Phytotaxa, 502(1), 51-66.

Fuchs, H. P. (1967). Pollen morphology of the family Bombacaceae. Review of Palaeobotany and Palynology, 3(1-4), 119-132.

Furness, C. A. (1990). Pollen morphology of Crossandra Salisbury and Crossandrella CB Clarke (Acanthaceae: Acantheae). Grana, 29(3), 161-176.

Furness, C. A. (1994). The pollen morphology of Hygrophila and Brillantaisia (Acanthaceae: Ruellieae). Acta botanica gallica, 141(2), 267-278.

Furness, C. A. (1995). A pollen morphological study of Dyschoriste Nees and Chaetacanthus Nees (Acanthaceae: Ruellieae). Review of Palaeobotany and Palynology, 84(3-4), 331-345.

Furness, C. A. (1996). Pollen morphology of Acanthopsis Harvey, Acanthus L. and Blepharis Jussieu (Acanthaceae: Acantheae). Review of Palaeobotany and Palynology, 92(3-4), 253-268.

Furness, C. A., & Rudall, P. J. (2006). Comparative structure and development of pollen and tapetum in Pandanales. International Journal of Plant Sciences, 167(2), 331-348.

Furness, C. A., Gregory, T., & Rudall, P. J. (2015). Pollen structure and diversity in Liliales. International Journal of Plant Sciences, 176(8), 697-723.

Fuxing, W., & Qiao, C. (1987). Spiniferous acritarchs from the lowest Cambrian, Emei, Sichuan, southwestern China. Review of palaeobotany and palynology, 52(2-3), 161-177.

Galloni, M., Podda, L., Vivarelli, D., & Cristofolini, G. (2007). Pollen presentation, pollen-ovule ratios, and other reproductive traits in Mediterranean Legumes (Fam. Fabaceae-Subfam. Faboideae). Plant Systematics and Evolution, 266(3), 147-164.

García Murillo, P. (1993). Estudio palinológico del género Potamogeton L. en la península Ibérica. Botanica complutensis, 18, 79-92.

García, A., & Playford, G. (2007). The fossil record of Algae. In: Algae of Australia: Introduction (McCarthy, P.M. & Orchard, A.E., eds.), pp. 104-128. ABRS, Canberra; CSIRO Publishing, Melbourne

Garcia-Jacas, N., Susanna, A., Garnatje, T., & Vilatersana, R. (2001). Generic delimitation and phylogeny of the subtribe Centaureinae (Asteraceae): a combined nuclear and chloroplast DNA analysis. Annals of Botany, 87(4), 503-515.

Garcia-Jacas, N., Susanna, A., Mozaffarian, V., & Ilarslan, R. (2000). The natural delimitation of Centaurea (Asteraceae: Cardueae): ITS sequence analysis of the Centaurea jacea group. Plant systematics and evolution, 223(3), 185-199.

Gastony, G. J. (1974). Spore morphology in the Cyatheaceae. I. The perine and sporangial capacity: general considerations. American Journal of Botany, 61(6), 672-680.

Gastony, G. J. (1979). Spore morphology in the Cyatheaceae. III. The genus Trichipteris. American Journal of Botany, 66(10), 1238-1260.

Gastony, G. J., & Tryon, R. M. (1976). Spore morphology in the Cyatheaceae. II. The genera Lophosoria, Metaxya, Sphaeropteris, Alsophila, and Nephelea. American journal of Botany, 63(6), 738-758.

Gentry, A. H., & Tomb, A. S. (1979). Taxonomic implications of Bignoniaceae palynology. Annals of the Missouri Botanical Garden, 756-777.

Giacosa, J. R. (2024). Spore morphology of Schizaea species (Schizaeaceae) from America. Review of Palaeobotany and Palynology, 326, 105127.

Giacosa, J. R., Morbelli, M. A., & Giudice, G. E. (2012). Spore morphology and wall ultrastructure of Anemia Swartz species (Anemiaceae) from Argentina. Review of Palaeobotany and Palynology, 174, 27-38.

Giacosa, J. R., Morbelli, M., & Giudice, G. (2004). Spore wall ultrastructure in Anogramma species (Pteridaceae) from Argentina. Grana, 43(4), 231-237.

Goh, T. K., & Hyde, K. D. (1999). A synopsis of Trichocladium species, based on the literature. Fungal Diversity.

Gomaa, F., Mitchell, E. A., & Lara, E. (2013). Amphitremida (Poche, 1913) is a new major, ubiquitous labyrinthulomycete clade. PloS one, 8(1), e53046.

Goncalves-Esteves, V., Cartaxo-Pinto, S., Marinho, E. B., Esteves, R. L., & Mendonca, C. B. F. (2022). Pollen morphology and evolutionary history of Sapindales. Brazilian Journal of Botany, 45(1), 341-366.

Gonçalves-Esteves, V., Vieira, G. R. M., Carvalho, R. J. P. D., Crespo, S. R. D. M., & Mendonça, C. B. F. (2020). Pollen morphology of some species of Spermacoceae ss (Rubiaceae) of the Atlantic Forest, Rio de Janeiro, Brazil. Acta Botanica Brasilica, 34, 243-255.

Gorrer, D. A., Berrueta, P. C., Ramos Giacosa, J. P., Luna, M. L., & Giudice, G. E. (2021). Spore atlas of isosporate ferns of Punta Lara Nature Reserve, Argentina. Boletín de la Sociedad Argentina de Botánica, 56.

Gosling, W. D., Miller, C. S., & Livingstone, D. A. (2013). Atlas of the tropical West African pollen flora. Review of Palaeobotany and Palynology, 199, 1-135.

Gözcü, M. C., Ceter, T., & Uyar, G. (2018). Spore morphology of some Turkish moss species. Communications Faculty of Sciences University of Ankara Series C Biology, 27(2), 204-214.

Graham, S. A., Diazgranados, M., & Barber, J. C. (2011). Relationships among the confounding genera Ammannia, Hionanthera, Nesaea and Rotala (Lythraceae). Botanical Journal of the Linnean Society, 166(1), 1-19.

Grant, M., Blackmore, S., & Morton, C. (2000). Pollen morphology of the subfamily Aurantioideae (Rutaceae). Grana, 39(1), 8-20.

Grayum, M. H. (1984). Palynology and phylogeny of the Araceae. University of Massachusetts Amherst.

Griffiths, M. E., Tsvuura, Z., Franklin, D. C., & Lawes, M. J. (2010). Pollination ecology of Isoglossa woodii, a long-lived, synchronously monocarpic herb from coastal forests in South Africa. Plant Biology, 12(3), 495-502.

Grímsson, F., Grimm, G. W., & Zetter, R. (2018). Evolution of pollen morphology in Loranthaceae. Grana, 57(1-2), 16-116.

Guarro, J. (1983). A new coprophilous species of Rhytidospora. Mycologia, 75(5), 927-930.

Gurdebeke, P. R., Mertens, K. N., Pospelova, V., Matsuoka, K., Li, Z., Gribble, K. E., ... & Louwye, S. (2020). Taxonomic revision, phylogeny, and cyst wall composition of the dinoflagellate cyst genus Votadinium Reid (Dinophyceae, Peridiniales, Protoperidiniaceae). Palynology, 44(2), 310-335.

Hall, J.D., McCourt, R.M. (2017). Zygnematophyta. In: Archibald, J., Simpson, A., Slamovits, C. (eds) Handbook of the Protists. Springer, Cham. https://doi.org/10.1007/978-3-319-28149-0_41

Hamdy, R., & Shamso, E. (2010). Pollen morphology of Sterculiaceae (s. str.) in Egypt and its taxonomic significance. Egyptian Journal of Botany, 50, 103-117.

Handa, K., Tsuji, S. I., & Tamura, M. (2001). Pollen morphology of Japanese Asparagales and Liliales (Lilianae). Japanese Journal of Historical Botany, 9(2), 85-125.

Harley, M. M. (1985). Pollen morphology and taxonomy of the tribe Fibraureae (Menispermaceae). Kew Bulletin, 553-565.

Harley, M. M. (1991). The pollen morphology of the Sapotaceae. Kew bulletin, 379-491.

Harley, M. M., & Baker, W. J. (2001). Pollen aperture morphology in Arecaceae: Application within phylogenetic analyses, and a summary of record of palm-like pollen the fossil. Grana, 40(1-2), 45-77.

Harley, M. M., & Ferguson, I. K. (1982). Pollen morphology and taxonomy of the tribe Menispermeae (Menispermaceae). Kew Bulletin, 353-366.

Harley, M. M., & Ferguson, I. K. (1982). Pollen morphology and taxonomy of the tribe Menispermeae (Menispermaceae). Kew Bulletin, 353-366.

Harley, M. M., Song, U., & Banks, H. I. (2005). Pollen morphology and systematics of Burseraceae. Grana, 44(4), 282-299.

Hawksworth, D. L., van Geel, B., & Wiltshire, P. E. J. (2016). The enigma of the Diporotheca palynomorph. Review of Palaeobotany and Palynology, 235, 94–98. doi:10.1016/j.revpalbo.2016.09.010

Head, M. J. (1992). Zygospores of the Zygnemataceae (Division Chlorophyta) and other freshwater algal spores from the uppermost Pliocene St. Erth Beds of Cornwall, southwestern England. Micropaleontology, 237-260.

Hennipman, E., Veldhoen, P., & Kramer, K. U. (1990). Polypodiaceae. In Pteridophytes and Gymnosperms (pp. 203-230). Berlin, Heidelberg Springer Berlin Heidelberg.

Herber, B. E. (2002). Pollen morphology of the Thymelaeaceae in relation to its taxonomy. Plant Systematics and Evolution, 232(1), 107-121.

Hoen, P. P., & Punt, W. (1989). Pollen morphology of the tribe Dorstenieae (Moraceae). Review of palaeobotany and palynology, 57(3-4), 187-220.

https://globalpollenproject.org/Reference/6828eff4-ff0d-4d59-9dfe-ff174d52f5d9/GPP21

https://globalpollenproject.org/Reference/6828eff4-ff0d-4d59-9dfe-ff174d52f5d9/GPP22

https://globalpollenproject.org/Reference/891fa5a1-ebfc-4360-83be-69fdf965219b/139.2.1%20-%201

https://globalpollenproject.org/Reference/891fa5a1-ebfc-4360-83be-69fdf965219b/139.2.1%20-%202

https://globalpollenproject.org/Reference/de35675e-55b9-4798-a3f0-e7a57c174c8a/GPP442

https://globalpollenproject.org/Taxon/ID/c0388edd-ba4e-4575-a3a2-9c605d10ebaa

https://palsys.org/species/10

https://www.algaebase.org/search/genus/detail/?genus_id=44645

https://www.algaebase.org/search/genus/detail/?genus_id=51491

https://www.marum.de/en/Karin-Zonneveld/Modern-Dinocyst-Key/Ataxodinium-choane.html

Hu, Z., Zhao, C., Zhao, Y., & Liu, J. (2021). Pollen morphology of Liliaceae and its systematic significance. Palynology, 45(3), 531-568.

Huysmans, S., Dessein, S., Smets, E., & Robbrecht, E. (2003). Pollen morphology of NW European representatives confirms monophyly of Rubieae (Rubiaceae). Review of Palaeobotany and Palynology, 127(3-4), 219-240.

Huysmans, S., Robbrecht, E., & Smets, E. (1994). Are the genera Hallea and Mitragyna (Rubiaceae-Coptosapelteae) pollen morphologically distinct?. Blumea: Biodiversity, Evolution and Biogeography of Plants, 39(1/2), 321-340.

Huysmans, S., Robbrecht, E., & Smets, E. (1998). A collapsed tribe revisited: pollen morphology of the Isertieae (Cinchonoideae–Rubiaceae). Review of Palaeobotany and Palynology, 104(2), 85-113.

Ibarra-Morales, A., Muñíz, M. E., & Valencia, S. (2015). The Genus Anthoceros (Anthocerotaceae, Anthocerotophyta) in Central Mexico. Phytotaxa, 205(4), 215-228.

Ickert-Bond, S. M., Skvarla, J. J., & Chissoe, W. F. (2003). Pollen dimorphism in Ephedra L.(Ephedraceae). Review of Palaeobotany and Palynology, 124(3-4), 325-334.

Irfan, M., Jan, G., Jan, F. G., & Murad, W. (2021). Taxonomy and spore morphology of genus Adiantum (Vittarioideae; Pteridaceae) from Pakistan. Microscopy Research and Technique, 84(11), 2727-2736.

Ivanov, D. & Belkinova, D. (2021). Closterium mosbruggeri sp. nov.: a new fossil species from the middle Miocene of Northwest Bulgaria. Palaeobiodiversity and Palaeoenvironments 101: 69-74, 4 figures.

Iwarsson, M. (1977). Pollen morphology of east African Caryophyllaceae. Grana, 16(1), 15-22.

Jaca, T. P., Boatwright, J. S., & Moteetee, A. N. (2018). Taxonomic studies of the genus Rhynchosia Lour.(Phaseoleae, Fabaceae) in South Africa A review of section Chrysoscias. South African Journal of Botany, 117, 119-133.

Jankovská, V., Komárek, J. Indicative value of Pediastrum and other coccal green algae in palaeoecology. Folia Geobot 35, 59–82 (2000). https://doi.org/10.1007/BF02803087

Jansen, S., Robbrecht, E., Beeckman, H., & Smets, E. (1996). Gaertnera and Pagamea: genera within the Psychotrieae or constituting the tribe Gaertnereae? A wood anatomical and palynological approach. Botanica Acta, 109(6), 466-476.

Jermy, A. C. (1990). Isoetaceae. In Pteridophytes and gymnosperms (pp. 26-31). Berlin, Heidelberg Springer Berlin Heidelberg.

Joly, C., Barillé, L., Barreau, M., Mancheron, A., & Visset, L. (2007). Grain and annulus diameter as criteria for distinguishing pollen grains of cereals from wild grasses. Review of palaeobotany and palynology, 146(1-4), 221-233.

Jones, M. R., & Clarke, G. C. S. (1981). Nymphaeaceae. Review of Palaeobotany and Palynology, 33(1), 57-67.

Joshi, H., Aggarwal, N. Palynological dating and incidence of the fossil Botryococcus in variable pH from the Mesozoic sediments of the Godavari Valley Coalfield, South India: insights in palaeoecology and palaeoenvironment. Environ Earth Sci 83, 218 (2024). https://doi.org/10.1007/s12665-024-11527-z

Júnior, C. E. A. S., e Lima, L. C. L., & Saba, M. D. (2015). Palynological study of heterostylous species of Melochia L.(Byttinerioideae-Malvaceae) occurring in Bahia, Brazil. Review of Palaeobotany and Palynology, 221, 192-203.

Katinas, L., Tellería, M. C., Susanna de la Serna, A., & Ortiz, S. (2008). Warionia (Asteraceae): a relict genus of Cichorieae?.

Keating, R. C. (1972). The comparative morphology of the Cochlospermaceae. III. The flower and pollen. Annals of the Missouri Botanical Garden, 282-296.

Keating, R. C. (1973). Pollen morphology and relationships of the Flacourtiaceae. Annals of the Missouri Botanical Garden, 273-305.

Koekemoer, M. (2019). Taxonomy and reclassification of South African Asteraceae genus Elytropappus (Gnaphalieae, Asteraceae), the description of two new genera and two new species. Phytotaxa, 403(4), 248.

Köhler, E. (1965). Die Pollenmorphologie der biovulaten Euphorbiaceae und ihre Bedeutung für die Taxonomie. Grana, 6(1), 26-120.

Kosenko, V. (1999). Pollen morphology in the family Asphodelaceae (Asphodeleae, Kniphofieae). Grana, 38(4), 218-227.

Kott, L., & Britton, D. M. (1983). Spore morphology and taxonomy of Isoetes in northeastern North America. Canadian Journal of Botany, 61(12), 3140-3163.

Kramer, K. U. (1990). Dennstaedtiaceae. In Pteridophytes and gymnosperms (pp. 81-94). Berlin, Heidelberg: Springer Berlin Heidelberg.

Kramer, K.U. (1990). Schizaeaceae. In: Kramer, K.U., Green, P.S. (eds) Pteridophytes and Gymnosperms. The Families and Genera of Vascular Plants, vol 1. Springer, Berlin, Heidelberg. https://doi.org/10.1007/978-3-662-02604-5_44

Kuang, Y., Kirchoff, B. K., Tang, Y., Liang, Y., & Liao, J. (2008). Palynological characters and their systematic significance in Naucleeae (Cinchonoideae, Rubiaceae). Review of Palaeobotany and Palynology, 151(3-4), 123-135.

Kubitzki, M. (1965) Palynologia Madagassica et Mascarenica Familles 147-154. Pollen et Spores 8(3) 492-508,

Kundu, S., Bianchinotti, M. V., & Khan, M. A. (2024). The first evidence of saprophytic Tetraploa on Siwalik (Late Miocene) monocot leaf from western Himalaya and its role in palaeoecology reconstruction. Fungal Biology, 128(3), 1742-1750.

Kutluk, H., & Mazei, Y. (2018). Organic-walled fossil testate amoebae records (late Cretaceous–holocene) from the Neotethyan–mediterranean region. Journal of Foraminiferal Research, 48(2), 121-141.

LAND, F. J. (1986). Jurassic and Lower Cretaceous palynomorph assemblages from Cape Flora, Franz Josef Land, Arctic, USSR. NORSK GEOLOGISK TIDSSKRIFT, 66(50), 55.

Large, M. F., & Mabberley, D. J. (1995). An assessment of pollen morphology in the genus Vitex L.(Labiatae). Grana, 34(5), 291-299.

Lattar, E. C., Bóbeda, G., & Zini, L. M. (2020). Pollen morphological and morphometric analysis in species of the Aeschynomeneae and Adesmieae tribes (Faboideae: Fabaceae). Palynology, 44(1), 187-194.

Leal, A., Martinez-Blanco, X., Beri, A., & del Puerto, L. (2021). A combined catalog of non-pollen palynomorphs (NPPs) of fungal origin from soil and airborne samples of Uruguay. Review of Palaeobotany and Palynology, 293, 104488.

Lee, S. J., & Park, C. W. (2014). Spore morphology of the genus Dryopteris Adans.(Dryopteridaceae) in Korea. Journal of Plant Biology, 57(5), 302-311.

Lens, F., Jansen, S., Huysmans, S., Robbrecht, E., & Smets, E. (2000). Pollen morphological variation in Vanguerieae (Ixoroideae Rubiaceae). Grana, 39(2-3), 90-102.

Lézine, A. M., Watrin, J., Vincens, A., & Hély, C. (2009). Are modern pollen data representative of west African vegetation?. Review of Palaeobotany and Palynology, 156(3-4), 265-276.

Lifante, Z. D. (1996). Pollen morphology of Asphodelus L.(Asphodelaceae): taxonomic and phylogenetic inferences at the infrageneric level. Grana, 35(1), 24-32.

Lipps, J. H., Stoeck, T., & Dunthorn, M. (2013). Fossil tintinnids. The biology and ecology of tintinnid ciliates: models for marine plankton. Oxford: Wiley-Blackwell, 186-197.

Liu, E., Liu, H. Y., & Liu, Y. C. (2024). Spore SEM studies on the genus Polystichum, Dryopteridaceae (Polypodiale, Pteridophyta). Taiwania, 69(2), 185-206.

Liu, Y. S., & Basinger, J. F. (2000). Fossil Cathaya (Pinaceae) pollen from the Canadian high arctic. International Journal of Plant Sciences, 161(5), 829-847.

Lobreau-Callen, D. (1983). Analyse de la repartition géographique des Malphigiaceae d'apres les caractêres du pollen et de la pollinisation. Bothalia, 14(3/4), 871-881.

López-Vinyallonga, S., Romaschenko, K., Susanna, A., & Garcia-Jacas, N. (2011). Systematics of the Arctioid group: Disentangling Arctium and Cousinia (Cardueae, Carduinae). Taxon, 60(2), 539-554.

Lowrie, S. R. 1982. The palynology of the Malpighiaceae and its contribution to family systematics. Ph.D. dissertation, University Microfilms #82–24999. University of Michigan, Ann Arbor, USA

Lu, K. Q., Xie, G., Li, M., Li, J. F., Trivedi, A., Ferguson, D. K., ... & Wang, Y. F. (2018). Dataset of pollen morphological traits of 56 dominant species among desert vegetation in the eastern arid central Asia. Data in Brief, 18, 1022-1046.

Luck-Allen, E. R., & Cain, R. F. (1975). Additions to the genus Delitschia. Canadian Journal of Botany, 53(17), 1827-1887.

Márquez, G. J., Morbelli, M. A., & Giudice, G. E. (2010). Spore morphology and ultrastructure of Cyathea (Cyatheaceae, Pteridophyta) species from southern South America. Grana, 49(4), 269-280.

Marret, F., & Zonneveld, K. A. (2003). Atlas of modern organic-walled dinoflagellate cyst distribution. Review of Palaeobotany and Palynology, 125(1-2), 1-200.

Martin, A. R. H. (1959). South African palynological studies. I: statistical and morphological variation in the pollen of the South African species of podocarpus. Grana, 2(1), 40-68.

Mathew, P. J., & Mathew, P. M. (2001). Pollen morphology of some members of Piperaceae and its bearing on the systematics and phylogeny of the family. RHEEDEA-KERALA-, 11(2), 65-78.

Maury, G., Muller, J., & Lugardon, B. (1975). Notes on the morphology and fine structure of the exine of some pollen types in Dipterocarpaceae. Review of Palaeobotany and Palynology, 19(4), 241-289.

McClymont, J. W. (1955). Spores of the Musci: their structure and significance in systematic research. University of Michigan.

McQueen, C. B. (1985). Spore morphology of four species of Sphagnum in section Acutifolia. Bryologist, 1-4.

Merckx, V., Schols, P., Geuten, K., Huysmans, S., & Smets, E. (2008). Phylogenetic relationships in Nartheciaceae (Dioscoreales), with focus on pollen and orbicule morphology. Belgian Journal of Botany, 64-77.

Mertens, K. N., Gu, H., Takano, Y., Price, A. M., Pospelova, V., Bogus, K., ... & Matsuoka, K. (2017). The cyst-theca relation of Trinovantedinium pallidifulvum, with erection of Protoperidinium lousianensis sp. nov. and their phylogenetic position within the Conica group. Palynology, 41(2), 183-202.

Merville M., (1965) Le pollen des Sapindacées d'Afrique Orientale. Pollen et Spores 8(3) 465-489

Mickel, J. T. (1974). A redefinition of the genus Hemionitis. American Fern Journal, 64(1), 3-12.

Miller, A. N., & Huhndorf, S. M. (2004). A natural classification of Lasiosphaeria based on nuclear LSU rDNA sequences. Mycological Research, 108(1), 26-34.

Mitra, K. (1975). Contribution to the pollen morphology of the family Capparaceae. Nelumbo-The Bulletin of the Botanical Survey of India, 7-31.

Miyosi, N. (1966). Spore morphology of Hepaticae in Japan.

Molina, R. T., & Jiménez, J. L. U. (1995). Tipos polínicos de la Tribu Cardueae en la península Ibérica. Monografías del Real Jardín Botánico de Córdoba, 2, 5-52.

Moore, P. D., Webb, J. A., & Collison, M. E. (1991). Pollen analysis. Blackwell scientific publications.

Morbelli, M. A., Piñeiro, M. R., & Giudice, G. E. (2010). Spore morphology and wall ultrastructure of Hymenophyllaceae Link (Pteridophyta) from north-west Argentina. Grana, 49(1), 37-46.

Morgado, L. N., Gonçalves-Esteves, V., Resendes, R., & Ventura, M. A. M. (2018). A pollen inventory of endemic species from the Azores archipelago, Portugal. Palynology, 42(2), 273-289.

Morton, C. M., & Dickison, W. C. (1992). Comparative pollen morphology of the Styracaceae. Grana, 31(1), 1-15.

Muller, J. (1969). Pollen-morphological notes on Ochnaceae. Review of Palaeobotany and Palynology, 9(3-4), 149-173.

Nagamasu, H. (1989). Pollen morphology of Japanese Symplocos (Symplocaceae). The botanical magazine= Shokubutsu-gaku-zasshi, 102(2), 149-164.

Nayar, B. K., & Bajpai, N. (1964). Morphology of the gametophytes of some species of Pellaea and Notholaena. Journal of the Linnean Society of London, Botany, 59(376), 63-76.

Nelson Mandela University Pollen Ref Collection: https://pollen.mandela.ac.za/default.aspx?moid=1545

Nickrent, D. L., Der, J. P., & Anderson, F. E. (2005). Discovery of the photosynthetic relatives of the" Maltese mushroom" Cynomorium. BMC Evolutionary Biology, 5, 1-11.

Nilsson, S. (1990). Taxonomic and evolutionary significance of pollen morphology in the Apocynaceae. In Morphology, development, and systematic relevance of pollen and spores (pp. 91-102). Springer Vienna.

Nour, I. H., Alhadead, K., Ellmouni, F. Y., Badr, R., Saad, T. I., El-Banhawy, A., & Abdel Rahman, S. M. (2023). Morphological, anatomical and chemical characterization of Ricinus communis L.(Euphorbiaceae). Agronomy, 13(4), 985.

Nowicke, J. W. (1968). Palynotaxonomic study of the Phytolaccaceae. Annals of the Missouri Botanical Garden, 294-364.

Nowicke, J. W. (1975). Pollen morphology in the order Centrospermae. Grana, 15(1-3), 51-77.

Nowicke, J. W. (1981). Pollen morphology and the phylogenetic realtionships of the Berberidaceae. Smithsonian Contributions to Botany.

Nowicke, J. W. (1994). A palynological study of Crotonoideae (Euphorbiaceae). Annals of the Missouri Botanical Garden, 245-269.

Nowicke, J. W., & Ridgway, J. E. (1973). Pollen studies in the genus Cordia (Boraginaceae). American Journal of Botany, 60(6), 584-591.

Nowicke, J.W., 1984. A palynological study of Pandaceae. Pollen et spores 26, 3 142.

Nzabandora, C. K. (2016). Atlas pollinique des régions montagneuses bordières du Lac Kivu. Geo-Eco-Trop, 40(1), 1-74.

Olejnik, N., Celka, Z., Szkudlarz, P., & Shevera, M. V. (2018). Taxonomic significance of morphological characters of spores in the family Ophioglossaceae (Psilotopsida). Review of Palaeobotany and Palynology, 252, 77-85.

Oliveira, A. C. D. S., De Borges, R. L. B., Perez, A. P. F., Lewis, G. P., & Silva, J. S. (2019). Characteristics of the exine and aperture of pollen grains of Eriosema and Rhynchosia (Leguminosae–Papilionoideae–Phaseoleae). Grana, 58(4), 292-307.

Orlova, T. Y., & Morozova, T. V. (2013). Dinoflagellate cysts in recent marine sediments of the western coast of the Bering Sea. Russian Journal of Marine Biology, 39(1), 15-29.

Osman, A., & Hassan, N. (2015). The palynology of Aizoaceae and Molluginaceae in Egypt and Sudan. Palynology, 39(1), 19-36.

Osman, A., Al-Ghamdi, F., & Guetat, A. (2014). Pollen morphology of some species of genus Astragalus L.(Fabaceae) in Northern region of Saudi Arabia. Life Science Journal, 11(11), 1006-1019.

Oybak Dönmez, E., & IşIK, S. (2008). Pollen morphology of Turkish Amaryllidaceae, Ixioliriaceae and Iridaceae. Grana, 47(1), 15-38.

Passarelli, L. M., Gabriel y Galán, J. M., Prada, C., & Rolleri, C. H. (2010). Spore morphology and ornamentation in the genus Blechnum (Blechnaceae). Grana, 49(4), 243-262.

Patel, V. C., Skvarla, J. J., & Raven, P. H. (1984). Pollen characters in relation to the delimitation of Myrtales. Annals of the Missouri Botanical Garden, 858-969.

Peng, T., & Zhu, R. L. (2013). A revision of the genus Anthoceros (Anthocerotaceae, Anthocerotophyta) in China. Phytotaxa, 100(1), 21-35.

Persson, C. (1993). Pollen morphology of the Gardenieae-Gardeniinae (Rubiaceae). Nordic Journal of Botany, 13(5), 561-582.

Perveen, A. , & Qaiser, M. (2001). Pollen flora of Pakistan-xxxi Capparidaceae. Turkish Journal of Botany, 25(6), 389-395.

Perveen, A. , & Qaiser, M. (2010). Pollen flora of Pakistan—LXVII: Acanthaceae. Pakistan Journal of Botany, 42(SI), 175-191.

Perveen, A. , & Qaiser, M.(2009). Pollen flora of Pakistan-Malvaceae: Dombeyoideae-Lxii. Pakistan Journal of Botany, 41(2), 491-494.

Perveen, A. N. J. U. M., & Qaiser, M. (2010). Pollen flora of Pakistan—LXVII: Acanthaceae. Pakistan Journal of Botany, 42(SI), 175-191.

Perveen, A., & Qaiser, M. (1998). Pollen Flora of Pakistan-VIII Leguminosae (subfamily: Papilionoideae). Turkish Journal of Botany, 22(2), 73-92.

Perveen, A., & Qaiser, M. (1998). Pollen Flora of Pakistan-X. Leguminosae (Subfamily: Caesalpinioideae). Turkish Journal of Botany, 22(3), 145-150.

Perveen, A., & Qaiser, M. (2000). Pollen Flora of Pakistan-XIX. Aizoaceae. Turkish Journal of Botany, 24(1), 29-33.

Perveen, A., & Qaiser, M. (2007). Pollen flora of Pakistan-LIII. Verbenaceae. Pak J Bot, 39(3), 663-669.

Perveen, A., Grafström, E., & El-Ghazaly, G. (2004). World Pollen and Spore Flora 23. Malvaceae Adams. Pp Subfamilies: Grewioideae, Tilioideae, Brownlowioideae. Grana, 43(3), 129-155.

Pierce, S. T. (1976). Morphology of schizosporis reticulatus cqqkson and Dettmann 1959. Geoscience and Man, 15(1), 25-33.

Pocknall, D. T. (1981). Pollen morphology of the New Zealand species of Dacrydium selander, Podocarpus L'heritier, and Dacrycarpus endlicher (podocarpaceae). New Zealand journal of botany, 19(1), 67-95.

Pocknall, D. T., Clowes, C. D., & Jarzen, D. M. (2023). Spinizonocolpites prominatus (McIntyre) Stover & Evans: fossil Nypa pollen, taxonomy, morphology, global distribution, and paleoenvironmental significance. New Zealand Journal of Geology and Geophysics, 66(3), 558-570.

Polevova, S., Tekleva, M., Neumann, F. H., Scott, L., & Stager, J. C. (2010). Pollen morphology, ultrastructure and taphonomy of the Neuradaceae with special reference to Neurada procumbens L. and Grielum humifusum E. Mey. ex Harv. et Sond. Review of Palaeobotany and Palynology, 160(3-4), 163-171.

Pöltl, M., Clark, A. T., Stadlober, T., & Berg, C. (2024). Spore variability in Hepaticae: a case study on four short-lived Riccia L. species. Journal of Bryology, 46(1), 31-44.

Poole, M. M., & Hunt, D. R. (1980). Pollen morphology and the taxonomy of the commelinaceae: an exploratory survey: American Commelinaceae: VIII. Kew Bulletin, 639-660.

Praglowski, J. (1970). The pollen morphology of the Haloragaceae with reference to taxonomy. Grana, 10(3), 159-239.

Praglowski, J. (1973) The pollen morphology of the Theligonaceae with reference to taxonomy. Pollen et Spores 15: 3

Punt, W. (1962). Pollen morphology of the Euphorbiaceae with special reference to taxonomy. Wentia, 7(1), 1-116.

Punt, W. (1975). Pollen morphology of the Dichapetalaceae with special reference to evolutionary trends and mutual relationships of pollen types. Review of Palaeobotany and Palynology, 19(1), 1-97.

Punt, W. (1978). Evolutionary trends in the Potalieae (Loganiaceae). Review of Palaeobotany and Palynology, 26(1-4), 313-335.

Punt, W., & Den Breejen, P. (1981). Linaceae. Review of Palaeobotany and Palynology, 33(1), 75-115.

Punt, W., & Eetgerink, E. (1982). On the pollen morphology of some genera of the tribe Moreae (Moraceae). Grana, 21(1), 15-19.

Punt, W., & Hoen, P. P. (1995). Caryophyllaceae. Review of Palaeobotany and Palynology, 88(1-4), 83-272.

Punt, W., & Hoen, P. P. (2009). The Northwest European Pollen Flora, 70: Asteraceae—Asteroideae. Review of Palaeobotany and Palynology, 157(1-2), 22-183.

Punt, W., & Langewis, E. A. (1988). Verbenaceae. Review of Palaeobotany and Palynology, 57(1-2), 75-79.

Punt, W., & Leenhouts, P. W. (1967). Pollen morphology and taxonomy in the Loganiaceae. Grana, 7(2-3), 469-516.

Punt, W., & Malotaux, M. (1984). Cannabaceae, moraceae and urticaceae. Review of Palaeobotany and Palynology, 42(1-4), 23-44.

Punt, W., & Marks, A. (1991). Buxaceae. Review of palaeobotany and palynology, 69(1-3), 113-115.

Punt, W., & Marks, A. (1991). Globulariaceae. Review of palaeobotany and palynology, 69(1-3), 109-112.

Punt, W., & Marks, A. (1995). Resedaceae. Review of Palaeobotany and Palynology, 88(1-4), 47-59.

Punt, W., & Monna-Brands, M. (1977). Solanaceae. Review of Palaeobotany and Palynology, 23(2), 1-30.

Punt, W., & Nienhuis, W. (1976). Gentianaceae. Review of Palaeobotany and Palynology, 21(2), 89-123.

Punt, W., & Schmitz, M. B. (1981). Aquifoliaceae. Review of Palaeobotany and Palynology, 33(1), 69-74.

Punt, W., Blackmore, S., Hoen, P. P., & Stafford, P. J. (Eds.). (2003). The Northwest European Pollen Flora: Reprinted from Review of Palaeobotany and Palynology, Volume 123/1-2 (Vol. 121). Elsevier.

Punt, W., Bos, J. A., & Hoen, P. P. (1991). The northwest European pollen flora, 45. Oleaceae.

Punt, W., Reitsma, T. J., & Reuvers, A. A. (1974). Caprifoliaceae. Review of Palaeobotany and Palynology, 17(3-4), 5-29.

Punt, W., Rovers, J., & Hoen, P. P. (2003). Onagraceae. Review of Palaeobotany and Palynology, 123(1-2), 107-161.

Qaiser, M. , & Perveen, A. (2004). Pollen Flora of Pakistan-XXXVII. Tamaricaceae. Pakistan Journal of Botany, 36(1), 1-18.

Quijano-Abril, M. A., Castaño-López, M. D. L. Á., Marín-Henao, D., Sánchez-Gómez, D., Rojas-Villa, J. M., & Sierra-Escobar, J. (2021). Functional traits of invasive species Thunbergia alata (Acanthaceae) and its importance in the adaptation to Andean forests. Acta botánica mexicana, (128).

Radi, T., Bonnet, S., Cormier, M. A., de Vernal, A., Durantou, L., Faubert, É., ... & Van Nieuwenhove, N. (2013). Operational taxonomy and (paleo-) autecology of round, brown, spiny dinoflagellate cysts from the Quaternary of high northern latitudes. Marine Micropaleontology, 98, 41-57.

Raj, B. (1961). Pollen morphological studies in the Acanthaceae. Almqvist & Wiksells, Uppsala

Raj, B. (1983). A contribution to the pollen morphology of Verbenaceae. Review of Palaeobotany and Palynology, 39(3-4), 343-422.

Rasoloarijao, T. M., Ramavovololona, P., Ramamonjisoa, R., Clemencet, J., Lebreton, G., & Delatte, H. (2019). Pollen morphology of melliferous plants for Apis mellifera unicolor in the tropical rainforest of Ranomafana National Park, Madagascar. Palynology, 43(2), 292-320.

Razafimandimbison, S. G., & Rydin, C. (2024). Phylogeny and classification of the coffee family (Rubiaceae, Gentianales): Overview and outlook. Taxon, 73(3), 673-717.

Réblová, M., Hernández-Restrepo, M., Fournier, J., & Nekvindová, J. (2020). New insights into the systematics of Bactrodesmium and its allies and introducing new genera, species and morphological patterns in the Pleurotheciales and Savoryellales (Sordariomycetes). Studies in Mycology, 95(1), 415-466.

Reille, M. (1967) Contribution a l'etude palynologique de la famille des Vitaceés. Pollen et spores 9(2) 282-363.

Reille, M. (1992). Pollen et spores d'Europe et d'Afrique du Nord. Marseille: Laboratoire de Botanique Historique et Palynologie.

Reille, M. (1995). Pollen et Spores d'Europe et d'Afrique du Nord, supplément 1, Marseille: Laboratoire de Botanique Historique et Palynologie.

Rich, F. J., Kuehn, D., & Davies, T. D. (1982). The paleoecological significance of Ovoidites. Palynology, 6(1), 19-28.

Rincón Baron, E. J., Hilda Rolleri, C., Passarelli, L. M., Espinosa Matías, S., & Torres, A. M. (2014). Sporogenesis, sporoderm and mature spore ornamentation in Lycopodiaceae. Revista de Biología Tropical, 62(3), 1161-1195.

Riollet, G., & Bonnefille, R. (1976). Pollen des Amaranthacées du bassin du lac Rodolphe (Afrique Orientale). Determinations générique et spécifique. Pollen et spores, 18(1), 67-92.

Robbrecht, E. (1982). Pollen morphology of the tribes Anthospermeae and Paederieae (Rubiaceae) in relation to taxonomy. Bulletin du Jardin botanique national de Belgique/Bulletin van de Nationale Plantentuin van Belgie, 349-366.

Robinson, H., Skvarla, J. J., & Funk, V. A. (2016). Vernonieae (Asteraceae) of southern Africa: A generic disposition of the species and a study of their pollen. PhytoKeys, (60), 49.

Rodondi, G., Beretta, M., & Andreis, C. (2010). Pollen morphology of alpine butterworts (Pinguicula L., Lentibulariaceae). Review of Palaeobotany and Palynology, 162(1), 1-10.

Rodrigues, K. M., & Rodrigues, B. F. (2020). Glomus. In Beneficial microbes in agro-ecology (pp. 561-569). Academic Press.

Rodríguez-Riaño, T., Ortega-Olivencia, A., & Devesa, J. A. (1999). Reproductive biology in two Genisteae (Papilionoideae) endemic of the western Mediterranean region: Cytisus striatus and Retama sphaerocarpa. Canadian Journal of Botany, 77(6), 809-820.

Rothfels, C. J., Sundue, M. A., Kuo, L. Y., Larsson, A., Kato, M., Schuettpelz, E., & Pryer, K. M. (2012). A revised family–level classification for eupolypod II ferns (Polypodiidae: Polypodiales). Taxon, 61(3), 515-533.

Roubik, D. W., & Moreno P, J. E. (1991). Pollen and spores of Barro Colorado Island [Panama].

Rowley, J. R., Skvarla, J. J., & Pettitt, J. M. (1992). Pollen wall development in Eucommia ulmoides (Eucommiaceae). Review of palaeobotany and palynology, 70(4), 297-323.

Rozefelds, A. C., Dettmann, M. E., Clifford, H. T., & Carpenter, R. J. (2017). Lygodium (Schizaeaceae) in southern high latitudes during the Cenozoic—a new species and new insights into character evolution in the genus. Review of Palaeobotany and Palynology, 247, 40-52.

Ščevková, J., Tropeková, M., Dušička, J., Štefániková, N., Žilka, M., Zahradníková, E., ... & Mišíková, K. (2024). Moss spores overlooked airborne bioparticles in an urban environment. Environmental Science and Pollution Research, 31(47), 58010-58020.

Saad, S. I. (1960). The sporoderm stratification in the Malvaceae. Pollen et Spores, 1(2), 13-41.

Saad, S. I., & El-Ghazaly, G. (1988). Pollen morphology of some species of Euphorbiaceae. Grana, 27(3), 165-175.

Sagun, V. G., Levin, G. A., & van der Ham, R. W. (2006). Pollen morphology and ultrastructure of Acalypha (Euphorbiaceae). Review of Palaeobotany and Palynology, 140(1-2), 123-143.

Sarkar, B., Basak, S., Das, A. P., Siddhanta, S., Maity, D., & Bera, S. (2021). Pollen morphology of some Eastern Himalayan Species of Maesa (Primulaceae) and its taxonomic significance. Proceedings of the National Academy of Sciences, India Section B: Biological Sciences, 91, 269-275.

Satabié, B. (1974). Contribution de la palynologie à l'étude des Irvingiacées d'Afrique Tropicale. Adansonia, 14(2), 277-289.

Saxena, R. K., Wijayawardene, N. N., Dai, D. Q., Hyde, K. D., & Kirk, P. M. (2021). Diversity in fossil fungal spores. Mycosphere, 12(1), 670-874.

Schlütz, F., & Shumilovskikh, L. S. (2017). Non-pollen palynomorphs notes: 1. Type HdV-368 (Podospora-type), descriptions of associated species, and the first key to related spore types. Review of palaeobotany and palynology, 239, 47-54.

Schneider, H., & Pryer, K. M. (2002). Structure and function of spores in the aquatic heterosporous fern family Marsileaceae. International Journal of Plant Sciences, 163(4), 485-505.

Schols, P., Wilkin, P., Furness, C. A., Huysmans, S., & Smets, E. (2005). Pollen evolution in yams (Dioscorea: Dioscoreaceae). Systematic botany, 30(4), 750-758.

Schori, M., & Furness, C. A. (2014). Pollen diversity in Aquifoliales. Botanical journal of the Linnean Society, 175(2), 169-190.

Schueler, L., & Hemp, A. (2016). Atlas of pollen and spores and their parent taxa of Mt Kilimanjaro and tropical East Africa. Quaternary International, 425, 301-386.

Scott, L. (1982). Late Quaternary fossil pollen grains from the Transvaal, South Africa. Review of Palaeobotany and Palynology, 36(3-4), 241-278.

Seetharam, Y. N. (1985). Clusiaceae: palynology and systematics. Travaux de la section scientifique et technique. Tome XXI. Institut Français de Pondichery.

Sengupta, S. (1972). On the pollen morphology of Convolvulaceae with special reference to taxonomy. Review of Palaeobotany and Palynology, 13(3-4), 157-212.

Shah, S. N., Ahmad, M., Ansari, M. J., Khan, M. N., Iqbal, M., Ali, B., & Al Obaid, S. (2025). Spore morphometric analysis in Sino-Himalayan and W-Himalayan Athyriaceae taxa: taxonomic insights and molecular phylogenetic comparison. Genetic Resources and Crop Evolution, 72(1), 1183-1204.

Shah, S. N., Ahmad, M., Zafar, M., Hadi, F., Khan, M. N., Noor, A., ... & Iqbal, M. (2020). Spore morphology and leaf epidermal anatomy as a taxonomic source in the identification of Asplenium species from Malakand division Pakistan. Microscopy Research and Technique, 83(11), 1354-1368.

Shamso, E. (2013). A palynological study of Acanthaceae in Egypt and its systematic implication. Egypt J. Bot, 53, 257-272.

Sheffy, M. V., & Dilcher, D. L. (1971). Morphology and taxonomy of fungal spores. E. Schweizerbart'sche Verlagsbuchhandlung (Nägele u. Obermiller).

Shi, W., Wen, J., & Lutz, S. (2013). Pollen morphology of the Maddenia clade of Prunus and its taxonomic and phylogenetic implications. Journal of Systematics and Evolution, 51(2), 164-183.

Silva, F. A. D., Kameyama, C., Betancur, J., & Zappi, D. C. (2023). Mendoncia amabilis (Acanthaceae), a remarkable new species from the Amazon basin. Phytotaxa, 612(2), 229-236.

Simpson, M. G. (1987). Pollen ultrastructure of the Pontederiaceae: evidence for exine homology with the Haemodoraceae. Grana, 26(2), 113-126.

Singh, V., & Barinova, S. (2022). Palynological Analysis of Surface Sediments in a High Arctic Pond, Revealing Desmids as Indicators of Wetlands and Climate Change. Transylvanian Review of Systematical and Ecological Research, 24(1), 1-16.

Smith, A.R. (1990). Thelypteridaceae. In: Kramer, K.U., Green, P.S. (eds) Pteridophytes and Gymnosperms. The Families and Genera of Vascular Plants, vol 1. Springer, Berlin, Heidelberg. https://doi.org/10.1007/978-3-662-02604-5_45

Song, J. H., Oak, M. K., & Hong, S. P. (2016). Morphological traits in an androdioecious species, Chionanthus retusus (Oleaceae). Flora, 223, 129-137.

Sorsa, P. (1969, January). Pollen morphological studies on the Mimosaceae. In Annales Botanici Fennici (pp. 1-34). SOCIETAS BIOLOGICA FENNICA VANAMO.

Sorsa, P., & Huttunen, P. (1975, January). On the pollen morphology of the Urticaceae. In Annales Botanici Fennici (pp. 165-182). SOCIETAS BIOLOGICA FENNICA VANAMO.

Souza, T., Helenes, J., Carvalho, M. A., Barreto, C. F., & Baptista Neto, J. A. (2024). Climatic variation of the last 29,000 years BP in the northern Santos basin (Rio de Janeiro Shelf) inferred by an alternation of cysts Tuberculodinium vancampoae and Operculodinium centrocarpum. Journal of South American Earth Sciences, 138, 104878. https://doi.org/10.1016/j.jsames.2024.104878

Sowunmi, M. A. (1995). Pollen of Nigerian plants: II woody species. Grana, 34(2), 120-141.

Sowunmi, M.A., 1973. Pollen grains of Nigerian plants. Grana 13, 145-186.

Spirlet, M.-L. (1965). Utilisation taxonomique des grains de pollen des Passifloracées. I. Pollen et Spores, 7(2), 249-301.

Stafford, P. J. (1995). Ulmaceae. Review of Palaeobotany and Palynology, 88(1-4), 25-46.

Stafford, P. J., & Heath, G. L. A. (1991). Cornaceae. Review of palaeobotany and palynology, 69(1-3), 97-108.

Steyn, E. M., Smith, G. F., Nilsson, S., & Grafström, E. (1998). Pollen morphology in Aloe (Aloaceae). Grana, 37(1), 23-27.

Stuchlik, L. (1967). Pollen morphology and taxonomy of the family Polemoniaceae. Review of Palaeobotany and Palynology, 4(1-4), 325-333.

Susanna, A., & Garcia-Jacas, N. (2009). Cardueae (Carduoideae). Systematics, evolution, and biogeography of Compositae, 293-313.

Tanaka, N., Uehara, K., & Murata, J. (2004). Correlation between pollen morphology and pollination mechanisms in the Hydrocharitaceae. Journal of plant research, 117, 265-276.

Taralova, E. H., Schlecht, J., Barnard, K., & Pryor, B. M. (2011). Modelling and visualizing morphology in the fungus Alternaria. Fungal biology, 115(11), 1163-1173.

Taylor, T. N., Taylor, E. L., & Krings, M. (2009). Algae (2nd ed., pp. 121–160). In T. N. Taylor, E. L. Taylor, & M. Krings (Eds.), Paleobotany. Academic Press. https://doi.org/10.1016/B978-0-12-373972-8.00004-8

Tellería, M. C., & Daners, G. (2003). Pollen types in Southern New World Convolvulaceae and their taxonomic significance. Plant Systematics and Evolution, 243, 99-118.

Testo, W. (2018). Novelties in Costa Rican Pityrogramma (Pteridaceae): A New Species and a New Hybrid from the Osa Peninsula. American Fern Journal, 108(1), 27-33.

Thornhill, A. H., Hope, G. S., Craven, L. A., & Crisp, M. D. (2012). Pollen morphology of the Myrtaceae. Part 1: tribes Eucalypteae, Lophostemoneae, Syncarpieae, Xanthostemoneae and subfamily Psiloxyloideae. Australian Journal of Botany, 60(3), 165-199.

Thornhill, A. H., Hope, G. S., Craven, L. A., & Crisp, M. D. (2012). Pollen morphology of the Myrtaceae. Part 2: tribes Backhousieae, Melaleuceae, Metrosidereae, Osbornieae and Syzygieae. Australian Journal of Botany, 60(3), 200-224.

Thornhill, A. H., Hope, G. S., Craven, L. A., & Crisp, M. D. (2012). Pollen morphology of the myrtaceae. part 4: tribes kanieae, myrteae and tristanieae. Australian Journal of Botany, 60(3), 260-289.

Thornhill, A. H., Wilson, P. G., Drudge, J., Barrett, M. D., Hope, G. S., Craven, L. A., & Crisp, M. D. (2012). Pollen morphology of the Myrtaceae. Part 3: tribes Chamelaucieae, Leptospermeae and Lindsayomyrteae. Australian Journal of Botany, 60(3), 225-259.

Tilney, P. M., & Van Wyk, A. E. (1997). Pollen morphology of Canthium, Keetia and Psydrax (Rubiaceae: Vanguerieae) in southern Africa. Grana, 36(5), 249-260.

Tobe, H., & Takahashi, M. (1990). Trichome and pollen morphology of Barbeya (Barbeyaceae) and its relationships. Taxon, 39(4), 561-567.

ToI, K. (1986). Rhizopod analysis.Handbook of Holocene Palaeoecology and Palaeohydrology

Trappe, J. M. (1971). A synopsis of the Carbomycetaceae and Terfeziaceae (Tuberales). Transactions of the British Mycological Society, 57(1), 85-92.

Tryon, A. F., & Lugardon, B. (2012). Spores of the Pteridophyta: surface, wall structure, and diversity based on electron microscope studies. Springer Science & Business Media.

Tseng, C. C., & Shoup, J. R. (1978). Pollen morphology of Schefflera (Araliaceae). American Journal of Botany, 65(4), 384-394.

Tsou, C. H. (1994). The classification and evolution of pollen types of Planchonioideae (Lecythidaceae). Plant Systematics and Evolution, 189(1), 15-27.

Tsukada, M. (1963). Pollen morphology and identification I. Eucaesalpiniae. Pollen et Spores, 5(2), 239-284.

Turner, F., Pott, R., Schwarz, A., & Schwalb, A. (2014). Response of Pediastrum in German floodplain lakes to Late Glacial climate changes. Journal of Paleolimnology, 52(4), 293-310.

Tyszka, J., Godos, K., Goleń, J., & Radmacher, W. (2021). Foraminiferal organic linings: functional and phylogenetic challenges. Earth-Science Reviews, 220, 103726.

Unkelbach, J., & Reinhardt, A. L. (2024). Atlas of the Oman pollen flora–Pollen morphology of the arid Arabian Peninsula vegetation. Review of Palaeobotany and Palynology, 331, 105204.

Vaganov, A. V. (2016). A comparative study of spore morphology of the subfamily Cryptogrammoideae genera. Ukrainian Journal of Ecology, 6(3), 333-346.

Vaganov, A. V., Gureyeva, I. I., Kuznetsov, A. A., Shmakov, A. I., Romanets, R. S., & König, V. A. (2017). Spore morphology of the representatives of the subfamily Ceratopteridoideae (J. Sm.) RM Tryon from the family Pteridaceae EDM Kirchn.(Pteridophyta). Ukrainian Journal of Ecology, 7(2), 124-129.

Vaganov, A. V., Metzgar, J. S., Sinitsyna, T. A., & Shmakov, A. I. (2020). Comprehensive analysis of Actiniopteris link and Onychium kaulf.(Pteridophyta) relationships according to their phylogeography, phylogeny and spore morphology.

Valdés, B., Díez, M. J., & Fernández, I. (1987). Atlas polínico de Andalucía Occidental. Instituto de Desarrollo Regional de la Universidad de Sevilla. Excma. Diputación de Cádiz.

Van Campo, M. & Hallé, N. (1959). Les pollens des Hippocratéacées d'Afrique de L'Ouest. Pollen et Spores 1(2) 191-272.

Van Campo, M. (1960). Palynologie africaine IV. Bulletin de l'Institut Fondamental d'Afrique Noire (A), 22, 1165-1198.

Van Campo, M., Nilsson, S., & Leeuwenberg, A. J. (1979). Palynotaxonomic studies in Tabernaemontana L. sensu lato (Apocynaceae). Grana, 18(1), 5-14.

van der Ham, R., Mennes, C., & Joan van Heuven, B. (2010). Fevilleoideae pollen (Cucurbitaceae): a study in striate ornamentation. Grana, 49(3), 157-169.

Van Geel, B. (2002). Non-Pollen Palynomorphs. In: Smol, J.P., Birks, H.J.B., Last, W.M., Bradley, R.S., Alverson, K. (eds) Tracking Environmental Change Using Lake Sediments. Developments in Paleoenvironmental Research, vol 3. Springer, Dordrecht. https://doi.org/10.1007/0-306-47668-1_6

van Geel, B., & Aptroot, A. (2006). Fossil ascomycetes in Quaternary deposits. Nova Hedwigia, 82(3), 313-330.

van Geel, B., Engels, S., Martin-Puertas, C., & Brauer, A. (2013). Ascospores of the parasitic fungus Kretzschmaria deusta as rainstorm indicators during a late Holocene beech-forest phase around lake Meerfelder Maar, Germany. Journal of Paleolimnology, 50(1), 33-40.

van Geel, B., Gelorini, V., Lyaruu, A., Aptroot, A., Rucina, S., Marchant, R., ... & Verschuren, D. (2011). Diversity and ecology of tropical African fungal spores from a 25,000-year palaeoenvironmental record in southeastern Kenya. Review of Palaeobotany and Palynology, 164(3-4), 174-190.

Van Leeuwen, P., Punt, W., & Hoen, P. P. (1988). Polygonaceae. Review of Palaeobotany and Palynology, 57(1-2), 81-151.

van Valkenburg, J. L., & Sunderland, T. C. (2008). A revision of the genus Podococcus (Arecaceae). Kew Bulletin, 63(2), 251-260.

Van Wichelen, J., Camelbeke, K., Chaerle, P., Goetghebeur, P., & Huysmans, S. (1999). Comparison of different treatments for LM and SEM studies and systematic value of pollen grains in Cyperaceae. Grana, 38(1), 50-58.

Vanhoorne, R. (1992). Azolla and Salvinia species (Azollaceae and Salviniaceae, Pteridophyta), from the Caenozoic of Belgium. Bull Inst R Sci Nat Belg Sci Terre, 62, 229-355.

Vánky, K., Lutz, M., & Bauer, R. (2008). About the genus Thecaphora (Glomosporiaceae) and its new synonyms. Mycological Progress, 7(1), 31-39.

Verbeek-Reuvers, A. A. (1977). Grossulariaceae. Review of Palaeobotany and Palynology, 24(3), A107-A116.

Verbeek-Reuvers, A. A. (1977). Parnassiaceae. Review of Palaeobotany and Palynology, 24(3), A123-A128.

Verbeek-Reuvers, A. A. (1977). Saxifragaceae. Review of Palaeobotany and Palynology, 24(1), A31-A58.

Verellen, J. E. F., Dessein, S., Razafimandimbison, S. G., Smets, E., & Huysmans, S. (2007). Pollen morphology of the tribes Naucleeae and Hymenodictyeae (Rubiaceae–Cinchonoideae) and its phylogenetic significance. Botanical Journal of the Linnean Society, 153(3), 329-341.

Verhoeven, R. L., & Venter, H. J. T. (1986). Pollen morphology of Monsonia. South African Journal of Botany, 52(4), 361-368.

Verhoeven, R. L., & Venter, H. J. T. (2001). Pollen morphology of the Periplocoideae, Secamonoideae, and Asclepiadoideae (Apocynaceae). Annals of the Missouri Botanical Garden, 569-582.

Verhoeven, R. L., Venter, H. J. T., & Kotze, J. D. (1989). Pollen morphology of Petopentia and Tacazzea (Periplocaceae). South African Journal of Botany, 55(2), 207-214.

Vezey, E. L., Shah, V. P., Skvarla, J. J., & Raven, P. H. (1988). Morphology and phenetics of Rhizophoraceae pollen. Annals of the Missouri Botanical Garden, 1369-1386.

Vilatersana, R., Villodre, J. M., Susanna, A., Garcia-Jacas, N., & Garnatje, T. (2001). Pollen studies in subtribe Centaureinae (Asteraceae): the Carthamus complex and the genus Aegialophila analyzed with electron microscopy. Plant Biology, 3(06), 607-615.

Vnukovskaya, Y. D., Kuzmina, O. B., & Rudaya, N. A. (2024). Local Environmental Conditions of Lake Balyktukel (Russian Altai) in the Holocene from Non-Pollen Palynomorphs. Paleontological Journal, 58(7), 841-850.

Volkova, O. A., Remizowa, M. V., Sokoloff, D. D., & Severova, E. E. (2016). A developmental study of pollen dyads and notes on floral development in Scheuchzeria (Alismatales: Scheuchzeriaceae). Botanical Journal of the Linnean Society, 182(4), 791-810.

Walker, J. W. (1971). Pollen morphology, phytogeography, and phylogeny of the Annonaceae. Contributions from the Gray Herbarium of Harvard University, (202), 1-130.

Walker, J. W., & Walker, A. G. (1980). Comparative pollen morphology of the mainland African genera of Myristicaceae (Cephalosphaera, Coelocaryon, Pycnanthus, and Scyphocephalium). American Journal of Botany, 67(5), 603-611.

Wanntorp, L., Praglowski, J., & Grafström, E. (2004). New insights into the pollen morphology of the genus Gunnera (Gunneraceae). Grana, 43(1), 15-21.

Webster, J., & Lucas, M. T. (1961). Observations on British species of Pleospora. II. Transactions of the British Mycological Society, 44(3), 417-436.

Wilce, J. H. (1972). Lycopod spores, I. General spore patterns and the generic segregates of Lycopodium. American fern journal, 62(3), 65-79.

Willis, K (2019). Cadia ellisiana (J M McWhirter 112). Digitised palynological slide. In: African Pollen Reference Collection (Version 5, published 10/17/2019). Original material located at Oxford Long-Term Ecology Laboratory. Retrieved from globalpollenproject.org on 2025-07-20.

Willis, K (2019). Calycobolus africanus (GPP96). Digitised palynological slide. In: African Pollen Reference Collection (Version 5, published 10/17/2019). Original material located at Oxford Long-Term Ecology Laboratory. Retrieved from globalpollenproject.org on 2025-07-16.

Willis, K (2019). Chytranthus macrobotrys (GPP387). Digitised palynological slide. In: African Pollen Reference Collection (Version 5, published 10/17/2019). Original material located at Oxford Long-Term Ecology Laboratory. Retrieved from globalpollenproject.org on 2025-07-15.

Willis, K (2019). Coriaria myrtifolia (93.1.1 - 1). Digitised palynological slide. In: European Reference Collection (Version 5, published 10/17/2019). Original material located at Oxford Long-Term Ecology Laboratory. Retrieved from globalpollenproject.org on 2025-06-07.

Willis, K (2019). Crossopteryx febrifuga (GPP351). Digitised palynological slide. In: African Pollen Reference Collection (Version 5, published 10/17/2019). Original material located at Oxford Long-Term Ecology Laboratory. Retrieved from globalpollenproject.org on 2025-07-20.

Willis, K (2019). Dobera (GPP383). Digitised palynological slide. In: African Pollen Reference Collection (Version 5, published 10/17/2019). Original material located at Oxford Long-Term Ecology Laboratory. Retrieved from globalpollenproject.org on 2025-07-15.

Willis, K (2019). Ehretia cymosa (GPP53). Digitised palynological slide. In: African Pollen Reference Collection (Version 5, published 10/17/2019). Original material located at Oxford Long-Term Ecology Laboratory. Retrieved from globalpollenproject.org on 2025-06-05

Willis, K (2019). Funtumia elastica (GPP27). Digitised palynological slide. In: African Pollen Reference Collection (Version 5, published 10/17/2019). Original material located at Oxford Long-Term Ecology Laboratory. Retrieved from globalpollenproject.org on 2025-07-17.

Willis, K (2019). Ganophyllum giganteum (GPP391). Digitised palynological slide. In: African Pollen Reference Collection (Version 5, published 10/17/2019). Original material located at Oxford Long-Term Ecology Laboratory. Retrieved from globalpollenproject.org on 2025-07-15.

Willis, K (2019). Gomphia elongata (GPP287). Digitised palynological slide. In: African Pollen Reference Collection (Version 5, published 10/17/2019). Original material located at Oxford Long-Term Ecology Laboratory. Retrieved from globalpollenproject.org on 2025-07-15.

Willis, K (2019). Hottonia palustris (135.6.1 - Thrum). Digitised palynological slide. In: European Reference Collection (Version 5, published 10/17/2019). Original material located at Oxford Long-Term Ecology Laboratory. Retrieved from globalpollenproject.org on 2025-07-15.

Willis, K (2019). Melochia melissifolia (GPP417). Digitised palynological slide. In: African Pollen Reference Collection (Version 5, published 10/17/2019). Original material located at Oxford Long-Term Ecology Laboratory. Retrieved from globalpollenproject.org on 2025-07-15.

Willis, K (2019). Nesogordonia papaverifera (GPP418). Digitised palynological slide. In: African Pollen Reference Collection (Version 5, published 10/17/2019). Original material located at Oxford Long-Term Ecology Laboratory. Retrieved from globalpollenproject.org on 2025-07-15

Willis, K (2019). Nuxia oppositifolia (GPP250). Digitised palynological slide. In: African Pollen Reference Collection (Version 5, published 10/17/2019). Original material located at Oxford Long-Term Ecology Laboratory. Retrieved from globalpollenproject.org on 2025-07-20

Willis, K (2019). Nuxia oppositifolia (GPP250). Digitised palynological slide. In: African Pollen Reference Collection (Version 5, published 10/17/2019). Original material located at Oxford Long-Term Ecology Laboratory. Retrieved from globalpollenproject.org on 2025-07-21

Willis, K (2019). Nuxia oppositifolia (GPP250). Digitised palynological slide. In: African Pollen Reference Collection (Version 5, published 10/17/2019). Original material located at Oxford Long-Term Ecology Laboratory. Retrieved from globalpollenproject.org on 2025-07-22

Willis, K (2019). Pentanisia ouranogyne (NMK 54). Digitised palynological slide. In: African Pollen Reference Collection (Version 5, published 10/17/2019). Original material located at Oxford Long-Term Ecology Laboratory. Retrieved from globalpollenproject.org on 2025-07-20.

Willis, K (2019). Quassia africana (Menga 106). Digitised palynological slide. In: African Pollen Reference Collection (Version 5, published 10/17/2019). Original material located at Oxford Long-Term Ecology Laboratory. Retrieved from globalpollenproject.org on 2025-07-17.

Willis, K (2019). Sabicea calycina (GPP372). Digitised palynological slide. In: African Pollen Reference Collection (Version 5, published 10/17/2019). Original material located at Oxford Long-Term Ecology Laboratory. Retrieved from globalpollenproject.org on 2025-07-20.

Willis, K (2019). Sterculia tragacantha (GPP420). Digitised palynological slide. In: African Pollen Reference Collection (Version 5, published 10/17/2019). Original material located at Oxford Long-Term Ecology Laboratory. Retrieved from globalpollenproject.org on 2025-07-15.

Willis, K (2019). Strychnos spinosa (Fernando 1444). Digitised palynological slide. In: African Pollen Reference Collection (Version 5, published 10/17/2019). Original material located at Oxford Long-Term Ecology Laboratory. Retrieved from globalpollenproject.org on 2025-06-09.

Willis, K (2019). Thomandersia hensii (GPP9). Digitised palynological slide. In: African Pollen Reference Collection (Version 5, published 10/17/2019). Original material located at Oxford Long-Term Ecology Laboratory. Retrieved from globalpollenproject.org on 2025-07-17.

Willis, K (2019). Triplochiton scleroxylon (GPP421). Digitised palynological slide. In: African Pollen Reference Collection (Version 5, published 10/17/2019). Original material located at Oxford Long-Term Ecology Laboratory. Retrieved from globalpollenproject.org on 2025-07-15.

Willis, K (2019). Vepris lanceolata (GPP381). Digitised palynological slide. In: African Pollen Reference Collection (Version 5, published 10/17/2019). Original material located at Oxford Long-Term Ecology Laboratory. Retrieved from globalpollenproject.org on 2025-07-15.

Willis, K., & Froyd, C. (2018). Phaseolus mollis (GPP119). Digitised palynological slide. In Galapagos Islands Modern Reference Material (Version 2, published 04/30/2018). Original material located at Oxford Long-Term Ecology Laboratory.

Willis, K; Brncic, T (2019). Strombosia grandifolia (GPP298). Digitised palynological slide. In: African Pollen Reference Collection (Version 5, published 10/17/2019). Original material located at Oxford Long-Term Ecology Laboratory. Retrieved from globalpollenproject.org on 2025-07-15.

Willis, K; Duffin, K (2019). Schrebera (GPP300). Digitised palynological slide. In: African Pollen Reference Collection (Version 5, published 10/17/2019). Original material located at Oxford Long-Term Ecology Laboratory. Retrieved from globalpollenproject.org on 2025-07-17.

Woutersen, A., Jardine, P. E., Bogotá-Angel, R. G., Zhang, H. X., Silvestro, D., Antonelli, A., ... & Hoorn, C. (2018). A novel approach to study the morphology and chemistry of pollen in a phylogenetic context, applied to the halophytic taxon Nitraria L.(Nitrariaceae). PeerJ, 6, e5055.

Wu, H. X., Schoch, C. L., Boonmee, S., Bahkali, A. H., Chomnunti, P., & Hyde, K. D. (2011). A reappraisal of Microthyriaceae. Fungal Diversity, 51(1), 189-248.

Xu, Z., Deng, M. (2017). Osmundaceae. In: Identification and Control of Common Weeds: Volume 2. Springer, Dordrecht. https://doi.org/10.1007/978-94-024-1157-7_3

Yan, Y., Fan, Y., Chen, X., Li, L., Warren, A., Al-Farraj, S. A., & Song, W. (2016). Taxonomy and phylogeny of three heterotrich ciliates (Protozoa, Ciliophora), with description of a new Blepharisma species. Zoological Journal of the Linnean Society, 177(2), 320-334.

Yang, J., Liu, L. L., Jones, E. G., Hyde, K. D., Liu, Z. Y., Bao, D. F., ... & Liu, J. K. (2023). Freshwater fungi from karst landscapes in China and Thailand. Fungal Diversity, 119(1), 1-212.

Yang, S., Mao, L., Zheng, Z., Chen, B., & Li, J. (2020). Pollen atlas for selected subfamilies of Euphorbiaceae from Southern China: a complementary contribution to Quaternary pollen analysis. Palynology, 44(4), 659-673.

Ybert, J. P. (1979). Atlas de pollens de Côte d'Ivoire (No. 40). IRD Editions.

Yurtseva, O. V., Severova, E. E., & Bovina, I. Y. (2014). Pollen morphology and taxonomy of Atraphaxis (Polygoneae, Polygonaceae). Plant Systematics and Evolution, 300, 749-766.

Zhou, X. M., & Zhang, L. B. (2015). A classification of Selaginella (Selaginellaceae) based on molecular (chloroplast and nuclear), macromorphological, and spore features. Taxon, 64(6), 1117-1140.

Zonneveld, K. A. (1997). New species of organic walled dinoflagellate cysts from modern sediments of the Arabian Sea (Indian Ocean). Review of Palaeobotany and Palynology, 97(3-4), 319-337.

Zonneveld, K. A., & Jurkschat, T. (1999). Bitectatodinium spongium (Zonneveld, 1997) Zonneveld et Jurkschat, comb. nov. from modern sediments and sediment trap samples of the Arabian Sea (northwestern Indian Ocean): taxonomy and ecological affinity. Review of Palaeobotany and Palynology, 106(3-4), 153-169.

Zonneveld, K. A., & Susek, E. (2007). Effects of temperature, light and salinity on cyst production and morphology of Tuberculodinium vancampoae (the resting cyst of Pyrophacus steinii). Review of Palaeobotany and Palynology, 145(1-2), 77-88.

Zonneveld, K.A.F. and Pospelova V. (2015). A determination key for modern dinoflagellate cysts. Palynology 39 (3), 387- 407.

5. References: classification of growth forms

Aduku, O., Abdullahi, S., Sule, M., Atiku, I., & Anyam, J. (2020). Isolation of taraxasterol and stigmasterol from the aerial part of Centaurea perrottetii DC. (Asteraceae). Bima Journal of Science and Technology, 4(2), 86–93.

Alturki, T., & Thomas, J. (2010). An account on the floral dimorphism and ecology of the genus Moltkiopsis I. M. Johnst. (Boraginaceae) in Saudi Arabia. Turkish Journal of Botany, 34(5), 367-377.

Aradhya, M. K., Potter, D. A. N. I. E. L., & Simon, C. J. (2004, November). Origin, evolution, and biogeography of Juglans: a phylogenetic perspective. In V International Walnut Symposium 705 (pp. 85-94).

Arana, M. D., & Mynssen, C. M. (2015). Cystopteris (Cystopteridaceae) del cono sur y Brasil. Darwiniana, nueva serie, 3(1), 73-88.

Arrington, J. M., & Kubitzki, K. (2003). Cistaceae. In Flowering Plants· Dicotyledons: Malvales, Capparales and Non-betalain Caryophyllales (pp. 62-70). Berlin, Heidelberg: Springer Berlin Heidelberg.

Backlund, M., & Thulin, M. (2007). Revision of the Mediterranean species of Plocama (Rubiaceae). Taxon, 56(2), 516-520.

Baker, W.J., Barfod, A.S., Cámara-Leret, R., Dowe, J.L., Heatubun, C.D., Petoe, P., Turner, J.H., Zona, S. & Dransfield, J. (2024) Palms of New Guinea. Royal Botanic Gardens, Kew, Richmond. 726 pp.

Balakumbahan, R., Rajamani, K., & Kumanan, K. (2010). Acorus calamus: An overview. Journal of Medicinal Plants Research, 4(25), 2740-2745.

Bamps, P., Robson, N., & Verdcourt, B. (1978). Flora of tropical East Africa. Guttiferae (pp. 35-pp).

Barbosa-Silva, R. G., Coutinho, T. S., Vasconcelos, S., da Silva, D. F., Oliveira, G., & Zappi, D. C. (2021). Preliminary placement and new records of an overlooked Amazonian tree, Christiana mennegae (Malvaceae). PeerJ, 9, e12244.

Barres, L., Sanmartín, I., Anderson, C. L., Susanna, A., Buerki, S., Galbany-Casals, M., & Vilatersana, R. (2013). Reconstructing the evolution and biogeographic history of tribe Cardueae (Compositae). American Journal of Botany, 100(5), 867-882.

Beentje, H. (2006). Ericaceae. In Flora of Tropical East Africa (published under the authority of the Secretary of State for the Colonies). Royal Botanic Gardens, Kew. ISBN: 9781842461440, 1842461443.

Beentje, H. (2008). Hymenophyllaceae. In Flora of Tropical East Africa. Royal Botanic Gardens, Kew.

Beentje, H. J., Jeffrey, C., & Hind, D. J. N. (2005). Flora of Tropical East Africa. Compositae (Part 3).

Beentje, H., & Beentje, H. (1989). Flora of Tropical East Africa-Bombacaceae (1989) (Vol. 27). CRC Press.

Beentje, H., Jeffrey, C., & Hind, D. J. N. (2005). Compositae. In Flora of Tropical East Africa. Royal Botanic Gardens, Kew.

Bentvelzen, P. A. J. (1960). Primulaceae. Flora Malesiana-Series 1, Spermatophyta, 6(1), 173-192.

Berg, C. C. (1991). Moraceae. In Flora Zambesiaca (Vol. 9, Part 6). Royal Botanic Gardens, Kew.

Berg, C. C., & Hijman, M. E. (1989). Flora of Tropical East Africa-Moraceae (1989).

Beveridge, F. C., Kalaipandian, S., Yang, C., & Adkins, S. W. (2022). Fruit biology of coconut (Cocos nucifera L.). Plants, 11(23), 3293.

Beyschlag, W. (2024). Bryophytes. In: Büdel, B., Friedl, T., Beyschlag, W. (eds) Biology of Algae, Lichens and Bryophytes. Springer Spektrum, Berlin, Heidelberg.

Biasuso, A. B. (2007). The genus Hedwigia (Hedwigiaceae, Bryophyta) in Argentina. Lindbergia, 5-17.

Bidgood, S., Verdcourt, B., & Vollesen, K. (2006). Bignoniaceae (Vol. 241, Flora of Tropical East Africa, Angiospermae). Published on behalf of the East African governments by Royal Botanic Gardens, Kew. ISBN: 1842461516, 9781842461518.

Bissiengou, P. (2014). Systematics, evolution and historical biogeography of the family Ochnaceae with emphasis on the genus Campylospermum. Wageningen University and Research.

Borosova, R., Utteridge, T. M. A., & Schuiteman, A. (2021). Taxonomy and morphology of Thalictrum (Ranunculaceae) in New Guinea. Kew Bulletin, 76, 805-817. https://doi.org/10.1007/s12225-021-09972-1

Boutique, R., & Verdcourt, B. (1973). Haloragaceae. In Flora of Tropical East Africa. Balkema.

Bramley, G., Trias-Blasi, A., & Wilford, R. (2023). The Kew Temperate Plant Families Identification Handbook. Royal Botanic Gardens, Kew.

Bridson, D. (1998). Rubiaceae. In Flora Zambesiaca (Vol. 5, Part 2). Royal Botanic Gardens, Kew.

Bridson, D. M. (1986). The reinstatement of the African genus Keetia (Rubiaceae subfam. Cinchonoideae, tribe Vanguerieae). Kew bulletin, 965-994.

Bridson, D. M., & Verdcourt, B. (2003). Flora Zambesiaca 5 (3): Rubiaceae, Part 3. royal Botanic Gardens.

Bridson, D. M., & Verdcourt, B. (2003). Rubiaceae. In Flora Zambesiaca (Vol. 5, Part 3). Royal Botanic Gardens, Kew.

Brock, T. C., Mielo, H., & Oostermeijer, G. (1989). On the life cycle and germination of Hottonia palustris L. in a wetland forest. Aquatic Botany, 35(2), 153-166.

Brummitt, R. K., Chikuni, A. C., Lock, J. M., & Polhill, R. M. (2007). Leguminosae, subfamily Caesalpinioideae. Flora Zambesiaca, 3(part 2), 1-228.

Bruza, J. D. (1982). A revision of the Diodia teres complex (Rubiaceae). Mississippi State University.

Butcher, R. W. (1947). Atropa Belladonna L. Journal of Ecology, 34(2), 345-353.

Carbone, K., & Gervasi, F. (2022). An updated review of the genus Humulus: a valuable source of bioactive compounds for health and disease prevention. Plants, 11(24), 3434.

Carlquist, S. (1970). Wood anatomy of Echium (Boraginaceae). Aliso: A Journal of Systematic and Floristic Botany, 7(2), 183-199.

Carta, A., Savio, L., Bedini, G., Peruzzi, L., Fisogni, A., & Galloni, M. (2016). All in an afternoon: mixed breeding system in one-day lasting flowers of Hypericum elodes L.(Hypericaceae). Plant Biosystems-An International Journal Dealing with all Aspects of Plant Biology, 150(5), 1001-1009.

Castroviejo, S., Aedo, C., Cirujano, S., Laínz, M., Montserrat, P., Morales, R., Muñoz Garmendia, F., Navarro, C., Paiva, J., & Soriano, C. (Eds.). (1993). Flora iberica (Vol. 4). Real Jardín Botánico, CSIC.

Castroviejo, S., Aedo, C., Cirujano, S., Laínz, M., Montserrat, P., Morales, R., Muñoz Garmendia, F., Navarro, C., Paiva, J., & Soriano, C. (Eds.). (1998). Flora iberica (Vol. 6). Real Jardín Botánico, CSIC.

Castroviejo, S., Aedo, C., Cirujano, S., Laínz, M., Montserrat, P., Morales, R., Muñoz Garmendia, F., Navarro, C., Paiva, J., & Soriano, C. (Eds.). (2001). Flora iberica (Vol. 14). Real Jardín Botánico, CSIC.

Castroviejo, S., Aedo, C., Cirujano, S., Laínz, M., Montserrat, P., Morales, R., Muñoz Garmendia, F., Navarro, C., Paiva, J., & Soriano, C. (Eds.). (2012). Flora iberica (Vol. 11). Real Jardín Botánico, CSIC.

Castroviejo, S., Aedo, C., Cirujano, S., Laínz, M., Montserrat, P., Morales, R., Muñoz Garmendia, F., Navarro, C., Paiva, J., & Soriano, C. (Eds.). (2015). Flora iberica (Vol. 9). Real Jardín Botánico, CSIC.

Cheek, M., & Dorr, L. J. (2007). Sterculiaceae. Flora of Tropical East Africa. Royal Botanic Gardens, Kew.

Christenhusz, M. J., Chase, M. W., Fay, M. F., Hidalgo, O., Leitch, I. J., Pellicer, J., & Viruel, J. (2021). Biogeography and genome size evolution of the oldest extant vascular plant genus, Equisetum (Equisetaceae). Annals of Botany, 127(5), 681-695.

Chung, K. S. (2008). A systematic study of genus Agrimonia (Rosaceae). The University of Oklahoma.

Coimbra, A. T., Ferreira, S., & Duarte, A. P. (2020). Genus Ruta: A natural source of high value products with biological and pharmacological properties. Journal of ethnopharmacology, 260, 113076.

Cook, C. D., Gut, B. J., Rix, E. M., & Schneller, J. (1974). Water plants of the world: a manual for the identification of the genera of freshwater macrophytes. Springer Science & Business Media.

Crowder, A. A., Pearson, M. C., Grubb, P. J., & Langlois, P. H. (1990). Drosera L. Journal of Ecology, 78(1), 233-267.

Cufodontis, G. (1966). Pittosporaceae. In Flora of Tropical East Africa. A.A. Balkema.

Cusma-Velari, T., & Feoli-Chiapella, L. (2009). The so-called primitive genera of Genisteae (Fabaceae): systematic and phyletic considerations based on karyological data. Botanical Journal of the Linnean Society, 160(2), 232-248.

Dafni, A., Shmida, A., & Avishai, M. (1981). Leafless autumnal-flowering geophytes in the Mediterranean region—phytogeographical, ecological and evolutionary aspects. Plant Systematics and Evolution, 137(3), 181-193.

Dahl, Å. E. (1990). Infrageneric division of the genus Hypecoum (Papaveraceae). Nordic journal of botany, 10(2), 129-140.

Dahlgren, R. M., Clifford, H. T., & Yeo, P. F. (2012). The families of the monocotyledons: structure, evolution, and taxonomy. Springer Science & Business Media. Chicago

Dai, X., Li, X., Song, X., Li, X., & Liu, X. (2021). The evolutionary history and phylogeographic pattern of Hippuris vulgaris: hybridization and long-distance dispersal from China. Plant Systematics and Evolution, 307, 1-8.

Damesin, C., Rambal, S., & Joffre, R. (1998). Co-occurrence of trees with different leaf habit: a functional approach on Mediterranean oaks. Acta Oecologica, 19(3), 195-204.

Darbyshire, I., Vollesen, K., & Kelbessa, E. (s.f.). Acanthaceae (Part 2). In Flora Zambesiaca. Royal Botanic Gardens, Kew.

Dauphin, B., Farrar, D. R., Maccagni, A., & Grant, J. R. (2017). A worldwide molecular phylogeny provides new insight on cryptic diversity within the moonworts (Botrychium ss, Ophioglossaceae). Systematic Botany, 42(4), 620-639.

Davidson, C. (1973). An anatomical and morphological study of Datiscaceae. Aliso: A Journal of Systematic and Floristic Botany, 8(1), 49-110.

Davies, F. G., & Verdcourt, B. (1998). Flora of Tropical East Africa-Sapindaceae. CRC Press.

De Benedetti, C., Gerasimenko, N., Ravazzi, C., & Magri, D. (2022). History of Tilia in Europe since the Eemian: Past distribution patterns. Review of Palaeobotany and Palynology, 307, 104778.

de Loewenstern, A. B., & Garbari, F. (2002). Karyological aspects of the genus Neurada L.(Neuradaceae JG Agardh). Caryologia, 55(4), 361-365.

De Ruiter, G. (1976). Revision of the genera Myrianthus and Musanga (Moraceae). Bulletin du Jardin botanique national de Belgique/Bulletin van de Nationale Plantentuin van Belgie, 471-510.

Decraene, L. R. (1989). The flower of Koenigia islandica L.(Polygonaceae): an interpretation. Watsonia., 17, 419.

Deng, T., Zhang, J. W., Meng, Y., Volis, S., Sun, H., & Nie, Z. L. (2017). Role of the Qinghai-Tibetan Plateau uplift in the Northern Hemisphere disjunction: evidence from two herbaceous genera of Rubiaceae. Scientific Reports, 7(1), 13411.

DeSilva, R., & Dodd, R. S. (2021). Patterns of fine-scale spatial genetic structure and pollen dispersal in giant sequoia (Sequoiadendron giganteum). Forests, 12(1), 61.

Dransfield, J. (1994). Palmae. In Flora of Tropical East Africa. A. A. Balkema.

Dubois, J. J. B., & Blazich, F. A. (2008). Lonicera L. The Woody Plant Seed Manual. US Department of Agriculture, Forest Service, Washington DC, 682-688.

Durrant, T. H., De Rigo, D., & Caudullo, G. (2016). Pinus sylvestris in Europe: distribution, habitat, usage and threats. European atlas of forest tree species, 14, 845-846.

Durrant, T. H., De Rigo, D., & Caudullo, G. (2016). Quercus suber in Europe: distribution, habitat, usage and threats. Eur. Atlas For. Tree Species, 164-165.

Ekalu, A. (2021). Medicinal uses, phytochemistry, and pharmacological activities of Mitracarpus species (Rubiaceae): A review. Scientific African, 11, e00692.

El Ghazali, G. E. B. (1993). A Study on the Pollen Flora of Sudan. Review of Palaeobotany and Palynology, 76, 99-345.

Enright, N. J., & Jaffré, T. (2011). Ecology and distribution of the Malesian podocarps. Smithsonian contributions to botany, 95, 57-78.

Erbar, C., & Leins, P. (2004). Callitrichaceae. In Flowering Plants· Dicotyledons: Lamiales (except Acanthaceae including Avicenniaceae) (pp. 50-56). Berlin, Heidelberg: Springer Berlin Heidelberg.

Ewédjè, E. E. B. K., Jansen, S., Koffi, G. K., Staquet, A., Piñeiro, R., Essaba, R. A., ... & Hardy, O. J. (2020). Species delimitation in the African tree genus Lophira (Ochnaceae) reveals cryptic genetic variation. Conservation genetics, 21, 501-514.

Fan, J., Fu, Q. C., & Liang, Z. (2019). Complete chloroplast genome sequence and phylogenetic analysis of Sinojackia sarcocarpa, an endemic plant in Southwest China. Mitochondrial DNA Part B, 4(1), 1350-1351.

Fang, J., & Lechowicz, M. J. (2006). Climatic limits for the present distribution of beech (Fagus L.) species in the world. Journal of Biogeography, 33(10), 1804-1819.

Farag, S., & Kayser, O. (2017). The Cannabis Plant: Botanical Aspects. In Handbook of Cannabis and Related Pathologies: Biology, Pharmacology, Diagnosis, and Treatment (pp. 3–12). https://doi.org/10.1016/B978-0-12-800756-3.00001-6

Feng, X. L., Yu, Y., Qin, D. P., Gao, H., & Yao, X. S. (2015). Acorus Linnaeus: a review of traditional uses, phytochemistry and neuropharmacology. RSC advances, 5(7), 5173-5182.

Fernandes, A. (1978). Lythraceae. In Flora Zambesiaca (Vol. 4). Royal Botanic Gardens, Kew.

Fernandes, R., & Fernandes, A. (1966). Anacardiaceae. In Flora Zambesiaca (Vol. 2, Part 2). Royal Botanic Gardens, Kew.

Ferreira, R. C., Piredda, R., Bagnoli, F., Bellarosa, R., Attimonelli, M., Fineschi, S., ... & Simeone, M. C. (2011). Phylogeography and conservation perspectives of an endangered Macaronesian endemic: Picconia azorica (Tutin) Knobl.(Oleaceae). European Journal of Forest Research, 130(2), 181-195.

Friis, I. (1991). Urticaceae Flora Zambesiaca 9 (6). Includes a picture, 81-83.

Funk, V., Susanna, A., Stuessy, T., & Bayer, R. (2009). Systematics, Evolution, and Biogeography of Compositae. Vienna: International Association for Plant Taxonomy. ISBN: 978-3-9501754-3-1.

García, M. A., Costea, M., Kuzmina, M., & Stefanović, S. (2014). Phylogeny, character evolution, and biogeography of Cuscuta (dodders; Convolvulaceae) inferred from coding plastid and nuclear sequences. American journal of botany, 101(4), 670-690.

Garg, S., Anvar Hussain, N. A., Syed, I., Asaithambi, N., & Mundhada, S. (2020). Water Chestnut (Trapa natans). Antioxidants in Vegetables and Nuts-Properties and Health Benefits, 453-465.

Ghazanfar, S. A., Edmondson, J. R. & Hind, D. J. N. (Eds). (2019). Flora of Iraq, Volume 6: Compositae. Kew Publishing

Ghazanfar, S. A., Edmondson, J. R. (Eds). (2013). Flora of Iraq, Volume 5, Part 2: Lythraceae to Campanulaceae. Kew Publishing

Ghazanfar, S. A., Edmondson, J. R. (Eds). (2016). Flora of Iraq, Volume 5, Part 1: Elatinaceae to Sphenocleaceae. Kew Publishing

Ghazanfar, S. A., Raven, P. H., Townsend, C. C., Taylor, P., & Mobayen, S. (2013). Flora of Iraq (Vol. 5, Part 2, pp. xi, 1-349). In S. A. Ghazanfar & J. R. Edmondson (Eds.), Kew, UK: Royal Botanic Gardens, Kew. Published on behalf of the Ministry of Agriculture, Republic of Iraq by Royal Botanic Gardens, Kew. ISBN 978-1-84246-493-9.

Giacosa, J. R., Morbelli, M., & Giudice, G. (2004). Spore wall ultrastructure in Anogramma species (Pteridaceae) from Argentina. Grana, 43(4), 231-237.

Gimingham, C. H., & Birse, E. M. (1957). Ecological studies on growth-form in bryophytes: I. Correlations between growth-form and habitat. The Journal of Ecology, 533-545.

Goyder, D. J., Gilbert, M. G. & Venter, H. J. T. (2020). Apocynaceae (part 2). In: M. A. García (ed.), Flora Zambesiaca, Vol. 7(3). Royal Botanic Gardens, Kew.

Goyder, D., Harris, T., Masinde, S., Meve, U., & Venter, J. (2012). Apocynaceae (Part 2). In Flora of Tropical East Africa. Royal Botanic Gardens, Kew.

Graham, S. A. (1985). A revision of Ammannia (Lythraceae) in the Western Hemisphere. Journal of the Arnold Arboretum, 66(4), 395-420.

Grey-Wilson, C. (1980). Impatiens of Africa. CrC Press.

Grimm, G. W., & Denk, T. (2008). ITS evolution in Platanus (Platanaceae): homoeologues, pseudogenes and ancient hybridization. Annals of Botany, 101(3), 403-419.

Grímsson, F., Ulrich, S., Coiro, M., Graham, S. A., Jacobs, B. F., Currano, E. D., ... & Zetter, R. (2021). Hagenia from the early Miocene of Ethiopia: Evidence for possible niche evolution?. Ecology and Evolution, 11(10), 5164-5186.

Gustafsson, C., & Persson, C. (2002). Phylogenetic relationships among species of the neotropical genus Randia (Rubiaceae, Gardenieae) inferred from molecular and morphological data. Taxon, 51(4), 661-674.

Guzmán, B., Fedriani, J. M., Delibes, M., & Vargas, P. (2017). The colonization history of the Mediterranean dwarf palm (Chamaerops humilis L., Palmae). Tree Genetics & Genomes, 13, 1-10.

Hadidi, M. N. (1985). Flora of tropical East Africa-Zygophyllaceae (1985) (No. 95). CRC Press.

Happi, G. M., Tiani, G. L. M., Gbetnkom, B. Y. M., Hussain, H., Green, I. R., Ngadjui, B. T., & Kouam, S. F. (2020). Phytochemistry and pharmacology of Harungana madagascariensis: mini review. Phytochemistry Letters, 35, 103-112.

Harris, S. A., Maberly, S. C., & Abbott, R. J. (1992). Genetic variation within and between populations of Myriophyllum alterniflorum DC. Aquatic botany, 44(1), 1-21.

Hawksworth, F. G., & Wiens, D. (1972). Biology and classification of dwarf mistletoes (Arceuthobium) (No. 401). US Forest Service.

Hayden, W. J., Simmons, M. P., & Swanson, L. J. (1993). Wood anatomy of Amanoa (Euphorbiaceae). IAWA Journal, 14(2), 205-213.

He, X. Y., Chen, J. M., & Li, Z. Z. (2024). Complete organelle genomes of the threatened aquatic species Scheuchzeria palustris (Scheuchzeriaceae): Insights into adaptation and phylogenomic placement. Ecology and Evolution, 14(9), e70248.

He-Nygrén, X., Juslén, A., Ahonen, I., Glenny, D., & Piippo, S. (2006). Illuminating the evolutionary history of liverworts (Marchantiophyta)—towards a natural classification. Cladistics, 22(1), 1-31.

Hearn, D. J. (2006). Adenia (Passifloraceae) and its adaptive radiation: phylogeny and growth form diversification. Systematic Botany, 31(4), 805-821.

Heine, H. (1963). The Genus Calycobolus Willd. ex. Roem. & Schultes (Convolvulaceae) in Africa. Kew Bulletin, 16(3), 387-391.

Hepper, F. N. (1972). Numerical Analysis of the'Flora of West Tropical Africa': II: Angiosperms (Monocotyledons). Kew Bulletin, 305-307.

Hepper, F. N. (1973). Lemnaceae. In Flora of Tropical East Africa. Crown Agents for Oversea Governments and Administrations.

Hepper, F. N., & Keay, R. W. J. (1963). Rubiaceae. In Flora of West Tropical Africa (Vol. 2). Crown Agents for Oversea Governments and Administrations.

Heuvel, B. D. V. (2011). Alnus. In Wild Crop Relatives: Genomic and Breeding Resources: Forest Trees (pp. 1-14). Berlin, Heidelberg: Springer Berlin Heidelberg.

Hildebrand, T. J. (2005). Sectional revision, speciation, and population biology in lycopodium (lycopodiaceae) (Order No. 3250052). Available from ProQuest Dissertations & Theses Global. (304989785). Retrieved from https://liverpool.idm.oclc.org/login?url=https://www.proquest.com/dissertations-theses/sectional-revision-speciation-population-biology/docview/304989785/se-2

Hoggard, R. K., Kores, P. J., Molvray, M., Hoggard, G. D., & Broughton, D. A. (2003). Molecular systematics and biogeography of the amphibious genus Littorella (Plantaginaceae). American Journal of Botany, 90(3), 429-435.

Huang, Y. J., Liu, Y. S., Wen, J., & Quan, C. (2015). First fossil record of Staphylea L.(Staphyleaceae) from North America, and its biogeographic implications. Plant Systematics and Evolution, 301(9), 2203-2218.

Hutchinson, J. and Dalziel, J.M. (1958) Flora of West Tropical Africa. In: Keay, R.W.J., Ed., 2nd Edition, Vol. 1. Part 2, Published on Behalf of the Governments of Nigeria, Ghana, Sierra Leone & The Gambia by Crown Agents for Overseas Governments and Administrations, Millbank, London.

Hutchinson, J., & Dalziel, J. M. (1952). Flora of West Tropical Africa (2nd ed., Vol. 1). Revised by R. W. J. Keay. Revision edited by F. N. Hepper. Published on behalf of the Governments of Nigeria, the Gold Coast, Sierra Leone, and The Gambia by Crown Agents for Overseas Governments and Administrations. Millbank, London.

Hutchinson, J., & Dalziel, J. M. (1954). Flora of West Tropical Africa.

Hutchinson, J., & Dalziel, J. M. (1954). Lecythidaceae. In Flora of West Tropical Africa (Vol. 1, Part 1). Crown Agents for Oversea Governments and Administrations.

Hutchinson, J., & Dalziel, J. M. (1958). Euphorbiaceae. In Flora of West Tropical Africa (Vol. 1, Part 2). Crown Agents for Oversea Governments and Administrations.

Hutchinson, J., & Dalziel, J. M. (1958). Flora of West Tropical Africa. In R. W. J. Keay (Ed.), 2nd ed. (Vol. 1, Part 2). Published on behalf of the Governments of Nigeria, Ghana, Sierra Leone, & The Gambia by Crown Agents for Overseas Governments and Administrations. Millbank, London.

Hutchinson, J., & Dalziel, J. M. (1958). Olacaceae. In Flora of West Tropical Africa (Vol. 1, Part 2). Crown Agents for Oversea Governments and Administrations.

Hutchinson, J., & Dalziel, J. M. (1958). Pandaceae. In Flora of West Tropical Africa (Vol. 1, Part 2). Crown Agents for Oversea Governments and Administrations.

Ithnin, M., Serdari, N. M., Abdullah, N., Kushairi, A., & Singh, R. (2017). Biodiversity and conservation of Elaeis species. Biodiversity and Conservation of Woody Plants, 245-272.

Itokawa, H., & Lee, K. H. (Eds.). (2003). Taxus: the genus Taxus. CRC Press.

Jacobs, M. (1964). The genus Crateva (Capparaceae). Blumea: Biodiversity, Evolution and Biogeography of Plants, 12(2), 177-208.

Jermy, A. C. (1990). Isoetaceae. In Pteridophytes and gymnosperms (pp. 26-31). Berlin, Heidelberg: Springer Berlin Heidelberg.

Johnson, O. O., Adeyemi, D. K., Abiodun, G., Ayoola, S. G. B., & Madayath, H. (2022), The phytochemistry and pharmacological potentials of Flabellaria paniculata cav.(malpighiaceae): A review of an unexplored medicinal plant. International Journal of Pharmacognosy and Life Science.

Kadereit, J. W. (1987). The taxonomy, distribution and variability of the genus Roemeria Medic.(Papaveraceae). Flora, 179(2), 135-153.

Kadereit, J. W., Schwarzbach, A. E., & Jork, K. B. (1997). The phylogeny of Papaver sl (Papaveraceae): polyphyly or monophyly?. Plant Systematics and Evolution, 204, 75-98.

Kamel, S., Ayed, S., & Cherif, M. (2010). Identification of Tunisian Barley Lines Tolerant to Both Net Botch and Scald in the Adult Stage. Tunisian Plant Science and Biotechnology II. The African Journal of Plant Science and Biotechnology, 4, 77-80.

Karl, R., & Strid, A. (2009). Bongardia chrysogonum (Berberidaceae) rediscovered on the East Aegean island of Chios. Phytologia Balcanica, 15, 337-342.

Katinas, L., Tellería, M. C., Susanna de la Serna, A., & Ortiz, S. (2008). Warionia (Asteraceae): a relict genus of Cichorieae?.

Kessler, P.J.A. (1993). Annonaceae. In: Kubitzki, K., Rohwer, J.G., Bittrich, V. (eds) Flowering Plants · Dicotyledons. The Families and Genera of Vascular Plants, vol 2. Springer, Berlin, Heidelberg. https://doi.org/10.1007/978-3-662-02899-5_9

Khan, I., Najeebullah, S., Ali, M., & Shinwari, Z. K. (2016). Phytopharmacological and ethnomedicinal uses of the Genus Berberis (Berberidaceae): A review. Tropical Journal of Pharmaceutical Research, 15(9), 2047-2057.

Kilian, N., Gemeinholzer, B., & Lack, H. W. (2009). Cichorieae. Systematics, evolution, and biogeography of Compositae, 343-383.

Kim, H., & Heo, K. (2025). Embryology of Menyanthes (Menyanthaceae): its description and taxonomic implications. Nordic Journal of Botany, e04575.

Kim, K. J. (1998). A new species of Fontanesia (Oleaceae) from China and taxonomic revision of the genus. Journal of Plant Biology, 41, 142-145.

Kim, K. J., & Jansen, R. K. (1998). A chloroplast DNA phylogeny of lilacs (Syringa, Oleaceae): plastome groups show a strong correlation with crossing groups. American Journal of Botany, 85(9), 1338-1351.

Kokwaro, J. O. (1986). Anacardiaceae. In Flora of Tropical East Africa. Royal Botanic Gardens, Kew.

Kokwaro, J. O. (1986). Flora of Tropical East Africa-Valerianaceae. CRC Press.

Korall, P., & Pryer, K. M. (2014). Global biogeography of scaly tree ferns (Cyatheaceae): evidence for Gondwanan vicariance and limited transoceanic dispersal. Journal of biogeography, 41(2), 402-413.

Kozlowski, G., Bétrisey, S., & Song, Y.-G. (2018). Wingnuts (Pterocarya) & walnut family. Relict trees: linking the past, present and future. [ISBN 978-2-9701096-1-7].

Kramer, K. U. (1993). Distribution patterns in major pteridophyte taxa relative to those of angiosperms. Journal of Biogeography, 287-291.

Krapovickas, A., Gregory, W. C., Williams, D. E., & Simpson, C. E. (2007). Taxonomy of the genus Arachis (Leguminosae). Bonplandia, 16, 7-205.

Kubitzki, K. (1993). Betulaceae. In Flowering Plants· Dicotyledons: Magnoliid, Hamamelid and Caryophyllid Families (pp. 152-157). Berlin, Heidelberg: Springer Berlin Heidelberg.

Kubitzki, K. (2003). Frankeniaceae. In Flowering Plants· Dicotyledons: Malvales, Capparales and Non-betalain Caryophyllales (pp. 209-212). Berlin, Heidelberg: Springer Berlin Heidelberg.

Kujawska, M., & Svanberg, I. (2019). From medicinal plant to noxious weed: Bryonia alba L.(Cucurbitaceae) in northern and eastern Europe. Journal of ethnobiology and ethnomedicine, 15, 1-12.

Kunzmann, L., Kvaček, Z., Mai, D. H., & Walther, H. (2009). The genus Taxodium (Cupressaceae) in the Palaeogene and Neogene of Central Europe. Review of Palaeobotany and Palynology, 153(1-2), 153-183.

Kupicha, F. (1983). Flora Zambesiaca (Vol. 7, Part 1). Royal Botanic Gardens.

Kupicha, F. K. (1983). Primulaceae. In Flora Zambesiaca (Vol. 7, Part 1). Royal Botanic Gardens, Kew.

Larridon, I., Tanaka, N., Liang, Y., Phillips, S. M., Barfod, A. S., Cho, S. H., ... & Ito, Y. (2019). First molecular phylogenetic insights into the evolution of Eriocaulon (Eriocaulaceae, Poales). Journal of plant research, 132, 589-600.

Lavender, D. P., & Hermann, R. K. (2014). Douglas-fir: the genus Pseudotsuga.

Lee, N. Y., Khoo, W. K., Adnan, M. A., Mahalingam, T. P., Fernandez, A. R., & Jeevaratnam, K. (2016). The pharmacological potential of Phyllanthus niruri. Journal of pharmacy and pharmacology, 68(8), 953-969.

Leeuwenberg, A. J. M. (1961). The Loganiaceae of Africa I. Anthocleista. Acta Botanica Neerlandica, 10(1), 1-53.

Leeuwenberg, A. J. M., Kupicha, F. K., et al. (1985). Apocynaceae. In Flora Zambesiaca (Vol. 7, Part 2). Royal Botanic Gardens, Kew.

Lehnert, M., Monjau, T., & Rosche, C. (2024). Synopsis of Osmunda (royal ferns; Osmundaceae): towards reconciliation of genetic and biogeographic patterns with morphologic variation. Botanical Journal of the Linnean Society, 205(4), 341-364.

LePage, B. A., & Basinger, J. F. (1995). The evolutionary history of the genus Larix (Pinaceae). Proceedings of an international symposium.

Levin, G. A., Cardinal-McTeague, W. M., Steinmann, V. W., & Sagun, V. G. (2022). Phylogeny, classification, and character evolution of Acalypha (Euphorbiaceae: Acalyphoideae). Systematic Botany, 47(2), 477-497.

Levin, S. A. (2013). Encyclopedia of biodiversity. Academic Press.

Lewis, G., Schrire, B., MacKinder, B., & Lock, M. (Eds.). (2005). Legumes of the world. Royal Botanic Gardens, Kew.

Li, J., Shoup, S., & Chen, Z. (2005). Phylogenetics of Betula (Betulaceae) inferred from sequences of nuclear ribosomal DNA. Rhodora, 107(929), 69-86.

Li, N., Du, C., Ma, B., Gao, Z., Wu, Z., Zheng, L., ... & Wang, Y. (2019). Functional analysis of ion transport properties and salt tolerance mechanisms of RtHKT1 from the recretohalophyte Reaumuria trigyna. Plant and Cell Physiology, 60(1), 85-106.

Li, S., Cheng, X., & Wang, C. (2017). A review on traditional uses, phytochemistry, pharmacology, pharmacokinetics and toxicology of the genus Peganum. Journal of Ethnopharmacology, 203, 127-162.

Li, Y., Li, X., Nie, S., Zhang, M., Yang, Q., Xu, W., ... & Wang, X. (2024). Reticulate evolution of the tertiary relict Osmanthus. The Plant Journal, 117(1), 145-160.

Lidén, M. (1993). Fumariaceae. In Flowering Plants· Dicotyledons: Magnoliid, Hamamelid and Caryophyllid Families (pp. 310-318). Berlin, Heidelberg: Springer Berlin Heidelberg.

Lima, J. B., Bovini, M. G., & Conceição, A. D. S. (2019). Bombacoideae, Byttnerioideae, Grewioideae and Helicterioideae (Malvaceae sl) in the Raso da Catarina Ecoregion, Bahia, Brazil. Biota Neotropica, 19(3), e20180569.

Limarino, T. O., & Borsch, T. (2020). Gomphrena (Amaranthaceae, Gomphrenoideae) diversified as a C4 lineage in the New World tropics with specializations in floral and inflorescence morphology, and an escape to Australia. Willdenowia, 50(3), 345-381.

Linder, H. P., Dlamini, T., Henning, J., & Verboom, G. A. (2006). The evolutionary history of Melianthus (Melianthaceae). American Journal of Botany, 93(7), 1052-1064.

Liu, X. I. N. G., Gituru, W. R., & Wang, Q. F. (2004). Distribution of basic diploid and polyploid species of Isoetes in East Asia. Journal of biogeography, 31(8), 1239-1250.

Liu, Z., Liu, H., Tan, B., Wang, X., & Chong, P. (2025). Physiological and transcriptomic analyses revealed the alleviating effects of exogenous Ca2+ and NO compound treatment on high salt stress in Reaumuria soongorica. BMC genomics, 26(1), 179.

Lock, J. M. (2020). Flora of Tropical East Africa-Xyridaceae (1999). CRC Press.

Lock, M. (2005). Legumes of the World (Vol. 577). G. P. Lewis, B. Schrire, & B. Mackinder (Eds.). Kew: Royal Botanic Gardens.

Lu, L., Wang, W., Chen, Z., & Wen, J. (2013). Phylogeny of the non-monophyletic Cayratia Juss.(Vitaceae) and implications for character evolution and biogeography. Molecular Phylogenetics and Evolution, 68(3), 502-515.

Lucas, G. Ll. (1968). Icacinaceae. In Flora of Tropical East Africa. Royal Botanic Gardens, Kew.

Lucas, G. Ll. (1968). Olacaceae. In Flora of Tropical East Africa. Royal Botanic Gardens, Kew.

Lutz, J. A., Larson, A. J., Furniss, T. J., Donato, D. C., Freund, J. A., Swanson, M. E., ... & Franklin, J. F. (2014). Spatially nonrandom tree mortality and ingrowth maintain equilibrium pattern in an old-growth Pseudotsuga–Tsuga forest. Ecology, 95(8), 2047-2054.

Ma, X. Y., Xu, H., Cao, Z. Y., Shu, L., & Zhu, R. L. (2022). Will climate change cause the global peatland to expand or contract? Evidence from the habitat shift pattern of Sphagnum mosses. Global Change Biology, 28(21), 6419-6432.

Madeira, P. T., Pemberton, R. W., & Center, T. D. (2008). A molecular phylogeny of the genus Lygodium (Schizaeaceae) with special reference to the biological control and host range testing of Lygodium microphyllum. Biological Control, 45(3), 308-318.

Malcomber, S. T. (2002). Phylogeny of Gaertnera Lam.(Rubiaceae) based on multiple DNA markers: evidence of a rapid radiation in a widespread, morphologically diverse genus. Evolution, 56(1), 42-57.

Malmir, M., Serrano, R., Caniça, M., Silva-Lima, B., & Silva, O. (2018). A comprehensive review on the medicinal plants from the genus Asphodelus. Plants, 7(1), 20.

Manchester, S. R., Chen, Z. D., Lu, A. M., & Uemura, K. (2009). Eastern Asian endemic seed plant genera and their paleogeographic history throughout the Northern Hemisphere. Journal of Systematics and Evolution, 47(1), 1-42.

Mannino, A. M., Menéndez, M., Obrador, B., Sfriso, A., & Triest, L. (2015). The genus Ruppia L.(Ruppiaceae) in the Mediterranean region: an overview. Aquatic Botany, 124, 1-9.

Mao, L., Huang, K., & Huang, H. (2024). Introduction to the special issue: Pollen diversity, vegetation history and range shift in the (sub) tropics through the Cenozoic. Review of Palaeobotany and Palynology, 105277.

Martins, L., & Hellwig, F. H. (2005). Phylogenetic relationships of the enigmatic species Serratula chinensis and Serratula forrestii (Asteraceae-Cardueae). Plant Systematics and Evolution, 255(3), 215-224.

Mathew, P., & Biju, S. D. (1991). Lepistemon verdcourtii, a new species of Convolvulaceae from India, with notes on L. binectariferum and L. leiocalyx. Kew bulletin, 559-562.

Mauri, A., Di Leo, M., De Rigo, D., & Caudullo, G. (2016). Pinus halepensis and Pinus brutia in Europe: distribution, habitat, usage and threats. European atlas of forest tree species, 122-123.

McCarthy, B. C., & Quinn, J. A. (1992). Fruit maturation patterns of Carya spp.(Juglandaceae): an intra-crown analysis of growth and reproduction. Oecologia, 91, 30-38.

Meerts, P., Rougelot, Q., & Sosef, M. (2017). Revision of the genus Monotes (Dipterocarpaceae) in DR Congo, with implications for Angola and its distinction from Marquesia. Phytotaxa, 308(2), 151-205.

Melamed, D., Segarra-Moragues, J. G., Puche, F., Garcia, C. A., & Sérgio, C. (2021). On the synonymization of Acaulon longifolium Herrnst. & Heyn with Acaulon fontiquerianum Casas & Sérgio (Pottiaceae). Cryptogamie, Bryologie, 42(18), 239-248.

Mellano, M. G., Beccaro, G. L., Donno, D., et al. (2012). Castanea spp. biodiversity conservation: collection and characterization of the genetic diversity of an endangered species. Genetic Resources and Crop Evolution, 59, 1727–1741. https://doi.org/10.1007/s10722-012-9794-x

Metzgar, J. S., Alverson, E. R., Chen, S., Vaganov, A. V., & Ickert-Bond, S. M. (2013). Diversification and reticulation in the circumboreal fern genus Cryptogramma. Molecular Phylogenetics and Evolution, 67(3), 589-599.

Milliken, W., Klitgaard, B., & Baracat, A. (Eds.). (2010). Neotropikey: Interactive key and information resources for flowering plants of the Neotropics. Retrieved from http://www.kew.org/science/tropamerica/neotropikey.htm

Milliken, W., Klitgard, B. and Baracat, A. (2009 onwards), Neotropikey - Interactive key and information resources for flowering plants of the Neotropics. http://creativecommons.org/licenses/by/3.0

Milne-Redhead, E. (1975). Dioscoreaceae. In Flora of Tropical East Africa. Crown Agents for Oversea Governments and Administrations.

Mo, X., Zhou, Y., Zhan, M., Zhang, Y., Liu, J., Quang, H., & Dong, L. (2025). A review of the traditional uses, phytochemistry, pharmacology and toxicity for the genus Geum (Rosaceae). Fitoterapia, 180, 106333.

Mogue Kamga, S., Brokamp, G., Cosiaux, A., et al. (2020). Use and cultural significance of Raphia palms. Economic Botany, 74, 207-225. https://doi.org/10.1007/s12231-020-09487-z

Molnar, T.J. (2011). Corylus. In: Kole, C. (eds) Wild Crop Relatives: Genomic and Breeding Resources. Springer, Berlin, Heidelberg

Morales, R. (1998). Filipendula Mill. Flora iberica, 6.

Murrell, Z. E. (1993). Phylogenetic relationships in Cornus (cornaceae). Systematic Botany, 469-495.

Mwachala, G., & Mbugua, P. (2007). Dracaenaceae. Flora of Tropical East Africa.

Nagalingum, N. S., Nowak, M. D., & Pryer, K. M. (2008). Assessing phylogenetic relationships in extant heterosporous ferns (Salviniales), with a focus on Pilularia and Salvinia. Botanical Journal of the Linnean Society, 157(4), 673-685.

Nagalingum, N. S., Schneider, H., & Pryer, K. M. (2007). Molecular phylogenetic relationships and morphological evolution in the heterosporous fern genus Marsilea. Systematic Botany, 32(1), 16-25.

Nesom, G. L. (2010). Infrageneric classification of Verbena (Verbenaceae). Guy L. Nesom.

Nogueira, I., Ortiz, S., Paiva, J. A. R., & Pope, G. V. (2006). Polygonaceae. Flora Zambesiaca, 9(3), 1-49.

Nooteboom, H. P. (1960). Simaroubaceae. Flora Malesiana-Series 1, Spermatophyta, 6(1), 193-226.

Norscia, I., & Borgognini-Tarli, S. M. (2006). Ethnobotanical reputation of plant species from two forests of Madagascar: A preliminary investigation. South African Journal of Botany, 72(4), 656-660.

Obbard, D. J., Harris, S. A., Buggs, R. J., & Pannell, J. R. (2006). Hybridization, polyploidy, and the evolution of sexual systems in Mercurialis (Euphorbiaceae). Evolution, 60(9), 1801-1815.

Oberprieler, C., Himmelreich, S., & Vogt, R. (2007). A new subtribal classification of the tribe Anthemideae (Compositae). Willdenowia, 37(1), 89-114.

Olesen, J. M., & Ehlers, B. K. (2001). Age determination of individuals of Corydalis species and other perennial herbs. Nordic Journal of Botany, 21(2), 187-194.

Öztürk, M., Çelik, A., Güvensen, A., & Hamzaoğlu, E. (2008). Ecology of tertiary relict endemic Liquidambar orientalis Mill. forests. Forest Ecology and Management, 256(4), 510-518.

Padgett, D. J. (2007). A monograph of nuphar (nymphaeaceae) 1. Rhodora, 109(937), 1-95.

Page, C. N. (1990). Cupressaceae. In Pteridophytes and Gymnosperms (pp. 302-316). Berlin, Heidelberg: Springer Berlin Heidelberg.

Parker, V. T., Rodriguez, C. Y., Wechsler, G., & Vasey, M. C. (2020). Allopatry, hybridization, and reproductive isolation in Arctostaphylos. American Journal of Botany, 107(12), 1798-1814.

Pell, S. K., Mitchell, J. D., Miller, A. J., & Lobova, T. A. (2011). Anacardiaceae. Flowering plants. Eudicots: sapindales, cucurbitales, myrtaceae, 7-50.

Pereira, A. S. D. S., Barbosa, C. V. D. O., Silva, E. F. D., Guimarães, J. T. F., Filgueira, J. P. P. S., Teixeira, L. A., & Félix-da-Silva, M. M. (2021). Flora of Anacardium (Anacardiaceae) in the state of Pará, Brazil. Rodriguésia, 72, e02142020.

Philcox, D. (1990). Scrophulariaceae. In Flora Zambesiaca (Vol. 8, Part 2). Royal Botanic Gardens, Kew.

Pichi Sermolli, R. E. (1985). The fern genus Cosentinia Todaro. Webbia, 39(1), 179-189.

Pijut, P. M. (2000). Cedrus—the true cedars. Arboriculture & Urban Forestry (AUF), 26(4), 218-224.

Pijut, P. M. (2008). Carpinus. Woody plant seed manual, Agriculture Handbook, 727, 328-332.

Polhill, R. M. (1971). Phytolaccaceae. In Flora of Tropical East Africa. A.A. Balkema.

Polhill, R. M., & Beentje, H. (2005). Flora of Tropical East Africa: Santalaceae. Royal Botanic Gardens Kew.

Polhill, R. M., & Wiens, D. (1999). Flora of Tropical East Africa-Loranthaceae (1999) (Vol. 179). CRC Press.

Pope, G. V., Polhill, R. M., & Martins, E. S. (2006). Flora Zambesiaca (Vol. 9, Part 3). Polygonaceae-Myriaceae. Royal Botanic Gardens, Kew.

Pope, G. V., Polhill, R. M., & Martins, E. S. (2006). Polygonaceae–Myriaceae. In Flora Zambesiaca (Vol. 9, Part 3). Royal Botanic Gardens, Kew.

Prance, G. T., & Jongkind, C. C. (2015). A revision of African Lecythidaceae. Kew Bulletin, 70(1), 6.

Prance, G. T., & White, F. (1988). The genera of Chrysobalanaceae: a study in practical and theoretical taxonomy and its relevance to evolutionary biology. Philosophical Transactions of the Royal Society of London. Series B, Biological Sciences, 1-184.

Pringle, J. S. (2014). Morphological characteristics of the family Gentianaceae. In The Gentianaceae-Volume 1: Characterization and Ecology (pp. 1-12). Berlin, Heidelberg: Springer Berlin Heidelberg.

Proctor, G. R. (2012). Flora of the Cayman Islands (2nd ed.). Royal Botanic Gardens, Kew.

Puff, C. (2012). A biosystematic study of the African and Madagascan Rubiaceae-Anthospermeae (Vol. 3). Springer Science & Business Media.

Radcliffe-Smith, A. (1993). Notes on African Euphorbiaceae XXIX: Uapaca. Kew bulletin, 611-617.

Radcliffe-Smith, A. (1996). Euphorbiaceae Flora Zambesiaca 9 (4). Includes a picture, 157-159.

Ranjbar, M., & Khalvati, S. (2019). World checklist of Moltkia (Boraginaceae) with notes on types. Phytotaxa, 408(3), 143-160.

Reveal, J. L. (1978). Distribution and phylogeny of Eriogonoideae (Polygonaceae). Great Basin Naturalist Memoirs, 169-190.

Robinson, H. (1999). Revisions in paleotropical Vernonieae (Asteraceae). Biological Soc..

Robson, N. K. B. (1963). Ochnaceae. In Flora Zambesiaca (Vol. 2, Part 1). Crown Agents for Oversea Governments and Administrations.

Robson, N. K. B., Hallé, N., Mathew, B., & Blakelock, R. (1994). Celastraceae. In Flora of Tropical East Africa. Balkema/Rotterdam; A.A. Balkema.

Rothfels, C. J., Sundue, M. A., Kuo, L. Y., Larsson, A., Kato, M., Schuettpelz, E., & Pryer, K. M. (2012). A revised family–level classification for eupolypod II ferns (Polypodiidae: Polypodiales). Taxon, 61(3), 515-533.

Roux, J. P., Shaffer-Fehre, M., & Verdcourt, B. (2007). Dryopteridaceae. In Flora of Tropical East Africa. Royal Botanic Gardens, Kew.

Sang, T. (1995). Phylogeny and biogeography of Paeonia (Paeoniaceae). The Ohio State University.

Schellenberg, J., & Bergmeier, E. (2022). The Calluna life cycle concept revisited: Implications for heathland management. Biodiversity and Conservation, 31(1), 119-141.

Schüler, L., & Hemp, A. (2016). Atlas of pollen and spores and their parent taxa of Mt Kilimanjaro and tropical East Africa. Quaternary International, 425, 301–386. https://doi.org/10.1016/j.quaint.2016.07.038

Shalimov, A. P., Shrestha, N., & Zhang, X. C. (2017). Taxonomic study of the genus Huperzia Bernh.(Lycopodiaceae) in the Pan-Himalayan region. Indian Fern Journal, 34(1-2), 130-168.

Simpson, M. G. (2019). Evolution and diversity of woody and seed plants. In M. G. Simpson (Ed.), Plant systematics (3rd ed., pp. 131–165). Academic Press. https://doi.org/10.1016/B978-0-12-812628-8.50005-5

Sleumer, H. O. (1980). Flacourtiaceae. Flora Neotropica, 1-499.

Smith, A. R. (1987). Euphorbiaceae. In Flora of Tropical East Africa. Royal Botanic Gardens, Kew.

Smith, D. L. (1966). Linaceae. In Flora of Tropical East Africa. Crown Agents for Oversea Governments and Administrations.

Soltis, D. E., Tago-Nakazawa, M., Xiang, Q. Y., Kawano, S., Murata, J., Wakabayashi, M., & Hibsch-Jetter, C. (2001). Phylogenetic relationships and evolution in Chrysosplenium (Saxifragaceae) based on matK sequence data. American Journal of Botany, 88(5), 883-893.

Sprague, E. F. (1962). Pollination and evolution in Pedicularis (Scrophulariaceae). Aliso: A Journal of Systematic and Floristic Botany, 5(2), 181-209.

Sreenath, A., & Rao, B. R. P. (2021). Nine species of Riccia (Mich.) L., additions to Bryoflora of South India. International Journal of Advanced Research in Biological Sciences, 8, 198-205.

Stannard, B. (2000). Simaroubaceae. In Flora of Tropical East Africa. Royal Botanic Gardens, Kew.

Steinmann, V. W., & Porter, J. M. (2002). Phylogenetic relationships in Euphorbieae (Euphorbiaceae) based on ITS and ndhF sequence data. Annals of the Missouri Botanical Garden, 453-490.

Stoffelen, P., Robbrecht, E., & Smets, E. (1996). A revision of Corynanthe and Pausinystalia (African Rubiaceae-Coptosapelteae). Botanical Journal of the Linnean Society, 120(4), 287-326.

Stride, G., Nylinder, S., & Swenson, U. (2014). Revisiting the biogeography of Sideroxylon (Sapotaceae) and an evaluation of the taxonomic status of Argania and Spiniluma. Australian Systematic Botany, 27(2), 104-118.

Styles, B. T., & White, F. (1991). Flora of Tropical East Africa-Meliaceae. CRC Press.

Sun, M., & Lin, Q. (2010). A revision of Elaeagnus L.(Elaeagnaceae) in mainland China. Journal of Systematics and Evolution, 48(5), 356-390.

Sun, Q., Wang, N., Xu, W., & Zhou, H. (2021). Genus Ribes Linn.(Grossulariaceae): A comprehensive review of traditional uses, phytochemistry, pharmacology and clinical applications. Journal of Ethnopharmacology, 276, 114166.

Tang, Z.X. et al. (2011). Secale. In: Kole, C. (eds) Wild Crop Relatives: Genomic and Breeding Resources. Springer, Berlin, Heidelberg.

Tawfeek, N., Mahmoud, M. F., Hamdan, D. I., Sobeh, M., Farrag, N., Wink, M., & El-Shazly, A. M. (2021). Phytochemistry, pharmacology and medicinal uses of plants of the genus Salix: An updated review. Frontiers in pharmacology, 12, 593856.

Taylor, P. (1958). The Genus Caylusea St. Hil. in Tropical Africa. Kew Bulletin, 13(2), 283-286.

Taylor, P. (1974). Lentibulariaceae. Flora Malesiana-Series 1, Spermatophyta, 8(1), 275-300.

Teichman, I. V., & Van Wyk, A. E. (1996). Taxonomic significance of pericarp and seed structure in Heeria argentea (Thunb.) Meisn. (Anacardiaceae), including reference to pachychalazy and recalcitrance. Botanical Journal of the Linnean Society, 122(4), 335-352.

Thambugala, K. M., Daranagama, D. A., Phillips, A. J., Bulgakov, T. S., Bhat, D. J., Camporesi, E., ... & Hyde, K. D. (2017). Microfungi on Tamarix. Fungal Diversity, 82, 239-306.

Thatoi, H., Samantaray, D., & Das, S. K. (2016). The genus Avicennia, a pioneer group of dominant mangrove plant species with potential medicinal values: a review. Frontiers in Life Science, 9(4), 267-291.

Thiv, M. (2016). Vahliaceae. In: Kadereit, J., Bittrich, V. (eds) Flowering Plants. Eudicots. The Families and Genera of Vascular Plants, vol 14. Springer, Cham. https://doi.org/10.1007/978-3-319-28534-4_34

Thulin, M. (1983). Lobeliaceae. In Flora Zambesiaca (Vol. 7, Part 1). Royal Botanic Gardens, Kew.

Thulin, M., et al. (2008). Flora of Somalia, Vol. 1-4. https://plants.jstor.org/collection/FLOS

Timberlake, J. R., & Martins, E. S. (Eds.). (2009). Flora Zambesiaca (Vol. 12, Part 2). Royal Botanic Gardens, Kew.

Timberlake, J. R., & Martins, E. S. (Eds.). (2013). Flora Zambesiaca (Vol. 8, Part 5). University of Chicago Press. ISBN: 978-1842464120

Tomou, E. M., Lytra, K., Rallis, S., Tzakos, A. G., & Skaltsa, H. (2022). An updated review of genus Cistus L. since 2014: traditional uses, phytochemistry, and pharmacological properties. Phytochemistry Reviews, 21(6), 2049-2087.

Townsend, C. C. (1988). Amaranthaceae. In Flora Zambesiaca (Vol. 9, Part 1). Royal Botanic Gardens, Kew.

Townsend, C. C. (1989). Flora of Tropical East Africa-Unibelliferae (Vol. 187). CRC Press.

Townsend, C. C. (1990). Three New Species of Psilotrichum from Somalia: Notes on Amaranthaceae: XX. Kew bulletin, 661-665.

Troupin, G. (1960). Menispermaceae. In Flora Zambesiaca (Vol. 1, Part 1). Royal Botanic Gardens, Kew.

Tryon, A. F. (1957). A revision of the fern genus Pellaea section Pellaea. Annals of the Missouri Botanical Garden, 44(2), 125-193.

Tsymbalyuk, Z. M., Çelenk, S., Bell, C. D., Nitsenko, L. M., & Mosyakin, S. L. (2022). Comparative palynomorphological study of the genus Symphoricarpos (Caprifoliaceae): exine sculpture and implications for evolution. Palynology, 46(4), 1-14.

Turner, I. M. (2018). A revised conspectus of Uncaria (Rubiaceae). Webbia, 73(1), 9-21.

Utteridge, T. M. A., & Jennings, L. V. S. (2022). Trees of New Guinea. Royal Botanic Gardens, Kew.

Utteridge, T., & Bramley, G. (2020). The Kew Tropical Plant Families Identification Handbook (2nd ed.). Kew Publishing, Royal Botanic Gardens, Kew.

Valcárcel, V., Guzmán, B., Medina, N. G., Vargas, P., & Wen, J. (2017). Phylogenetic and paleobotanical evidence for late Miocene diversification of the Tertiary subtropical lineage of ivies (Hedera L., Araliaceae). BMC Evolutionary Biology, 17, 1-14.

Van der Plas, F. (1972). Lemnaceae. Flora Malesiana-Series 1, Spermatophyta, 7(1), 219-237.

Verdcourt, B. (1953). A revision of certain African genera of herbaceous Rubiaceae II. The genus Otomeria Benth. and the new genus Batopedina Verdcourt. Bulletin du Jardin botanique de l'Etat, Bruxelles/Bulletin van den Rijksplantentuin, Brussel, 5-34.

Verdcourt, B. (1971). Hamamelidaceae. In Flora of Tropical East Africa. Royal Botanic Gardens, Kew.

Verdcourt, B. (1976). Rubiaceae. In Flora of Tropical East Africa. Royal Botanic Gardens, Kew.

Verdcourt, B. (1985). Hernandiaceae. In Flora of Tropical East Africa. Royal Botanic Gardens, Kew.

Verdcourt, B. (1989). Flora of Tropical East Africa-Nymphaeceae (1989) (Vol. 128). CRC Press.

Verdcourt, B. (1991). Boraginaceae. In Flora of Tropical East Africa. Balkema.

Verdcourt, B. (1997). Flora of Tropical East Africa-Myristicaceae (1997). CRC Press.

Verdcourt, B. (2000). Salviniaceae. In Flora of Tropical East Africa. Royal Botanic Gardens, Kew.

Verdcourt, B., & Mwachala, G. M. (2009). Malvaceae. In H. J. Beentje & S. A. Ghazanfar (Eds.), Flora of Tropical East Africa (pp. 1-170). Published on behalf of the East African Governments by Royal Botanic Gardens, Kew. Kew, UK. ISBN: 978-1-84246-189-1.

Vickery, A. R. (1983). Salvadoraceae. In Flora Zambesiaca (Vol. 7, Part 1). Royal Botanic Gardens, Kew.

Viñas, R. A., Caudullo, G., Oliveira, S., & De Rigo, D. (2016). Pinus pinea in Europe: distribution, habitat, usage and threats. European atlas of forest tree species, 204.

Vollesen, K. (2008). Acanthaceae (Part 1). In Flora of Tropical East Africa. Royal Botanic Gardens, Kew.

Vollesen, K. (2008). Acanthaceae (Part 2). In Flora of Tropical East Africa. Royal Botanic Gardens, Kew.

Vorontsova, M. S., & Hoffmann, P. (2008). A phylogenetic classification of tribe Poranthereae (Phyllanthaceae, Euphorbiaceae sensu lato). Kew Bulletin, 63, 41-59.

Vorontsova, M. S., & Knapp, S. (2012). Solanum sections Oliganthes, Melongena and Monodolichopus. Edmonds JM, Solanaceae, In: Beentje H, editor. Flora of Tropical East Africa. Richmond: RBG Kew, 164-186.

Wala, M., Kołodziejek, J., Mazur, J., & Cienkowska, A. (2021). Reactions of two xeric-congeneric species of Centaurea (Asteraceae) to soils with different pH values and iron availability. PeerJ, 9, e12417.

Wallander, E. (2012). Systematics and floral evolution in Fraxinus (Oleaceae). Belgische Dendrologie Belge, 2012, 39-58.

Wang, Y. F., Li, C. S., Collinson, M. E., Lin, J., & Sun, Q. G. (2003). Eucommia (Eucommiaceae), a potential biothermometer for the reconstruction of paleoenvironments. American Journal of Botany, 90(1), 1-7.

Wang, Y., Liu, Y. F., Liu, S. B., & Huang, H. W. (2009). Molecular phylogeny of Myricaria (Tamaricaceae): implications for taxonomy and conservation in China. Bot Stud, 50, 343-352.

Wei, R., Ebihara, A., Zhu, Y. M., Zhao, C. F., Hennequin, S., & Zhang, X. C. (2018). A total-evidence phylogeny of the lady fern genus Athyrium Roth (Athyriaceae) with a new infrageneric classification. Molecular Phylogenetics and Evolution, 119, 25-36.

Wendel, J. F., Brubaker, C. L., & Seelanan, T. (2010). The origin and evolution of Gossypium. In Physiology of cotton (pp. 1-18). Dordrecht: Springer Netherlands.

Widuri, R., & van Welzen, P. E. (1998). A revision of the genus Cephalomappa (Euphorbiaceae) in Malesia. Reinwardtia, 11(3), 153–184.

Wild, H. (1961). Sterculiaceae. In Flora Zambesiaca (Vol. 1, Part 2). Royal Botanic Gardens, Kew.

Wild, H., & Phipps, J. B. (1963). Simaroubaceae. In Flora Zambesiaca (Vol. 2, Part 1). Crown Agents for Oversea Governments and Administrations.

Wilmot-Dear, C. M. (1985). Flora of Tropical East Africa-Ceratophyllaceae (1985). CRC Press.

Wilson, P.G. (2010). Myrtaceae. In: Kubitzki, K. (eds) Flowering Plants. Eudicots. The Families and Genera of Vascular Plants, vol 10. Springer, Berlin, Heidelberg. https://doi.org/10.1007/978-3-642-14397-7_14

Winkworth, R. C., & Donoghue, M. J. (2005). Viburnum phylogeny based on combined molecular data: implications for taxonomy and biogeography. American Journal of Botany, 92(4), 653-666.

Winter, P. J. D., & Van Wyk, B. E. (1996). A revision of the genus Heteromorpha (Apiaceae). Kew Bulletin, 225-265.

Woodson, R. E. (1930). Studies in the Apocynaceae. I. A Critical Study of the Apocynoideae (With Special Reference to the FGenus Apocynum). Annals of the Missouri Botanical Garden, 17(1/2), 1-212.

Wortley, A. (2004). Systematics of Thomandersia Baill (Doctoral dissertation, University of Oxford).

Xiang, X., Cao, M., & Zhou, Z. (2007). Fossil history and modern distribution of the genus Abies (Pinaceae). Frontiers of Forestry in China, 2, 355-365.

Xu, Z., Chang, L., Xu, Z., & Chang, L. (2017). Hydrocharitaceae. Identification and Control of Common Weeds: Volume 3, 753-763.

Yaniv, Z., & Dudai, N. (Eds.). (2014). Medicinal and aromatic plants of the middle-east (Vol. 2, pp. 67-150). Dordrecht Heidelberg New York London: Springer.

Youngblood, A. (2005). Frangula P. Mill. Woody plant seed manual. United States Department of Agriculture, 28.

Yurtseva, O. V., Kuznetsova, O. I., Mavrodieva, M. E., & Mavrodiev, E. V. (2016). What is Atraphaxis L.(Polygonaceae, Polygoneae): cryptic taxa and resolved taxonomic complexity instead of the formal lumping and the lack of morphological synapomorphies. PeerJ, 4, e1977.

Zhang, H. J., Zhang, X., Landis, J. B., Sun, Y. X., Sun, J., Kuang, T. H., ... & Wang, H. C. (2022). Phylogenomic and comparative analyses of Rheum (Polygonaceae, Polygonoideae). Journal of Systematics and Evolution, 60(6), 1229-1240.

Zhang, M. L., Temirbayeva, K., Sanderson, S. C., & Chen, X. (2015). Young dispersal of xerophil Nitraria lineages in intercontinental disjunctions of the Old World. Scientific Reports, 5(1), 13840.

Zhang, Z., Li, S., & Lian, X. Y. (2010). An overview of genus Aesculus L.: ethnobotany, phytochemistry, and pharmacological activities. Pharmaceutical Crops, 1(1).

Zhao, J., Zhang, Z., Zhou, H., Bai, Z., & Sun, K. (2023). The study on sea buckthorn (genus Hippophae L.) fruit reveals cell division and cell expansion to promote morphogenesis. Plants, 12(5), 1005.

Zhou, P., Li, J., Chen, Q., Wang, L., Yang, J., Wu, A., ... & Wu, J. (2021). A comprehensive review of genus Sanguisorba: Traditional uses, chemical constituents and medical applications. Frontiers in Pharmacology, 12, 750165.

Zomlefer, W. B. (1997). The genera of Tofieldiaceae in the southeastern United States. Harvard Papers in Botany, 2(2), 179-194.

6. References: phytogeographical affinity classification

Abidkulova, K. T., Almabek, D. M., Ivashchenko, A. A., Mukhitdinov, N. M., Kurbatova, N. V., Turalin, B. A., & Karasholakova, L. N. (2024). Herbarium as tools in study of plant distribution and ecology (on the examples Kazakh species of Leontice and Gymnospermium). Eurasian Journal of Ecology, 78(1).

Abo, K. A., & Olugbuyiro, J. A. O. (2004). Phytochemical and antibacterial studies of extracts of Flabellaria. African Journal of Biomedical Research, 7(1).

Aduku, O., Abdullahi, S., Sule, M., Atiku, I., & Anyam, J. (2020). Isolation of taraxasterol and stigmasterol from the aerial part of Centaurea perrottetii DC. (Asteraceae). Bima Journal of Science and Technology, 4(2), 86–93.

Akalezi, C. O., Enenebaku, C. K., & Oguzie, E. E. (2013). Inhibition of acid corrosion of mild steel by biomass extract from the Petersianthus macrocarpus plant. J. Mater. Environ. Sci, 4(2), 217-226.

Alonso, M. Á., Crespo, M. B., Martínez-Azorín, M., & Mucina, L. (2021). Taxonomic identity and evolutionary relationships of South African taxa related to the Spergularia media group (Caryophyllaceae). Plant Systematics and Evolution, 307(2), 24.

Alzahrani, D. A., Albokhari, E. J., & Khoj, A. (2021). Taxonomic studies on some members of the genus Abutilon Mill.(Malvaceae). American Journal of Plant Sciences, 12(02), 199.

An-Ming, L., & Zhi-Yun, Z. (1990). The differentiation, evolution and systematic relationship of Juglandales. Journal of Systematics and Evolution, 28(2), 96.

Aradhya, M. K., Potter, D. A. N. I. E. L., & Simon, C. J. (2004, November). Origin, evolution, and biogeography of Juglans: a phylogenetic perspective. In V International Walnut Symposium 705 (pp. 85-94).

Arana, M. D., & Mynssen, C. M. (2015). Cystopteris (Cystopteridaceae) del cono sur y Brasil. Darwiniana, nueva serie, 3(1), 73-88.

Arya, S., Singh, H., Vishnu, K. W., & Iamonico, D. (2024). Study on the Genus Drymaria (Caryophyllaceae)—A New Species from North-East India. Plants, 13(23), 3378.

Backlund, M., & Thulin, M. (2007). Revision of the Mediterranean species of Plocama (Rubiaceae). Taxon, 56(2), 516-520.

Bahramsoltani, R., Kalkhorani, M., Zaidi, S. M. A., Farzaei, M. H., & Rahimi, R. (2020). The genus Tamarix: Traditional uses, phytochemistry, and pharmacology. Journal of ethnopharmacology, 246, 112245.

Baker, W.J., Barfod, A.S., Cámara-Leret, R., Dowe, J.L., Heatubun, C.D., Petoe, P., Turner, J.H., Zona, S. & Dransfield, J. (2024) Palms of New Guinea. Royal Botanic Gardens, Kew, Richmond. 726 pp.

Barres, L., Sanmartín, I., Anderson, C. L., Susanna, A., Buerki, S., Galbany-Casals, M., & Vilatersana, R. (2013). Reconstructing the evolution and biogeographic history of tribe Cardueae (Compositae). American Journal of Botany, 100(5), 867-882.

Beardsley, P. M., & Olmstead, R. G. (2002). Redefining Phrymaceae: the placement of Mimulus, tribe Mimuleae, and Phryma. American journal of Botany, 89(7), 1093-1102.

Beentje, H. (2008). Hymenophyllaceae. In Flora of Tropical East Africa. Royal Botanic Gardens, Kew.

Beentje, H., Jeffrey, C., & Hind, D. J. N. (2005). Compositae. In Flora of Tropical East Africa.

Bentvelzen, P. A. J. (1960). Primulaceae. Flora Malesiana-Series 1, Spermatophyta, 6(1), 173-192.

Beyschlag, W. (2024). Bryophytes. In: Büdel, B., Friedl, T., Beyschlag, W. (eds) Biology of Algae, Lichens and Bryophytes. Springer Spektrum, Berlin, Heidelberg.

Bezverkhniaia, E. A., Ermilova, E. V., Kadyrova, T. V., Krasnov, E. A., Brazovskii, K. S., Ponkratova, A. O., ... & Belousov, M. V. (2023). Phytochemistry, ethnopharmacology and pharmacology of the genus Empetrum: A review. Advances in Traditional Medicine, 23(3), 659-672.

Biasuso, A. B. (2007). The genus Hedwigia (Hedwigiaceae, Bryophyta) in Argentina. Lindbergia, 5-17.

Bissiengou, P. (2014). Systematics, evolution and historical biogeography of the family Ochnaceae with emphasis on the genus Campylospermum. Wageningen University and Research.

Borosova, R., Utteridge, T.M.A. & Schuiteman, A.(2021). Taxonomy and morphology of Thalictrum (Ranunculaceae) in New Guineo. Kew Bull 76, 805–817

Boutique, R., & Verdcourt, B. (1973). Haloragaceae. In Flora of Tropical East Africa. Balkema.

Bouzarisaravani, Z., Sharifnia, F., Salimpour, F., Arbabian, S., & Geran, A. (2021). Molecular systematic studies in the genus Glaucium (Papaveraceae). Genetika, 53(3), 1179-1192.

Bramley, G., Trias-Blasi, A., & Wilford, R. (2023). The Kew Temperate Plant Families Identification Handbook. Royal Botanic Gardens, Kew.

Brenan, J.P.M. (1953) Flora of Tropical East Africa: Trapaceae. London: Crown Agents for Overseas Governments & Administrations. 4 pp. ISBN 978-1-84246-322-2

Breteler, F. J. (2001). The genus Trichoscypha (Anacardiaceae) in Upper Guinea: A synoptic revision. Adansonia, 23(2), 247-264.

Bricca, A., Jiménez-Alfaro, B., Chytrý, M., Chytrý, K., Padullés Cubino, J., Fernández-González, F., ... & Bonari, G. (2025). Biodiversity within and beyond the native distribution of tree species: The case of Pinus nigra forests in Europe. Global Ecology and Biogeography, 34(4), e70036.

Bridson, D. M. (1986). The reinstatement of the African genus Keetia (Rubiaceae subfam. Cinchonoideae, tribe Vanguerieae). Kew bulletin, 965-994.

Brock, T. C., Mielo, H., & Oostermeijer, G. (1989). On the life cycle and germination of Hottonia palustris L. in a wetland forest. Aquatic Botany, 35(2), 153-166.

Butcher, R. W. (1947). Atropa Belladonna L. Journal of Ecology, 34(2), 345-353.

Cantó, P. (2011). Biogeographic and bioclimatic distribution of Klasea Cass. and Serratula L. Acta Botanica Gallica, 158(2), 239-249.

Carbone, K., & Gervasi, F. (2022). An updated review of the genus Humulus: a valuable source of bioactive compounds for health and disease prevention. Plants, 11(24), 3434.

Carlson, S. E. (2010). Morphological innovation, dispersal, and diversification in the plant clade Dipsacaceae (Dipsacales). Yale University.

Carneiro, M. R. B., Sallum, L. O., Martins, J. L. R., Peixoto, J. D. C., Napolitano, H. B., & Rosseto, L. P. (2023). Overview of the Justicia genus: insights into its chemical diversity and biological potential. Molecules, 28(3), 1190.

Carrion, J. S., Delgado, M. J., & Garcia, M. (1993). Pollen grain morphology of Coris (Primulaceae). Plant systematics and evolution, 184, 89-100.

Carta, A., Savio, L., Bedini, G., Peruzzi, L., Fisogni, A., & Galloni, M. (2016). All in an afternoon: mixed breeding system in one-day lasting flowers of Hypericum elodes L.(Hypericaceae). Plant Biosystems-An International Journal Dealing with all Aspects of Plant Biology, 150(5), 1001-1009.

Carter, S., & Smith, A. R. (2020). Flora of Tropical East Africa-Euphorbiac v2 (1988). CRC Press.

Castroviejo, S., Aedo, C., Cirujano, S., Laínz, M., Montserrat, P., Morales, R., Muñoz Garmendia, F., Navarro, C., Paiva, J. & Soriano, C. (eds.). 1993. Flora iberica vol. CXLVI GLOBULARIACEAE. Real Jardín Botánico, CSIC, Madrid.

Castroviejo, S., Aedo, C., Cirujano, S., Laínz, M., Montserrat, P., Morales, R., Muñoz Garmendia, F., Navarro, C., Paiva, J. & Soriano, C. (eds.). 1993. Flora iberica vol. LXXXVII. Real Jardín Botánico, CSIC, Madrid.

Chawla, R., Kumar, S., & Sharma, A. (2012). The genus Clematis (Ranunculaceae): chemical and pharmacological perspectives. Journal of ethnopharmacology, 143(1), 116-150.

Cheek, M. & Jongkind, C.C.H. (2008). Two new names in West-Central African Quassia L. (Simaroubaceae). Kew Bulletin 63: 247. https://doi.org/10.1007/s12225-008-9022-1

Chen, JF., Zhou, XH. (2011). Cucumis. In: Kole, C. (eds) Wild Crop Relatives: Genomic and Breeding Resources. Springer, Berlin, Heidelberg.

Chen, L. Y., Zhao, S. Y., Mao, K. S., Les, D. H., Wang, Q. F., & Moody, M. L. (2014). Historical biogeography of Haloragaceae: An out-of-Australia hypothesis with multiple intercontinental dispersals. Molecular Phylogenetics and Evolution, 78, 87-95.

Christenhusz, M. J., Chase, M. W., Fay, M. F., Hidalgo, O., Leitch, I. J., Pellicer, J., & Viruel, J. (2021). Biogeography and genome size evolution of the oldest extant vascular plant genus, Equisetum (Equisetaceae). Annals of Botany, 127(5), 681-695.

Chung, K. S. (2008). A systematic study of genus Agrimonia (Rosaceae). The University of Oklahoma.

Cook, C. D., Gut, B. J., Rix, E. M., & Schneller, J. (1974). Water plants of the world: a manual for the identification of the genera of freshwater macrophytes. Springer Science & Business Media.

Coutinho, T. S., Barbosa-Silva, R. G., & Dorr, L. J. (2025). A synopsis of Christiana DC.(Malvaceae, Brownlowioideae), with a new species from the Brazilian Atlantic Forest. PhytoKeys, 253, 33.

Cramer, L. H. (1991). Brillantaisia P. Beauv.(Acanthaceae), a new generic record for Asia. Kew bulletin, 335-338.

Crockett, S. L., & Robson, N. K. (2011). Taxonomy and chemotaxonomy of the genus Hypericum. Medicinal and aromatic plant science and biotechnology, 5(Special Issue 1), 1.

Cusma-Velari, T., & Feoli-Chiapella, L. (2009). The so-called primitive genera of Genisteae (Fabaceae): systematic and phyletic considerations based on karyological data. Botanical Journal of the Linnean Society, 160(2), 232-248.

Dahl, Å. E. (1990). Infrageneric division of the genus Hypecoum (Papaveraceae). Nordic journal of botany, 10(2), 129-140.

Dahlgren, R. M., Clifford, H. T., & Yeo, P. F. (2012). The families of the monocotyledons: structure, evolution, and taxonomy. Springer Science & Business Media. Chicago

Dai, X., Li, X., Song, X., Li, X., & Liu, X. (2021). The evolutionary history and phylogeographic pattern of Hippuris vulgaris: hybridization and long-distance dispersal from China. Plant Systematics and Evolution, 307, 1-8.

Darbyshire, I., Vollesen, K., & Kelbessa, E. (2015). Acanthaceae (Part 2). In Flora Zambesiaca. Royal Botanic Gardens, Kew.

Dauphin, B., Farrar, D. R., Maccagni, A., & Grant, J. R. (2017). A worldwide molecular phylogeny provides new insight on cryptic diversity within the moonworts (Botrychium ss, Ophioglossaceae). Systematic Botany, 42(4), 620-639.

Davidson, C. (1973). An anatomical and morphological study of Datiscaceae. Aliso: A Journal of Systematic and Floristic Botany, 8(1), 49-110.

Davis, C. C., Bell, C. D., Fritsch, P. W., & Mathews, S. (2002). Phylogeny of Acridocarpus-Brachylophon (Malpighiaceae): implications for Tertiary tropical floras and Afroasian biogeography. Evolution, 56(12), 2395-2405.

De Benedetti, C., Gerasimenko, N., Ravazzi, C., & Magri, D. (2022). History of Tilia in Europe since the Eemian: Past distribution patterns. Review of Palaeobotany and Palynology, 307, 104778.

de Oliveira, L. D. S., da Silva, M. J., & de Sales, M. F. (2013). Synopsis of the tribe Hureae (Euphorbioideae, Euphorbiaceae). Brittonia, 65, 310-329.

Del Rio, C., Stull, G. W., & De Franceschi, D. (2019). New species of Iodes fruits (Icacinaceae) from the early Eocene Le Quesnoy locality, Oise, France. Review of Palaeobotany and Palynology, 262, 60-71.

Deng, T., Zhang, J. W., Meng, Y., Volis, S., Sun, H., & Nie, Z. L. (2017). Role of the Qinghai-Tibetan Plateau uplift in the Northern Hemisphere disjunction: evidence from two herbaceous genera of Rubiaceae. Scientific Reports, 7(1), 13411.

DeSilva, R., & Dodd, R. S. (2021). Patterns of fine-scale spatial genetic structure and pollen dispersal in giant sequoia (Sequoiadendron giganteum). Forests, 12(1), 61.

Dessein, S., Jansen, S., Huysmans, S., Robbrecht, E., & Smets, E. (2001). A morphological and anatomical survey of Virectaria (African Rubiaceae), with a discussion of its taxonomic position. Botanical Journal of the Linnean Society, 137(1), 1-29.

Dinda, B., Das, N., Dinda, S., Dinda, M., & SilSarma, I. (2015). The genus Sida L.–A traditional medicine: Its ethnopharmacological, phytochemical and pharmacological data for commercial exploitation in herbal drugs industry. Journal of Ethnopharmacology, 176, 135-176.

Dorr, L. J., & Barnett, L. C. (1990). A new species of Hildegardia (Sterculiaceae) from Somalia. Kew Bulletin, 577-580.

Dransfield, J. (1994). Palmae. In Flora of Tropical East Africa. A. A. Balkema.

Durka, W. (1999). Genetic diversity in peripheral and subcentral populations of Corrigiola litoralis L.(Illecebraceae). Heredity, 83(4), 476-484.

Durrant, T. H., De Rigo, D., & Caudullo, G. (2016). Pinus sylvestris in Europe: distribution, habitat, usage and threats. European atlas of forest tree species, 14, 845-846.

Durrant, T. H., De Rigo, D., & Caudullo, G. (2016). Quercus suber in Europe: distribution, habitat, usage and threats. Eur. Atlas For. Tree Species, 164-165.

Ekalu, A. (2021). Medicinal uses, phytochemistry, and pharmacological activities of Mitracarpus species (Rubiaceae): A review. Scientific African, 11, e00692.

El Ghazali, G. E. B. (1993). A Study on the Pollen Flora of Sudan. Review of Palaeobotany and Palynology, 76, 99-345.

Enright, N. J., & Jaffré, T. (2011). Ecology and distribution of the Malesian podocarps. Smithsonian contributions to botany, 95, 57-78.

Erbar, C., & Leins, P. (1995). Portioned pollen release and the syndromes of secondary pollen presentation in the Campanulales-Asterales-complex. Flora, 190(4), 323-338.

Fan, D. M., Chen, J. H., Meng, Y., Wen, J., Huang, J. L., & Yang, Y. P. (2013). Molecular phylogeny of Koenigia L.(Polygonaceae: Persicarieae): Implications for classification, character evolution and biogeography. Molecular Phylogenetics and Evolution, 69(3), 1093-1100.

Fang, J., & Lechowicz, M. J. (2006). Climatic limits for the present distribution of beech (Fagus L.) species in the world. Journal of Biogeography, 33(10), 1804-1819.

Feng, A. L., Lin, M., Tian, L., Zhu, H. Y., Guo, H., Singamaneni, S., ... & Xu, F. (2015). Selective enhancement of red emission from upconversion nanoparticles via surface plasmon-coupled emission. RSC advances, 5(94), 76825-76835.

Fernandes, R., & Diniz, M. A. (2005). Avicenniaceae, Nesogenaceae, Verbenaceae, and Lamiaceae (subfamilies Viticoideae and Ajugoideae). Flora Zambesiaca 8(7). Richmond: Royal Botanic Gardens, Kew.

Ferreira, R. C., Piredda, R., Bagnoli, F., Bellarosa, R., Attimonelli, M., Fineschi, S., ... & Simeone, M. C. (2011). Phylogeography and conservation perspectives of an endangered Macaronesian endemic: Picconia azorica (Tutin) Knobl.(Oleaceae). European Journal of Forest Research, 130(2), 181-195.

Feuer, S. M. (1978). Aperture evolution in the genus Ptychopetalum Benth.(Olacaceae). American Journal of Botany, 65(7), 759-763.

Friis, I. (1987). A reconsideration of Pittosporum in Africa and Arabia. Kew bulletin, 319-335.

Fritsch, P. W. (2001). Phylogeny and biogeography of the flowering plant genus Styrax (Styracaceae) based on chloroplast DNA restriction sites and DNA sequences of the internal transcribed spacer region. Molecular Phylogenetics and Evolution, 19(3), 387-408.

Frodin, D. G., Lowry II, P. P., & Plunkett, G. M. (2010). Schefflera (Araliaceae): taxonomic history, overview and progress. Plant Diversity and Evolution, 128(3), 561.

Funez, L. A., & Hassemer, G. (2018). Novelties in the genus Persicaria (Polygonaceae) in Brazil: A new species, a new combination, and a diagnostic key to all species. Nordic Journal of Botany, 36(1_2), njb-01631.

Funk, V., Susanna, A., Stuessy, T., & Bayer, R. (2009). Systematics, Evolution, and Biogeography of Compositae. Vienna: International Association for Plant Taxonomy. ISBN: 978-3-9501754-3-1.

García, M. A., Costea, M., Kuzmina, M., & Stefanović, S. (2014). Phylogeny, character evolution, and biogeography of Cuscuta (dodders; Convolvulaceae) inferred from coding plastid and nuclear sequences. American journal of botany, 101(4), 670-690.

Ghazanfar, S. A. & Edmondson, J. R (Eds). (2014) Flora of Iraq, Volume 5 Part 2: Lythraceae to Campanulaceae.

Ghazanfar, S. A., Edmondson, J. R. & Hind, D. J. N. (Eds). (2019). Flora of Iraq, Volume 6: Compositae.Kew Publishing

Ghazanfar, S. A., Edmondson, J. R. (Eds). (2016). Flora of Iraq, Volume 5, Part 1: Elatinaceae to Sphenocleaceae. Kew Publishing

Ghazanfar, S. A., Raven, P. H., Townsend, C. C., Taylor, P., & Mobayen, S. (2013). Flora of Iraq (Vol. 5, Part 2, pp. xi, 1-349). In S. A. Ghazanfar & J. R. Edmondson (Eds.), Kew, UK: Royal Botanic Gardens, Kew. Published on behalf of the Ministry of Agriculture, Republic of Iraq by Royal Botanic Gardens, Kew. ISBN 978-1-84246-493-9.

Giacosa, J. R., Morbelli, M., & Giudice, G. (2004). Spore wall ultrastructure in Anogramma species (Pteridaceae) from Argentina. Grana, 43(4), 231-237.

Gildenhuys, E., Ellis, A. G., Carroll, S. P., & Le Roux, J. J. (2015). Combining natal range distributions and phylogeny to resolve biogeographic uncertainties in balloon vines (Cardiospermum, Sapindaceae). Diversity and Distributions, 21(2), 163-174.

Gonella, P. M. (2017). Systematics of Drosera sect. Drosera ss (Droseraceae) (Doctoral dissertation, Universidade de São Paulo).

Gosline, G., Bidault, E., van der Burgt, X., Cahen, D., Challen, G., Condé, N., ... & Cheek, M. (2023). A Taxonomically-verified and Vouchered Checklist of the Vascular Plants of the Republic of Guinea. Scientific Data, 10(1), 327.

Goula, K., & Goula, K. (2023). Flora of Greece Volume 1 Apocynaceae: Apocynum (ed. 1).

Goyder, D. J. (2006). An overview of Asclepiad biogeography. Taxonomy and ecology of African plants, their conservation and sustainable use, 205-214.

Goyder, D. J., Gilbert, M. G. & Venter, H. J. T. (2020). Apocynaceae (part 2). In: M. A. García (ed.), Flora Zambesiaca, Vol. 7(3). Royal Botanic Gardens, Kew.

Grey-Wilson, C. (1980). Impatiens of Africa. CrC Press.

Grimm, G. W., & Denk, T. (2008). ITS evolution in Platanus (Platanaceae): homoeologues, pseudogenes and ancient hybridization. Annals of Botany, 101(3), 403-419.

Gustafsson, C., & Persson, C. (2002). Phylogenetic relationships among species of the neotropical genus Randia (Rubiaceae, Gardenieae) inferred from molecular and morphological data. Taxon, 51(4), 661-674.

Harley, R. M., et al. (2004). Labiatae. In K. Kubitzki (Ed.), The Families and Genera of Vascular Plants (Vol. VI, pp. 167-275). Springer-Verlag.

Harpke, D., Peruzzi, L., Kerndorff, H., Karamplianis, T., Constantinidis, T., Randelovic, V., ... & Blattner, F. R. (2014). Phylogeny, geographic distribution, and new taxonomic circumscription of the Crocus reticulatus species group (Iridaceae). Turkish Journal of Botany, 38(6), 1182-1198.

Hawksworth, F. G., & Wiens, D. (1972). Biology and classification of dwarf mistletoes (Arceuthobium) (No. 401). US Forest Service.

Hayden, W. J., Simmons, M. P., & Swanson, L. J. (1993). Wood anatomy of Amanoa (Euphorbiaceae). IAWA Journal, 14(2), 205-213.

Hearn, D. J. (2007). Novelties in Adenia (Passifloraceae): Four new species, a new combination, a vegetative key, and diagnostic characters for known Madagascan species. Brittonia, 59(4), 308-327.

Heine, H. (1963). The Genus Calycobolus Willd. ex. Roem. & Schultes (Convolvulaceae) in Africa. Kew Bulletin, 16(3), 387-391.

Heitzman, M. E., Neto, C. C., Winiarz, E., Vaisberg, A. J., & Hammond, G. B. (2005). Ethnobotany, phytochemistry and pharmacology of Uncaria (Rubiaceae). Phytochemistry, 66(1), 5-29.

Hély, C., Lézine, A.-M., & Contributors, A. (2014). Holocene changes in African vegetation: Tradeoff between climate and water availability. Clim. Past, 10, 681–686. https://doi.org/10.5194/cp-10-681-2014

Henkin, Z., Rosenzweig, T., Yaniv, Z. (2014). Sarcopoterium spinosum . In: Yaniv, Z., Dudai, N. (eds) Medicinal and Aromatic Plants of the Middle-East. Medicinal and Aromatic Plants of the World, vol 2.

Henrickson, J. (1987). A taxonomic reevaluation of Gossypianthus and Guilleminea (Amaranthaceae). SIDA, Contributions to Botany, 12(4), 307–337.

Hildebrand, T. J. (2005). Sectional revision, speciation, and population biology in lycopodium (lycopodiaceae) (Order No. 3250052). Available from ProQuest Dissertations & Theses Global. (304989785). Retrieved from https://liverpool.idm.oclc.org/login?url=https://www.proquest.com/dissertations-theses/sectional-revision-speciation-population-biology/docview/304989785/se-2

Hoggard, R. K., Kores, P. J., Molvray, M., Hoggard, G. D., & Broughton, D. A. (2003). Molecular systematics and biogeography of the amphibious genus Littorella (Plantaginaceae). American Journal of Botany, 90(3), 429-435.

Hou, D., Larsen, K., & Larsen, S. S. (1996). Caesalpiniaceae (Leguminosae-Caesalpinioideae). Flora Malesiana-Series 1, Spermatophyta, 12(2), 409-730.

Huang, Y. J., Liu, Y. S., Wen, J., & Quan, C. (2015). First fossil record of Staphylea L.(Staphyleaceae) from North America, and its biogeographic implications. Plant Systematics and Evolution, 301(9), 2203-2218.

Hummer, K. E., Bassil, N., & Njuguna, W. (2010). Fragaria. In Wild Crop Relatives: Genomic and Breeding Resources: Temperate Fruits (pp. 17-44). Berlin, Heidelberg: Springer Berlin Heidelberg.

Hutchinson, J. (1933). The Genus Trichocladus Pers.(Hamamelidaceae). Bulletin of Miscellaneous Information (Royal Botanic Gardens, Kew), 1933(9), 427-430.

Ibarra-Morales, A., Muñíz, M. E., & Valencia, S. (2015). The Genus Anthoceros (Anthocerotaceae, Anthocerotophyta) in Central Mexico. Phytotaxa, 205(4), 215-228.

Ihlenfeldt, H. D. (2004). Pedaliaceae. In Flowering Plants· Dicotyledons: Lamiales (except Acanthaceae including Avicenniaceae) (pp. 307-322). Berlin, Heidelberg: Springer Berlin Heidelberg.

Inocencio, C., Rivera, D., Obón de Castro, C., Alcaraz, F., & Barreña, J. (2006). A systematic revision of Capparis section Capparis (Capparaceae). Annals of the Missouri Botanical Garden, 93(1), 122-149.

Iwashina, T., & Matsumoto, S. (2013). Flavonoid glycosides from the Fern, Schizaea (Schizaeaceae) in south pacific region, and their distribution pattern. Bull. Natl. Mus. Nat. Sci. Ser. B, 39, 195-201.

Jermy, A. C. (1990). Isoetaceae. In Pteridophytes and gymnosperms (pp. 26-31). Berlin, Heidelberg: Springer Berlin Heidelberg.

Jin, J., Boersch, M., Nagarajan, A., Davey, A. K., & Zunk, M. (2020). Antioxidant properties and reported ethnomedicinal use of the genus Echium (Boraginaceae). Antioxidants, 9(8), 722.

Kadereit, J. W. (1987). The taxonomy, distribution and variability of the genus Roemeria Medic.(Papaveraceae). Flora, 179(2), 135-153.

Kadereit, J. W., & Bittrich, V. (Eds.). (2016). Flowering Plants. Eudicots: Aquifoliales, Boraginales, Bruniales, Dipsacales, Escalloniales, Garryales, Paracryphiales, Solanales (except Convolvulaceae), Icacinaceae, Metteniusaceae, Vahliaceae (Vol. 14). Springer.

Kadereit, J. W., Schwarzbach, A. E., & Jork, K. B. (1997). The phylogeny of Papaver sl (Papaveraceae): polyphyly or monophyly?. Plant Systematics and Evolution, 204, 75-98.

Kagame, S. P., Gichira, A. W., Chen, L. Y., & Wang, Q. F. (2021). Systematics of Lobelioideae (Campanulaceae): review, phylogenetic and biogeographic analyses. PhytoKeys, 174, 13.

Kamel, S., Ayed, S., & Cherif, M. (2010). Identification of Tunisian Barley Lines Tolerant to Both Net Botch and Scald in the Adult Stage. Tunisian Plant Science and Biotechnology II. The African Journal of Plant Science and Biotechnology, 4, 77-80.

Kamenetsky, R. (1996). Life cycle and morphological features of Allium L. species in connection with geographical distribution. Bocconea, 5, 251-257.

Kandemir, N., Çelik, A., Shah, S. N., & Razzaq, A. (2020). Comparative micro-anatomical investigation of genus Heliotropium (Boraginaceae) found in Turkey. Flora, 262, 151495.

Karl, R., & Strid, A. (2009). Bongardia chrysogonum (Berberidaceae) rediscovered on the East Aegean island of Chios. Phytologia Balcanica, 15, 337-342.

Kaur, N., Kaur, B., & Sirhindi, G. (2017). Phytochemistry and pharmacology of Phyllanthus niruri L.: a review. Phytotherapy research, 31(7), 980-1004.

Kenicer, G. (2005). Legumes of the World. En G. Lewis, B. Schrire, B. MacKinder & M. Lock (Eds.), Royal Botanic Gardens, Kew. ISBN 1 900 34780 6. £55.00 (hardback). Edinburgh Journal of Botany, 62(3), 195-196. https://doi.org/10.1017/S0960428606190198

Khan, I., Najeebullah, S., Ali, M., & Shinwari, Z. K. (2016). Phytopharmacological and ethnomedicinal uses of the Genus Berberis (Berberidaceae): A review. Tropical Journal of Pharmaceutical Research, 15(9), 2047-2057.

Kim, H., & Heo, K. (2025). Embryology of Menyanthes (Menyanthaceae): its description and taxonomic implications. Nordic Journal of Botany, e04575.

Kim, K. J. (1998). A new species of Fontanesia (Oleaceae) from China and taxonomic revision of the genus. Journal of Plant Biology, 41, 142-145.

Kim, K. J., & Jansen, R. K. (1998). A chloroplast DNA phylogeny of lilacs (Syringa, Oleaceae): plastome groups show a strong correlation with crossing groups. American Journal of Botany, 85(9), 1338-1351.

Knapp, S., & Vorontsova, M. S. (2013). From introduced American weed to Cape Verde Islands endemic: the case of Solanum rigidum Lam. (Solanaceae, Solanum subgenus Leptostemonum). PhytoKeys, 25, 35-46. https://doi.org/10.3897/phytokeys.25.4692

Kokwaro, J. O. (1986). Anacardiaceae. In Flora of Tropical East Africa. Royal Botanic Gardens, Kew.

Korall, P., & Pryer, K. M. (2014). Global biogeography of scaly tree ferns (Cyatheaceae): evidence for Gondwanan vicariance and limited transoceanic dispersal. Journal of biogeography, 41(2), 402-413.

Krakau, U. K., Liesebach, M., Aronen, T., Lelu-Walter, M. A., & Schneck, V. (2013). Scots pine (Pinus sylvestris L.). In Forest tree breeding in Europe: Current state-of-the-art and perspectives (pp. 267-323). Dordrecht: Springer Netherlands.

Kramer, K. U. (1993). Distribution patterns in major pteridophyte taxa relative to those of angiosperms. Journal of Biogeography, 287-291.

Kubitzki, K. (2003). Moringaceae. In Flowering Plants· Dicotyledons: Malvales, Capparales and Non-betalain Caryophyllales (pp. 312-314). Berlin, Heidelberg: Springer Berlin Heidelberg.

Kubitzki, K. (2010). Coriariaceae. In Flowering Plants. Eudicots: Sapindales, Cucurbitales, Myrtaceae (pp. 105-108). Berlin, Heidelberg: Springer Berlin Heidelberg.

Kujawska, M., & Svanberg, I. (2019). From medicinal plant to noxious weed: Bryonia alba L.(Cucurbitaceae) in northern and eastern Europe. Journal of ethnobiology and ethnomedicine, 15, 1-12.

Kumar, N., Kumar, R., & Kishore, K. (2013). Onosma L.: A review of phytochemistry and ethnopharmacology. Pharmacognosy reviews, 7(14), 140.

Kunzmann, L., Kvaček, Z., Mai, D. H., & Walther, H. (2009). The genus Taxodium (Cupressaceae) in the Palaeogene and Neogene of Central Europe. Review of Palaeobotany and Palynology, 153(1-2), 153-183.

Lammers, T. G. (2011). Revision of the Infrageneric Classification of Lobelia L. (Campanulaceae: Lobelioideae). Annals of the Missouri Botanical Garden, 98(1), 37-62. https://doi.org/10.3417/2007150

Långström, E., & Oxelman, B. (2003). Phylogeny of Echiochilon (Echiochileae, Boraginaceae) based on ITS sequences and morphology. Taxon, 52(4), 725-735.

Larridon, I., Tanaka, N., Liang, Y., Phillips, S. M., Barfod, A. S., Cho, S. H., ... & Ito, Y. (2019). First molecular phylogenetic insights into the evolution of Eriocaulon (Eriocaulaceae, Poales). Journal of plant research, 132, 589-600.

Larsen, K. (2002). Caryophyllaceae. Flora Malesiana-Series 1, Spermatophyta, 16(1), 1-51.

Lavender, D. P., & Hermann, R. K. (2014). Douglas-fir: the genus Pseudotsuga.

Lehnert, M., Monjau, T., & Rosche, C. (2024). Synopsis of Osmunda (royal ferns; Osmundaceae): towards reconciliation of genetic and biogeographic patterns with morphologic variation. Botanical Journal of the Linnean Society, 205(4), 341-364.

Leistner, O.A. (2005) Seed plants of southern tropical Africa: families and genera SABONET Report No. 26 SABONET, Pretoria

Léonard, J. (1989). Révision du genre africain Martretia Beille (Euphorbiaceae) et la nouvelle tribu des Martretieae. Bulletin du Jardin botanique national de Belgique/Bulletin van de Nationale Plantentuin van Belgie, 319-332.

LePage, B. A., & Basinger, J. F. (1995). The evolutionary history of the genus Larix (Pinaceae). Proceedings of an international symposium.

Lewis, G., Schrire, B., MacKinder, B., & Lock, M. (Eds.). (2005). Legumes of the world. Royal Botanic Gardens, Kew.

Lézine, A. M., & Casanova, J. (1991). Correlated oceanic and continental records demonstrate past climate and hydrology of North Africa (0-140 ka). Geology, 19(4), 307-310.

Li, Y., Li, X., Nie, S., Zhang, M., Yang, Q., Xu, W., ... & Wang, X. (2024). Reticulate evolution of the tertiary relict Osmanthus. The Plant Journal, 117(1), 145-160.

Lindelof, K., Lindo, J. A., Zhou, W., Ji, X., & Xiang, Q. Y. (2020). Phylogenomics, biogeography, and evolution of the blue or white-fruited dogwoods (Cornus)—Insights into morphological and ecological niche divergence following intercontinental geographic isolation. Journal of Systematics and Evolution, 58(5), 604-645.

Liu, X. I. N. G., Gituru, W. R., & Wang, Q. F. (2004). Distribution of basic diploid and polyploid species of Isoetes in East Asia. Journal of biogeography, 31(8), 1239-1250.

Lovett, P. N., & Haq, N. (2000). Evidence for anthropic selection of the Sheanut tree (Vitellaria paradoxa). Agroforestry systems, 48(3), 273-288.

Lu, H., Zheng, Y., Zhao, T., Tang, L., Zhang, F., & Xie, W. (2025). The potential spatiotemporal distribution patterns of Avena nuda and Avena sativa from global perspective provide new insights for the cultivation of commonly cultivated oats. BMC Plant Biology, 25(1), 1-19.

Lu, L., Wen, J., & Chen, Z. (2012). A combined morphological and molecular phylogenetic analysis of Parthenocissus (Vitaceae) and taxonomic implications. Botanical Journal of the Linnean Society, 168(1), 43-63.

Lupton, D., & Skeffington, M. S. (2020). A review of the ecology and status of the Kerry Lily Simethis mattiazzii (S. planifolia) Asphodelaceae in Ireland. British & Irish Botany, 2(4), 309-334.

Ma, X. Y., Xu, H., Cao, Z. Y., Shu, L., & Zhu, R. L. (2022). Will climate change cause the global peatland to expand or contract? Evidence from the habitat shift pattern of Sphagnum mosses. Global Change Biology, 28(21), 6419-6432.

Madeira, P. T., Pemberton, R. W., & Center, T. D. (2008). A molecular phylogeny of the genus Lygodium (Schizaeaceae) with special reference to the biological control and host range testing of Lygodium microphyllum. Biological Control, 45(3), 308-318.

Malcomber, S. T. (2002). Phylogeny of Gaertnera Lam.(Rubiaceae) based on multiple DNA markers: evidence of a rapid radiation in a widespread, morphologically diverse genus. Evolution, 56(1), 42-57.

Manchester, S. R. (1979). Triplochitioxylon (Sterculiaceae): A new genus of wood from the Eocene of Oregon and its bearing on xylem evolution in the extant genus Triplochiton. American Journal of Botany, 66(6), 699-708.

Manchester, S. R., Chen, Z. D., Lu, A. M., & Uemura, K. (2009). Eastern Asian endemic seed plant genera and their paleogeographic history throughout the Northern Hemisphere. Journal of Systematics and Evolution, 47(1), 1-42.

Mannino, A. M., Menéndez, M., Obrador, B., Sfriso, A., & Triest, L. (2015). The genus Ruppia L.(Ruppiaceae) in the Mediterranean region: an overview. Aquatic Botany, 124, 1-9.

Mao, L., Huang, K., & Huang, H. (2024). Introduction to the special issue: Pollen diversity, vegetation history and range shift in the (sub) tropics through the Cenozoic. Review of Palaeobotany and Palynology, 105277.

Marcussen, T., & Meseguer, A. S. (2017). Species-level phylogeny, fruit evolution and diversification history of Geranium (Geraniaceae). Molecular Phylogenetics and Evolution, 110, 134-149.

Martin, M. D., Quiroz-Claros, E., Brush, G. S., & Zimmer, E. A. (2018). Herbarium collection-based phylogenetics of the ragweeds (Ambrosia, Asteraceae). Molecular Phylogenetics and Evolution, 120, 335-341.

Martínez, I., González-Taboada, F., Wiegand, T., Camarero, J. J., & Gutiérrez, E. (2012). Dispersal limitation and spatial scale affect model based projections of Pinus uncinata response to climate change in the P yrenees. Global Change Biology, 18(5), 1714-1724.

Martínez, S. R. (1984). Sobre el género" Cosentinia" en la Península Ibérica. In Anales del Jardin Botánico de Madrid (Vol. 41, No. 1, p. 196). Real Jardín Botánico.

Mauri, A., Di Leo, M., De Rigo, D., & Caudullo, G. (2016). Pinus halepensis and Pinus brutia in Europe: distribution, habitat, usage and threats. European atlas of forest tree species, 122-123.

Melamed, D., Segarra-Moragues, J. G., Puche, F., Garcia, C. A., & Sérgio, C. (2021). On the synonymization of Acaulon longifolium Herrnst. & Heyn with Acaulon fontiquerianum Casas & Sérgio (Pottiaceae). Cryptogamie, Bryologie, 42(18), 239-248.

Mellano, M. G., Beccaro, G. L., Donno, D., Marinoni, D. T., Boccacci, P., Canterino, S., ... & Bounous, G. (2012). Castanea spp. biodiversity conservation: collection and characterization of the genetic diversity of an endangered species. Genetic Resources and Crop Evolution, 59, 1727-1741.

Meng, H. H., Jacques, F. M., Su, T., Huang, Y. J., Zhang, S. T., Ma, H. J., & Zhou, Z. K. (2014). New biogeographic insight into Bauhinia sl (Leguminosae): integration from fossil records and molecular analyses. BMC evolutionary biology, 14, 1-14.

Mennega, A. M. (1997). Wood anatomy of the Hippocrateoideae (Celastraceae). Iawa Journal, 18(4), 331-368.

Metzgar, J. S., Alverson, E. R., Chen, S., Vaganov, A. V., & Ickert-Bond, S. M. (2013). Diversification and reticulation in the circumboreal fern genus Cryptogramma. Molecular Phylogenetics and Evolution, 67(3), 589-599.

Meyer, S. E. (2008). Artemisia. The woody plant seed manual. Washington (DC): USDA Forest Service Agriculture Handbook, 727, 274-280.

Milliken, W., Klitgaard, B., & Baracat, A. (Eds.). (2010). Neotropikey: Interactive key and information resources for flowering plants of the Neotropics. Retrieved from http://www.kew.org/science/tropamerica/neotropikey.htm

Miyamoto, F., Akiyama, S., Wu, S. K., & Ohba, H. (2002). New and noteworthy species of Bistorta (Polygonaceae) from the Sino-Himalayan Region. Bull Natl Sci Mus Tokyo Ser B, 28, 141-148.

Mo, X., Zhou, Y., Zhan, M., Zhang, Y., Liu, J., Quang, H., & Dong, L. (2025). A review of the traditional uses, phytochemistry, pharmacology and toxicity for the genus Geum (Rosaceae). Fitoterapia, 180, 106333.

Molnar, T. J. (2011). Corylus. In C. Kole (Ed.), Wild crop relatives: Genomic and breeding resources of forest trees (Vol. 10, pp. 15-48). Springer-Verlag.

Montazerolghaem, S., Rahiminejad, M. R., Mozaffarian, V., & Susanna, A. (2016). Taxonomic notes on the genus Echinops (Compositae, Cardueae–Echinopsinae) in Iran. Phytotaxa, 263(2), 81-97.

Morales, R. (1998). Filipendula Mill. Flora iberica, 6.

Muhoya, F. K., Kadima, J. N., Ranarivelo, N., Frédérich, M., Hubert, P., & Marini Djang'eing'a, R. (2017). Preliminary phytochemical content and antidiabetic potential investigations of Panda oleosa (Pierre) used in Kisangani Areas. American Journal of Analytical Chemistry, 8(9), 564-581.

Murshida, C., & Robi, A. J. (2025). Pollen morphology of Rotala L.(Lythraceae) from South India and its taxonomic significance. Palynology, 2517036.

Nagalingum, N. S., Nowak, M. D., & Pryer, K. M. (2008). Assessing phylogenetic relationships in extant heterosporous ferns (Salviniales), with a focus on Pilularia and Salvinia. Botanical Journal of the Linnean Society, 157(4), 673-685.

Namoff, S., Luke, Q., Jiménez, F., Veloz, A., Lewis, C. E., Sosa, V., ... & Francisco-Ortega, J. (2010). Phylogenetic analyses of nucleotide sequences confirm a unique plant intercontinental disjunction between tropical Africa, the Caribbean, and the Hawaiian Islands. Journal of plant research, 123, 57-65.

Neuba, D. F. R., Malan, D. F., & Kouadio, Y. L. (2014). Notes sur le genre Africain Leptactina Hook. f.(Rubiaceae, Pavetteae). Adansonia, 36(1), 121-153.

Nickrent, D. L. (2020). Parasitic angiosperms: how often and how many?. Taxon, 69(1), 5-27.

Nickrent, D. L., Su, H. J., Lin, R. Z., Devkota, M. P., Hu, J. M., & Glatzel, G. (2021). Examining the needle in the haystack: Evolutionary relationships in the mistletoe genus Loranthus (Loranthaceae). Systematic Botany, 46(2), 403-415.

Nimmakayala, P., Vajja, G., Reddy, U.K. (2011). Ipomoea. In: Kole, C. (eds) Wild Crop Relatives: Genomic and Breeding Resources. Springer, Berlin, Heidelberg

Nogueira, I. (1977). The genus Stoebe (Compositae) in the Flora Zambesiaca area and Angola. Boletim da Sociedade Broteriana, 127.

Nooteboom, H. (1962). Generic delimitation in Simaroubaceae tribus Simaroubeae and a conspectus of the genus Quassia L. Blumea: Biodiversity, Evolution and Biogeography of Plants, 11(2), 509-528.

Norscia, I., & Borgognini-Tarli, S. M. (2006). Ethnobotanical reputation of plant species from two forests of Madagascar: A preliminary investigation. South African Journal of Botany, 72(4), 656-660.

Nowicke, Joan W., and Masamichi Takahashi. "Pollen morphology, exine structure and systematics of Acalyphoideae (Euphorbiaceae), part 4: tribes Acalypheae pro parte (Erythrococca, Claoxylon, Claoxylopsis, Mareya, Mareyopsis, Discoclaoxylon, Micrococca, Amyrea, Lobanilia, Mallotus, Deuteromallotus, Cordemoya, Cococceras, Trewia, Neotrewia, Rockinghamia, Octospermum, Acalypha, Lasiococca, Spathiostemon, Homonoia), Plukenetieae (Haematostemon, Astrococcus, Angostyles, Romanoa, Eleutherostigma, Plukenetia, Vigia, Cnesmone, Megistostigma ...." Review of Palaeobotany and Palynology 121.3-4 (2002): 231-336.

Nuñez, C. L., Clark, J. S., & Poulsen, J. R. (2019). Afrotropical Tree Communities May Have Distinct Responses to Forecasted Climate Change. bioRxiv, 823724.

Obbard, D. J., Harris, S. A., Buggs, R. J., & Pannell, J. R. (2006). Hybridization, polyploidy, and the evolution of sexual systems in Mercurialis (Euphorbiaceae). Evolution, 60(9), 1801-1815.

Oberprieler, C., Himmelreich, S., & Vogt, R. (2007). A new subtribal classification of the tribe Anthemideae (Compositae). Willdenowia, 37(1), 89-114.

Øllgaard, B. (1990). Lycopodiaceae. In Pteridophytes and gymnosperms (pp. 31-39). Berlin, Heidelberg: Springer Berlin Heidelberg.

Öztürk, M., Çelik, A., Güvensen, A., & Hamzaoğlu, E. (2008). Ecology of tertiary relict endemic Liquidambar orientalis Mill. forests. Forest Ecology and Management, 256(4), 510-518.

Padgett, D. J. (2007). A monograph of nuphar (nymphaeaceae) 1. Rhodora, 109(937), 1-95.

Parker, V. T., Rodriguez, C. Y., Wechsler, G., & Vasey, M. C. (2020). Allopatry, hybridization, and reproductive isolation in Arctostaphylos. American Journal of Botany, 107(12), 1798-1814.

Patiño, J., & Vanderpoorten, A. (2018). Bryophyte biogeography. Critical Reviews in Plant Sciences, 37(2-3), 175-209.

Pellegrin, F. (1955). Sapindacées nouvelles du Gabon: Chytranthus, Hook. f. Pancovia Wild., Pseudopancovia Pellegr. et Placodiscus Radlk. Bulletin de la Société Botanique de France, 102(4-6), 226-229.

Persson, K., Petersen, G., del Hoyo, A., Seberg, O., & Jørgensen, T. (2011). A phylogenetic analysis of the genus Colchicum L.(Colchicaceae) based on sequences from six plastid regions. Taxon, 60(5), 1349-1365.

Philbrick, C. T., & Les, D. H. (2000). Phylogenetic studies in Callitriche: implications for interpretation of ecological, karyological and pollination system evolution. Aquatic Botany, 68(2), 123-141.

Phillips, E. P. (1922). The Genus Bersama. Bothalia, 1(1), 33-38.

Phoon, S. N. (2012). A new variety of Strombosia ceylanica (Olacaceae) from Malaysia. Kew Bulletin, 67(2), 191-203.

Pijut, P. M. (2008). Carpinus. Woody plant seed manual, Agriculture Handbook, 727, 328-332.

Pope, G. V., Polhill, R. M., & Martins, E. S. (2006). Flora Zambesiaca (Vol. 9, Part 3). Polygonaceae-Myriaceae. Royal Botanic Gardens, Kew.

Pope, G. V., Polhill, R. M., & Martins, E. S. (2006). Polygonaceae–Myriaceae. In Flora Zambesiaca (Vol. 9, Part 3). Royal Botanic Gardens, Kew.

Posluszny, U. (1983). Re-evaluation of certain key relationships in the Alismatidae: floral organogenesis of Scheuchzeria palustris (Scheuchzeriaceae). American Journal of Botany, 70(6), 925-933.

Prance, G. T., & Jongkind, C. C. (2015). A revision of African Lecythidaceae. Kew Bulletin, 70(1), 6.

Pringle, J. S. (2014). Morphological characteristics of the family Gentianaceae. In The Gentianaceae-Volume 1: Characterization and Ecology (pp. 1-12). Berlin, Heidelberg: Springer Berlin Heidelberg.

Proctor, G. R. (2012). Flora of the Cayman Islands (2nd ed.). Royal Botanic Gardens, Kew.

Puff, C. (2012). A biosystematic study of the African and Madagascan Rubiaceae-Anthospermeae (Vol. 3). Springer Science & Business Media.

Puff, C., Robbrecht, E., Buchner, R., & De Block, P. (1996). A survey of secondary pollen presentation. Opera Bot. Belg

Qiao, D., Wang, J., Lu, M. H., Xin, C., Chai, Y., Jiang, Y., ... & Chen, Q. J. (2023). Optimized prime editing efficiently generates heritable mutations in maize. Journal of Integrative Plant Biology, 65(4), 900-906.

Quézel, P. (1978). Analysis of the flora of Mediterranean and Saharan Africa. Annals of the Missouri Botanical Garden, 479-534.

Radcliffe-Smith, A. (1987). Euphorbiaceae. In Flora of Tropical East Africa. A. A. Balkema.

Rakotoarivelo, N., Andriambololonera, S., Phillipson, P. B., & Callmander, M. W. (2014). Une espèce nouvelle du genre Nesogordonia Baill.(Malvaceae) endémique du Nord de Madagascar. Candollea, 69(2), 165-170.

Ran, J. H., Wei, X. X., & Wang, X. Q. (2006). Molecular phylogeny and biogeography of Picea (Pinaceae): implications for phylogeographical studies using cytoplasmic haplotypes. Molecular Phylogenetics and Evolution, 41(2), 405-419.

Ranjbar, M., & Khalvati, S. (2019). World checklist of Moltkia (Boraginaceae) with notes on types. Phytotaxa, 408(3), 143-160.

Retief, E., & Van Wyk, A. E. (1997). Palynology of southern African Boraginaceae: the genera Lobostemon, Echiostachys and Echium. Grana, 36(5), 271-278.

Reveal, J. L. (1978). Distribution and phylogeny of Eriogonoideae (Polygonaceae). Great Basin Naturalist Memoirs, 169-190.

Robbrecht, E., De Block, P., & Van Hecke, P. (1991). Incomplete rheophytic speciation in Pouchetia (African Rubiaceae—Hypobathreae). Belgian Journal of Botany, 137-151.

Robson, N. K. B. (1963). Ochnaceae. In Flora Zambesiaca (Vol. 2, Part 1). Crown Agents for Oversea Governments and Administrations.

Roché, B. F., & Roché, C. T. (1992). Identification, Introduction, Distribution, Ecology, and Economics of Centaurea Species. In Noxious Range Weeds. CRC Press. https://doi.org/10.1201/9780429046483-28

Romo, A. (2021). Apomictic species of Alchemilla from the High Atlas Mountains: revision of the genus Alchemilla (Rosaceae) in Morocco.

Ronse, A. C., Popper, Z. A., Preston, J. C., & Watson, M. F. (2010). Taxonomic revision of European Apium L. sl: Helosciadium WDJ Koch restored. Plant Systematics and Evolution, 287(1), 1-17.

Rothfels, C. J., Sundue, M. A., Kuo, L. Y., Larsson, A., Kato, M., Schuettpelz, E., & Pryer, K. M. (2012). A revised family–level classification for eupolypod II ferns (Polypodiidae: Polypodiales). Taxon, 61(3), 515-533.

Roux, J. P., Shaffer-Fehre, M., & Verdcourt, B. (2007). Dryopteridaceae. In Flora of Tropical East Africa. Royal Botanic Gardens, Kew.

Rugini, E., De Pace, C., Gutiérrez-Pesce, P., Muleo, R. (2011). Olea. In: Kole, C. (eds) Wild Crop Relatives: Genomic and Breeding Resources. Springer, Berlin, Heidelberg

Sadiq Wada, A., Julde, S. A. M., Borodo, S. B., Ahmad, M. H., Malami, S., & Yaro, A. H. (2022). Phytochemistry, ethnomedicinal uses and pharmacological activity of Diodia scandens; a review of current scientific literature. Egyptian Journal of Basic and Applied Sciences, 9(1), 533-541.

Salinas, M. J., Romero, A. T., Blanca, G., Herrán, R. D. L., Garrido-Ramos, M., Ruíz-Rejón, C., ... & Suárez, V. (2003). Contribution to the taxonomy and phylogeny of Sarcocapnos DC.(Fumariaceae). Plant Systematics and Evolution, 237, 153-164.

Sang, T. (1995). Phylogeny and biogeography of Paeonia (Paeoniaceae). The Ohio State University.

Sarika-Hatzinikolaou, M., Koumpli-Sovantzi, L., & Yannitsaros, A. (1994). Myriophyllum alterniflorum DC. (Haloragaceae), a new record for the Greek flora. Phyton, Annales rei botanicae (Horn, Austria), 34, 243-246.

Schneller, J. J. (1990). Salviniaceae. In Pteridophytes and Gymnosperms (pp. 256-258). Berlin, Heidelberg: Springer Berlin Heidelberg.

Schüler, L., & Hemp, A. (2016). Atlas of pollen and spores and their parent taxa of Mt Kilimanjaro and tropical East Africa. Quaternary International, 425, 301–386. https://doi.org/10.1016/j.quaint.2016.07.038

Semerdjieva, I., Petrova, G., Yankova-Tsvetkova, E., Doncheva, T., Kostova, N., Nikolova, R., & Zheljazkov, V. D. (2020). Genetic diversity, reproductive capacity and alkaloids content in three endemic Alkanna species. PLoS One, 15(6), e0233516.

Semwal, D. K., Badoni, R., Semwal, R., Kothiyal, S. K., Singh, G. J. P., & Rawat, U. (2010). The genus Stephania (Menispermaceae): chemical and pharmacological perspectives. Journal of Ethnopharmacology, 132(2), 369-383.

Shaik, Z. (2024). Extensions of the Multispecies Coalescent in Bayesian Phylogenetics: A Study of the Southern African-centred Stoebe Clade (Gnaphalieae: Asteraceae).

Shalimov, A. P., Shrestha, N., & Zhang, X. C. (2017). Taxonomic study of the genus Huperzia Bernh.(Lycopodiaceae) in the Pan-Himalayan region. Indian Fern Journal, 34(1-2), 130-168.

Simpson, M. G. (2019). Evolution and diversity of woody and seed plants. In M. G. Simpson (Ed.), Plant systematics (3rd ed., pp. 131–165). Academic Press. https://doi.org/10.1016/B978-0-12-812628-8.50005-5

Siniscalchi, C. M., Loeuille, B., Funk, V. A., Mandel, J. R., & Pirani, J. R. (2019). Phylogenomics yields new insight into relationships within Vernonieae (Asteraceae). Frontiers in Plant Science, 10, 1224.

Sleumer, H. O. (1980). Flacourtiaceae. Flora Neotropica, 1-499.

Smith, S. A., & Donoghue, M. J. (2010). Combining historical biogeography with niche modeling in the Caprifolium clade of Lonicera (Caprifoliaceae, Dipsacales). Systematic biology, 59(3), 322-341.

Sofiah, S., & Sulistyaningsih, L. D. (2019). The diversity of Smilax (Smilacaceae) in Besiq-Bermai and bontang forests, east Kalimantan, Indonesia. Biodiversitas Journal of Biological Diversity, 20(1).

Soltis, D. E., Tago-Nakazawa, M., Xiang, Q. Y., Kawano, S., Murata, J., Wakabayashi, M., & Hibsch-Jetter, C. (2001). Phylogenetic relationships and evolution in Chrysosplenium (Saxifragaceae) based on matK sequence data. American Journal of Botany, 88(5), 883-893.

Song, Y. G., Li, Y., Meng, H. H., Fragnière, Y., Ge, B. J., Sakio, H., ... & Kozlowski, G. (2020). Phylogeny, taxonomy, and biogeography of Pterocarya (Juglandaceae). Plants, 9(11), 1524.

Song, Y., Jiang, F., Shi, J., Wang, C., Xiang, N., & Zhu, S. (2022). Phylogenomics reveals the evolutionary history of phytolacca (Phytolaccaceae). Frontiers in Plant Science, 13, 844918.

Sonké, B., Simo, M., & Dessein, S. (2009). Synopsis of the genus Mitriostigma (Rubiaceae) with a new monocaulous species from south Cameroon. Nordic Journal of Botany, 27(4), 305-312.

Sprague, E. F. (1962). Pollination and evolution in Pedicularis (Scrophulariaceae). Aliso: A Journal of Systematic and Floristic Botany, 5(2), 181-209.

Stannard, B. (2000). Simaroubaceae. In Flora of Tropical East Africa. Royal Botanic Gardens, Kew.

Staples, G. W. (2007). A synopsis of Lepistemon (Convolvulaceae) in Australasia. Kew Bulletin, 223-232.

Stoffelen, P., Robbrecht, E., & Smets, E. (1996). A revision of Corynanthe and Pausinystalia (African Rubiaceae-Coptosapelteae). Botanical Journal of the Linnean Society, 120(4), 287-326.

Stride, G., Nylinder, S., & Swenson, U. (2014). Revisiting the biogeography of Sideroxylon (Sapotaceae) and an evaluation of the taxonomic status of Argania and Spiniluma. Australasian Systematic Botany, 27(2), 104-118.

Sun, M., & Lin, Q. (2010). A revision of Elaeagnus L.(Elaeagnaceae) in mainland China. Journal of Systematics and Evolution, 48(5), 356-390.

Sun, Q., Wang, N., Xu, W., & Zhou, H. (2021). Genus Ribes Linn.(Grossulariaceae): A comprehensive review of traditional uses, phytochemistry, pharmacology and clinical applications. Journal of Ethnopharmacology, 276, 114166.

Sunderland, T. C., Cunningham, A. B., Tchoundjeu, Z., Ngo-Mpeck, M. L., & Yohimbe, L. S. (2004). Pausinystalia johimbe. The key non-timber forest products of Central Africa: State of the Knowledge, 122, 121-140.

Surendran, S., Raju, R., Prasannan, P., & Surendran, A. (2021). A comprehensive review on ethnobotany, phytochemistry and pharmacology of Rauvolfia L.(Apocynaceae). The Botanical Review, 87(3), 311-376.

Swenson, U., & Bartish, I. V. (2002). Taxonomic synopsis of Hippophae (Elaeagnaceae). Nordic Journal of Botany, 22(3), 369-374.

Szalontai, B., Stranczinger, S., Mesterházy, A., Scribailo, R. W., Les, D. H., Efremov, A. N., Jacono, C. C., Kipriyanova, L. M., Kaushik, K., Laktionov, A. P., Terneus, E., & Csiky, J. (2018). Molecular phylogenetic analysis of Ceratophyllum L. taxa: A new perspective. Botanical Journal of the Linnean Society, 188(2), 161–172. https://doi.org/10.1093/botlinnean/boy057

Tang, Z.X. et al. (2011). Secale. In: Kole, C. (eds) Wild Crop Relatives: Genomic and Breeding Resources. Springer, Berlin, Heidelberg.

Tavakkoli, S., Kazempour Osaloo, Sh., & Maassoumi, A. A. (2008). Morphological cladistic analysis of Calligonum and Pteropyrum (Polygonaceae) in Iran. Iran J. Bot., 14(2), 117-125. Tehran.

Tawfeek, N., Mahmoud, M. F., Hamdan, D. I., Sobeh, M., Farrag, N., Wink, M., & El-Shazly, A. M. (2021). Phytochemistry, pharmacology and medicinal uses of plants of the genus Salix: An updated review. Frontiers in pharmacology, 12, 593856.

Teixeira, L. J., Araújo, M. D. D. F., Deccache, L. S. J., & de Fraga, C. N. (2025). When nomenclature and morphology unravel an enigma: the true identity of Ouratea crassinervia (Ochnaceae). Plant Ecology and Evolution, 158(2), 237.

Tharakan, S. T. (2021). Phytochemical and pharmacological properties of five different species of Jasminum. Plant Arch, 21(2), 126-136.

Thulin, M., et al. (2008). Flora of Somalia, Vol. 1-4. https://plants.jstor.org/collection/FLOS

Timberlake, J. R., & Martins, E. S. (2009). Flora Zambesiaca (Vol. 12, Part 2). Royal Botanic Gardens, Kew.

Timberlake, J. R., & Martins, E. S. (Eds.). (2013). Flora Zambesiaca (Vol. 8, Part 5). University of Chicago Press. ISBN: 978-1842464120

Tomooka, N., Kaga, A., Isemura, T., Vaughan, D. (2011). Vigna. In: Kole, C. (eds) Wild Crop Relatives: Genomic and Breeding Resources. Springer, Berlin, Heidelberg

Tomou, E. M., Lytra, K., Rallis, S., Tzakos, A. G., & Skaltsa, H. (2022). An updated review of genus Cistus L. since 2014: traditional uses, phytochemistry, and pharmacological properties. Phytochemistry Reviews, 21(6), 2049-2087.

Townsend, C. C. (1989). Flora of Tropical East Africa-Unibelliferae (Vol. 187). CRC Press.

Townsend, C. C., Melzheimer, V., Kandemir, A., Ghazanfar, S. A., Haloob, A., Edmondson, J. R., Akeroyd, J. R., Sukhorukov, A. P., Aellen, P., & Alizzi, H. A. (2016). Flora of Iraq. Volume 5, Part 1. Elatinaceae to Sphenocleaceae. Published on behalf of the Ministry of Agriculture, Republic of Iraq by Royal Botanic Gardens, Kew. Richmond, Surrey, United Kingdom: Royal Botanic Gardens, Kew. ISBN: 978-1-84246-594-3.

Tremetsberger, K., Gemeinholzer, B., Zetzsche, H., Blackmore, S., Kilian, N., & Talavera, S. (2013). Divergence time estimation in Cichorieae (Asteraceae) using a fossil-calibrated relaxed molecular clock. Organisms Diversity & Evolution, 13, 1-13.

Tryon, A. F. (1957). A revision of the fern genus Pellaea section Pellaea. Annals of the Missouri Botanical Garden, 44(2), 125-193.

Tsymbalyuk, Z. M., Çelenk, S., Bell, C. D., Nitsenko, L. M., & Mosyakin, S. L. (2022). Comparative palynomorphological study of the genus Symphoricarpos (Caprifoliaceae): exine sculpture and implications for evolution. Palynology, 46(4), 1-14.

Turki, Z. A. (2007). The genus Ammannia L.(Lythraceae) in Egypt. Flora Mediterranea, 17, 97-114.

Tütüncü, M. (2020). In vitro culture of primula: a review. International Journal of Agricultural and Natural Sciences, 13(2), 118-125.

Utteridge, T. M. A., & Jennings, L. V. S. (2022). Trees of New Guinea. Royal Botanic Gardens, Kew.

Utteridge, T., & Bramley, G. (2020). The Kew Tropical Plant Families Identification Handbook (2nd ed.). Kew Publishing, Royal Botanic Gardens, Kew.

Valcárcel, V., Guzmán, B., Medina, N. G., Vargas, P., & Wen, J. (2017). Phylogenetic and paleobotanical evidence for late Miocene diversification of the Tertiary subtropical lineage of ivies (Hedera L., Araliaceae). BMC Evolutionary Biology, 17, 1-14.

Van der Plas, F. (1972). Lemnaceae. Flora Malesiana-Series 1, Spermatophyta, 7(1), 219-237.

Van Dilst, F. J. H. (1995). Baissea A. DC. Series of revisions of Apocynaceae XXXIX. Bulletin du Jardin botanique national de Belgique/Bulletin van de Nationale Plantentuin van Belgie, 89-178.

Van Wyk, A. E., & Potgieter, M. J. (1994). Two new species of Apodytes (Icacinaceae) from southern Africa. South African Journal of Botany, 60(5), 231-239.

Varol, T., Cetin, M., Ozel, H. B., Sevik, H., & Zeren Cetin, I. (2022). The effects of climate change scenarios on Carpinus betulus and Carpinus orientalis in Europe. Water, Air, & Soil Pollution, 233(2), 45.

Vendramin, G. G., Anzidei, M., Madaghiele, A., & Bucci, G. (1998). Distribution of genetic diversity in Pinus pinaster Ait. as revealed by chloroplast microsatellites. Theoretical and Applied Genetics, 97, 456-463.

Verdcourt, B. (1953). A revision of certain African genera of herbaceous Rubiaceae II. The genus Otomeria Benth. and the new genus Batopedina Verdcourt. Bulletin du Jardin botanique de l'Etat, Bruxelles/Bulletin van den Rijksplantentuin, Brussel, 5-34.

Verstraete, B., Lachenaud, O., Smets, E., Dessein, S., & Sonké, B. (2013). Taxonomy and phylogenetics of Cuviera (Rubiaceae–Vanguerieae) and reinstatement of Globulostylis with the description of three new species. Botanical Journal of the Linnean Society, 173(3), 407-441.

Vilatersana, R., Calleja, J. A., Herrando-Moraira, S., Garcia-Jacas, N., & Susanna, A. (2022). Molecular insights on the conflicting generic boundaries in the Carduncellus-Carthamus complex (Compositae). Taxon, 71(6), 1268-1286.

Viñas, R. A., Caudullo, G., Oliveira, S., & De Rigo, D. (2016). Pinus pinea in Europe: distribution, habitat, usage and threats. European atlas of forest tree species, 204.

Vollesen, K. (2008). Acanthaceae (Part 1). In Flora of Tropical East Africa. Royal Botanic Gardens, Kew.

Vollesen, K. (2008). Acanthaceae (Part 2). In Flora of Tropical East Africa. Royal Botanic Gardens, Kew.

Wala, M., Kołodziejek, J., Mazur, J., & Cienkowska, A. (2021). Reactions of two xeric-congeneric species of Centaurea (Asteraceae) to soils with different pH values and iron availability. PeerJ, 9, e12417.

Walker, C. C. (2023). Laportea Urticaceae. In Dicotyledons: Rosids (pp. 1075-1076). Cham: Springer International Publishing.

Wang, T. X., Del Rio, C., Manchester, S. R., Liu, J., Wu, F. X., Deng, W. Y. D., ... & Zhou, Z. K. (2021). Fossil fruits of Illigera (Hernandiaceae) from the Eocene of central Tibetan Plateau. Journal of Systematics and Evolution, 59(6), 1276-1286.

Wang, T. X., Huang, J., Ding, W. N., Del Rio, C., Su, T., & Zhou, Z. K. (2020). Fossil involucres of Ostrya (Betulaceae) from the early Oligocene of Yunnan and their biogeographic implications. Palaeoworld, 29(4), 752-760.

Wang, Y. F., Li, C. S., Collinson, M. E., Lin, J., & Sun, Q. G. (2003). Eucommia (Eucommiaceae), a potential biothermometer for the reconstruction of paleoenvironments. American Journal of Botany, 90(1), 1-7.

Warf, B. (2014). High points: an historical geography of cannabis. Geographical Review, 104(4), 414-438.

Waswa, E. N., Li, J., Mkala, E. M., Wanga, V. O., Mutinda, E. S., Nanjala, C., ... & Wang, Q. F. (2022). Ethnobotany, phytochemistry, pharmacology, and toxicology of the genus Sambucus L.(Viburnaceae). Journal of Ethnopharmacology, 292, 115102.

Watrin J., Lézine A.M, Hély C. (2009). Plant migration and plant communities at the time of the “green Sahara”. Comptes Rendus. Géoscience, Histoire climatique des déserts d'Afrique et d'Arabie, Volume 341. no. 8-9, pp. 656-670. doi:10.1016/j.crte.2009.06.007.

Webster, J., & Weber, R. (2007). Introduction to fungi. Cambridge university press.

Wei, R., Ebihara, A., Zhu, Y. M., Zhao, C. F., Hennequin, S., & Zhang, X. C. (2018). A total-evidence phylogeny of the lady fern genus Athyrium Roth (Athyriaceae) with a new infrageneric classification. Molecular Phylogenetics and Evolution, 119, 25-36.

Wendel, J. F., Brubaker, C. L., & Seelanan, T. (2010). The origin and evolution of Gossypium. In Physiology of cotton (pp. 1-18). Dordrecht: Springer Netherlands.

Wheeler, J. A. (1998). Molecular phylogenetic analyses of Riccia and Marchantiales. Oregon State University.

White, F. (1983). The Vegetation of Africa: A Descriptive Memoir to Accompany the UNESCO/AETFAT/UNSO Vegetation Map of Africa (3 Plates, Northwestern Africa, Northeastern Africa, and Southern Africa, 1:5,000,000). UNESCO.

Widuri, R., & van Welzen, P. E. (1998). A revision of the genus Cephalomappa (Euphorbiaceae) in Malesia. Reinwardtia, 11(3), 153–184.

Winkworth, R. C., & Donoghue, M. J. (2005). Viburnum phylogeny based on combined molecular data: implications for taxonomy and biogeography. American Journal of Botany, 92(4), 653-666.

Wortley, A. (2004). Systematics of Thomandersia Baill (Doctoral dissertation, University of Oxford).

Xiao, S., Li, S., Huang, J., Wang, X., Wu, M., Karim, R., ... & Su, T. (2024). Influence of climate factors on the global dynamic distribution of Tsuga (Pinaceae). Ecological Indicators, 158, 111533.

Xu, Z., & Deng, M. (2017). Marsileaceae. In Identification and Control of Common Weeds: Volume 2 (pp. 95-97). Dordrecht: Springer Netherlands.

Xu, Z., Chang, L., Xu, Z., & Chang, L. (2017). Hydrocharitaceae. Identification and Control of Common Weeds: Volume 3, 753-763.

Xu, Z., Deng, M., Xu, Z., & Deng, M. (2017). Geraniaceae. Identification and Control of Common Weeds: Volume 2, 629-637.

Yang, Y. Y., Meng, Y., Wen, J., Sun, H., & Nie, Z. L. (2016). Phylogenetic analyses of Searsia (Anacardiaceae) from eastern Asia and its biogeographic disjunction with its African relatives. South African Journal of Botany, 106, 129-136.

Youngblood, A. (2005). Frangula P. Mill. Woody plant seed manual. United States Department of Agriculture, 28.

Yurtseva, O. V., Kuznetsova, O. I., Mavrodieva, M. E., & Mavrodiev, E. V. (2016). What is Atraphaxis L.(Polygonaceae, Polygoneae): cryptic taxa and resolved taxonomic complexity instead of the formal lumping and the lack of morphological synapomorphies. PeerJ, 4, e1977.

Zemagho Mbouzang, L. A. (2016). Phylogeny and Taxonomy of Continental African Sabicea (Rubiaceae) (Doctoral dissertation).

Zhang, H. J., Zhang, X., Landis, J. B., Sun, Y. X., Sun, J., Kuang, T. H., ... & Wang, H. C. (2022). Phylogenomic and comparative analyses of Rheum (Polygonaceae, Polygonoideae). Journal of Systematics and Evolution, 60(6), 1229-1240.

Zhang, M. L., Meng, H. H., Zhang, H. X., Vyacheslav, B. V., & Sanderson, S. C. (2014). Himalayan origin and evolution of Myricaria (Tamaricaeae) in the Neogene. PloS one, 9(6), e97582.

Zhang, M., Hao, X., Sanderson, S. C., Vyacheslav, B. V., Sukhorukov, A. P., & Zhang, X. (2014). Spatiotemporal evolution of Reaumuria (Tamaricaceae) in Central Asia: insights from molecular biogeography. Phytotaxa, 167(1), 89-103.

Zhang, Z., Li, S., & Lian, X. Y. (2010). An overview of genus Aesculus L.: ethnobotany, phytochemistry, and pharmacological activities. Pharmaceutical Crops, 1(1).

Zhou, P., Li, J., Chen, Q., Wang, L., Yang, J., Wu, A., ... & Wu, J. (2021). A comprehensive review of genus Sanguisorba: Traditional uses, chemical constituents and medical applications. Frontiers in Pharmacology, 12, 750165.

Ziyuan, W. A. N. G., Xiuying, C. H. U., & Wei, L. I. (2023). Geographic Distribution and Ecological Adaptability of Fagopyrum Species in Yunnan Province. Journal of Resources and Ecology, 14(6), 1252-1259.

Zizka, A., Carvalho-Sobrinho, J. G., Pennington, R. T., Queiroz, L. P., Alcantara, S., Baum, D. A., ... & Antonelli, A. (2020). Transitions between biomes are common and directional in Bombacoideae (Malvaceae). Journal of Biogeography, 47(6), 1310-1321.

Zomlefer, W. B. (1997). The genera of Tofieldiaceae in the southeastern United States. Harvard Papers in Botany, 2(2), 179-194.

Zumrutdal, E., & Ozaslan, M. (2012). A miracle plant for the herbal pharmacy; henna (Lawsonia inermis).

Zwetsloot, H. J. C. (1981). A revision of Farquharia Stapf and Funtumia Stapf (Apocynaceae).

7. References leaf type (TRY database)

Cornelissen, J. H. C. (1996). An experimental comparison of leaf decomposition rates in a wide range of temperate plant species and types. Journal of Ecology, 84, 573–582.

Fan, Y., Miguez-Macho, G., Jobbágy, E. G., Jackson, R. B., & Otero-Casal, C. (2017). Hydrologic regulation of plant rooting depth. Proceedings of the National Academy of Sciences, 114(40), 10572–10577. https://doi.org/10.1073/pnas.1712381114

Han, W., Chen, Y., Zhao, F.-J., Tang, L., Jiang, R., & Zhang, F. (2012). Floral, climatic and soil pH controls on leaf ash content in China's terrestrial plants. Global Ecology and Biogeography. https://doi.org/10.1111/j.1466-8238.2011.00677.x

He, T., Pausas, J. P., Belcher, C. M., Schwilk, D. W., & Lamont, B. B. (2012). Fire-adapted traits of Pinus arose in the fiery Cretaceous. New Phytologist, 194, 751–759. https://doi.org/10.1111/j.1469-8137.2012.04079.x

Iversen, C. M., McCormack, M. L., Powell, A. S., Blackwood, C. B., Freschet, G. T., Kattge, J., Roumet, C., Stover, D. B., Soudzilovskaia, N. A., Valverde-Barrantes, O. J., Bodegom, P. M., & Violle, C. (2017). A global Fine-Root Ecology Database to address below-ground challenges in plant ecology. New Phytologist, 215, 15–26. https://doi.org/10.1111/nph.14486

Kattge, J., Knorr, W., Raddatz, T., & Wirth, C. (2009). Quantifying photosynthetic capacity and its relationship to leaf nitrogen content for global-scale terrestrial biosphere models. Global Change Biology, 15, 976–991.

Onoda, Y., Westoby, M., Adler, P. B., Choong, A. M. F., Clissold, F. J., Cornelissen, J. H. C., … Yamashita, N. (2011). Global patterns of leaf mechanical properties. Ecology Letters, 14, 301–312.

Onstein, R. E., Carter, R. J., & Xing, Y. (2014). Diversification rate shifts in the Cape Floristic Region: The right traits in the right place at the right time. Perspectives in Plant Ecology, Evolution and Systematics, 16(6), 331–340. https://doi.org/10.1016/j.ppees.2014.08.002

Prentice, I. C., Meng, T., Wang, H., Harrison, S. P., Ni, J., & Wang, G. (2011). Evidence for a universal scaling relationship of leaf CO2 drawdown along a moisture gradient. New Phytologist, 190, 169–180.

Quested, H. M., Cornelissen, J. H. C., Press, M. C., Callaghan, T. V., Aerts, R., Trosien, F., … Jonasson, S. E. (2003). Decomposition of sub-arctic plants with differing nitrogen economies: A functional role for hemiparasites. Ecology, 84, 3209–3221.

Reich, P. B., Oleksyn, J., & Wright, I. J. (2009). Leaf phosphorus influences the photosynthesis-nitrogen relation: A cross-biome analysis of 314 species. Oecologia, 160, 207–212.

White, M. A., Thornton, P. E., Running, S. W., & Nemani, R. R. (2000). Parameterization and sensitivity analysis of the BIOME-BGC terrestrial ecosystem model: Net primary production controls. Earth Interactions, 4, 1–85.

Wirth, C., & Lichstein, J. W. (2009). The imprint of species turnover on old-growth forest carbon balances: Insights from a trait-based model of forest dynamics. In C. Wirth, G. Gleixner, & M. Heimann (Eds.), Old-Growth Forests: Function, Fate and Value (pp. 81–113). Springer.

Wright, I. J., Reich, P. B., Westoby, M., Ackerly, D. D., Baruch, Z., Bongers, F., … Villar, R. (2004). The worldwide leaf economics spectrum. Nature, 428, 821–827.

Yahan Chen, Han, W., Tang, L., Tang, Z., & Fang, J. (2011). Leaf nitrogen and phosphorus concentrations of woody plants differ in responses to climate, soil and plant growth form. Ecography, 34. https://doi.org/10.1111/j.1600-0587.2011.06833.x

8. References plant functional traits (BIEN database)

Aakala, T., Shimatani, K., Abe, T., Kubota, Y., & Kuuluvainen, T. (2016). Crown asymmetry in high latitude forests: disentangling the directional effects of tree competition and solar radiation. Oikos, 125(7), 1035-1043.

Abakumova, M., Zobel, K., Lepik, A., & Semchenko, M. (2016). Plasticity in plant functional traits is shaped by variability in neighbourhood species composition. New Phytologist, 211(2), 455-463.

Ackerly, D. D. (2004). Adaptation, niche conservatism, and convergence: Comparative studies of leaf evolution in the California chaparral. The American Naturalist, 163(5), 654–671.

Améztegui González, A., Paquette, A., Shipley, B., Heym, M., Messier, C., & Gravel, D. (2017). Shade tolerance and the functional trait-demography relationship in temperate and boreal forests.

Anderson-Teixeira, K. J., McGarvey, J. C., Muller-Landau, H. C., Park, J. Y., Gonzalez-Akre, E. B., Herrmann, V., ... & McShea, W. J. (2015). Size-related scaling of tree form and function in a mixed-age forest. Functional Ecology, 29(12), 1587-1602.

Atkinson, R. R., Mockford, E. J., Bennett, C., Christin, P. A., Spriggs, E. L., Freckleton, R. P., ... & Osborne, C. P. (2016). C4 photosynthesis boosts growth by altering physiology, allocation and size. Nature plants, 2(5), 1-5.

Balzotti, C. S., Asner, G. P., Taylor, P. G., Cleveland, C. C., Cole, R., Martin, R. E., ... & Townsend, A. R. (2016). Environmental controls on canopy foliar nitrogen distributions in a Neotropical lowland forest. Ecological Applications, 26(8), 2451-2464.

Beaulieu, J., Doerksen, T., Clément, S., MacKay, J., & Bousquet, J. (2014). Accuracy of genomic selection models in a large population of open-pollinated families in white spruce. Heredity, 113(4), 343-352.

Bezeng, S. B., Davies, J. T., Yessoufou, K., Maurin, O., & Van der Bank, M. (2015). Revisiting Darwin's naturalization conundrum: explaining invasion success of non-native trees and shrubs in southern Africa. Journal of Ecology, 103(4), 871-879.

Bhaskar, R., Dawson, T. E., & Balvanera, P. (2014). Community assembly and functional diversity along succession post-management. Functional Ecology, 28(5), 1256-1265.

Bhattarai, G. P., Meyerson, L. A., Anderson, J., Cummings, D., Allen, W. J., & Cronin, J. T. (2017). Biogeography of a plant invasion: genetic variation and plasticity in latitudinal clines for traits related to herbivory. Ecological Monographs, 87(1), 57-75.

Bonal, D., Sabatier, D., Montpied, P., Tremeaux, D., & Guehl, J.-M. (2000). Interspecific variability of δ13C among trees in rainforests of French Guiana: Functional groups and canopy integration. Oecologia, 124(3), 454–468.

Bufford, J. L., Lurie, M. H., & Daehler, C. C. (2016). Biotic resistance to tropical ornamental invasion. Journal of ecology, 104(2), 518-530.

Burns, J. H., Halpern, S. L., & Winn, A. A. (2007). A test for a cost of opportunism in invasive species in the Commelinaceae. Biological Invasions, 9(2), 213-225.

Carmona, C., Rota, C., AzcA!rate, F., & Peco, B. (2014). More for less: Sampling strategies of plant functional traits across local environmental gradients. In Functional Ecology. Dryad Digital Repository. https://doi.org/doi:10.5061/dryad.53550

Carus, J., Paul, M., & Schröder, B. (2016). Vegetation as self-adaptive coastal protection: Reduction of current velocity and morphologic plasticity of a brackish marsh pioneer. Ecology and Evolution, 6(6), 1579-1589.

Cavender-Bares, J., González-Rodríguez, A., Eaton, D. A., Hipp, A. A., Beulke, A., & Manos, P. S. (2015). Phylogeny and biogeography of the American live oaks (Quercus subsection Virentes): a genomic and population genetics approach. Molecular ecology, 24(14), 3668-3687.

Cornwell, W. K., Schwilk, D. W., & Ackerly, D. D. (2006). A trait-based test for habitat filtering: Convex hull volume. Ecology, 87(6), 1465–1471.

Correia, M., Montesinos, D., French, K., & Rodríguez-Echeverría, S. (2016). Evidence for enemy release and increased seed production and size for two invasive Australian acacias. Journal of Ecology, 104(5), 1391-1399.

Dalponte, M., & Coomes, D. (2016). Tree-centric mapping of forest carbon density from airborne laser scanning and hyperspectral data. In Methods in Ecology and Evolution. Dryad Digital Repository. https://doi.org/doi:10.5061/dryad.hf5rh

de la Riva, E. G., Pérez-Ramos, I. M., Tosto, A., Navarro-Fernández, C. M., Olmo, M., Marañón, T., & Villar, R. (2016). Disentangling the relative importance of species occurrence, abundance and intraspecific variability in community assembly: a trait-based approach at the whole-plant level in Mediterranean forests. Oikos, 125(3), 354-363.

Deraison, H., Badenhausser, I., Börger, L., & Gross, N. (2015). Herbivore effect traits and their impact on plant community biomass: an experimental test using grasshoppers. Functional Ecology, 29(5), 650-661.

DeWalt, S. J., Bourdy, G., Chavez de Michel, L. R., & Quenevo, C. (1999). Ethnobotany of the Tacana: Quantitative inventories of two permanent plots of Northwestern Bolivia. Economic Botany, 53(3), 237–260. https://doi.org/10.1007/BF02866635

Dostál, P., Fischer, M., Chytrý, M., & Prati, D. (2017). No evidence for larger leaf trait plasticity in ecological generalists compared to specialists. Journal of Biogeography, 44(3), 511-521.

Easdale, T. A., & Healey, J. R. (2009). Resource-use-related traits correlate with population turnover rates, but not stem diameter growth rates, in 29 subtropical montane tree species. Perspectives in Plant Ecology, Evolution and Systematics, 11(3), 203–218. http://dx.doi.org/10.1016/j.ppees.2009.03.001

Edwards, E., Chatelet, D., Sack, L., & Donoghue, M. (2014). Leaf lifespan and the leaf economic spectrum in the context of whole plant architecture. In Journal of Ecology. Dryad Digital Repository. https://doi.org/doi:10.5061/dryad.61g42

Engemann, K., Sandel, B., Boyle, B., Enquist, B. J., Jørgensen, P. M., Kattge, J., ... & Svenning, J. C. (2016). A plant growth form dataset for the New World. Ecology, 97(11), 3243-3243.

Feng, Y., & van, M., Kleunen. (2016). Phylogenetic and functional mechanisms of direct and indirect interactions among alien and native plants. In Journal of Ecology. Dryad Digital Repository. https://doi.org/doi:10.5061/dryad.0672g

Forest Inventory and Analysis National Program. (2013). http://www.fia.fs.fed.us/

Forsyth, D., Scroggie, M., Arthur, A., Lindeman, M., Ramsey, D., McPhee, S., Bloomfield, T., & Stuart, I. (2015). Density-dependent effects of a widespread invasive herbivore on tree survival and biomass during reforestation. In Ecosphere. Dryad Digital Repository. https://doi.org/doi:10.5061/dryad.2q242

Fricke, E. C., & Wright, S. J. (2016). The mechanical defence advantage of small seeds. Ecology letters, 19(8), 987-991.

Gamal, O., El-Dien, Ratcliffe, B., KlA!pste, J., Chen, C., Porth, I., & El-Kassaby, Y. (2015). Prediction accuracies for growth and wood attributes of interior spruce in space using genotyping-by-sequencing. In BMC Genomics. Dryad Digital Repository. https://doi.org/doi:10.5061/dryad.8kb37

Gavinet, J., Prévosto, B., & Fernandez, C. (2016). Introducing resprouters to enhance Mediterranean forest resilience: importance of functional traits to select species according to a gradient of pine density. Journal of Applied Ecology, 53(6), 1735-1745.

Goodman, R., Phillips, O., & Baker, T. (2013). The importance of crown dimensions to improve tropical tree biomass estimates. In Ecological Applications. Dryad Digital Repository. https://doi.org/doi:10.5061/dryad.p281g

Grime, J. P., Hodgson, J. G., & Hunt, R. (2014). Comparative Plant Ecology: A Functional Approach to Common British Species. Springer.

Grootemaat, S., Wright, I. J., van Bodegom, P. M., Cornelissen, J. H., & Cornwell, W. K. (2015). Burn or rot: leaf traits explain why flammability and decomposability are decoupled across species. Functional Ecology, 29(11), 1486-1497.

Hamilton, J. A., Lexer, C., & Aitken, S. N. (2013). Genomic and phenotypic architecture of a spruce hybrid zone (P icea sitchensis× P. glauca). Molecular Ecology, 22(3), 827-841.

Han, W., Fang, J., Guo, D., & Zhang, Y. (2005). Leaf nitrogen and phosphorus stoichiometry across 753 terrestrial plant species in China. New Phytologist, 168(2), 377–385.

Hayes, P., Turner, B. L., Lambers, H., & Laliberté, E. (2014). Foliar nutrient concentrations and resorption efficiency in plants of contrasting nutrient-acquisition strategies along a 2-million-year dune chronosequence. Journal of Ecology, 102(2), 396-410.

He, J.-S., Wang, Z., Wang, X., Schmid, B., Zuo, W., Zhou, M., Zheng, C., Wang, M., & Fang, J. (2006). A test of the generality of leaf trait relationships on the Tibetan Plateau. New Phytologist, 170(4), 835–848.

Hess, L. J., & Austin, A. T. (2014). Pinus ponderosa alters nitrogen dynamics and diminishes the climate footprint in natural ecosystems of Patagonia. Journal of Ecology, 102(3), 610-621.

Ishizuka, W., & Goto, S. (2012). Modeling intraspecific adaptation of Abies sachalinensis to local altitude and responses to global warming, based on a 36-year reciprocal transplant experiment. Evolutionary Applications, 5(3), 229-244.

Karagatzides, J., & Ellison, A. (2008). Construction Costs of Carnivorous Plants and Non-Carnivorous Plants. Harvard Forest Data Archive: HF112.

King, D. A. (1996). Allometry and life history of tropical trees. Journal of Tropical Ecology, 12(01), 25–44.

Kleinschroth, F., Healey, J. R., Sist, P., Mortier, F., & Gourlet-Fleury, S. (2016). How persistent are the impacts of logging roads on Central African forest vegetation?. Journal of Applied Ecology, 53(4), 1127-1137.

Kleyer, M., Bekker, R., Knevel, I., Bakker, J., Thompson, K., Sonnenschein, M., Poschlod, P., Van Groenendael, J., Klimeš, L., Klimešová, J., & others. (2008). The LEDA Traitbase: A database of life-history traits of the Northwest European flora. Journal of Ecology, 96(6), 1266–1274.

Kraft, N. J., Valencia, R., & Ackerly, D. D. (2008). Functional traits and niche-based tree community assembly in an Amazonian forest. Science, 322(5901), 580–582.

Kraft, T., Wright, S., Turner, I., Lucas, P., Oufiero, C., Noor, M., Sun, I., & Dominy, N. (2015). Seed size and the evolution of leaf defences. In Journal of Ecology. Dryad Digital Repository. https://doi.org/doi:10.5061/dryad.69ph0

Leishman, M. R., Cooke, J., & Richardson, D. M. (2014). Evidence for shifts to faster growth strategies in the new ranges of invasive alien plants. Journal of Ecology, 102(6), 1451-1461.

Letcher, S. G., Lasky, J. R., Chazdon, R. L., Norden, N., Wright, S. J., Meave, J. A., ... & Williamson, G. B. (2015). Environmental gradients and the evolution of successional habitat specialization: a test case with 14 Neotropical forest sites. Journal of Ecology, 103(5), 1276-1290.

Li, W., Xu, F., Zheng, S., Taube, F., & Bai, Y. (2016). Patterns and thresholds of grazing-induced changes in community structure and ecosystem functioning: Species-level responses and the critical role of species traits. In Journal of Applied Ecology. Dryad Digital Repository. https://doi.org/doi:10.5061/dryad.9n859

Li, X., Schmid, B., Wang, F., & Paine, C. (2016). Net assimilation rate determines the growth rates of 14 species of subtropical forest trees. In PLOS ONE. Dryad Digital Repository. https://doi.org/doi:10.5061/dryad.5kb61

Liu, K., Eastwood, R., Flynn, S., Turner, R., & Stuppy, W. (2008). Seed information database (release 7.1, May 2008). Available at Ht Tp://Www. Kew. Org/Data/Sid.

Liu, Y., & van Kleunen, M. (2017). Responses of common and rare aliens and natives to nutrient availability and fluctuations. Journal of Ecology, 105(4), 1111-1122.

Louda, S. M., Dixon, P. M., & Huntly, N. J. (1987). Herbivory in sun versus shade at a natural meadow-woodland ecotone in the Rocky Mountains. Plant Ecology, 72(3), 141–149.

Loughnan, D., & Gilbert, B. (2017). Trait-mediated community assembly: Distinguishing the signatures of biotic and abiotic filters. Oikos, 126(8), 1112-1122.

Maire, V., Wright, I. J., Prentice, I. C., Batjes, N. H., Bhaskar, R., van Bodegom, P. M., ... & Santiago, L. S. (2015). Global effects of soil and climate on leaf photosynthetic traits and rates. Global Ecology and Biogeography, 24(6), 706-717.

Manzano-Piedras, E., Marcer, A., Alonso-Blanco, C., & Pico, F. X. (2014). Deciphering the adjustment between environment and life history in annuals: lessons from a geographically-explicit approach in Arabidopsis thaliana. PLoS One, 9(2), e87836.

Martin, A. R., Rapidel, B., Roupsard, O., Van den Meersche, K., de Melo Virginio Filho, E., Barrios, M., & Isaac, M. E. (2017). Intraspecific trait variation across multiple scales: the leaf economics spectrum in coffee. Functional Ecology, 31(3), 604-612.

Marx, H. E., Giblin, D. E., Dunwiddie, P. W., & Tank, D. C. (2016). Deconstructing Darwin's Naturalization Conundrum in the San Juan Islands using community phylogenetics and functional traits. Diversity and Distributions, 22(3), 318-331.

Mason, C. M., & Donovan, L. A. (2015). Evolution of the leaf economics spectrum in herbs: evidence from environmental divergences in leaf physiology across Helianthus (Asteraceae). Evolution, 69(10), 2705-2720.

Mason, C. M., Goolsby, E. W., Humphreys, D. P., & Donovan, L. A. (2016). Phylogenetic structural equation modelling reveals no need for an 'origin'of the leaf economics spectrum. Ecology letters, 19(1), 54-61.

Mazer, S. J. (1989). Ecological, taxonomic, and life history correlates of seed mass among Indiana dune angiosperms. Ecological Monographs, 59(2), 153–175.

McHugh, N., Edmondson, J. L., Gaston, K. J., Leake, J. R., & O'Sullivan, O. S. (2015). Modelling short-rotation coppice and tree planting for urban carbon management–a citywide analysis. Journal of Applied Ecology, 52(5), 1237-1245.

Medrano, M., Herrera, C. M., & Bazaga, P. (2014). Epigenetic variation predicts regional and local intraspecific functional diversity in a perennial herb. Molecular ecology, 23(20), 4926-4938.

Meers, T. L., Kasel, S., Bell, T. L., & Enright, N. J. (2010). Conversion of native forest to exotic Pinus radiata plantation: Response of understorey plant composition using a plant functional trait approach. Forest Ecology and Management, 259(3), 399–409.

Milla, R., Morente-López, J., Alonso-Rodrigo, J. M., Martín-Robles, N., & Stuart Chapin III, F. (2014). Shifts and disruptions in resource-use trait syndromes during the evolution of herbaceous crops. Proceedings of the Royal Society B: Biological Sciences, 281(1793), 20141429.

Molinari, N. A., & D'Antonio, C. M. (2014). Structural, compositional and trait differences between native-and non-native-dominated grassland patches. Functional Ecology, 28(3), 745-754.

Moreno-Gutiérrez, C., Battipaglia, G., Cherubini, P., Delgado Huertas, A., & Querejeta, J. I. (2015). Pine afforestation decreases the long-term performance of understorey shrubs in a semi-arid Mediterranean ecosystem: a stable isotope approach. Functional Ecology, 29(1), 15-25.

Mottet, M. J., DeBlois, J., & Perron, M. (2015). High genetic variation and moderate to high values for genetic parameters of Picea abies resistance to Pissodes strobi. Tree Genetics & Genomes, 11(3), 58.

Murali, K. (1997). Patterns of Seed Size, Germination and Seed Viability of Tropical Tree Species in Southern India. Biotropica, 29(3), 271–279.

Mutete, P., Murepa, R., & Gapare, W. J. (2015). Genetic parameters in subtropical pine F1 hybrids: heritabilities, between-trait correlations and genotype-by-environment interactions. Tree Genetics & Genomes, 11(5), 93.

Neba, G. A., Newbery, D. M., & Chuyong, G. B. (2016). Limitation of seedling growth by potassium and magnesium supply for two ectomycorrhizal tree species of a Central African rain forest and its implication for their recruitment. Ecology and Evolution, 6(1), 125-142.

Nidzgorski, D. A., & Hobbie, S. E. (2016). Urban trees reduce nutrient leaching to groundwater. Ecological Applications, 26(5), 1566-1580.

Niu, K., He, J. S., & Lechowicz, M. J. (2016). Grazing-induced shifts in community functional composition and soil nutrient availability in Tibetan alpine meadows. Journal of Applied Ecology, 53(5), 1554-1564.

Norghauer, J. M., Glauser, G., & Newbery, D. M. (2014). Seedling resistance, tolerance and escape from herbivores: insights from co-dominant canopy tree species in a resource-poor A frican rain forest. Functional Ecology, 28(6), 1426-1439.

Onstein, R. E., Jordan, G. J., Sauquet, H., Weston, P. H., Bouchenak-Khelladi, Y., Carpenter, R. J., & Linder, H. P. (2016). Evolutionary radiations of Proteaceae are triggered by the interaction between traits and climates in open habitats. Global Ecology and Biogeography, 25(10), 1239-1251.

Osuri, A. M., & Sankaran, M. (2016). Seed size predicts community composition and carbon storage potential of tree communities in rain forest fragments in India's Western Ghats. Journal of Applied Ecology, 53(3), 837-845.

Paine, C. T., Amissah, L., Auge, H., Baraloto, C., Baruffol, M., Bourland, N., ... & Hector, A. (2015). Globally, functional traits are weak predictors of juvenile tree growth, and we do not know why. Journal of Ecology, 103(4), 978-989.

Paynter, Q., Buckley, Y. M., Peterson, P., Hugh Gourlay, A., & Fowler, S. V. (2016). Breaking and remaking a seed and seed predator interaction in the introduced range of Scotch Broom (Cytisus scoparius) in New Zealand. Journal of Ecology, 104(1), 182-192.

Peet, R. K., Lee, M. T., Boyle, M. F., Wentworth, T. R., Schafale, M. P., & Weakley, A. S. (2012). Vegetation-plot database of the Carolina Vegetation Survey. Biodiversity and Ecology, 4, 243–253.

Pélabon, C., Hennet, L., Strimbeck, R., Johnson, H., & Armbruster, W. S. (2015). Blossom colour change after pollination provides carbon for developing seeds. Functional Ecology, 29(9), 1137-1143.

Pérez-de-Lis, G., Olano, J. M., Rozas, V., Rossi, S., Vázquez-Ruiz, R. A., & García-González, I. (2017). Environmental conditions and vascular cambium regulate carbon allocation to xylem growth in deciduous oaks. Functional Ecology, 31(3), 592-603.

Ploton, P., Barbier, N., Takoudjou Momo, S., Réjou-Méchain, M., Boyemba Bosela, F., Chuyong, G., ... & Pélissier, R. (2016). Closing a gap in tropical forest biomass estimation: taking crown mass variation into account in pantropical allometries. Biogeosciences, 13(5), 1571-1585.

Poorter, L. (2008). The Relationships of Wood-, Gas- and Water Fractions of Tree Stems to Performance and Life History Variation in Tropical Trees. Annals of Botany, 102(3), 367. https://doi.org/10.1093/aob/mcn103

Poorter, L., & Bongers, F. (2006). Leaf traits are good predictors of plant performance across 53 rain forest species. Ecology, 87(7), 1733–1743.

Poorter, L., & Rozendaal, D. M. (2008). Leaf size and leaf display of thirty-eight tropical tree species. Oecologia, 158(1), 35–46.

Price, C., Wright, I., Ackerly, D., Niinemets, A., Reich, P., & Veneklaas, E. (2014). Are leaf functional traits“invariance” with plant size, and what is “invariance” anyway? In Functional Ecology. Dryad Digital Repository. https://doi.org/doi:10.5061/dryad.r3n45

Prunier, R., Holsinger, K. E., & Carlson, J. E. (2012). The effect of historical legacy on adaptation: do closely related species respond to the environment in the same way?. Journal of Evolutionary Biology, 25(8), 1636-1649.

Ramírez-Valiente, J. A., Lorenzo, Z., Soto, A., Valladares, F., Gil, L., & Aranda, I. (2009). Elucidating the role of genetic drift and natural selection in cork oak differentiation regarding drought tolerance. Molecular Ecology, 18(18), 3803–3815. https://doi.org/10.1111/j.1365-294X.2009.04355.x

Robinson, K. M., Hauzy, C., Loeuille, N., & Albrectsen, B. R. (2015). Relative impacts of environmental variation and evolutionary history on the nestedness and modularity of tree–herbivore networks. Ecology and Evolution, 5(14), 2898-2915.

Rodríguez-Quilón, I., Santos-del-Blanco, L., Serra-Varela, M. J., Koskela, J., González-Martínez, S. C., & Alía, R. (2016). Capturing neutral and adaptive genetic diversity for conservation in a highly structured tree species. Ecological Applications, 26(7), 2254-2266.

Roe, A. D., MacQuarrie, C. J., Gros-Louis, M. C., Simpson, J. D., Lamarche, J., Beardmore, T., ... & Isabel, N. (2014). Fitness dynamics within a poplar hybrid zone: II. Impact of exotic sex on native poplars in an urban jungle. Ecology and evolution, 4(10), 1876-1889.

Royer, D. L., Wilf, P., Janesko, D. A., Kowalski, E. A., & Dilcher, D. L. (2005). Correlations of climate and plant ecology to leaf size and shape: potential proxies for the fossil record. American journal of botany, 92(7), 1141-1151.

Russo, S. E., Jenkins, K. L., Wiser, S. K., Uriarte, M., Duncan, R. P., & Coomes, D. A. (2010). Interspecific relationships among growth, mortality and xylem traits of woody species from New Zealand. Functional Ecology, 24(2), 253–262.

S, M. B., Brad, B., Nathan, C., Rick, C., John, D., M, D. S., Daniel, G., E, H. C., M, J. P., J.B,Maitner, B. S., Boyle, B., Casler, N., Condit, R., Donoghue, J., Durán, S. M., ... & Enquist, B. J. (2018). The bien r package: A tool to access the Botanical Information and Ecology Network (BIEN) database. Methods in Ecology and Evolution, 9(2), 373-379. K. N., Brian, M., Cory, M., Naia, M.-H., K, P. R., Brody, S., Mark, S., A, S. S., Jens-Christian, S., Barbara, T., … J, E. B. (n.d.). The bien r package: A tool to access the Botanical Information and Ecology Network (BIEN) database. Methods in Ecology and Evolution, 9(2), 373–379. https://doi.org/10.1111/2041-210X.12861

Sánchez-Robles, J. M., García-Castaño, J. L., Balao, F., Terrab, A., Navarro-Sampedro, L., Tremetsberger, K., & Talavera, S. (2014). Effects of tree architecture on pollen dispersal and mating patterns in A bies pinsapo Boiss.(Pinaceae). Molecular Ecology, 23(24), 6165-6178.

Schneider, G., Krauss, J., Riedinger, V., Holzschuh, A., & Steffan-Dewenter, I. (2015). Biological pest control and yields depend on spatial and temporal crop cover dynamics. Journal of Applied Ecology, 52(5), 1283-1292.

Shibata, R., Kurokawa, H., Shibata, M., Tanaka, H., Iida, S., Masaki, T., & Nakashizuka, T. (2016). Relationships between resprouting ability, species traits and resource allocation patterns in woody species in a temperate forest. Functional Ecology, 30(7), 1205-1215.

Shugart Jr, H., Hopkins, M., Burgess, I., & Mortlock, A. (1980). Development of a succession model for subtropical rain forest and its application to assess the effects of timber harvest at Wiangaree State Forest, New South Wales. J. Environ. Manage.;(United States), 11(3).

Simpson, K. J., Ripley, B. S., Christin, P. A., Belcher, C. M., Lehmann, C. E., Thomas, G. H., & Osborne, C. P. (2016). Determinants of flammability in savanna grass species. Journal of Ecology, 104(1), 138-148.

Snell-Rood, E. C., Espeset, A., Boser, C. J., White, W. A., & Smykalski, R. (2014). Anthropogenic changes in sodium affect neural and muscle development in butterflies. Proceedings of the National Academy of Sciences, 111(28), 10221-10226.

Spasojevic, M. J., Turner, B. L., & Myers, J. A. (2016). When does intraspecific trait variation contribute to functional beta-diversity?. Journal of Ecology, 104(2), 487-496.

Steane, D. A., Potts, B. M., McLean, E., Prober, S. M., Stock, W. D., Vaillancourt, R. E., & Byrne, M. (2014). Genome?wide scans detect adaptation to aridity in a widespread forest tree species. Molecular Ecology, 23(10), 2500-2513.

Szefer, P., Carmona, C. P., Chmel, K., Konečná, M., Libra, M., Molem, K., ... & Lepš, J. (2017). Determinants of litter decomposition rates in a tropical forest: functional traits, phylogeny and ecological succession. Oikos, 126(8), 1101-1111.

Umaña, M. N., Forero-Montaña, J., Muscarella, R., Nytch, C. J., Thompson, J., Uriarte, M., ... & Swenson, N. G. (2016). Interspecific functional convergence and divergence and intraspecific negative density dependence underlie the seed-to-seedling transition in tropical trees. The American Naturalist, 187(1), 99-109.

Urrutia-Jalabert, R., Malhi, Y., & Lara, A. (2015). The oldest, slowest rainforests in the world? Massive biomass and slow carbon dynamics of Fitzroya cupressoides temperate forests in southern Chile. PLoS One, 10(9), e0137569.

van der Plas, F., Howison, R. A., Mpanza, N., Cromsigt, J. P., & Olff, H. (2016). Different-sized grazers have distinctive effects on plant functional composition of an A frican savannah. Journal of Ecology, 104(3), 864-875.

Vincent, J. B., Weiblen, G. D., & May, G. (2016). Host associations and beta diversity of fungal endophyte communities in New Guinea rainforest trees. Molecular ecology, 25(3), 825-841.

Welsh, M. E., Cronin, J. P., & Mitchell, C. E. (2016). The role of habitat filtering in the leaf economics spectrum and plant susceptibility to pathogen infection. Journal of Ecology, 104(6), 1768-1777.

Weremijewicz, J., & Seto, K. (2016). Mycorrhizas influence functional traits of two tallgrass prairie species. Ecology and Evolution, 6(12), 3977-3990.

Wigley, B. J., Slingsby, J. A., Diaz, S., Bond, W. J., Fritz, H., & Coetsee, C. (2016). Leaf traits of African woody savanna species across climate and soil fertility gradients: evidence for conservative versus acquisitive resource-use strategies. Journal of Ecology, 104(5), 1357-1369.

Wood, Z. T., Peart, D. R., Palmiotto, P. A., Kong, L., & Peart, N. V. (2015). Asymptotic allometry and transition to the canopy in A bies balsamea. Journal of Ecology, 103(6), 1658-1666.

Wright, I. J., Reich, P. B., Westoby, M., Ackerly, D. D., Baruch, Z., Bongers, F., ... & Villar, R. (2004). The worldwide leaf economics spectrum. nature, 428(6985), 821-827.

Yang, X., Xia, H., Wang, W., Wang, F., Su, J., Snow, A. A., & Lu, B. R. (2011). Transgenes for insect resistance reduce herbivory and enhance fecundity in advanced generations of crop–weed hybrids of rice. Evolutionary Applications, 4(5), 672-684.

Zanne, A. E., Oberle, B., Dunham, K. M., Milo, A. M., Walton, M. L., & Young, D. F. (2015). A deteriorating state of affairs: How endogenous and exogenous factors determine plant decay rates. Journal of Ecology, 103(6), 1421-1431.

Zanne, A. E., Tank, D. C., Cornwell, W. K., Eastman, J. M., Smith, S. A., FitzJohn, R. G., ... & Beaulieu, J. M. (2014). Three keys to the radiation of angiosperms into freezing environments. Nature, 506(7486), 89-92.

Zas, R., & Sampedro, L. (2015). Heritability of seed weight in Maritime pine, a relevant trait in the transmission of environmental maternal effects. Heredity, 114(1), 116-124.

Zas, R., Cendán, C., & Sampedro, L. (2013). Mediation of seed provisioning in the transmission of environmental maternal effects in Maritime pine (Pinus pinaster Aiton). Heredity, 111(3), 248-255.

Zheng, S., Ren, H., Lan, Z., Li, W., Wang, K., & Bai, Y. (2010). Effects of grazing on leaf traits and ecosystem functioning in Inner Mongolia grasslands: Scaling from species to community. Biogeosciences, 7(3), 1117–1132.

Zhu, H., Fu, B., Wang, S., Zhu, L., Zhang, L., Jiao, L., & Wang, C. (2015). Reducing soil erosion by improving community functional diversity in semi-arid grasslands. Journal of Applied Ecology, 52(4), 1063-1072.

Zuppinger-Dingley, D., Schmid, B., Petermann, J. S., Yadav, V., De Deyn, G. B., & Flynn, D. F. (2014). Selection for niche differentiation in plant communities increases biodiversity effects. Nature, 515(7525), 108-111.

Züst, T., & Agrawal, A. A. (2016). Population growth and sequestration of plant toxins along a gradient of specialization in four aphid species on the common milkweed Asclepias syriaca. Functional Ecology, 30(4), 547-556.
